# Using Genotyping-By-Sequencing (GBS) for Genomic Discovery in Cultivated Oat

**DOI:** 10.1371/journal.pone.0102448

**Published:** 2014-07-21

**Authors:** Yung-Fen Huang, Jesse A. Poland, Charlene P. Wight, Eric W. Jackson, Nicholas A. Tinker

**Affiliations:** 1 Eastern Cereal and Oilseed Research Centre, Agriculture and Agri-Food Canada, Ottawa, Ontario, Canada; 2 Department of Plant Pathology, Kansas State University, Manhattan, Kansas, United States of America; 3 General Mills Crop Biosciences, Manhattan, Kansas, United States of America; University of California, Riverside, United States of America

## Abstract

Advances in next-generation sequencing offer high-throughput and cost-effective genotyping alternatives, including genotyping-by-sequencing (GBS). Results have shown that this methodology is efficient for genotyping a variety of species, including those with complex genomes. To assess the utility of GBS in cultivated hexaploid oat (*Avena sativa* L.), seven bi-parental mapping populations and diverse inbred lines from breeding programs around the world were studied. We examined technical factors that influence GBS SNP calls, established a workflow that combines two bioinformatics pipelines for GBS SNP calling, and provided a nomenclature for oat GBS loci. The high-throughput GBS system enabled us to place 45,117 loci on an oat consensus map, thus establishing a positional reference for further genomic studies. Using the diversity lines, we estimated that a minimum density of one marker per 2 to 2.8 cM would be required for genome-wide association studies (GWAS), and GBS markers met this density requirement in most chromosome regions. We also demonstrated the utility of GBS in additional diagnostic applications related to oat breeding. We conclude that GBS is a powerful and useful approach, which will have many additional applications in oat breeding and genomic studies.

## Introduction

Cultivated oat (*Avena sativa* L.) is an allohexaploid (2*n* = 6*x* = 42) crop species that is grown as a source of food and feed. Oat and other crop species require continuous genetic improvement to meet the agronomic and nutritional needs of modern agriculture and food production. Major crop species such as corn, rice, wheat, canola, and soybean are benefiting considerably from advances in genome science and molecular breeding. These advances include the discovery and marker-assisted selection of single genes and quantitative trait loci (QTL), as well as the use of genomic selection (GS; [1], [2]) to identify genotypes with superior performance and breeding value. Oat has also benefited from a long history of genomic and nutritional research [3] and from recent advances provided by a SNP platform and consensus map [4]. However, further advancements and applications of genomic technologies that can be integrated into traditional breeding strategies to accelerate and improve the development of superior oat cultivars are needed.

GS can be more efficient than phenotypic selection or marker-assisted selection for improving complex traits [5], and this has been demonstrated in oat [6]. Beyond GS, genomic characterization of breeding material offers many additional opportunities, including: the ability to monitor, maintain and expand germplasm diversity; the ability to diagnose identity or parentage of unknown material; and the ability to discover and deploy specific beneficial alleles [7], [8]. Opportunities also exist for gene discovery, since species such as oat have unique biochemical pathways and adaptations not found in model plant species [9]. There are many technologies that can be applied routinely to whole genome characterization. These include parallel assays that target semi-random polymorphisms, such as Amplified Fragment Length Polymorphism (AFLP) and Diversity Array Technology (DArT), as well as parallel assays for specific single nucleotide polymorphisms (SNPs), such as the Golden Gate assays (Illumina, San Diego, CA). All technologies have strengths and weaknesses. Those that identify semi-random polymorphisms may not provide an adequate density of markers throughout the genome, and the technology may not transfer well between laboratories and different germplasm sets. The application of DArT technology in oat has been successful [10], but only a few hundred of the currently developed markers will segregate in a given population (unpublished results). The recently-developed SNP assay for oat [4] has a similar marker density to the DArT assay, but provides more precise and well-characterized gene-based predictions that may be more uniformly distributed throughout the genome and amenable to comparative mapping.

In oat, as in other polyploid species, genotyping is complicated because of the presence of homoeologous sub-genomes. Markers must be filtered to eliminate those that are confounded by multiple loci. This problem is less prevalent in pre-filtered SNP assays than it is in untargeted assays, although there are still SNP markers known to target different loci in different populations [4]. In all technologies, cost remains a critical factor. Currently, costs for DArT analysis and Illumina-based SNP assays range from $US 50–60 per sample, which can be prohibitive for routine genomics-assisted breeding where a large number of the lines will be discarded following genotyping and selection.

Recently, a robust genotyping method based on the sequencing of partial genome representations has been developed for parallel high-throughput genotyping. This is referred to as genotyping-by-sequencing (GBS) [11]. GBS utilizes one or more restriction enzymes [12] to digest the genome into fragments that are then sequenced by parallel high-throughput methods. Based on the sequencing data, SNP calling can be done using various bioinformatics pipelines [12]–[22]. Low per sample cost in GBS is achieved by multiplexing samples from many (*e.g.*, 48, 96, or 384) different genetic entities (hereafter ‘lines’) simultaneously through the use of short specific ‘barcodes’ ligated to each sample prior to sequencing. Thus, it is possible to reduce the cost per sample by multiplexing more lines for sequencing. For example, if 96 samples are sequenced in a reaction costing $960, the cost per sample would be $10 over and above the costs of sample preparation and bioinformatic analysis. The cost will also be affected by the choice of single-end or paired-end sequencing. GBS has been useful in a variety of applications in crop plants including: saturating an existing genetic map [19], genome characterization in wheat and barley [12], genomic selection in wheat [23], the genetic ordering of a draft genome sequence in barley [24], [25], and the characterization of germplasm diversity in maize and switchgrass [8], [16]. These results suggest that GBS could be utilized for basic and applied genomic studies in oat.

Here, we report the development and application of GBS in oat mapping populations and a diverse set of oat germplasm. Our objectives were: (1) to evaluate the effects of different factors that influence the quality and quantity of GBS SNP calls and establish a baseline of operating parameters and expectations for GBS in oat; (2) to compare alternate methods of bioinformatics analysis and establish a pragmatic workflow and nomenclature for GBS data analysis in oat; (3) to saturate a consensus linkage map with GBS loci and establish a positional reference for future work; and (4) to investigate the utility of GBS to address a variety of questions that are typical of potential uses, including: *de novo* linkage mapping, characterizing population structure and linkage disequilibrium, and solving diagnostic issues in breeding germplasm. We discuss these results in the context of where GBS is likely to be most useful in crop development.

## Materials and Methods

### Genetic materials

Sets of germplasm used in this study are listed in Table 1. Additional diverse oat lines not reported in this study were prepared and sequenced in parallel with this work, which led to a total number of 2,664 oat lines being genotyped with GBS. These samples are mentioned because their presence may have had a minor influence on the parallel sequencing results or global allele-calling pipelines. These effects would be marginal, since more stringent filters were applied within sub-populations.

**Table 1:** Populations and germplasm samples used in this study.

Genetic material	Abbreviation	Number of lines	Reference*	No. of SNP**
Bi-parental mapping populations				
Otana x PI269616 (F_6_)	OxP	98	[4]	17,137
Provena x GS7 (F_8_)	PxG	98	[4]	11,755
Ogle x TAMO-301 (F_6:7_)	OxT	53	[48]	30,726
CDC SolFi x HiFi (F_7_)	CxH	52	[4]	8,324
Hurdal x Z-597 (F_6_)	HxZ	53	[4]	4,219
Kanota x Ogle (F_7_)	KxO	52	[49]	2,582
VAO-44 x Leggett (F_4:5_)	VxL	145	This study	280 (373)
Diversity panels				
Oat diversity panel	IOI	340	[31]	2,155

*First publication that refers to the population

**No. of SNP filtered for subsequent analyses. Please refer to the text for filtering criteria. For VxL, two sets of filtering criteria were used. The only difference between the filtering criteria sets is the heterozygosity level: 8% or 13% (SNP number is between brackets). For IOI, only markers passing filtering criteria with a map position were reported in the table.

### DNA sample preparation

The isolation of DNA was performed using a variety of methods, as some samples were available from previous studies. The preparation of DNA stocks from the CxH, HxZ, OxT, OxP, and PxG populations was described by Oliver *et al.* [4], while stocks from the KxO population were prepared as described by Wight *et al.* [26]. The latter stocks still contained RNA, which was removed using a standard RNase procedure followed by phenol/chlorofrom extraction and ethanol precipitation.

For the diversity population (IOI panel), eight seeds of each line were germinated in cyg growth pouches (Mega International, Minneapolis, MN, USA). Leaf tissue was harvested in bulk as the second leaves emerged and was put into paper envelopes containing a 5:1 mix of non-indicating and indicating silica gel desiccant. The paper envelopes were then placed in sealed containers for drying. For the VxL population, leaf tissue was harvested from plants growing in the field, then dried in the same manner. DNA was extracted from the VxL and IOI leaf samples using DNeasy Plant Maxi kits (Qiagen Inc., Mississauga, ON, Canada).

### GBS library preparation and sequencing

The GBS libraries were constructed in 95-plex using the P384A adapter set (Table S2 in [12]). For each plate, a single random blank well was included for quality control to ensure that libraries were not switched during construction, sequencing, and analysis. Genomic DNA was co-digested with the restriction enzymes *Pst*I (CTGCAG) and *Msp*I (CCGG) and barcoded adapters were ligated to individual samples. Samples were pooled by plate into libraries and polymerase chain reaction-amplified. Detailed protocols can be found in [23]. Each 95-plex library was sequenced to 100 bp on a single lane of Illumina HiSeq 2000 or HiSeq 2500 by the DNA Technologies core facility at the National Research Council, Saskatoon, SK, Canada.

### UNEAK GBS pipeline

Sequence results were analysed using the UNEAK GBS pipeline [16], which is part of the TASSEL 3.0 bioinformatics analysis package [27]. This method does not require a reference sequence, since SNP discovery is performed directly within pairs of matched sequence tags and filtered through network analysis. In this method, tags (a tag is an unique sequence representing a group of reads) belonging to complex multi-locus families (as determined by network analysis) are ignored. Parameters in the UNEAK pipeline were set for maximum number of expected reads per sequence file (300,000,000), restriction enzymes used for library construction (*Pst*I*-Msp*I), minimum number of tags required for output (10), maximum tag number in the merged tag counts (200,000,000), option to merge multiple samples per line (yes), error tolerance rate (0.02), minimum/maximum minor allele frequencies (MAF, 0.02 and 0.5), and minimum/maximum call rates (0 and 1). Call rate is defined as the proportion of samples that are covered by at least one tag. The MAF and call rate were set at a low value for global analysis because these parameters were filtered within sub-populations in later steps.

### GBS pipeline using population-level filter

A second SNP calling pipeline was employed as described by Poland *et al.* [23]. This pipeline is implemented in TASSEL 3 and was functionally identical to UNEAK to the point of developing a binary presence/absence matrix for each tag across multiple lines. To identify putative SNPs, tags were internally aligned allowing up to 3 bp mismatch in a 64 bp tag. From aligned tags, SNP alleles were identified and the number of lines in the population with each respective tag was tallied in a 2×2 table, counting the number of lines with one or the other tag, both, or neither [23]. A Fisher Exact Test was then used to determine if the two alleles were independent, as would be expected for a single locus, bi-allelic SNP in a population of inbred lines. If the null hypothesis of independence for the putative SNP was rejected (*p*<0.001), we assumed that the tags were allelic in the population (and, therefore, that the putative SNP was a true single locus, bi-allelic SNP). A significance threshold of *p*<0.001 was selected for the size of population, based on previous work testing false discovery rates in duplicate samples.

### Filtering and merging GBS SNP calls

Both of the above pipelines were applied globally to all available sequencing data, except where we deliberately tested SNP identification in partial datasets. This global strategy reduced the need to access large sequencing files repeatedly. However, there was then a need to generate genotype data for specific sub-populations, and to apply population-specific filters for allele frequency, heterozygosity, and data completeness (data completeness is defined as 100% - % missing data; *e.g.*, for a marker genotyped on 100 individuals with 10 individuals showing missing data points, the completeness of the marker is 90%). Furthermore, for genotypes of mapping progeny, it was necessary to recognize the parental phase of alleles, and to represent alleles using conventions required by the mapping software. These filters and secondary analyses were applied using in-house software (‘CbyT’) written in the Pascal programing language (Text S1). This software provided the additional feature of maintaining a cumulative index of unique SNPs with a consistent naming convention, such that data from different pipelines or subsequent assays could be merged to remove redundancy and to index matching SNPs with the same unique name. Each subsequent analysis required specific filtering criteria, which can be found at the beginning of the method section for each type of analysis.

### Linkage mapping

For bi-parental mapping populations, parental lines were genotyped together with the progeny. GBS loci called using both pipelines across six bi-parental RIL populations (OxP, PxG, OxT, CxH, HxZ, and KxO) were filtered at ≥50% completeness, MAF ≥35%, and heterozygosity ≤8% inside each population, which gave a total of 45,117 GBS markers. The SNP data from the six mapping populations reported by Oliver *et al.* [4] were filtered to the same standards as the GBS SNP data, and the two data types concatenated. Marker phases were determined using parental genotypes when the latter were available and not monomorphic. Monomorphic parental genotypes can result from genotyping errors or genetic variation within the lines used to make the cross. Markers for which there were no good parental data were converted into both parental phases for further analysis. For each mapping population, the phase of parental alleles was re-checked across the concatenated data by enumerating, for each SNP, the number of linked loci in the same phase (recombination fraction, *r*<20%) *vs.* the number in opposite phase (*r*>80%). Loci having a greater number of out-of-phase matches than in-phase matches were rescored in the opposite phase, or were eliminated through a recursive process if this did not improve the in-phase/out-of-phase ratio.

An updated version of the oat consensus map developed by Oliver *et al.* [4] was generated by placing each new candidate locus (GBS or other non-framework SNP) relative to framework SNPs from the existing map. Pair-wise recombination fraction (*rf*) was first calculated for all marker pairs, including both framework and non-framework markers. Marker placements were then made relative to the two framework loci showing the smallest *rf* among any of the six populations. The approximate map position of each placed marker was subsequently estimated by interpolating the cM position proportional to the recombination fraction with the closest two framework loci. When the closest framework locus was at the end of a linkage group, and the recombination with the next-closest framework locus was greater than that between the two framework loci, the candidate was placed distal to the end of the linkage group. In addition to this crude approximation of marker position, a detailed report of each placed marker was produced to show the actual recombination frequencies within each population and across populations between a given marker and all other loci that were within 20% recombination in any of the component populations. Marker data used for marker placement on the oat consensus map are in Table S1.

*De novo* linkage map construction was performed using MSTMap [28] for the VxL population. GBS loci for *de novo* map construction were called using the UNEAK GBS pipeline and filtered at high stringency (MAF ≥35%, completeness ≥90%) at two different levels of heterozygosity (8% and 13%). The resulting data contained 858 (heterozygosity ≥8%) and 1053 (heterozygosity ≥13%) GBS markers. The choices of 8% and 13% corresponded to the expected heterozygosity at F_5_ and F_4_, respectively, factoring in sequencing error and out-crossing rate. For MSTMap, a *p*-value equal to 10^−11^ was used for the marker clustering threshold. Markers were excluded as unlinked if they were 15 cM away from any other locus or if they belonged to a group containing only two loci. A simple recombination count was used for the objective function. Since map distances estimated by MSTMap are inflated (based on simulated data, result not shown), we re-estimated the recombination fractions between pairs of loci based on the marker order from MSTMap and converted them to map distances using the Kosambi mapping function.

### Population structure and LD analysis

For population structure and LD analysis, GBS markers called by the UNEAK pipeline were filtered at ≥90% completeness, MAF ≥5%, and heterozygosity ≤5%. Population structure was investigated using principal component analysis (PCA). PCA was performed with the ‘smartpca’ function implemented in EIGENSOFT [29]. This function takes into account marker dependency (*i.e.*, markers in LD blocks) through the use of multiple-regression on adjacent markers prior to PCA. The maximum interval distance between markers (ldlimit) was set to 0.001. The number of adjacent markers included in LD adjustment (ldregress) was set at 0, 10, or 50 (designated k0, k10, and k50), such that k0 provided no LD correction and k10 and k50 corresponded to the median and maximum LD block sizes in the IOI dataset. Eigenvalues and Eigenvectors were transferred to the R statistical package [30] for scree plot drawing and other analyses.

A model-based approach was used to investigate the clustering pattern among lines in the diversity panel further, because it determines simultaneously the number of clusters and cluster membership and does not have underlying genetic assumptions that are rarely met [31]. Model-based clustering was based on the first ten PC and conducted using the clustCombi function of the R package mclust [32]. The purpose of clustCombi is to represent a non-Gaussian cluster by a mixture of two or more Gaussian distributions [33]. It first uses the Bayesian information criterion (BIC) to identify the number of Gaussian mixture components and then hierarchically combines components according to an entropy criterion. The final decision concerning the number of clusters to use was made based on an entropy plot; *e.g.*, if six components were identified by BIC and successive component combinations showed no large entropy decrease after four clusters, then the data were represented by four clusters.

Linkage disequilibrium (LD) between two loci was estimated as squared allele-frequency correlations (*r^2^*) by an optimized version (Stéphane Nicolas, personal communication) of LD.Measure in the R package LDcorSV [34]. Four *r^2^* estimates were calculated: conventional *r^2^* based on raw genotype data, *r^2^* with population structure included in the calculation (*r_s_^2^*), *r^2^* with relatedness included in the calculation (*r_v_^2^*), and *r^2^* with both population structure and relatedness included (*r_sv_^2^*). Population structure was represented by the first four PC after scaling the coordinate identifiers across a range of zero to one. A matrix of relatedness was calculated by A.mat, implemented in the rrBLUP package [35].

The relationship between LD and genetic distance was modeled by fitting two alternate non-linear regression models: a drift-recombination equilibrium model [36] or a modified recombination-drift model including low level of mutation and an adjustment for sample size [37]. Both models were summarized in [38].

### Other statistical analyses

We wished to examine how GBS technology could be used to solve diagnostic problems that arise occasionally in any plant breeding program. Germplasm diagnostics were performed using DARwin software [39] to generate clusters based on genetic distances among lines, estimated using simple allele-matching for bi-allelic diploid loci:
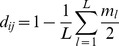
where *d_ij_* is the dissimilarity between lines *i* and *j*, *L* is the number of informative loci shared by those lines, and *m_l_* is the number of matching alleles for locus *l*. Cluster analysis was performed using the un-weighted paired group mean analysis (UPGMA) method.

## Results

### Library construction, sequencing and coverage

For this study, a total of 38 libraries were generated, each multiplexing 95 lines. A single lane of Illumina HiSeq 2000 or HiSeq 2500 was used to sequence each library. The GBS libraries were constructed as previously described for wheat, with the exception that the forward barcode adapter concentration was reduced to 0.06 pmol for 200 ng of genomic DNA (*vs.* 0.1 pmol used for wheat in [12]). This adapter concentration was found to improve the oat libraries, reducing adapter dimers.

A complete set of short read archives for all GBS oat samples analysed to date has been made available for download from the NCBI short read archive (http://www.ncbi.nlm.nih.gov/sra/) under project accession number SRP037730. Details of these archives, including number of reads and number of good barcoded reads at the level of each flow-cell, single lane, and individual taxon are available in Table S4. Table S4 also provides the key file needed to support re-analysis of the raw short read archives by either of the GBS pipelines reported here.

From a total of 6.3×10^9^ reads, 84.4% (5.3×10^9^) included the barcode sequence and enzyme cut-site, and had no unreadable base (‘N’) in the sequence. The UNEAK pipeline found an average of 732,396 tags per oat line in the samples reported here, a total merged tag count across all samples of 358,177,647 tags, and a filtered tag count (tags appearing >10 times) of 17,700,128 that were covered by 564,946,411 matching reads.

**Figure 1:**
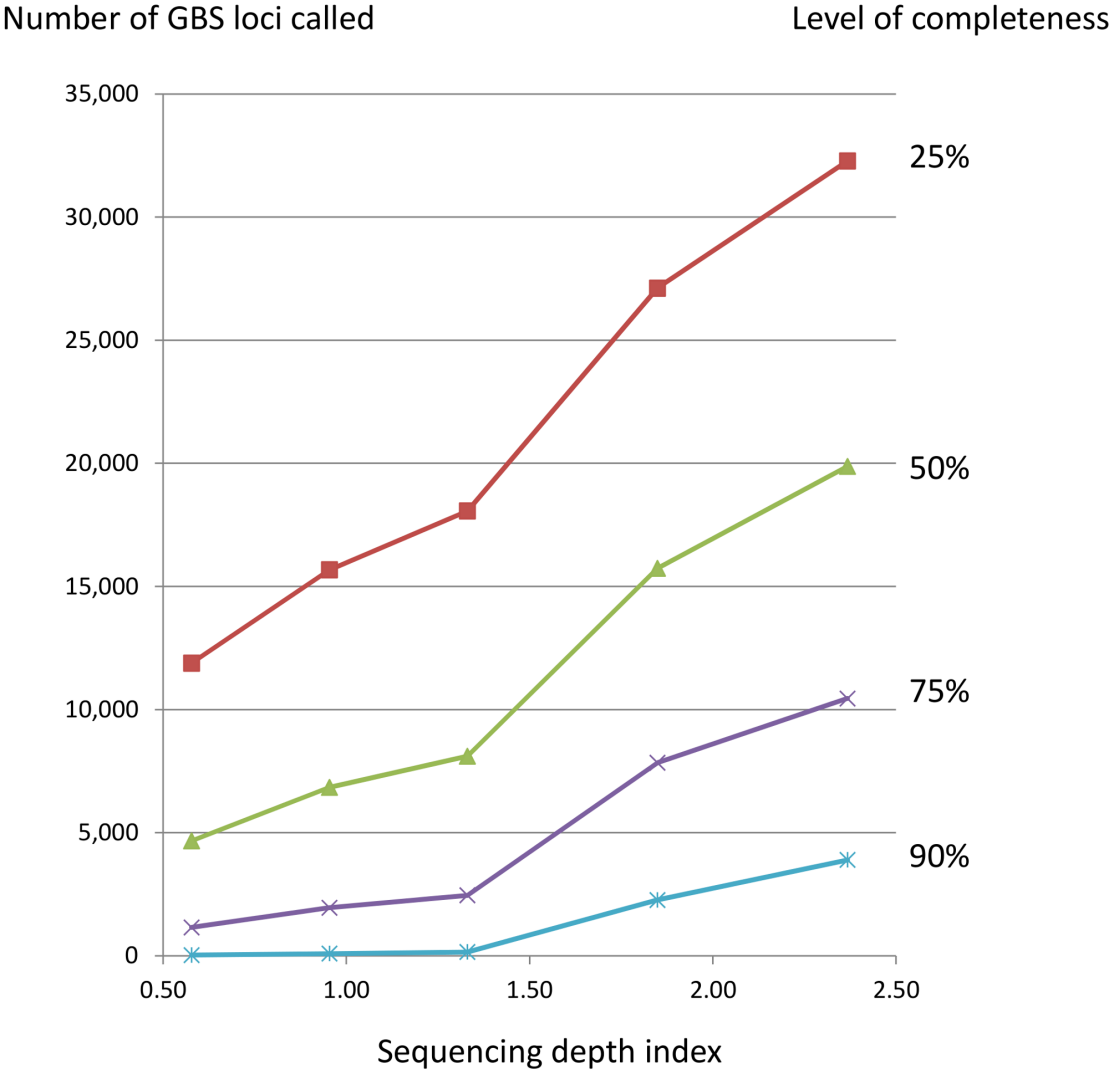
Number of GBS loci *vs.* sequencing depth. Number of GBS SNP loci called in 53 OxT mapping progeny at increasing sequencing depth, filtered at four levels of completeness (25%, 50%, 75%, and 90%). Other filtering parameters were constant, with heterozygosity ≤10% and minor allele frequency ≥30%. A sequencing depth index of 1 represents the average read depth that would be achieved with 95 samples multiplexed in a standard Illumina sequencing run giving approximately 2×10^8^ short reads. Thus, an index of 2 would be equivalent to twice this number of reads or half of this plexity.

Each sequencing lane generated approximately 2×10^8^ 100 bp reads. After discarding reads that did not have an exact match to one of the barcodes, there were approximately 2×10^6^ 100 bp reads per sequenced DNA sample. We designated the 2×10^6^ 100 bp-base reads/DNA sample as one unit of ‘depth index’. To test the influence of plexity and sequencing depth on GBS data completeness, we used data from 53 OxT mapping progeny sequenced in three separate lanes. We split the raw sequencing data from two lanes in half and added these incrementally to the un-split lane, which contained a lower number of reads. This provided five different sequencing depths with mixed levels of plexity, from which we computed average depth indices of 0.58, 0.95, 1.33, 1.85, and 2.37. For example, the depth index of 0.58 means there were, on average, 0.58×2×10^6^ = 1.16×10^6^ reads/sample for that experiment. The exact read depth for individual samples varied because of sample quality and/or variations in barcode efficiencies. The UNEAK GBS pipeline was run on these five data subsets and SNPs were filtered at four levels of completeness (25%, 50%, 75%, and 90%). The results (Figure 1) showed that an increasing number of SNPs were called as the depth index was increased at all four completeness levels. The response to sequencing depth appears to be linear within the range tested. One of the sequencing runs, added at the second and third levels, had a higher variation in read depth among samples, which explains the lower slope at these levels and the fact that almost no SNPs had a completeness of 90%.

### Population size *vs*. number of GBS SNPs

**Figure 2:**
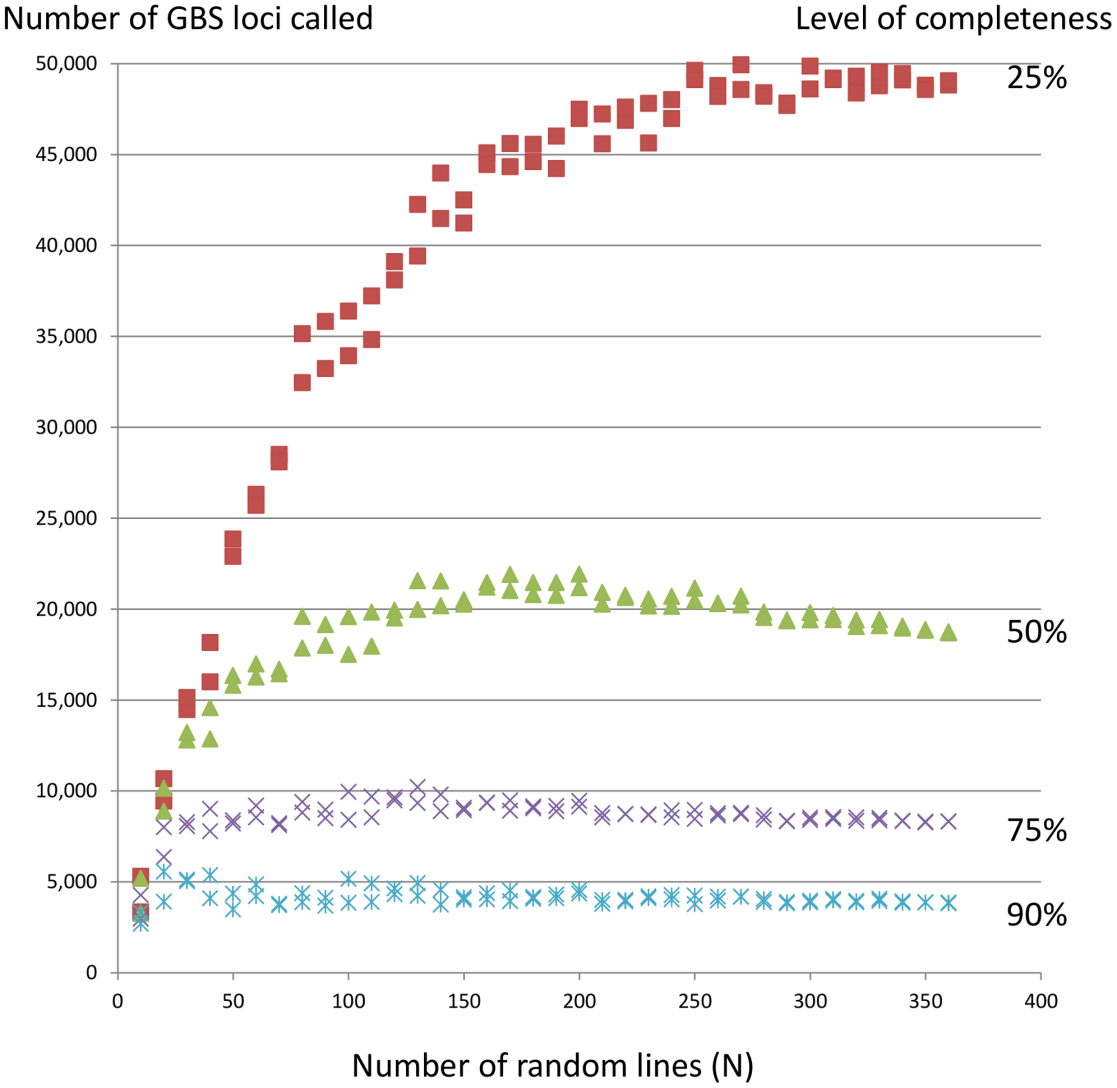
Number of GBS loci *vs.* sample size. Number of GBS loci called in samples of size *N* from a set of 360 diverse oat lines filtered at four levels of completeness (25%, 50%, 75%, and 90%) is shown. Other filtering parameters were constant, with heterozygosity ≥10% and MAF ≥5%.

We examined random subsets of 366 diverse oat varieties, including the IOI set and 26 additional winter oat varieties, to determine how sample size would affect the number of GBS SNPs called at differing levels of completeness. Sample size was varied between 10 and 360 at increments of 10, with two randomly chosen subsets as replicates for each sample size. These data were filtered at a maximum heterozygosity of 10%, MAF of 5%, and minimum completeness of 25%, 50%, 75%, or 90%. At each sample size, the number of SNPs passing these filters was counted. At a low threshold for completeness (25%), the number of SNPs increased with sample size, plateauing at approximately 50,000 SNPs once 250 of the 360 oat lines had been included (Figure 2). At higher thresholds for completeness (50%, 75%, and 90%), the number of SNPs plateaued at approximately 20,000, 10,000, and 5,000 SNPs, respectively. These plateaus occurred at increasingly smaller sample sizes, and the number of SNPs appeared to decrease slightly as sample size increased beyond the initial plateau.

### Multi-allelic SNPs

Although multi-allelic loci were not specifically identified by either of the two GBS pipelines employed in this work, it was apparent that the population-based method would frequently call separate pairs of bi-allelic SNPs at the same site, providing evidence for third and, occasionally, fourth alleles. We used this result to make an approximate estimate of the frequency at which multi-allelic SNPs can be identified from existing pipelines. Of the 355,731 unique bi-allelic SNPs from all oat projects called using both pipelines, there were 343 sets of tri-nucleotide SNPs (*i.e.*, two bi-allelic SNPs having identical context sequence except for a third allele at the SNP position), and 21 sets of tetra-nucleotide SNPs (*i.e.* two bi-allelic SNPs having identical context sequence except for the SNP position). This analysis was not expected to give comprehensive access to all multi-allelic SNPs, since most of the multi-allelic SNPs would have been filtered out during SNP calling. However, this result suggests that tri- and tetra-nucleotide SNPs are extremely rare, which is expected when SNPs arise primarily as random neutral mutations.

### Integration of GBS SNPs with an existing genetic consensus map

45,117 GBS loci, filtered across six bi-parental RIL populations, were placed on the oat consensus map of Oliver *et al.* [4] based on simple counts of recombination fractions. Starting from the initial consensus map, each additional population provided from 2,535 (KxO) to 30,369 (OxT) more loci (Figure S16). As more populations were used for marker placement, fewer new markers were added to the map, but the number of new markers was always proportional to the number of markers available from the source population; *e.g.*, there were always more new loci from OxT (21,894–30,369) than KxO (1,065–2,535) (Figure S16). The complete report for this map is available as a supplementary HTML file in Text S2 (or online at: http://ahoy.aowc.ca/html_link_gbs_text_S2.html). The report format is described in Figure S1. Each individual marker on this HTML map is linked to a separate, detailed report that shows a complete matrix of recombination fractions between the reported marker and the neighbouring loci from the consensus map, as well as other placed markers across all populations.

**Figure 3:**
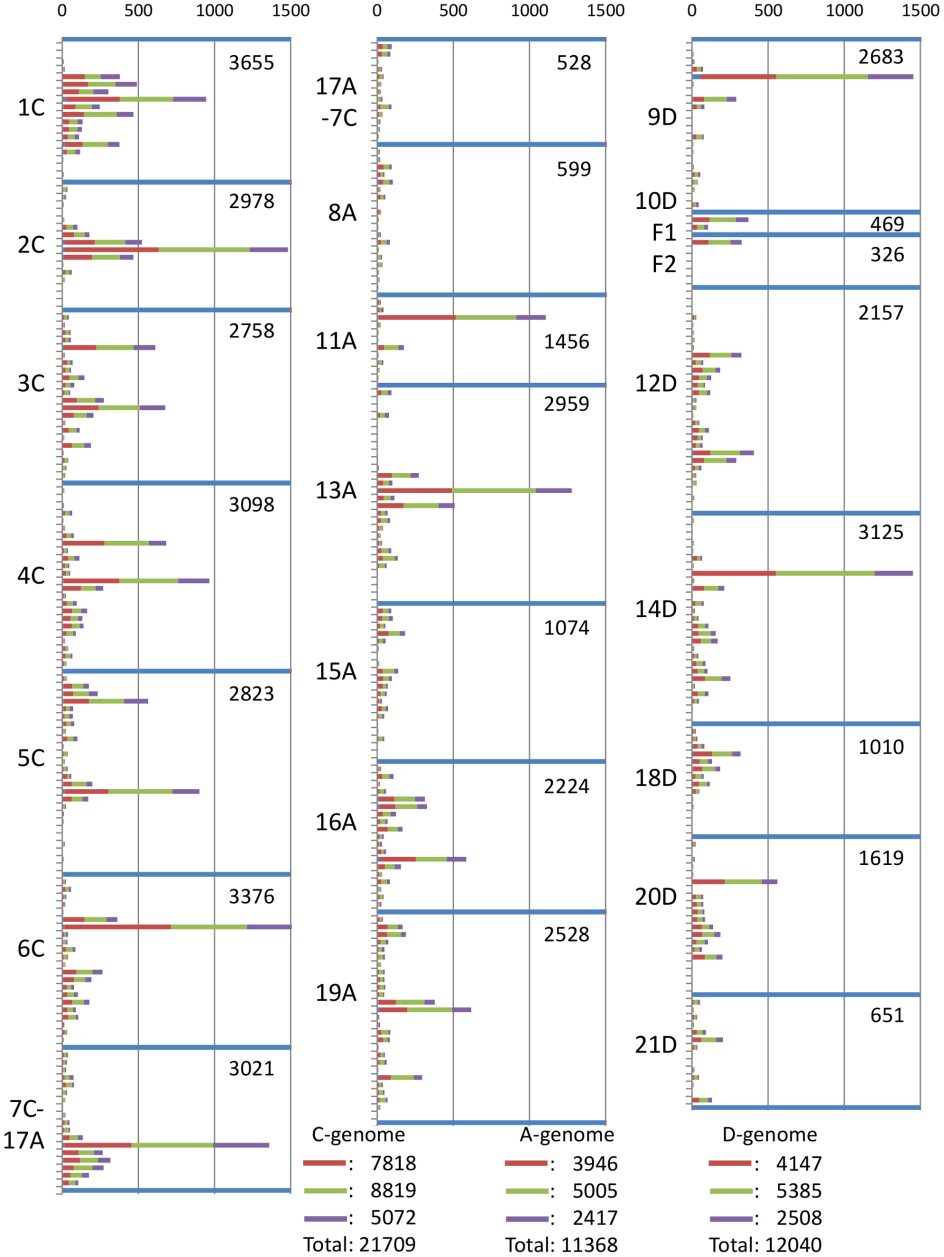
Distribution of GBS loci across the oat genome. Maps of each chromosome (delineated by blue lines and labeled on left) are divided into 5 cM bins with 0 cM starting at the top. Red bars show numbers of loci detected by two pipelines, green shows those detected only by the population-filtering pipeline, and violet shows those detected only by the UNEAK pipeline. Numerals inside boxes show total GBS loci by chromosome. A summary of placed GBS loci by pipeline and by sub-genome is shown.

To investigate the approximate global distribution of GBS loci relative to the SNP consensus map, we divided the map into bins of 5 cM intervals and produced a density histogram which showed the number of placed GBS loci in each bin along the genome (Figure 3). Some bins contained no markers, while some contained a much larger number of markers. In general, GBS loci tended to cluster in the same locations as array-based SNPs. Some clusters probably reflect centromeres, where suppressed recombination causes genetic clustering. However, some chromosomes contained multiple regions of clustering, especially 3C, 4C, 5C, 16A, 19A, 12D, and 21D. This may be caused in part by cytogenetic differences among the parents of the mapping populations, whereby individual maps contain underlying differences in the structure and order of genetic markers. The consensus map would have compressed these differences into a single ‘average’ map, but the underlying differences among populations remain, and placed markers may appear to cluster at points where the consensus has averaged these differences.

The inclusion of GBS markers appeared to fill gaps within the consensus map. For example, 112 marker intervals larger than 5 cM were present on the original consensus map [4], while only 25 are present on the same map once GBS markers are placed, and the maximum gap size decreased from 26.98 cM to 15.74 cM (Figure S2). These results do need to be interpreted with caution, because the interpolated positions of markers with miss-scored alleles may appear to fill some gaps. An accurate re-interpretation of the consensus map can only be achieved by a complete reanalysis and reinterpretation of the component maps. This work is in progress and will be reported elsewhere together with a complete report on additional SNP loci. Preliminary results of this work (unpublished data) indicate that GBS markers fill some gaps, but that their greatest benefit is to increase the number of loci that are mapped in multiple populations.

### Examination of orthology with other crops

The high density of approximately-placed GBS markers provides a new opportunity to examine orthologous relationships between oat and model genomes. The orthology analysis was performed to determine whether matches of short GBS sequence to model genomes would be sufficient to identify major regions of genome co-linearity. Using dot-plots, we explored the locations of sequence similarity between the oat consensus map and pseudomolecule sequence assemblies from *Brachypodium distachyon* L. (Bd), rice, and barley (Figures S3 to S6). As in a previous analysis using only array-based SNPs [4], we observed that *Brachypodium* had a greater number of matches and better colinearity with oat than did rice. Several stretches of colinearity were observed, such as those on oat 19A (similar to parts of chromosomes Bd1, Bd2, and rice1) and oat 20D (similar to Bd5 and rice4). In the current analysis, it is clear that, in regions of collinear sequence-based matching, both the GBS and other SNPs are contributing similar information. In some cases, the GBS loci appeared to extend the regions of colinearity (for an example, see Bd1 and Bd4 in Figure S3). It was also clear from the higher density of GBS matches to *Brachypodium* sequences that a greater number of non-coding sequences were similar between *Brachypodium* and oat than between rice and oat, despite the larger genome of rice. This is probably because of the closer ancestral relationship between oat and *Brachypodium*. Barley showed very poor colinearity with oat, based on sequence matching to the newly-available ordered shotgun assembly [25]. We suspect this to be a result of the incomplete nature of the barley assembly. It was also notable that many GBS SNPs showed highly repetitive matches to the barley genome (Figure S5), which were mostly eliminated by removing GBS loci determined to have multiple matches to other GBS loci within oat (Figure S6). This suggests that there are many repetitive elements that are shared between the oat and barley genomes.

### GBS SNP annotation

In order to give an approximation of the number and distribution of genic and intergenic GBS SNPs in oat, we compared a complete set of 355,731 context tags of GBS SNPs from all oat projects available at the time of analysis to the chromosome-based genome assembly and accompanying gene predictions from *Brachypodium* (release 2.1; http://www.brachypodium.org) by BLAST. Of these, 19,656 tags (5.5%) showed protein matches (BLASTx) with expectation <0.1, a level that corresponds approximately to a minimum 60% identity over the full tag length or 100% identity over half the tag length. Although there will be oat genes that do not have *Brachypodium* orthologues, it is still likely that fewer than 5% of GBS tags are within transcribed oat genes, because many of the protein signatures matching *Brachypodium* will likely represent vestigial genes in oat. Of the tags with protein matches, 16,712 showed DNA similarity within the transcribed region at the threshold expectation of <0.1. However, we noted that the average BLASTn expectation corresponding with a BLASTx expectation of 0.1 was approximately 0.001; therefore, we conducted further nucleotide matches at this level. This allowed us to identify a total of 46,370 tags (13% of total) with nucleotide matches in *Brachypodium*, among which 30,713 (66% out of matched tags or 8.6% out of total tags) were in intergenic regions, and 15,657 (34% of matched tags or 4.4% of total tags) inside gene regions, of which only 300 did not match a protein. Since gene regions in *Brachypodium* correspond to approximately 43% of the genome, it appears that there is some bias toward GBS nucleotide matches outside of gene regions.

Out of the 46,370 tags matched to *Brachypodium* sequences, 9,684 were positioned somewhere on the consensus map (*cf.* Results/Integration of GBS SNPs with an existing genetic consensus map). On average, 20.5% of mapped loci were positioned inside a gene, ranging from 14.2% to 29.08%, which is a slightly smaller than the proportion in overall tags having BLAST matches to the *Brachypodium* genome. The distribution of *Brachypodium* orthologous SNPs along the oat genome is similar to that of the overall oat GBS SNPs (Figure S17 and Figure 3). Genic and intergenic SNP counts per chromosome and their genome distribution can be found in Figure S17. While this result gives an approximation of the proportion and distribution of genic and intergenic SNPs along the oat genome, care should be taken in interpreting this result to form a general conclusion about oat, not just because of the partial coverage of the present consensus map, but also because of the approximate nature of the annotations made through the use of orthologues.

### A *de novo* genetic linkage map using GBS

In the above work, mapping was performed in six populations of reduced size, primarily to approximate the positions of a large inventory of GBS loci relative to an oat consensus map. We also wished to examine the utility of GBS markers for generating a *de novo* linkage map in the absence of other marker types, and to evaluate how well this map could be matched to the current consensus. For this work, we used the VxL population, composed of 145 F_4:5_ RIL families. The GBS loci for map construction were called using the UNEAK GBS pipeline and filtered at high stringency (MAF ≥35%, completeness ≥90%) at two different levels of heterozygosity (8% and 13%). The resulting data contained 858 (heterozygosity ≥8%) and 1053 (heterozygosity ≥13%) GBS loci. From this, a map with 35 linkage groups having a total length of 1713 cM was constructed. A comparison between this map and the consensus (Figure S7) showed that 280 (heterozygosity ≥8%) or 373 (heterozygosity ≥13%) markers were present in VxL but not in the six mapping populations used for consensus map saturation. Most VxL linkage groups corresponded to single consensus chromosomes, and the relative positioning of loci within groups was approximately linear. Several sets of VxL linkage groups (*e.g*., LG07 and LG20) likely represent single oat chromosomes (in this case, 12D). This suggests that there is good opportunity to perform comparative mapping of traits that are identified in new populations using only GBS technology. The filtering of loci at different levels of heterozygosity provided an opportunity to observe that certain regions of the VxL genetic map are more highly heterozygous than others (red dots in Figure S7, and graphical genotypes in Table S2), as would be expected in an F_4_ population. In addition, most of the heterozygous loci were clustered at what are likely centromeres (Figure 3 and Figure S7), explained by the fact that low recombination in centromeric regions has been found to contribute to the retention of residual heterozygosity [40]. This provides good evidence that heterozygous genotype calls in the GBS pipeline are generally accurate and genetically consistent.

### Use of GBS to evaluate population structure

PCA and model-based clustering were used to examine the effectiveness of GBS markers to identify population structure in 340 oat lines of global origin. Of these, 41% originated from North American breeding programs (81 lines from Canada and 59 from USA) and the remainder originated elsewhere (Table S3). GBS markers called by the UNEAK pipeline were filtered at ≥90% completeness, MAF ≥5%, and heterozygosity ≤5%. Of the filtered SNPs, only those that were placed on the consensus map (2155 loci) were considered. Because of genetic clustering, a large number of these SNP loci (1159, or 54%) co-segregated at identical positions, and 1755 (81%) were within 1 cM intervals (Figure S8). Since this dependency is also reflected in LD (see next section), it was likely to distort the Eigenvector/Eigenvalues and to bias the interpretation of population structure [29]. Therefore, we applied two levels of LD correction (k10 and k50), in addition to using an uncorrected analysis (k0) for PCA. At k0, no obvious reflection point was observed in the scree plot, while we could distinguish slight two-stage plateaus in k10 and k50, where the drop of Eigenvalues slowed at approximately PC5 and PC10 (Figure S9). The first ten PC explained 37.6, 32.1, and 31.6 percent of the total variation for k0, k10, and k50, respectively, whereas 25.0, 21.2, and 20.9 percent of the total variation was explained by the first four PC (the approximate point of the first plateau).

**Figure 4:**
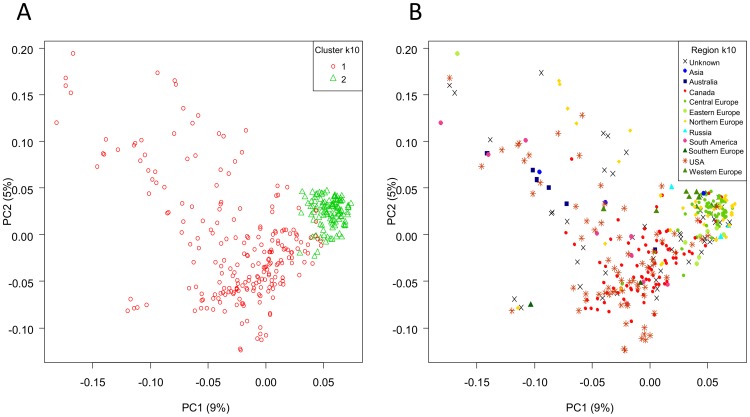
Scatter plots of PC1 *vs*. PC2. The k10 correction is shown: (A) coloured based on clustering from genotypic data, (B) coloured based on geographic origins.

Since no obvious groups were separated by the first two PCs, model-based clustering was performed to explore the grouping of oat lines based on the first ten PC. The best solutions for k0, k10 and k50 were four, two, and two clusters, although the entropy plot of k50 could be understood as three clusters (Figure S10). The clusterings from k0, k10, and k50 were generally in agreement and reflected the geographic origins of the oat lines, with European lines tightly clustered together and lines from elsewhere spread out across the plot (Figures 4 and S11). The possible third cluster in k50 was positioned between European and North American lines and was comprised of oat lines from Eastern or Northern Europe, as well as some North American lines. One set of five lines was separated by PC4 and this separation is particularly obvious in the k0 data set (Figure S12). The separation of this cluster seemed to be related to growth habit (three of the five lines are winter oats, Figure S12), but because the number of lines was so small, a definitive conclusion could not be made. Our results showed that this diversity panel does not show substantial structural stratification, and this is in agreement with previous work based on DArT markers [31].

### Linkage disequilibrium analysis

**Figure 5:**
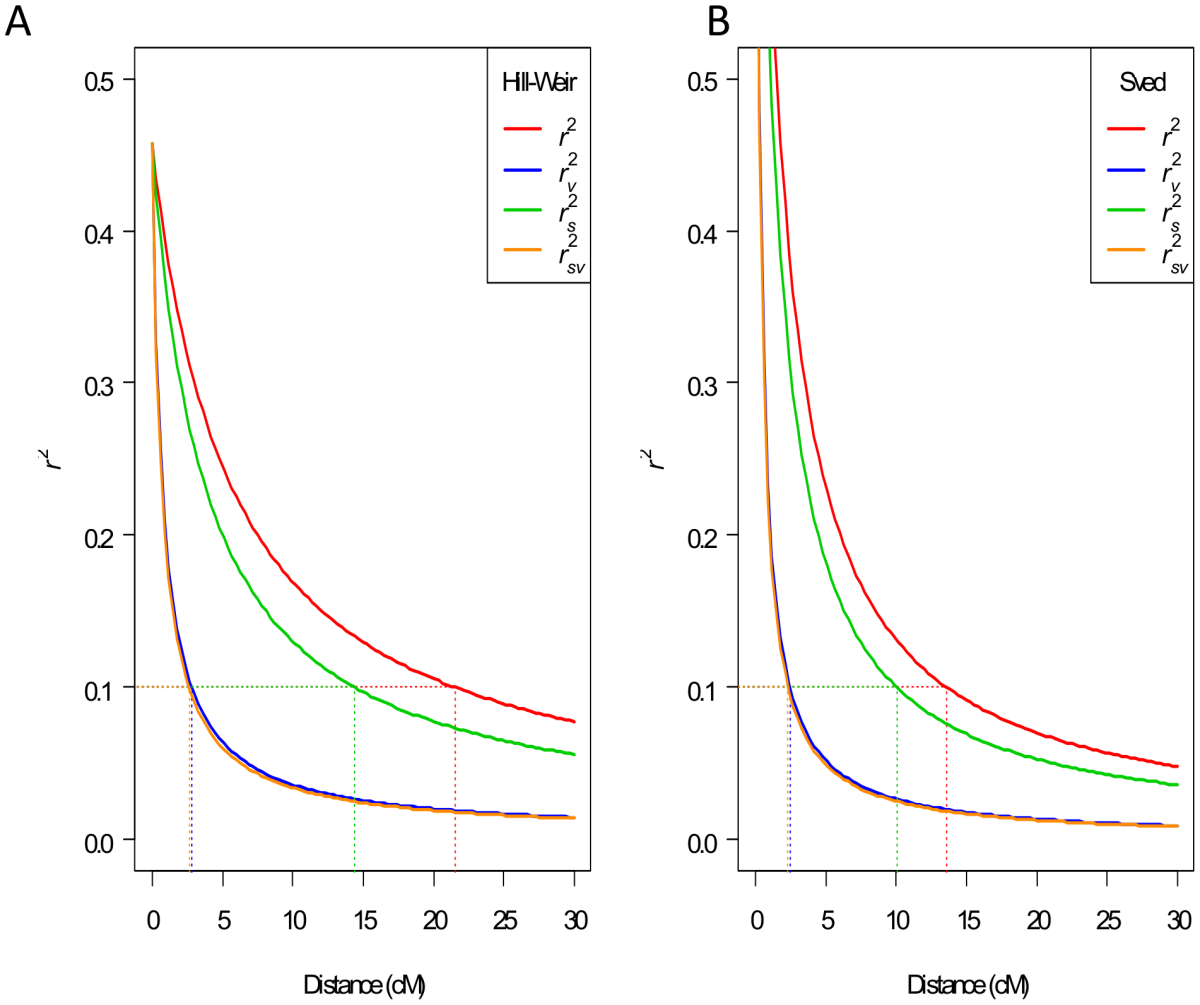
LD decay plot. *r^2^* estimates were plotted against the average map distance (recombination frequency expressed in cM): (A) relationship fit using the mutation model (Hill-Weir), (B) relationship fit using the recombination-drift model (Sved). Population structure was estimated using the k10 correction.

From the original 2155 markers, we retained *r^2^* estimates from 51,850 marker pairs with an average or minimum map distance less than 30 cM. Plotting these *r^2^* estimates against map distance (Figure 5, Figure S13, and Table 2) showed that LD decays such that, at 0.1, conventional *r^2^* is equal to an average distance of 21.5 cM (Hill-Weir model) or 13.6 cM (Sved model), while *r_v_^2^* is equal to 2.8 cM (Hill-Weir model) or 2.5 cM (Sved model). The fact that *r_v_* (corrected for relatedness) is much smaller than *r^2^* (uncorrected) illustrates the necessity of removing the effect of coancestry to reduce the inflation of *r^2^* estimates, and probably reflects that the IOI panel contained groups of related lines originating from the same breeding programs. Estimates of *r_s_^2^* (accounting for population structure) did not reduce the bias in *r^2^* as substantially as did the models accounting for coancestry, which is consistent with earlier observations that this population is not highly structured. These results suggest that good genome coverage for GWAS will require a marker spacing of approximately 2.0 cM (*r_sv_^2^* k0, min *rf*, Sved model) to 2.8 cM (*r_v_^2^*, average *rf*, Hill-Weir model, Table 2). Non-linear model fitting enabled us to estimate the effective population size required for GWAS, which varied from 68 to 110 lines, depending on the choice of *r^2^* estimates and evolution models (Table 2).

**Table 2:** LD decay estimated in IOI.

	Hill-Weir					Sved				
	Average *rf*			Min *rf*		Average *rf*			Min *rf*	
*r* ^2^	Map distance (cM)	*N*_e_ ± SE		Map distance (cM)	*N*_e_ ± SE	Map distance (cM)	*N*_e_ ± SE		Map distance (cM)	*N*_e_ ± SE
*r* ^2^	21.5	9±0.1		19.5	10±0.1	13.6	17±0.1		12.3	18±0.1
*r_s_* ^2^ k0	14.2	14±0.1		12.7	15±0.2	9.9	23±0.2		8.9	25±0.2
*r_s_* ^2^ k10	14.4	13±0.1		12.9	15±0.2	10	22±0.2		9	25±0.2
*r_s_* ^2^ k50	14.5	13±0.1		13	15±0.2	10.1	22±0.2		9.1	25±0.2
*r_sv_* ^2^ k0	2.6	75±0.8		2.2	87±1	2.3	99±1.5		2	111±2
*r_sv_* ^2^ k10	2.6	73±0.8		2.3	85±1	2.3	97±1.5		2.1	109±1.9
*r_sv_* ^2^ k50	2.6	73±0.8		2.3	84±1	2.3	96±1.5		2.1	108±1.9
*r_v_* ^2^	2.8	68±0.7		2.5	79±0.9	2.5	91±1.3		2.2	102±1.7

The relationship between LD and map distance was modeled by fitting two alternate non-linear regression models: a drift-recombination equilibrium model [36] or a modified recombination-drift model including low level of mutation and an adjustment for sample size [37]. Map distance at *r^2^* = 0.1 was shown. Both average distance across six bi-parental mapping population and minimum distance from available mapping populations were used. *N_e_*, effective population size; SE, standard error.

### Germplasm diagnostics using GBS

We wished to examine how GBS technology could be used to solve two common diagnostic problems that arise occasionally in any plant breeding program. The first example involved a suspected error in the planting of one replication in an oat variety registration test. The questionable replication could have been discarded, but it had been grown and harvested at a cost that was more than double that required for genotyping the unknown samples, and discarding the replication would jeopardize the statistical power of the experiment. Problems of this nature can have great economic impact if they delay or improperly influence the release of improved plant varieties. We filtered a set of 1518 diagnostic GBS loci that were polymorphic among known and unknown lines from this test. We then applied UPGMA cluster analysis to the simple allele dissimilarity (*d*) among samples. The results (Figure S14) illustrate that each unknown sample could be paired with one or more known samples. It was then clear that the source of error was a simple reversal of seed envelopes in one of the planting trays. The phenotypic data could then be reassigned to complete the analysis. Although the unknown experimental units could be unambiguously corrected based on the genetic data, a few of the distances between samples known to originate from the same variety (*e.g.*, samples 128 and 232 in Figure S14) were larger than expected. We expect that this is because the DNA samples were prepared from a few seeds sampled randomly from bulks that were harvested by combine from the registration test. If so, this draws attention to the fact that seed harvested from yield trials is often impure and should be used with caution in genetic studies.

The second diagnostic problem was to determine whether an F_2_ population originated from a true cross or from selfed seed of a parent. In this case, the progeny appeared very homogeneous and an error was suspected. However, resources had been invested in the cross, and it seemed worthwhile to address this issue before discarding F_3_ seed. We filtered GBS loci for ten F_2_ progeny and the intended male parent of the cross, together with 340 additional progeny from the IOI diversity panel. The intended female parent was unavailable, but a maternal grandparent (Leggett) was available from the IOI set. Filtering at 10% heterozygosity, 10% MAF, and 90% completeness gave 2205 locus calls. All 2205 loci were completely homogeneous among the suspected progeny and the male parent, with the exception of minor variants that appeared random and fell within a 1% tolerance for scoring error. A cluster analysis (Figure S15) supported this result. Thus, it was concluded that these seeds were not from a true cross, and that they probably represented a harvesting error in the crossing block.

## Discussion

### Factors that influence the quality and quantity of GBS SNP calls

The completeness of GBS SNP calls and the number of SNPs filtered at a given completeness are influenced by several factors, including: (1) the actual number of genomic fragments produced by the complexity reduction, (2) the sequencing depth, as determined by the number of reads and the number of samples that are multiplexed, and (3) the underlying density of SNPs, which is related to the diversity and structure of the population [41]. Although other restriction enzymes can be used for complexity reduction (*e.g.*, [11], [17]), we limited our present investigation in oat to the *Pst*I*-Msp*I combination which had previously been optimized for the similar-sized genome of wheat [12], [23]. This method provided suitable results in oat with minor modifications, and identified tens of thousands of sequence polymorphisms. Although the results in Figure 1 show a somewhat linear response in the number of SNPs identified as sequencing depth increases, the number of SNP calls for all levels of completeness would eventually plateau at a limit (possibly more than 100,000) determined by the complexity reduction and the population. The depth of sequencing required to obtain complete data for all SNP-containing fragments is currently not practical nor cost effective, nor is it required to obtain meaningful genetic data and results. However, we strongly recommend that additional replicate samples be used for the parents of mapping populations or other material that is critical to an experiment to achieve greater sequencing depth for these lines. In addition, there may be opportunities to produce and analyze reduced complexities generated using selective bases added to primers of existing enzyme systems [18]. Use of selective bases may have value for certain investigations where a more complete and consistent genotyping is required, and would have the advantage of providing data at a subset of loci already characterized in the present work. An example of an application where this might be useful is the routine diagnosis of variety identity or other diagnostic applications that do not rely on a high density of genetic markers. One can also “complete” the missing data points through data imputation. Various imputation methods have been developed which can be highly effective for subsequent analyses (reviewed in [42]). Although most methods require known marker order, often provided by a reference genome sequence, imputation can also be applied to unordered markers and could increase the accuracy of further analyses.

As the number of diverse lines was increased, the number of GBS loci also increased, but this number plateaued at a relatively small number of lines. This result is consistent with the notion that each additional line added an increasing likelihood of identifying rare alleles, such that an increasing number of loci will meet the allele frequency threshold. Meanwhile, larger samples may make it increasingly unlikely that loci will meet filtering criteria and/or that spurious loci will be included. This is possibly why the plateau occurs earlier as data are filtered for higher completeness, and provides a possible explanation for the decrease in SNP number beyond the plateau (Figure 2). In addition, completeness is stochastically variable among samples and among loci. Thus, loci that are included in two diversity subsets are not necessarily those that are the most complete across the entire panel. This phenomenon may make it difficult to obtain consistent loci among different experiments if thresholds for completeness are set too high. For this reason, we recommend that filtering be performed at multiple levels, depending on the purpose of the experiment. For example, the VxL population was filtered at high stringency to develop an initial *de-novo* map, then at low stringency to identify additional GBS loci that mapped across populations.

One of the factors of concern in oat and other polyploid species is that the analysis of genetic loci may be confounded by duplicated homoeologous regions. This factor has made it difficult to discover and apply gene-based SNP loci, and has resulted in some assays where SNPs must be scored with dominant alleles [4], [43]. Based on the relative scarcity of BLAST matches in related species, the majority of the GBS loci from the *Pst*I*-Msp*I complexity reduction appear to be located in non-genic regions. Furthermore, because the GBS marker calling is based on counts of specific allele variants, all GBS loci are scored with co-dominant alleles. For these reasons, and also because of intense filtering to remove loci with non-diploid inheritance, the GBS method provides good representation of single genetic loci. This would also tend to remove SNPs that fall in conserved genic regions, as these GBS tags would align to all three genomes and the resulting SNPs would not segregate as single loci. Although heterozygote calls are more subject to genotyping errors, our results show the interest of including heterozygous genotypes in certain applications. In particular, we observed that the overall quality of heterozygote determination in the VxL F_4_ population was good, and that it enabled meaningful characterization of heterozygous regions in the graphical genotypes (Table S2). However, we have not thoroughly investigated the use of GBS markers in F_2_ populations, where it is possible to confuse single loci with duplicated loci having similar genetic ratios. For this reason, GBS should be used with caution in F_2_ mapping populations, unless a reference genome or an ordered scaffold is available such that heterozygous regions can be imputed.

### Annotation of oat GBS SNPs

We estimated that 5% of oat GBS SNPs were within protein coding sequences. This estimate should be considered preliminary because of the current lack of public oat gene sequences. Using an automated SNP annotation pipeline, Kono *et al.* [44] estimated that only 1.3% of a preliminary subsample of 5000 oat GBS SNPs were genic SNPs. Most of their matches were also based on *Brachypodium*. Their estimate may be lower because of sampling bias and because they used a higher stringency in protein matching (e<5×10^−5^). Using the same annotation pipeline, Kono *et al*. [44] identified 10.6% of a sample of barley GBS tags as being genic SNPs. The rate in barley may be substantially higher because of the greater availability of barley gene sequences.

### Methods of bioinformatics analysis and workflow

We used two bioinformatics pipelines to perform SNP calling. The motivation for using two pipelines was that they were the only two non-reference-genome GBS pipelines of which we were aware. While the UNEAK pipeline gave clear, predictable results, the number of loci passing secondary filtering was low compared to those called by the population-level pipeline. In addition, as observed elsewhere [22], the GBS SNPs identified can vary considerably with different methods. Across all SNPs placed on the oat consensus map, 43% (19,209 out of 45,117) were called only by the population-based method, 22% (9997 out of 45,117) were called only by the UNEAK method, and 36% were called by both methods (Figure 3). Although the population filtering method called more SNPs, these SNPs contained a higher redundancy, and multiple SNPs (linked or unlinked) were sometimes assigned to the same context sequence in the report (data not shown). This is a result of calling SNPs in tags that belong to complex gene families and/or in context sequences of haplotypes that contain multiple SNPs. Such loci are usually ignored by the UNEAK pipeline, which reports only tags with single SNPs. However, in some cases, the direct use of SNPs from the population-filtering report would prevent the correct development of secondary allele assays, if no detailed validation of the assembly was performed to generate unambiguous diagnostic probes for correct SNP interrogation. For these reasons, we performed some of this work using only the UNEAK pipeline. Nevertheless, it was apparent that a much greater number of SNPs were called by the population-filtering method, and these additional SNPs may provide important information which would otherwise be discarded. Until bioinformatics methods can be further refined, we recommend using a combined data set composed of SNPs called by both pipelines. We also recommend the preferential use of SNPs called by the UNEAK pipeline in the development of secondary assays, and the use of appropriate statistical methods to reduce the influence of marker redundancy in subsequent applications such as association mapping.

### Saturation of a consensus linkage map with GBS loci

High density maps are required for the precise mapping of important agronomic traits to be targeted in marker-assisted breeding. The high-throughput GBS technology enabled the placement of 45,117 GBS loci identified across six bi-parental mapping populations on the oat consensus map [4]. This high-density map showed marker clustering along chromosomes, similar to a barley map saturated with GBS markers [12]. Since many of these clusters represent centromeres, GBS loci are likely to be more evenly distributed along the physical map. However, gaps of up to 15 cM were still observed in the high-density map. Some gaps may result from lack of polymorphism in the mapping populations, which can be further improved by integrating other mapping populations. Gaps could also be filled in by using GBS libraries produced using different restriction enzymes, as shown in wheat [17]. Gaps and multiple clusters may also be related to the construction of the initial consensus map and it is possible that the consensus map will be improved once GBS markers are fully integrated with additional gene-based SNP loci.

### Utility of GBS markers for genetic analysis in oat

We have investigated different GBS applications relevant to oat breeding. In addition to saturating an existing consensus map, GBS markers were suitable for building a *de-novo* linkage map with good genome coverage that revealed colinearity with the consensus. These results were successful because GBS provided a large number of markers that were polymorphic in multiple populations. This will facilitate comparative mapping to validate and refine the location of target alleles in diverse genetic backgrounds, and will increase the options available for molecular breeding.

Analyses of diverse germplasm showed weak population structure in our sample. This weak structure was observed previously when DArT markers were used across a larger oat diversity panel that included the IOI set used in this study [31]. While rice, barley, and maize are known for having strong population structure [45]–[47], oat, despite having four recognizable types (naked, covered, spring, and winter), has not shown obvious population structure within the samples analysed to date. Although the majority of lines in the IOI set are the spring type (318 out of 340) and covered-seeded (312 out of 340), the remaining naked or winter lines did not form distinct sub-clusters. Instead, the scatterplot tended to reflect the geographic locations of breeding programs and (by inference) the degree of coancestry among lines. A possible explanation for these results could be that, while oat breeders tend to make most crosses among parents that are locally adapted, they have also exchanged elite germplasm with some regularity. The relatively small effective population size compared to the number of IOI lines also supports this interpretation.

The use of GWAS is widely considered to be an attractive alternative to the use of structured (*e.g.*, bi-parental) populations for identifying adaptive alleles for use in molecular breeding. However, effective GWAS requires prior knowledge of LD decay and an awareness of the population under investigation. Our results highlight that there is a strong gradient of coancestry that needs to be accounted for through the use of an appropriate model, but that other sources of population structure are not important in the population investigated. Although GBS appears to provide a much higher density of markers than required for GWAS, it is possible that target loci are within gaps that do not contain a suitable marker density. For this reason, it may be useful to test additional enzyme combinations for GBS for use in a large association panel when a large investment has been made in phenotyping.

## Conclusion

The choice of marker technologies is critical to the success and future application of genetic and genomic research. GBS is attractive because it provides thorough genome coverage and can be applied at low cost with or without a reference genome sequence. However, GBS requires intense bioinformatic analysis, an awareness of the need to filter data, and a tolerance for incomplete data. In this work, we have shown that GBS is an effective method to discover and apply SNPs in the large and complex oat genome, and that GBS integrates and compares favourably with an established SNP technology. The resulting data have provided whole-genome coverage at a density that enables detailed analysis of genetic diversity and high power to detect specific genetic variants.

Our overall conclusion and recommendation is that GBS be used as a cost-effective primary tool in any application similar to those that we have explored in oat. Other applications of GBS in oat, including QTL analysis, genomic selection, and the ordering of genome sequence scaffolds, remain to be fully tested, but are expected to be successful based on indications from this work and from similar use in other species. In future work, we intend to apply GBS routinely to genotype and select among advanced oat breeding lines. As a side benefit to improved selection, we hope to provide new information about the sources and locations of alleles for better adaptation in oat, and to integrate this information with the existing genomic knowledge for oat.

## Supporting Information

Figure S1 **HTML map format.** Instructions for using the HTML-formatted map. The map can be found locally in Text S2 as “HTML_Local_text_S2.html” or online at: http://ahoy.aowc.ca/html_link_gbs_text_s2.html.
(PDF)

Figure S2 **Distribution of map gaps in the original and updated oat consensus maps.** Empty bars show the distribution of map gaps in the first oat consensus map (Oliver *et al.*, 2013); solid bars show the distribution of map gaps in the consensus map with the GBS markers placed on it (this study). Only gaps larger than 5 cM are shown.
(TIF)

Figure S3 **Orthology between oat and** ***Brachypodium distachyon*** **.** Each dot represents the position of a sequence match (BLASTn, *E*<10^-12^) between the oat consensus map (blue dots for GBS loci, red dots for array-based SNPs) and the assembled *Brachypodium distachyon* (Bd) pseudomolecule (release 2.1; http://www.brachypodium.org).
(TIF)

Figure S4 **Orthology between oat and rice.** Each dot represents the position of a sequence match (BLASTn, *E*<10^−12^) between the oat consensus map (blue dots for GBS loci, red dots for array-based SNPs) and the genome sequence of rice (*Oryza sativa* L., release 6.1 from http://rice.plantbiology.msu.edu).
(TIF)

Figure S5 **Orthology between oat and barley.** Each dot represents the position of a sequence match (BLASTn, *E*<10^−12^) between the oat consensus map (blue dots for GBS loci, red dots for array-based SNPs) and barley (*Hordeum vulgare* L., *cv.* Morex, release 2.0 from ftp://ftp.ensemblgenomes.org/pub/plants/release-20/fasta/hordeum_vulgare/dna/ non repeat-masked versions). Barley pseudomolecules are assembled according to chromosome arm (long (2HL to 7HL) or short (2HS to 7HS)), except for chromosome 1H.
(TIF)

Figure S6 **Orthology between oat and barley (multiple matches removed).** Each dot represents the position of a sequence match (BLASTn, *E*<10^−12^) between the oat consensus map (blue dots for GBS loci, red dots for array-based SNPs) and barley (*Hordeum vulgare* L., *cv*. Morex, release 2.0 from ftp://ftp.ensemblgenomes.org/pub/plants/release-20/fasta/hordeum_vulgare/dna/ non repeat-masked versions). A subset of matches from Figure S5 is shown: oat sequences that matched other *Hv* sequences more than 6 times at the same BLASTn expectation have been removed.
(TIF)

Figure S7 **Comparison between the VxL map and the consensus map.** Each dot represents a marker shared by the two maps. Red dots highlight markers of higher heterozygosity (between 8 and 13%).
(TIF)

Figure S8 **Distribution of distances between adjacent markers used for LD analysis.** Markers were first sorted according to map position, then the distances were calculated as Position_ m_ minus Position_ m-1_. For the first marker of each chromosome, the interval was calculated as the difference between its position and the position of the second marker.
(TIFF)

Figure S9 **Scree plots of principal components of IOI genotypic data.** The first 20 components at three levels of LD correction were used to draw the plots: k0 (without correction), k10 (using 10 adjacent markers for LD adjustment), and k50 (using 50 adjacent markers for LD adjustment). No obvious “elbows” were observed but there was a two-stage decay: at PC5 and at PC10.
(TIF)

Figure S10 **IOI population structure scatter plot (PC1** ***vs.*** **PC2) based on genetic clustering.** Three levels of LD correction are shown: k0 (A), k10 (B), and k50 (C and D).
(TIF)

Figure S11 **IOI population structure scatter plot (PC1** ***vs.*** **PC2) coloured based on the geographical origins of the lines.** Three levels of LD correction are shown: k0 (A), k10 (B), and k50 (C).
(TIFF)

Figure S12 **IOI population structure scatter plot (PC3** ***vs.*** **PC4) coloured based on genetic clustering (left) or plant habitat (right).** Three levels of LD correction are shown: k0 (up), k10 (middle), and k50 (bottom).
(TIFF)

Figure S13 **LD decay plot.** *r^2^* estimates were plotted against both minimum and average map distance (recombination frequency expressed in cM): (A) relationship fit using the mutation model (Hill-Weir), (B) relationship fit using the recombination-drift model (Sved).
(TIF)

Figure S14 **Using GBS markers to resolve an issue in a field experiment.** UPGMA cluster analysis of simple allele-matching metric (d) based on 1518 GBS loci with heterozygosity <8%, MAF >20%, and completeness >95%. This evidence was used to correct a planting error in a field experiment. The samples in one replication (red samples) were out of order compared to those in a second, correct replication (green samples), and a set of known controls (blue samples). Analyzing the sub-clusters in the above dendrogram and assigning corrected identities (entry numbers 1–32, above) to the samples in replication 1 made it obvious that the planting order of the first replication had been reversed.
(TIF)

Figure S15 **Using GBS markers to resolve an issue with breeding materials.** UPGMA cluster analysis of simple allele-matching metric (*d*) based on 2205 GBS loci with >90% completeness. GBS calls were made across samples from 343 diverse oat varieties plus ten putative F_2_ segregants (green) from a putative cross between SA060123 (red) and a progeny of Leggett (blue). Eight closely related oat cultivars are also shown in this partial cluster dendrogram. Of the 2205 loci, only 131 (6%) showed any variation among the ten progeny plus SA060123, and this variation was within the expectations of heterozygous miscalls. This evidence was used to conclude that the ten progeny were actually from selfed seed of SA060123 rather than true segregants from a hybrid.
(TIF)

Figure S16
**Effect of adding populations on the number of markers placed on the oat consensus map.** Using the consensus map [4] as a framework and starting with a different population each time, markers from the six populations were placed sequentially in all possible combinations (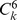, k = 1 to 6). The number of additional markers contributed by the final map at each step is represented by different colours and shapes. The box represents the range between the first and third quartiles and the thick horizontal bar represents the median.(TIFF)

Figure S17 **Distribution of annotated GBS markers across the oat consensus map.** Maps of each chromosome were divided into 5 cM bins and the number of intergenic/genic markers counted for each bin. Some markers are in the negative range because they are placed off the beginning of the linkage group.
(PDF)

Table S1 **GBS mapping data for the six bi-parental populations used to update the oat consensus map (Oliver** ***et al*** **., 2013).**
(ZIP)

Table S2 **Graphical genotypes of markers comprising the VxL map.**
(XLSX)

Table S3 **Information about the lines comprising the IOI panel.**
(XLSX)

Table S4 **Raw reads statistics and key file for GBS pipeline.**
(XLSX)

Text S1 **Custom Pascal code for ‘CbyT’.**
(TXT)

Text S2 **Updated oat consensus map (HTML_Local_text_S2.html).** See Figure S1 for instructions.
(HTML)
