# Supplementary material for: Using Genotyping-By-Sequencing (GBS) for Genomic Discovery in Cultivated Oat
Source: PLoS One. 2014 Jul 21;9(7):e102448. doi: 10.1371/journal.pone.0102448 (PMC4105502; doi:10.1371/journal.pone.0102448)
Supplement: Figure S1 — HTML map format. Instructions for using the HTML-formatted map. The map can be found locally in Text S2 as “HTML_Local_text_S2.html” or online at: http://ahoy.aowc.ca/html_link_gbs_text_s2.html. (PDF) [file pone.0102448.s001.pdf]

Open “HTML_local_text_S2.html” in your favorite browser

Ctrl-F to search, and find your favorite marker

Chromosome 5C

Chr	Pos	Framework	Placed SNP	Placed GBS
5C	-16			avgbs_52659 avgbs_21618
5C	-15			avgbs_102165 avgbs_32759 avgbs_15178
5C	-14			avgbs_71135
5C	-13			avgbs_213052
5C	-12			avgbs_7199 avgbs_63268 avgbs_73244 avgbs_73245 avgbs_224566
5C	-11			avgbs_87990 avgbs_235486
5C	-10			avgbs_110756 avgbs_6K_57836 avgbs_9090 avgbs_74977 avgbs_105828
5C	-9			[illegible] avgbs_76025
5C	-8		GMI_ES02_c5703_238	[illegible] avgbs_49703
5C	-7			[illegible] avgbs_109203 avgbs_223349 avgbs_34978
5C	-5			avgbs_3[illegible]94 avgbs_81402 avgbs_14041 avgbs_14042 avgbs_225390 avgbs_38195 avgbs_[illegible]59
5C	-4			[illegible]
5C	-3			[illegible]
5C	-2			[illegible]
5C	-1			[illegible]
5C	0	GMI_ES17_c5197_503	[illegible]	[illegible]41

Click!

Marker passport: (opens in a new window)

- Target marker is highlighted in green
- Its approximated position on the framework is shown (CHR-POS)
- Recombination Fractions (RF) are actual informative counts
- RF are shown across all progeny and within populations
- Shows all markers within 20% RF of target marker in ***any*** population
  Even if they have been placed on a different consensus chromosome!

FW	MARKER	CHR	POS	RF%-All	RF-All	RF%-Min	RF-Min	OP	PG	OT	KO	CH	HZ
	avgbs_71561	1C	50.6	38.5	30/78	14.3	4/28	26/50				4/28	
	avgbs_15178	5C	-14.8	0.0	0/26	0.0	0/26					0/26	
	avgbs_213052	5C	-13.2	1.6	2/95	1.6	2/95		2/95				
	avgbs_63268	5C	-12.2	1.4	1/74	1.4	1/74		1/74				
	avgbs_224566	5C	-11.8	3.0	1/33	3.0	1/33					1/33	
	avgbs_7199	5C	-11.6	1.8	2/82	1.8	2/82		2/82				
	avgbs_87990	5C	-11.5	5.7	4/61	5.7	4/61		4/61				
	avgbs_110756	5C	-10.3	5.2	6/116	5.1	4/78	4/78				2/38	
	avgbs_6K_578[illegible]	[illegible]	[illegible]	[illegible]	[illegible]	[illegible]	[illegible]		[illegible]				
	avgbs_74977	[illegible]	[illegible]	[illegible]	[illegible]	[illegible]	[illegible]	[illegible]	[illegible]	[illegible]	[illegible]	[illegible]	[illegible]
	avgbs_9090	[illegible]	[illegible]	[illegible]	[illegible]	[illegible]	[illegible]	[illegible]	[illegible]	[illegible]	[illegible]	[illegible]	[illegible]
	avgbs_105828	[illegible]	[illegible]	[illegible]	[illegible]	[illegible]	[illegible]	[illegible]	[illegible]	[illegible]	[illegible]	[illegible]	[illegible]
	avgbs_76024	[illegible]	[illegible]	[illegible]	[illegible]	[illegible]	[illegible]	[illegible]	[illegible]	[illegible]	[illegible]	[illegible]	[illegible]
	avgbs_76025	[illegible]	[illegible]	[illegible]	[illegible]	[illegible]	[illegible]	[illegible]	[illegible]	[illegible]	[illegible]	[illegible]	[illegible]
	avgbs_49703	[illegible]	[illegible]	11.1	[illegible]	11.1	[illegible]					[illegible]	
	GMI_ES02_c5703_238	5C	-7.7										

Is this a framework marker on the consensus map of Oliver et al., 2013 ?
→ Y for “Yes”, blank for “No”.

For example:
GMI_ES02_c5703_238 is linked to avgbs_110756
with **6/116** recombinants across all populations and **4/78** recombinants in the OP population (min RF).
GMI_ES02_c5703_238 is also linked to avgbs_71561 in CH population but is placed to chromosome 1C across all available populations.
