# Supplementary figures and images for: Using Genotyping-By-Sequencing (GBS) for Genomic Discovery in Cultivated Oat
Source: PLoS One. 2014 Jul 21;9(7):e102448. doi: 10.1371/journal.pone.0102448 (PMC4105502; doi:10.1371/journal.pone.0102448)

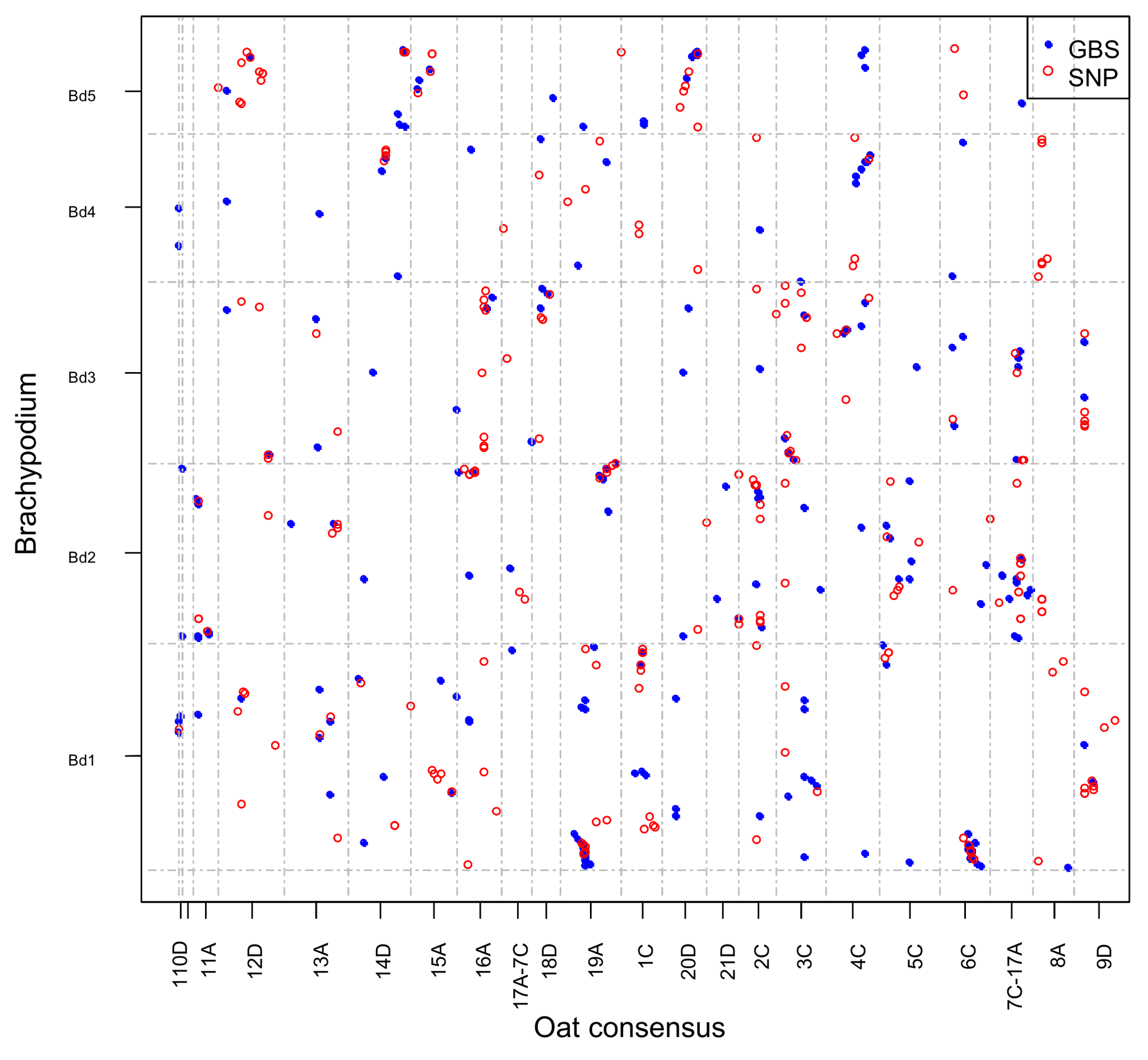

Supplement: Figure S3 — Orthology between oat and Brachypodium distachyon . Each dot represents the position of a sequence match (BLASTn, E<10-12) between the oat consensus map (blue dots for GBS loci, red dots for array-based SNPs) and the assembled Brachypodium distachyon (Bd) pseudomolecule (release 2.1; http://www.brachypodium.org). (TIF) [file pone.0102448.s003.tif]

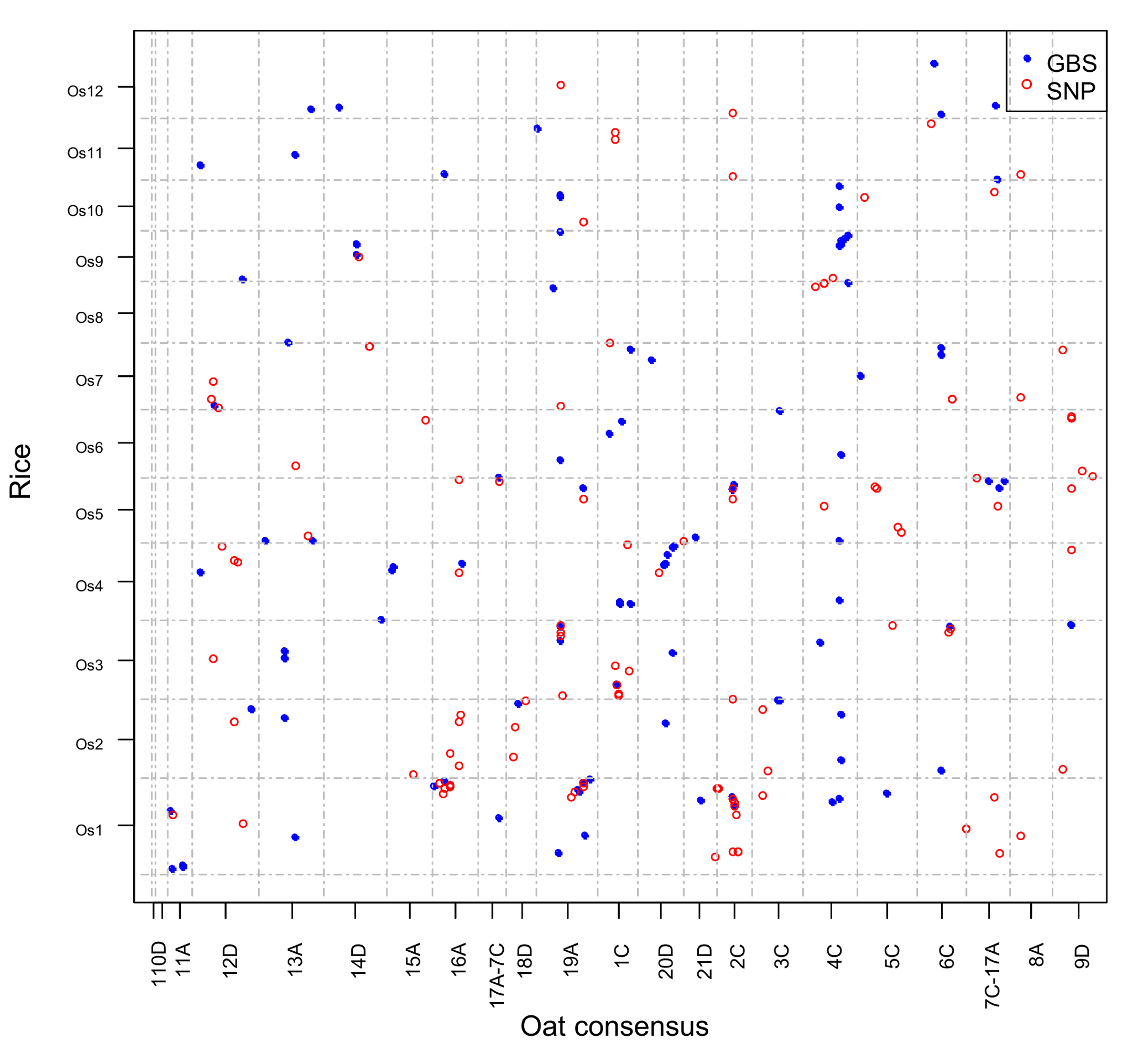

Supplement: Figure S4 — Orthology between oat and rice. Each dot represents the position of a sequence match (BLASTn, E<10−12) between the oat consensus map (blue dots for GBS loci, red dots for array-based SNPs) and the genome sequence of rice (Oryza sativa L., release 6.1 from http://rice.plantbiology.msu.edu). (TIF) [file pone.0102448.s004.tif]

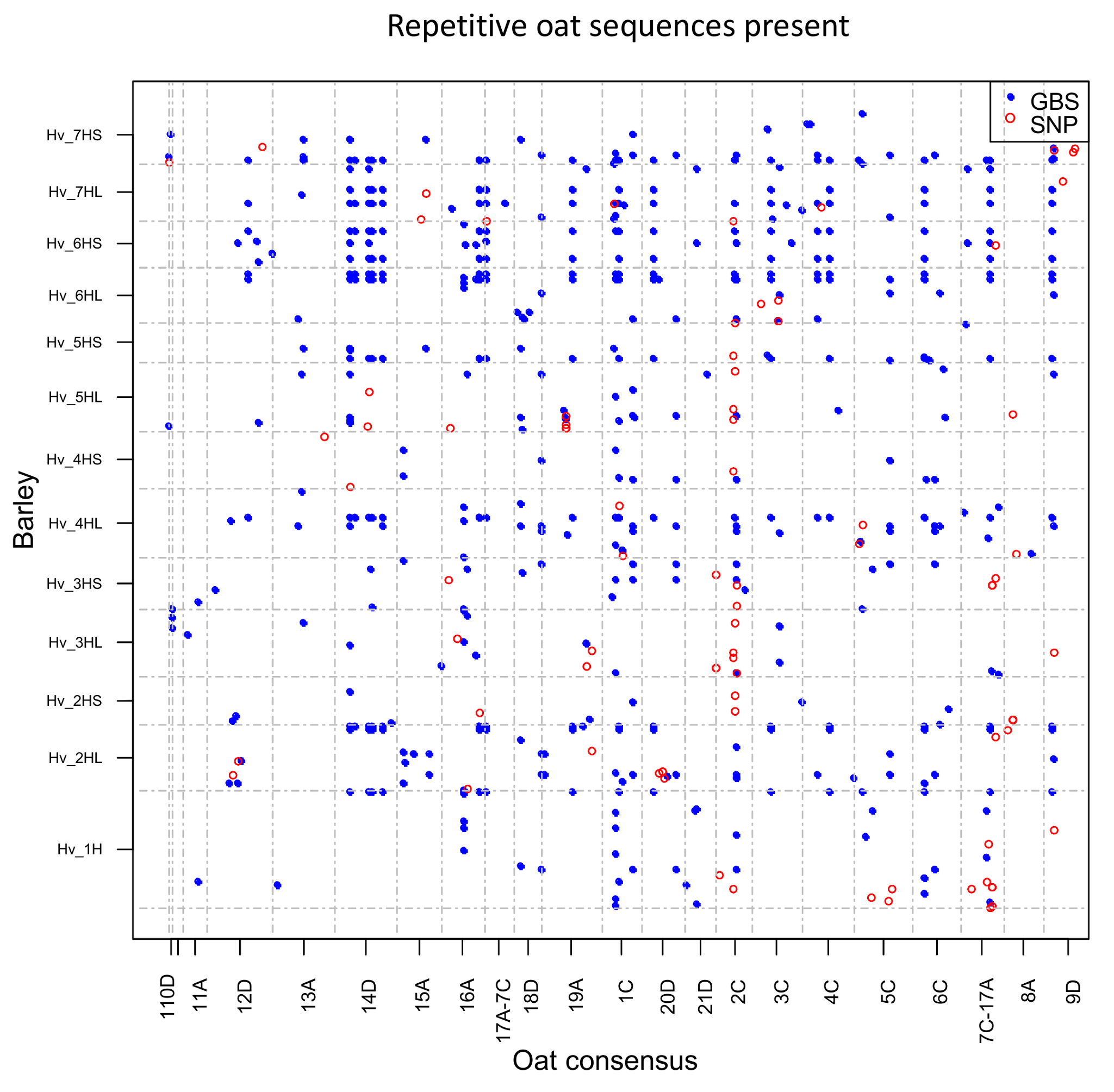

Supplement: Figure S5 — Orthology between oat and barley. Each dot represents the position of a sequence match (BLASTn, E<10−12) between the oat consensus map (blue dots for GBS loci, red dots for array-based SNPs) and barley (Hordeum vulgare L., cv. Morex, release 2.0 from ftp://ftp.ensemblgenomes.org/pub/plants/release-20/fasta/hordeum_vulgare/dna/ non repeat-masked versions). Barley pseudomolecules are assembled according to chromosome arm (long (2HL to 7HL) or short (2HS to 7HS)), except for chromosome 1H. (TIF) [file pone.0102448.s005.tif]

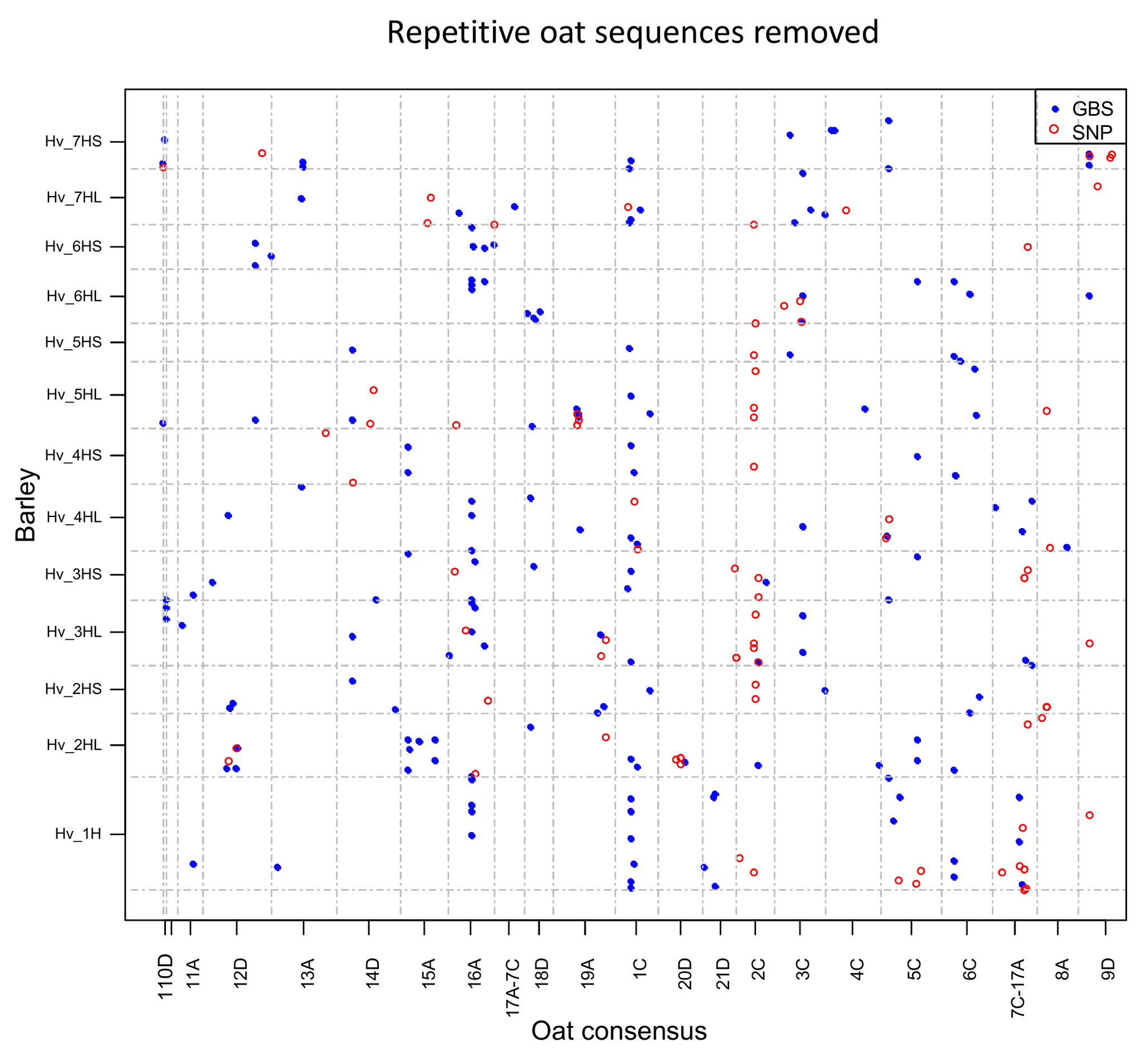

Supplement: Figure S6 — Orthology between oat and barley (multiple matches removed). Each dot represents the position of a sequence match (BLASTn, E<10−12) between the oat consensus map (blue dots for GBS loci, red dots for array-based SNPs) and barley (Hordeum vulgare L., cv. Morex, release 2.0 from ftp://ftp.ensemblgenomes.org/pub/plants/release-20/fasta/hordeum_vulgare/dna/ non repeat-masked versions). A subset of matches from Figure S5 is shown: oat sequences that matched other Hv sequences more than 6 times at the same BLASTn expectation have been removed. (TIF) [file pone.0102448.s006.tif]

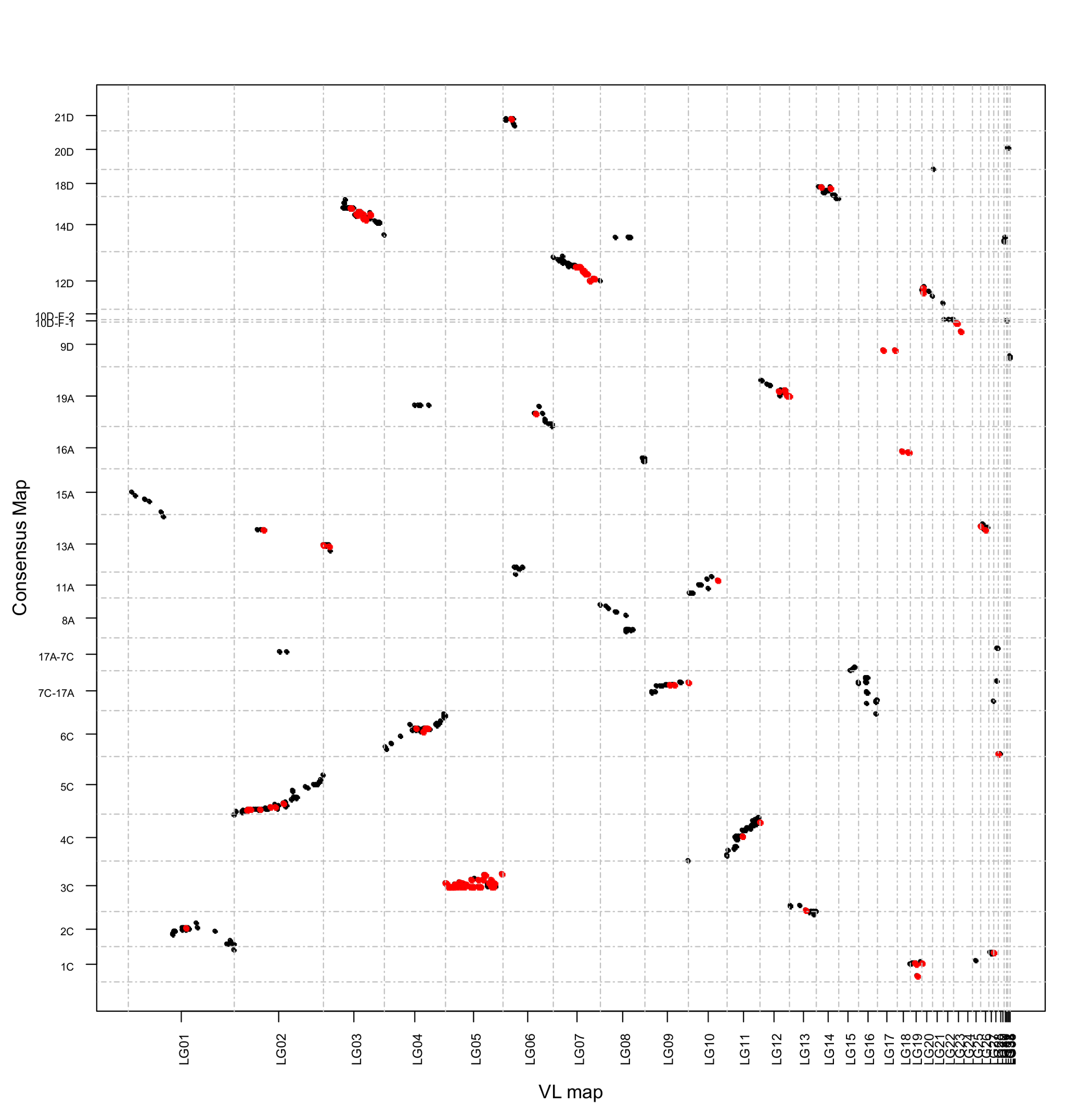

Supplement: Figure S7 — Comparison between the VxL map and the consensus map. Each dot represents a marker shared by the two maps. Red dots highlight markers of higher heterozygosity (between 8 and 13%). (TIF) [file pone.0102448.s007.tif]

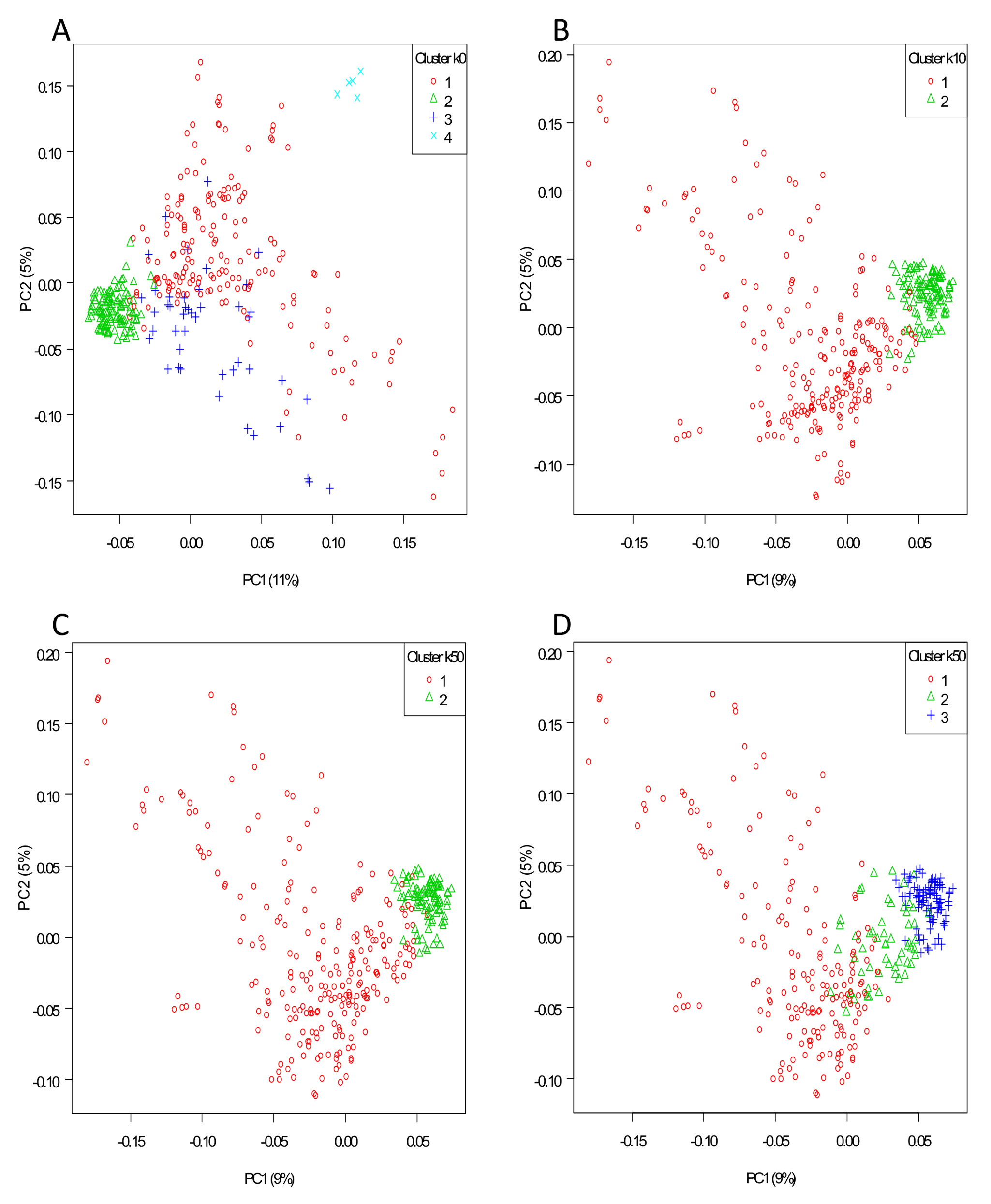

Supplement: Figure S10 — IOI population structure scatter plot (PC1 vs. PC2) based on genetic clustering. Three levels of LD correction are shown: k0 (A), k10 (B), and k50 (C and D). (TIF) [file pone.0102448.s010.tif]

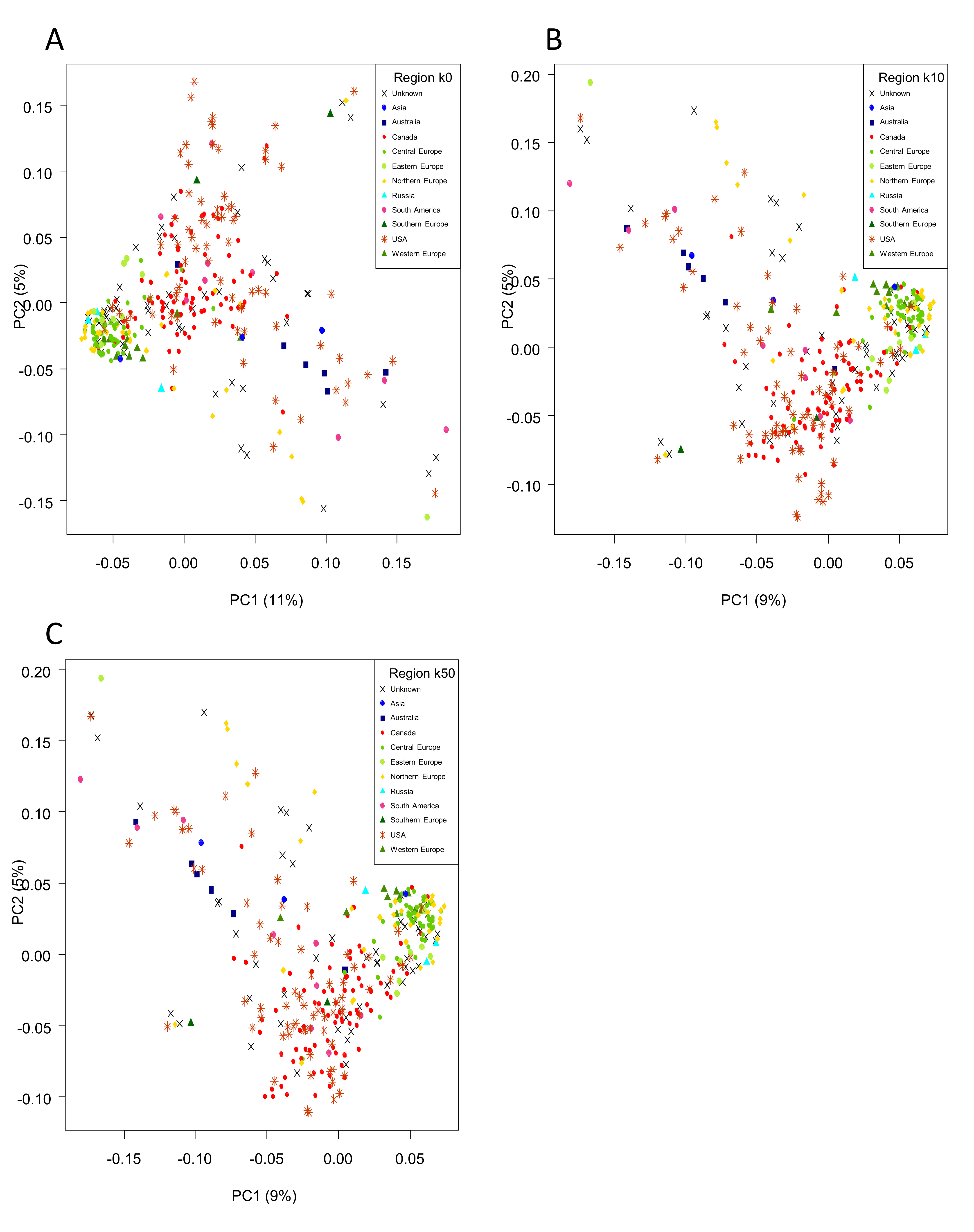

Supplement: Figure S11 — IOI population structure scatter plot (PC1 vs. PC2) coloured based on the geographical origins of the lines. Three levels of LD correction are shown: k0 (A), k10 (B), and k50 (C). (TIFF) [file pone.0102448.s011.tiff]

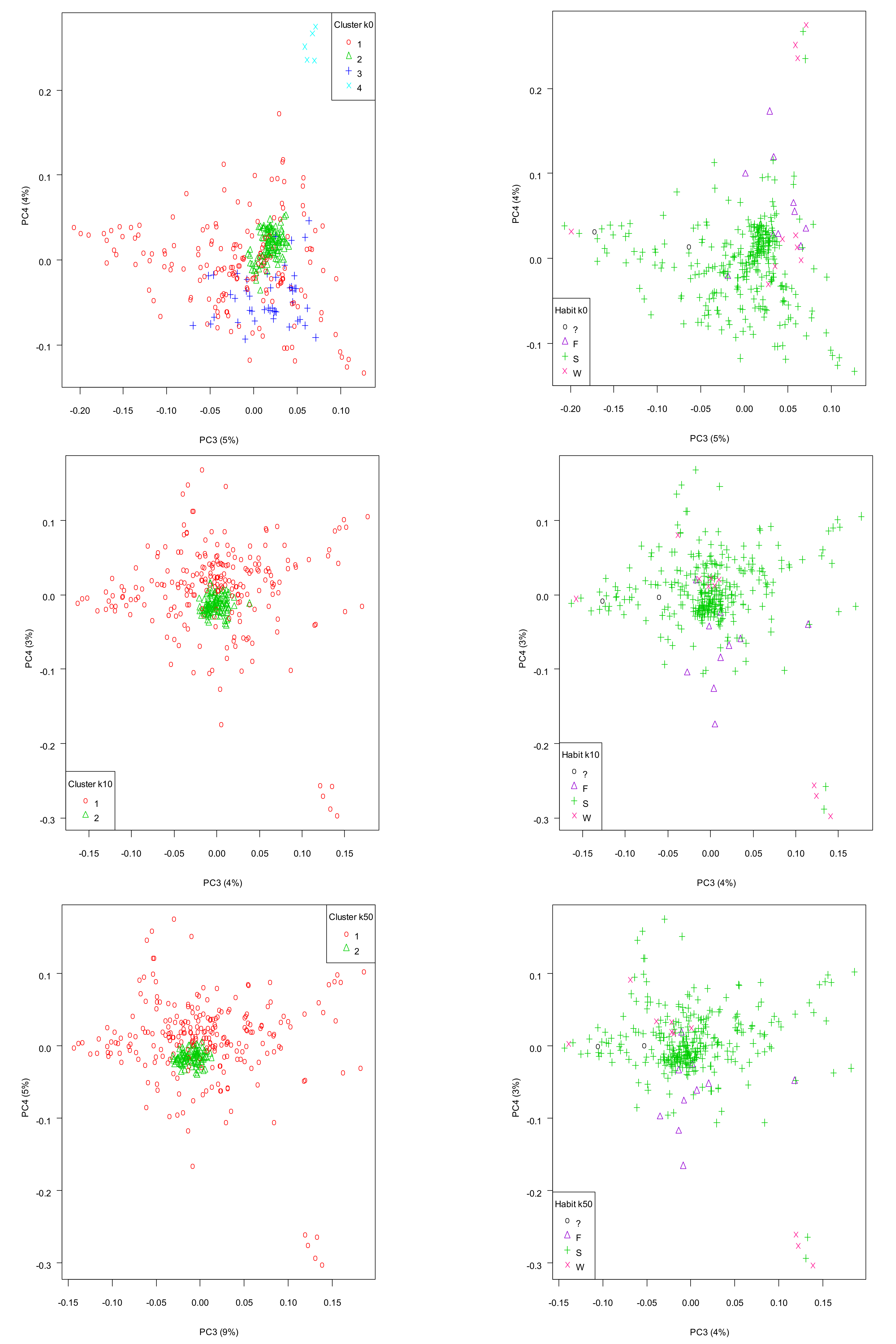

Supplement: Figure S12 — IOI population structure scatter plot (PC3 vs. PC4) coloured based on genetic clustering (left) or plant habitat (right). Three levels of LD correction are shown: k0 (up), k10 (middle), and k50 (bottom). (TIFF) [file pone.0102448.s012.tiff]

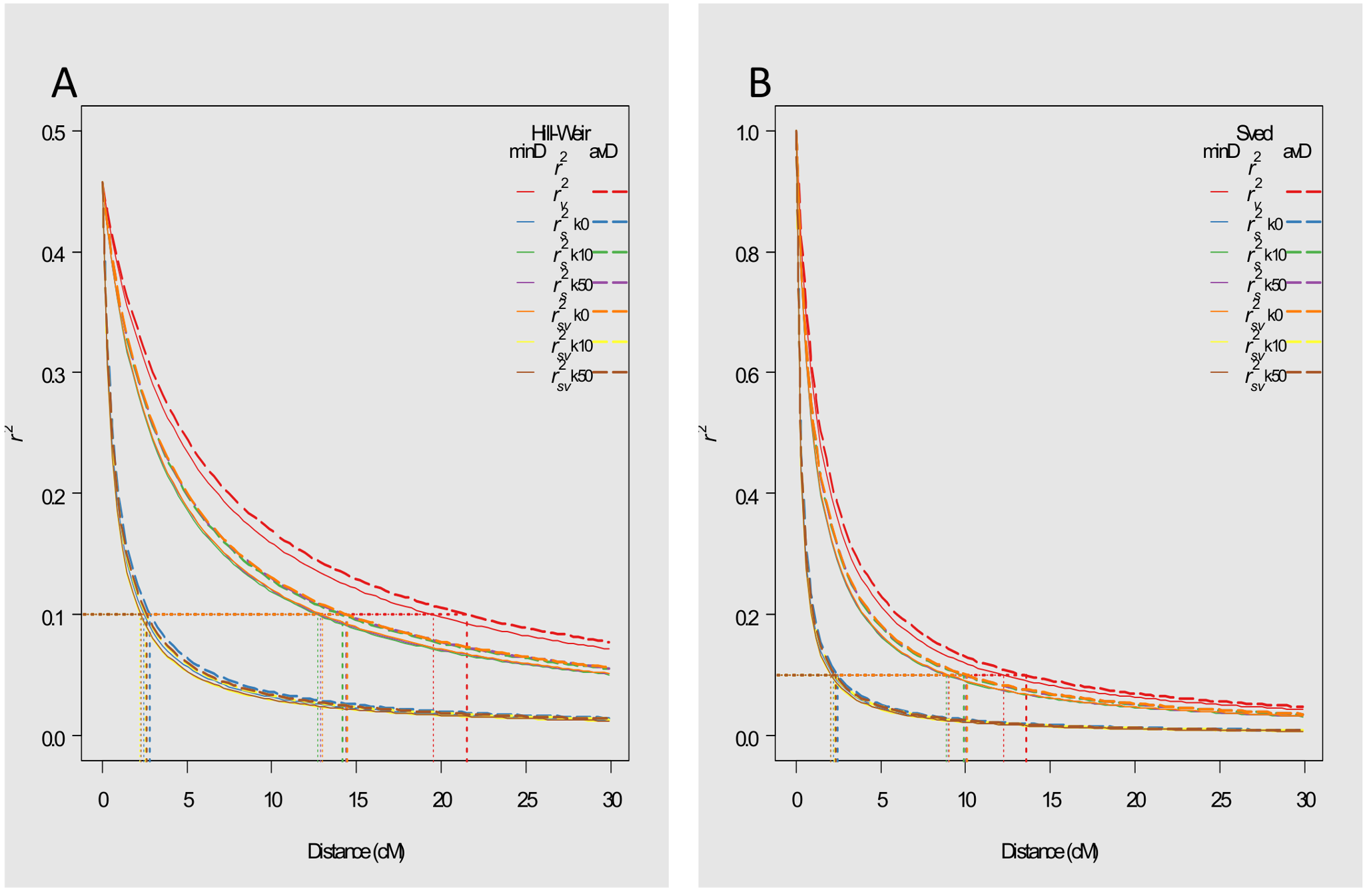

Supplement: Figure S13 — LD decay plot. r2 estimates were plotted against both minimum and average map distance (recombination frequency expressed in cM): (A) relationship fit using the mutation model (Hill-Weir), (B) relationship fit using the recombination-drift model (Sved). (TIF) [file pone.0102448.s013.tif]

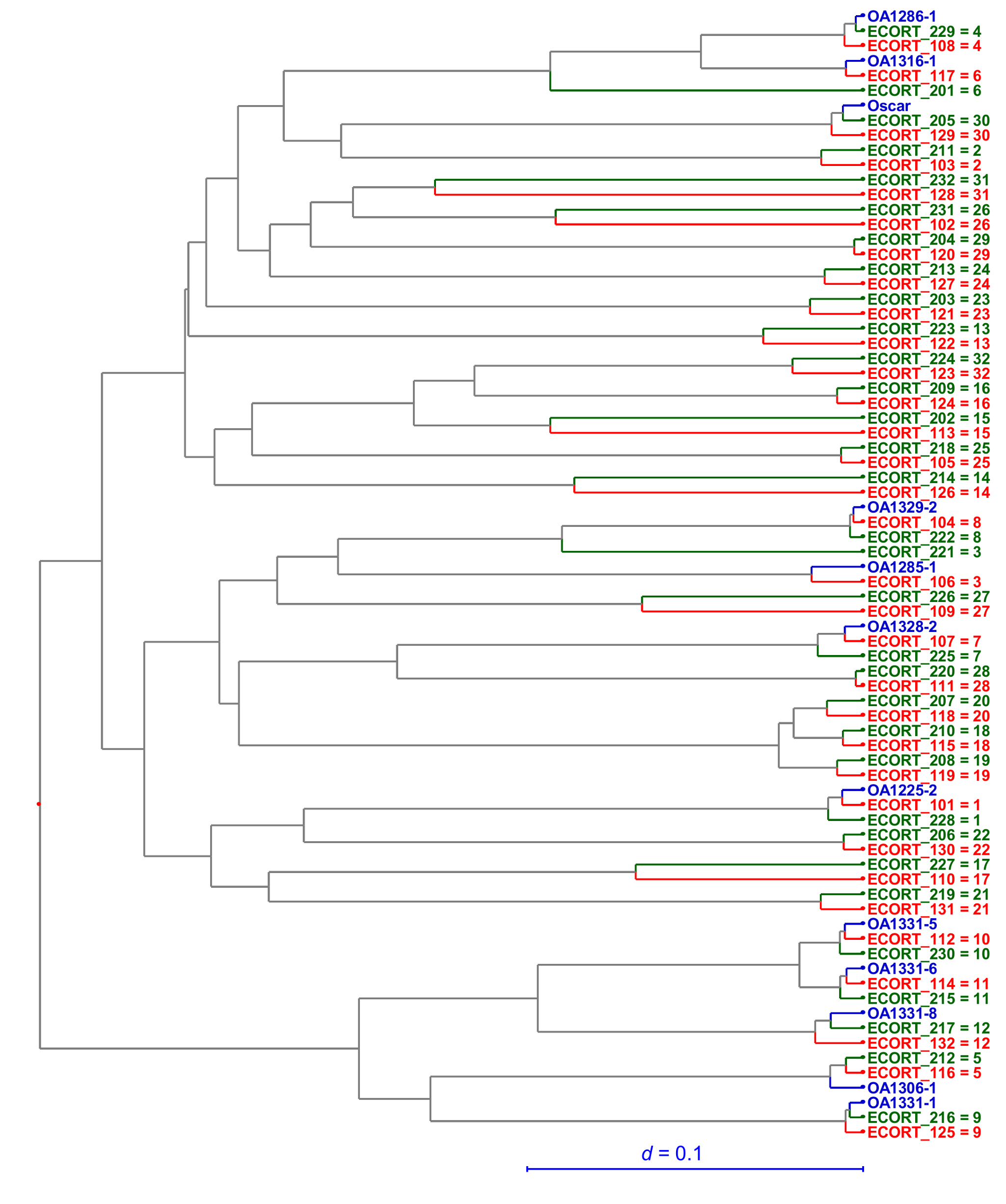

Supplement: Figure S14 — Using GBS markers to resolve an issue in a field experiment. UPGMA cluster analysis of simple allele-matching metric (d) based on 1518 GBS loci with heterozygosity <8%, MAF >20%, and completeness >95%. This evidence was used to correct a planting error in a field experiment. The samples in one replication (red samples) were out of order compared to those in a second, correct replication (green samples), and a set of known controls (blue samples). Analyzing the sub-clusters in the above dendrogram and assigning corrected identities (entry numbers 1–32, above) to the samples in replication 1 made it obvious that the planting order of the first replication had been reversed. (TIF) [file pone.0102448.s014.tif]

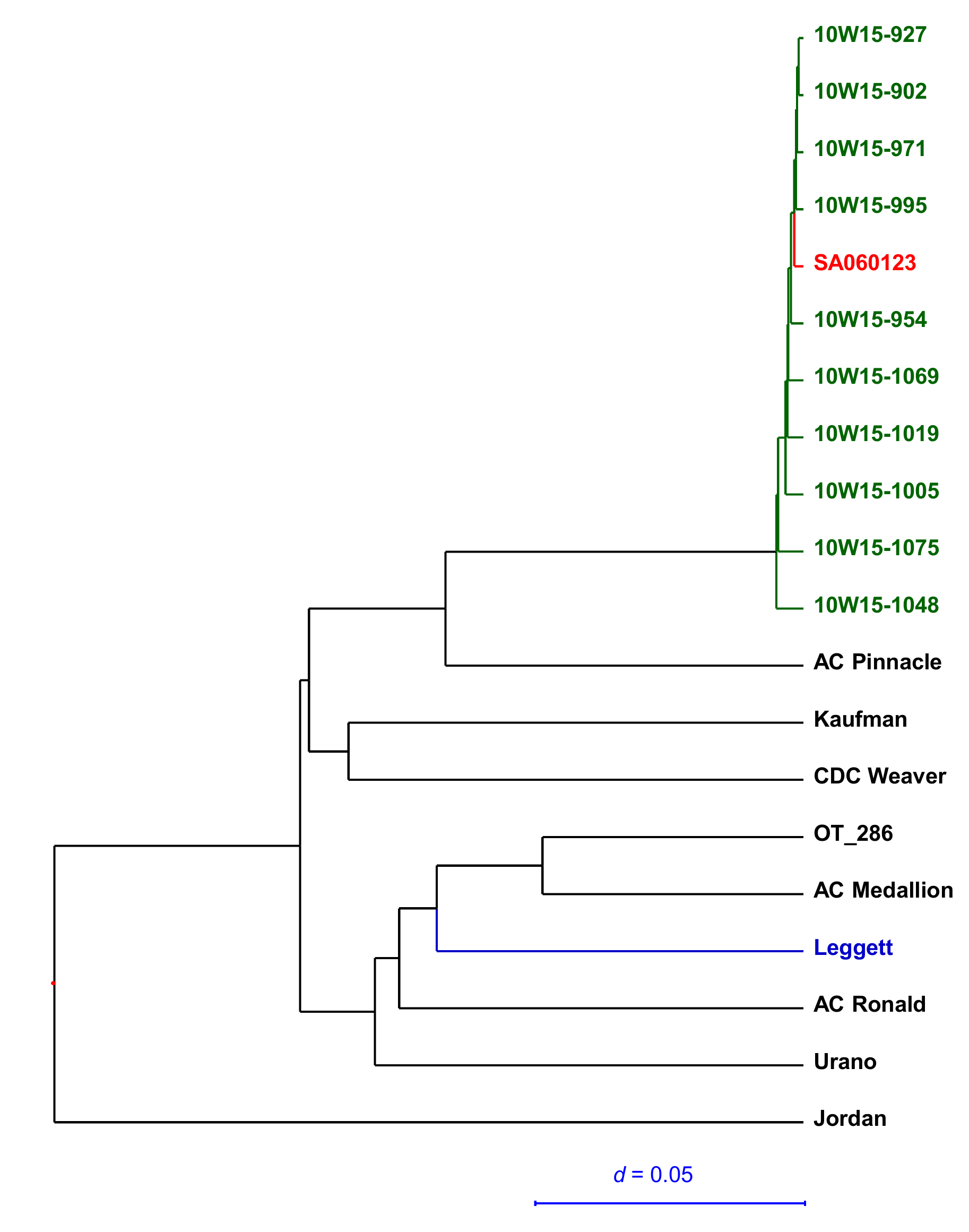

Supplement: Figure S15 — Using GBS markers to resolve an issue with breeding materials. UPGMA cluster analysis of simple allele-matching metric (d) based on 2205 GBS loci with >90% completeness. GBS calls were made across samples from 343 diverse oat varieties plus ten putative F2 segregants (green) from a putative cross between SA060123 (red) and a progeny of Leggett (blue). Eight closely related oat cultivars are also shown in this partial cluster dendrogram. Of the 2205 loci, only 131 (6%) showed any variation among the ten progeny plus SA060123, and this variation was within the expectations of heterozygous miscalls. This evidence was used to conclude that the ten progeny were actually from selfed seed of SA060123 rather than true segregants from a hybrid. (TIF) [file pone.0102448.s015.tif]

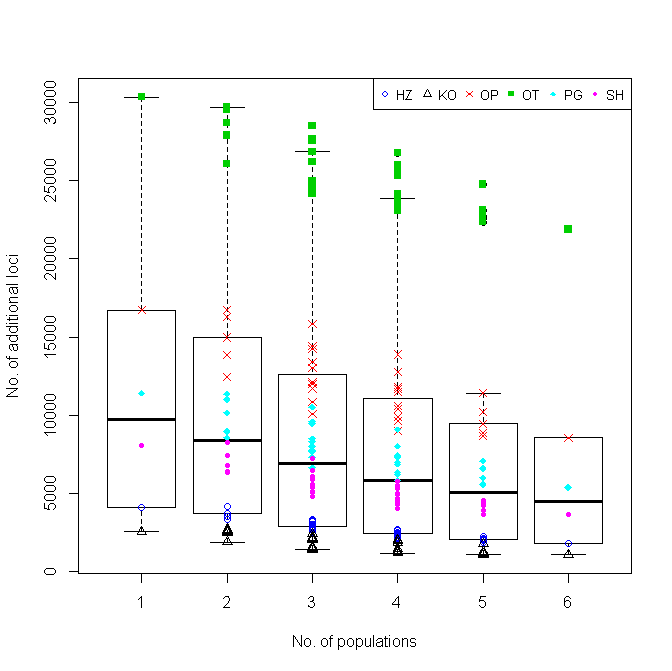

Supplement: Figure S16 — Effect of adding populations on the number of markers placed on the oat consensus map. Using the consensus map [4] as a framework and starting with a different population each time, markers from the six populations were placed sequentially in all possible combinations (, k = 1 to 6). The number of additional markers contributed by the final map at each step is represented by different colours and shapes. The box represents the range between the first and third quartiles and the thick horizontal bar represents the median. (TIFF) [file pone.0102448.s016.tiff]

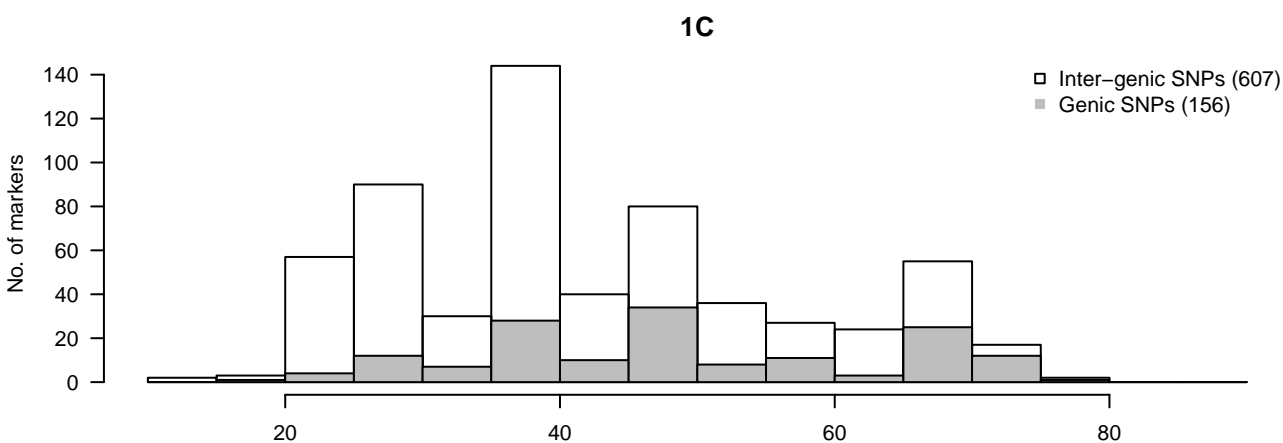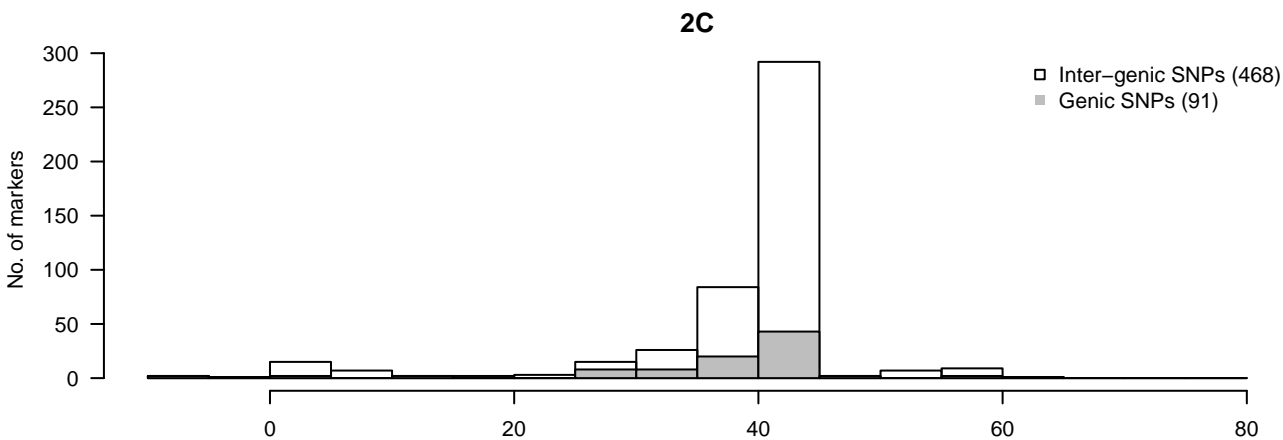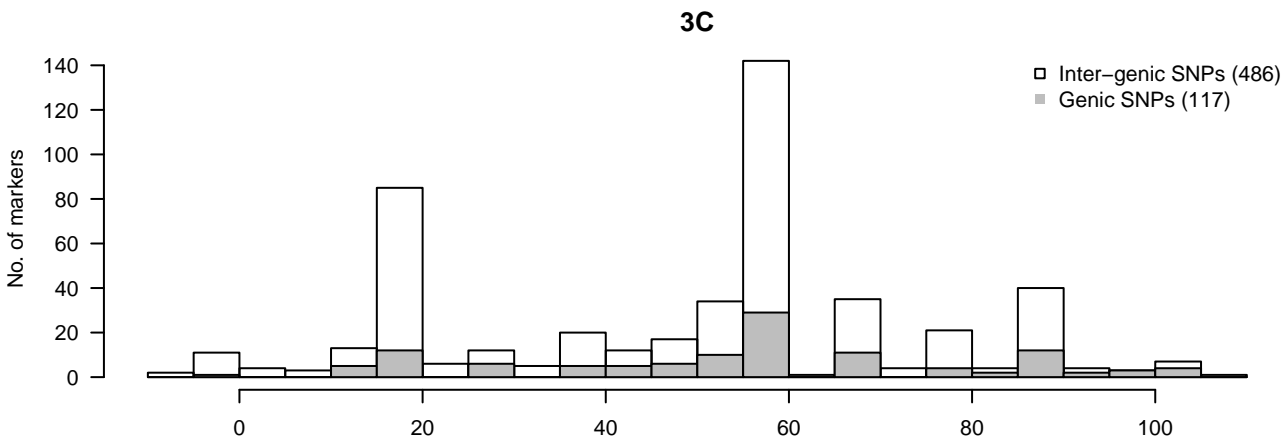

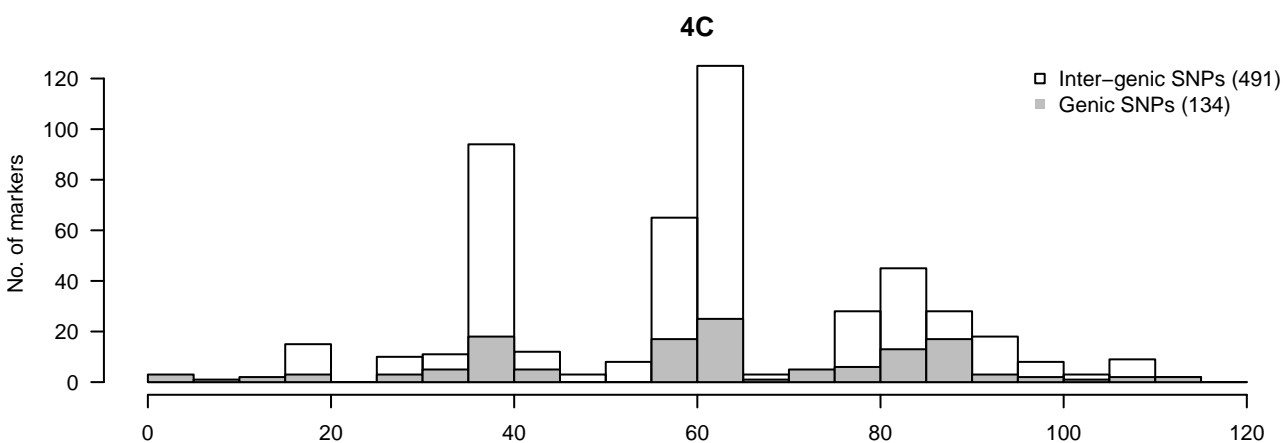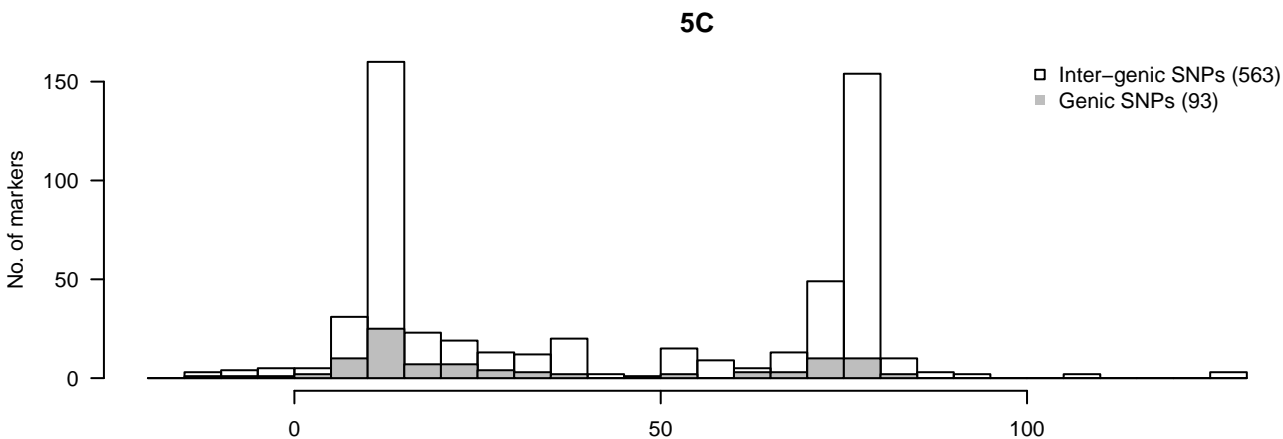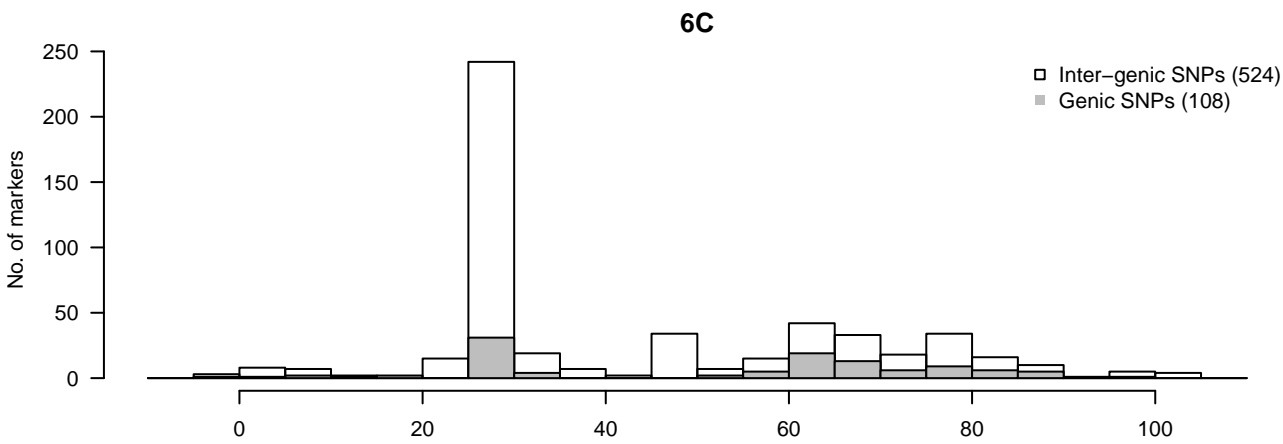

**7C-17A**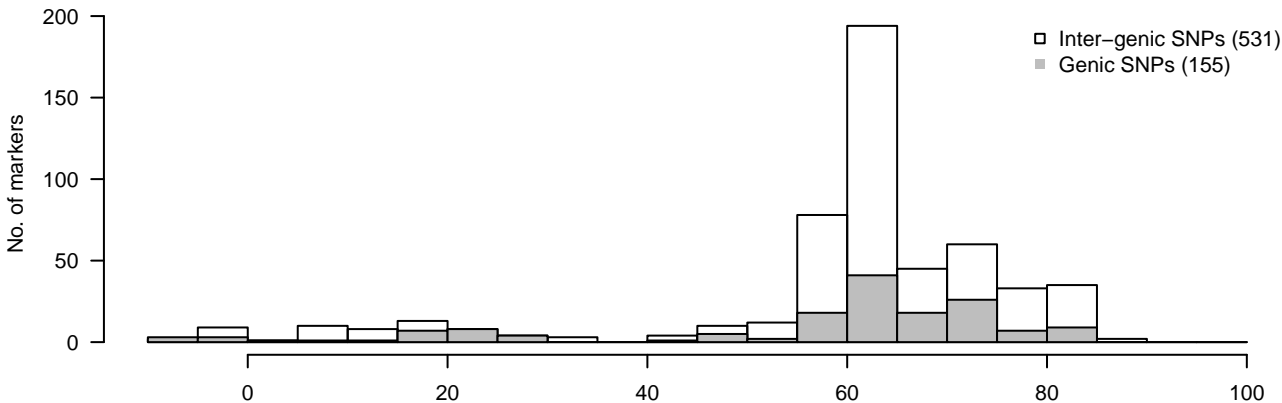**17A-7C**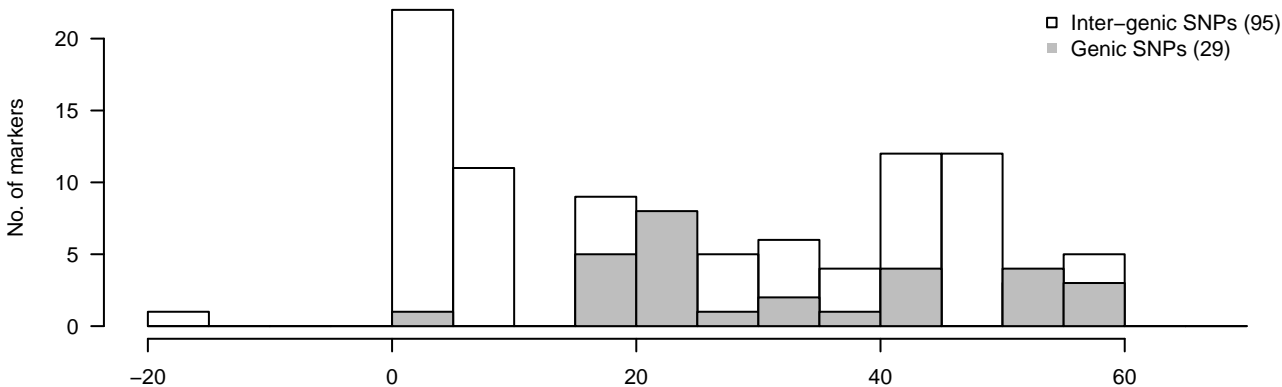**8A**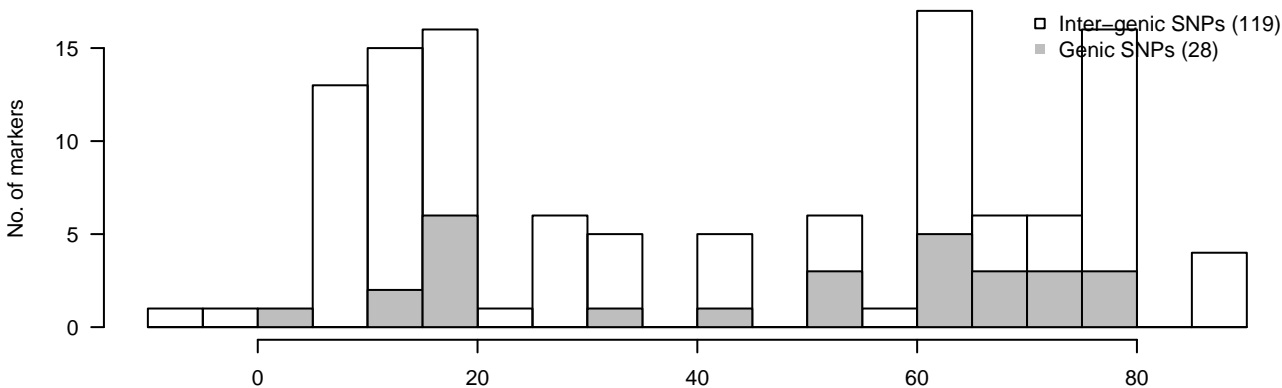

**11A**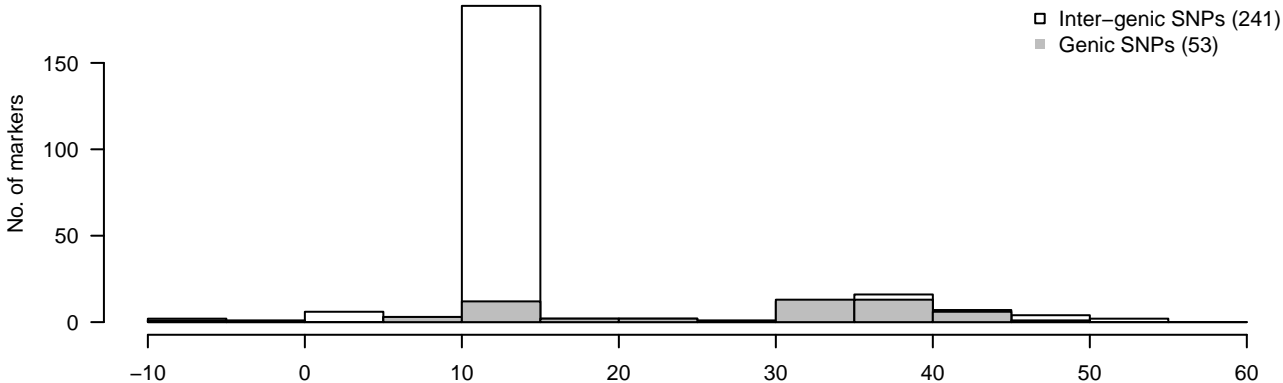**13A**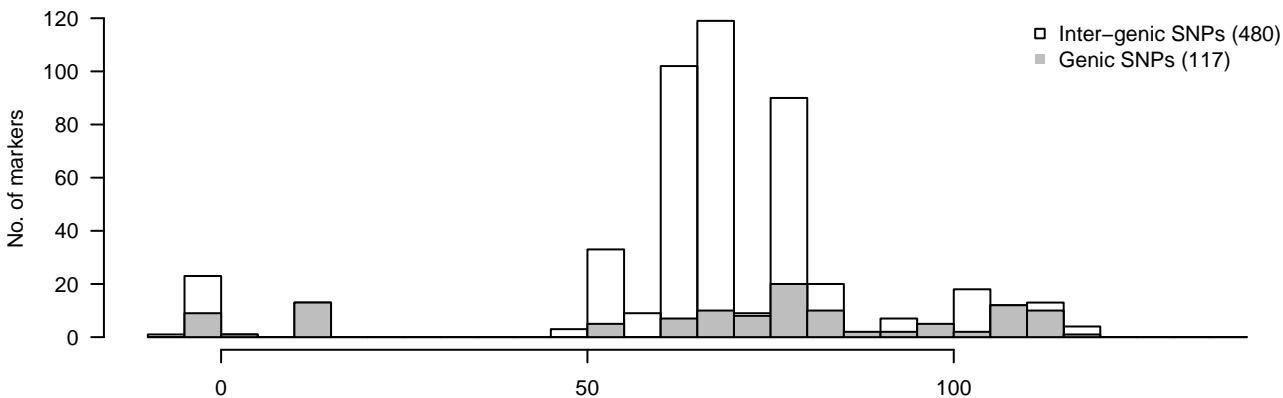**15A**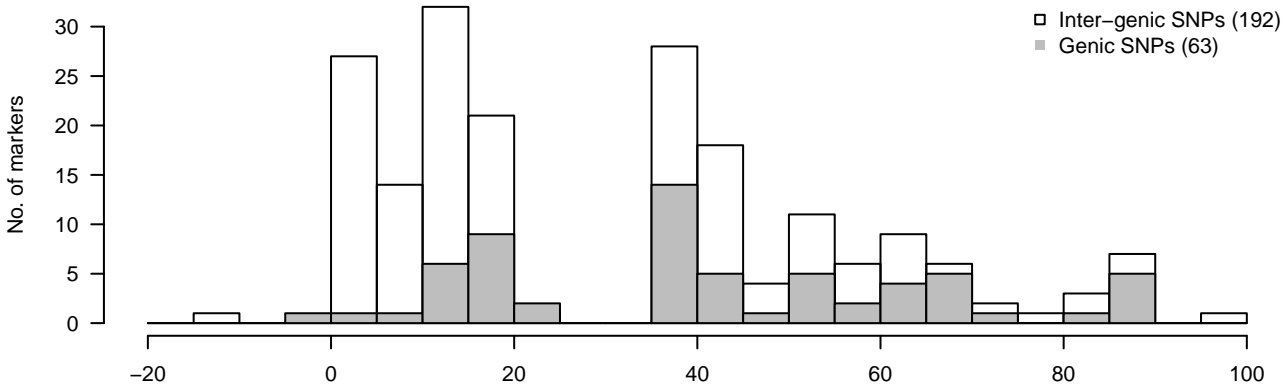

**16A**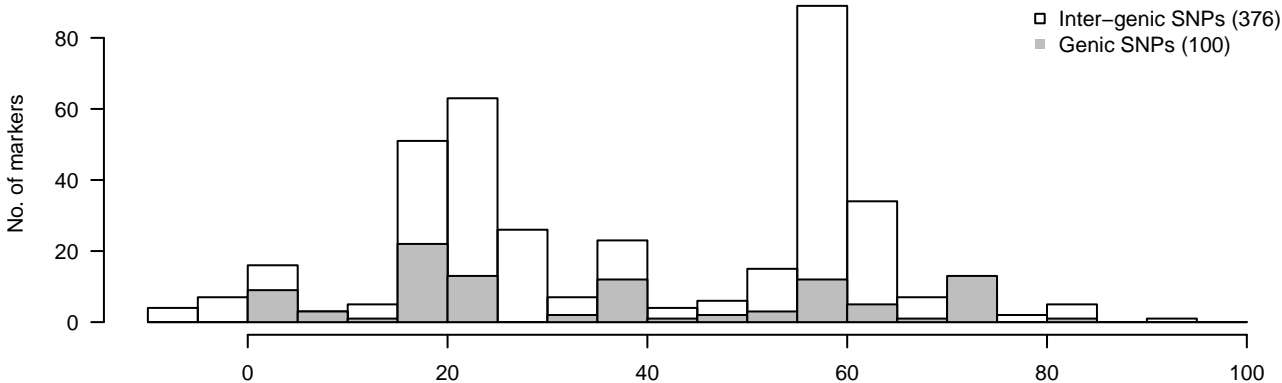**19A**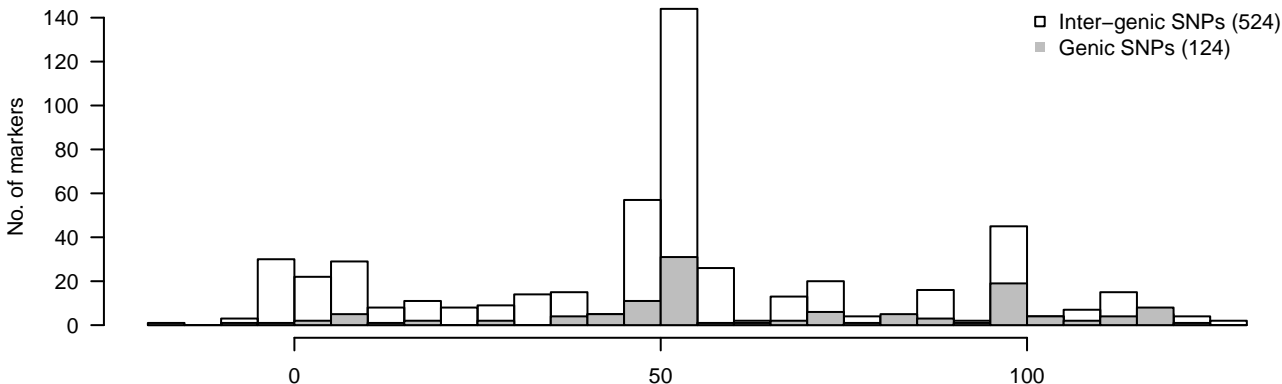**9D**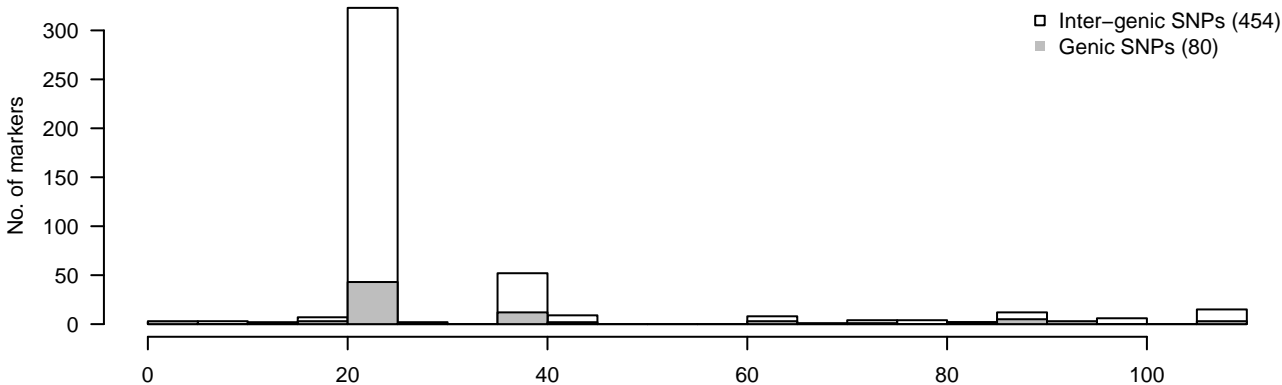

**10D-F-1**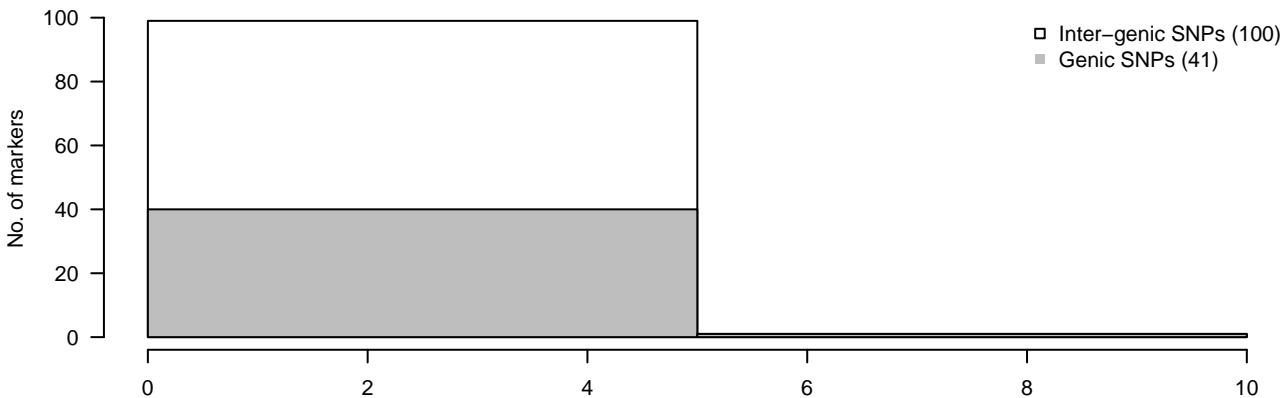**10D-F-2**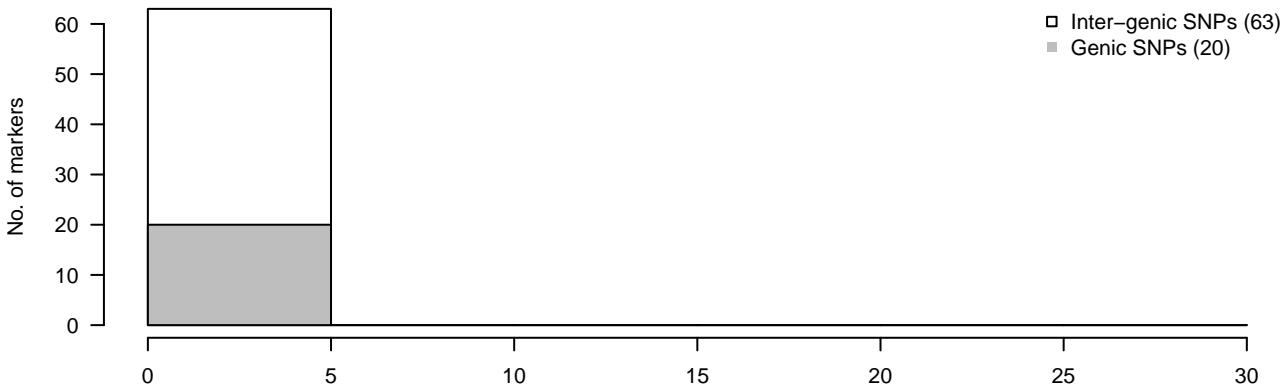**12D**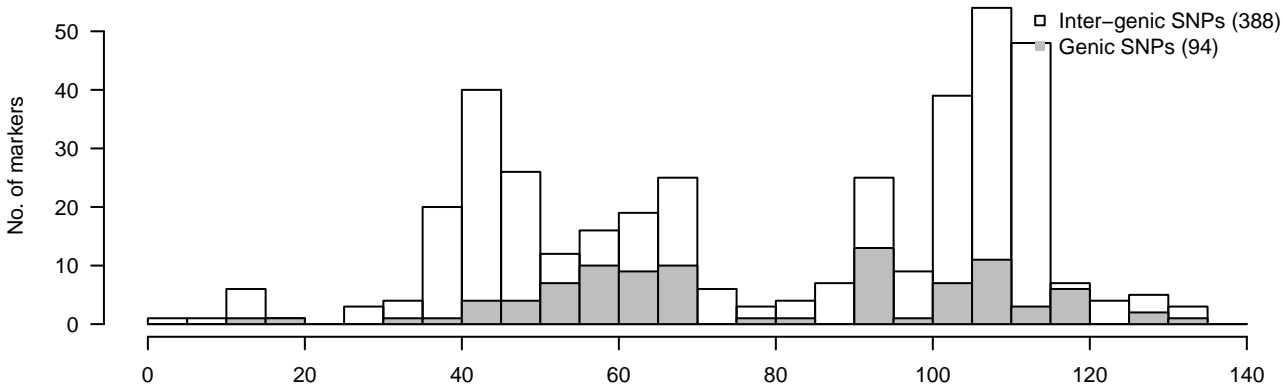

**14D**

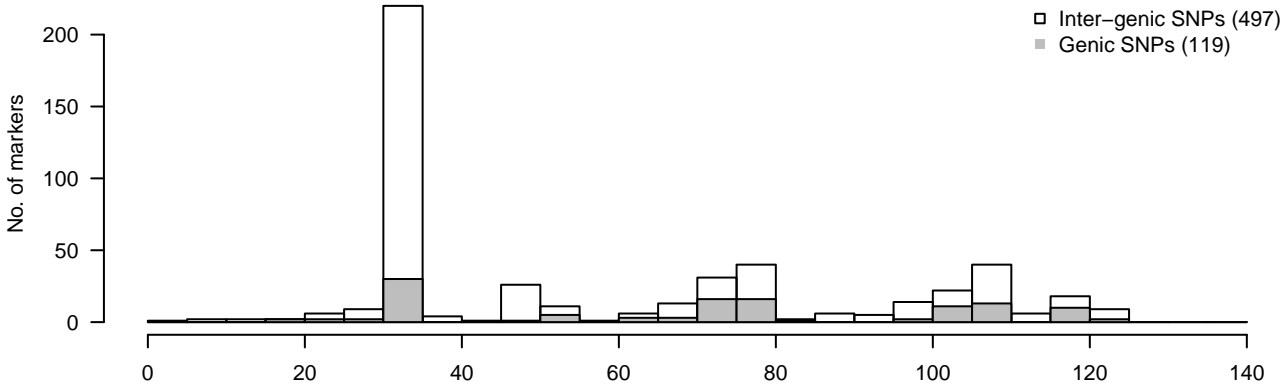

**18D**

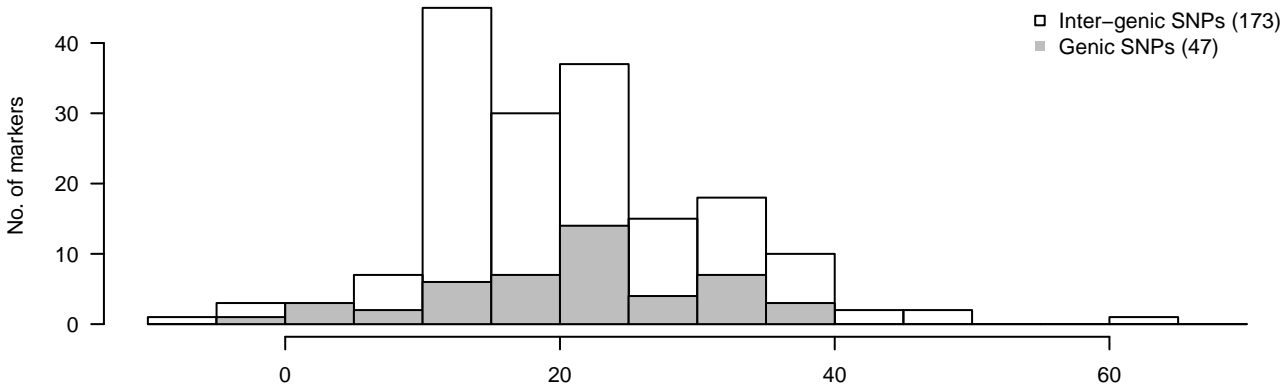

**20D**

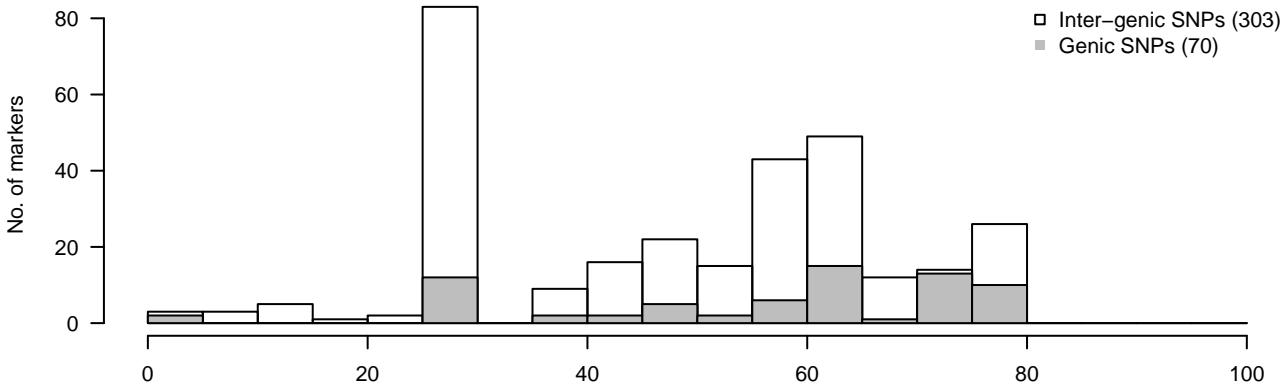

21D

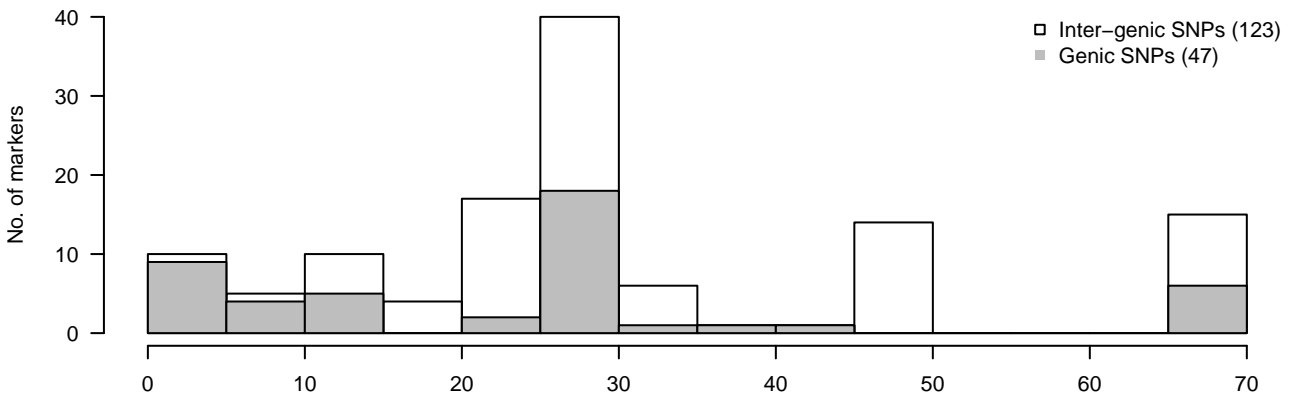

Supplement: Figure S17 — Distribution of annotated GBS markers across the oat consensus map. Maps of each chromosome were divided into 5 cM bins and the number of intergenic/genic markers counted for each bin. Some markers are in the negative range because they are placed off the beginning of the linkage group. (PDF) [file pone.0102448.s017.pdf]
