# Supplementary material for: Using Genotyping-By-Sequencing (GBS) for Genomic Discovery in Cultivated Oat
Source: PLoS One. 2014 Jul 21;9(7):e102448. doi: 10.1371/journal.pone.0102448 (PMC4105502; doi:10.1371/journal.pone.0102448)
Supplement: Text S2 — Updated oat consensus map (HTML_Local_text_S2.html). See Figure S1 for instructions. (HTML) [file pone.0102448.s023.html]

 
 
 
  SNPs AHOY!  
 
 
 SNPs AHOY: PseudoMap based on CORE consensus map 
 Reference:  PLoS ONE 8(3):e58068 
 
   Chromosome 1C    Chr   Pos   Framework   Placed SNP  Placed GBS  
   1C 9 
 
 
  avgbs_208881   
   1C 11 
 
 
  avgbs_116365   
   1C 12 
 
 
  avgbs_40683   
   1C 13 
  GMI_ES_CC14616_68    
 
  avgbs_120880    avgbs_120881    avgbs_230899    avgbs_122898    avgbs_89339    avgbs_200730   
   1C 14 
 
 
  avgbs_67597    avgbs_223393    avgbs_9484    avgbs_30957   
   1C 17 
 
 
  avgbs_232596    avgbs_6304   
   1C 18 
 
 
  avgbs_76396    avgbs_122182    avgbs_70531    avgbs_224665   
   1C 19 
 
 
  avgbs_16702    avgbs_209160    avgbs_6401    avgbs_33195    avgbs_106549    avgbs_75140    avgbs_200512    avgbs_842   
   1C 20 
 
 
  avgbs_225517    avgbs_66509    avgbs_96243   
   1C 21 
 
 
  avgbs_22302    avgbs_22303    avgbs_84371    avgbs_224994    avgbs_225774    avgbs_4282   
   1C 23 
 
 
  avgbs_62178    avgbs_85273   
   1C 24 
 
 
  avgbs_217273    avgbs_76108    avgbs_204020    avgbs_2679    avgbs_21445    avgbs_21447   
   1C 25 
  GMI_ES15_c3908_380     GMI_DS_CC9481_218    
 
  avgbs_4079    avgbs_4080    avgbs_16012    avgbs_16416    avgbs_17745    avgbs_18758    avgbs_20772    avgbs_28539    avgbs_42652    avgbs_42653    avgbs_42654    avgbs_43633    avgbs_55167    avgbs_66601    avgbs_67523    avgbs_69799    avgbs_69800    avgbs_71497    avgbs_73602    avgbs_73780    avgbs_79281    avgbs_83804    avgbs_86173    avgbs_87797    avgbs_89667    avgbs_92295    avgbs_93787    avgbs_94841    avgbs_105451    avgbs_106424    avgbs_110244    avgbs_110676    avgbs_110953    avgbs_112016    avgbs_112017    avgbs_112039    avgbs_112092    avgbs_116228    avgbs_116229    avgbs_116230    avgbs_122591    avgbs_122603    avgbs_124321    avgbs_234552    avgbs_18673    avgbs_235774    avgbs_204516    avgbs_238782    avgbs_238856    avgbs_207365    avgbs_207917    avgbs_208044    avgbs_208284    avgbs_41568    avgbs_64017    avgbs_88138    avgbs_88139    avgbs_99603    avgbs_124070    avgbs_1858    avgbs_1859    avgbs_2031    avgbs_3977    avgbs_16390    avgbs_20065    avgbs_33198    avgbs_36604    avgbs_39716    avgbs_39717    avgbs_40467    avgbs_43592    avgbs_43593    avgbs_49554    avgbs_50235    avgbs_55883    avgbs_61505    avgbs_62171    avgbs_66522    avgbs_66523    avgbs_68713    avgbs_71441    avgbs_74796    avgbs_80608    avgbs_81193    avgbs_81394    avgbs_86414    avgbs_86596    avgbs_87741    avgbs_99244    avgbs_100975    avgbs_105070    avgbs_110046    avgbs_111889    avgbs_111890    avgbs_118465    avgbs_125781    avgbs_125782    avgbs_202496    avgbs_203601    avgbs_203764    avgbs_203996    avgbs_205325    avgbs_205403    avgbs_240853    avgbs_206302    avgbs_241997    avgbs_243262    avgbs_207857    avgbs_49959    avgbs_55243    avgbs_55446    avgbs_65153    avgbs_67308    avgbs_71242    avgbs_107365    avgbs_203562    avgbs_203919    avgbs_204164    avgbs_205284    avgbs_207200    avgbs_11463    avgbs_42779    avgbs_48509    avgbs_62079    avgbs_62462    avgbs_91670    avgbs_92877    avgbs_98466    avgbs_100139    avgbs_201873    avgbs_38139    avgbs_203225    avgbs_203694    avgbs_204177    avgbs_204297    avgbs_205947    avgbs_206237    avgbs_35805    avgbs_36627    avgbs_69687    avgbs_74910    avgbs_95631    avgbs_203557    avgbs_204595    avgbs_24443    avgbs_30391    avgbs_31108    avgbs_38174    avgbs_43217    avgbs_43665    avgbs_43666    avgbs_54059    avgbs_58968    avgbs_89663    avgbs_93777    avgbs_103669    avgbs_121953    avgbs_125169    avgbs_201451    avgbs_202327    avgbs_203181    avgbs_203924    avgbs_204464    avgbs_240668    avgbs_206280    avgbs_206391    avgbs_42304    avgbs_66612    avgbs_206501    avgbs_10544    avgbs_39307    avgbs_43795    avgbs_48456    avgbs_61936    avgbs_67515    avgbs_77626    avgbs_79257    avgbs_111797    avgbs_201079    avgbs_201307    avgbs_202636    avgbs_202697    avgbs_236875    avgbs_203961    avgbs_237944    avgbs_204885    avgbs_205475    avgbs_52517    avgbs_106744    avgbs_202095    avgbs_235783    avgbs_206444    avgbs_58830    avgbs_69604    avgbs_74153    avgbs_110100    avgbs_110101    avgbs_113052    avgbs_113823    avgbs_205319    avgbs_15707    avgbs_22630    avgbs_36382    avgbs_49940    avgbs_58799    avgbs_85013    avgbs_202295    avgbs_238489    avgbs_125319    avgbs_58794    avgbs_90549    avgbs_109656    avgbs_110177    avgbs_112761    avgbs_202488    avgbs_203541    avgbs_241188    avgbs_207044    avgbs_207255    avgbs_15513    avgbs_22483    avgbs_46550    avgbs_55362    avgbs_62297    avgbs_77670    avgbs_106189    avgbs_108447    avgbs_201949    avgbs_236347    avgbs_239111    avgbs_240120    avgbs_206788    avgbs_43733    avgbs_43734    avgbs_81169    avgbs_89664    avgbs_96535    avgbs_202229    avgbs_236786    avgbs_238124    avgbs_239459    avgbs_90371    avgbs_90373    avgbs_125320    avgbs_205303    avgbs_24429    avgbs_31784    avgbs_60721    avgbs_101428    avgbs_108718    avgbs_201915    avgbs_20252    avgbs_42962    avgbs_52298    avgbs_102333    avgbs_235444    avgbs_203349    avgbs_204548    avgbs_204818    avgbs_206107    avgbs_207042    avgbs_6028    avgbs_34160    avgbs_63532    avgbs_74553    avgbs_76533    avgbs_79265    avgbs_84177    avgbs_84178    avgbs_99475    avgbs_104586    avgbs_204704    avgbs_208135    avgbs_6437    avgbs_27724    avgbs_28746    avgbs_58678    avgbs_201318    avgbs_201503    avgbs_202505    avgbs_205085    avgbs_207568    avgbs_208027    avgbs_32272    avgbs_32273    avgbs_32274    avgbs_37348    avgbs_52339    avgbs_53942    avgbs_68597    avgbs_74887    avgbs_81161    avgbs_82036    avgbs_91497    avgbs_93395    avgbs_95075    avgbs_204986    avgbs_206213    avgbs_55974    avgbs_64566    avgbs_71319    avgbs_76811    avgbs_100339    avgbs_103368    avgbs_201510    avgbs_202251    avgbs_205127    avgbs_239931    avgbs_41596    avgbs_69801    avgbs_69802    avgbs_88518    avgbs_88519    avgbs_201063    avgbs_207616    avgbs_207973    avgbs_18457    avgbs_90782    avgbs_91222    avgbs_103164    avgbs_118997    avgbs_203092    avgbs_203214    avgbs_204275    avgbs_239141    avgbs_40254    avgbs_60990    avgbs_66357    avgbs_74348    avgbs_94742    avgbs_103554    avgbs_202917    avgbs_203022    avgbs_237123    avgbs_1911    avgbs_3738    avgbs_52486    avgbs_103546    avgbs_236692    avgbs_237946    avgbs_208023    avgbs_56409    avgbs_234077    avgbs_234270    avgbs_241526    avgbs_206342    avgbs_242917    avgbs_202458    avgbs_236444    avgbs_204816    avgbs_238789    avgbs_37677    avgbs_49532    avgbs_89241    avgbs_89242    avgbs_103118    avgbs_59445    avgbs_204113    avgbs_206663    avgbs_241556    avgbs_234026    avgbs_62144    avgbs_237963    avgbs_25784    avgbs_203051    avgbs_38109    avgbs_7698    avgbs_225096    avgbs_125767    avgbs_126202    avgbs_24440    avgbs_225006    avgbs_27940    avgbs_223762    avgbs_223775    avgbs_4700   
   1C 26 
  GMI_ES02_c501_285     GMI_ES_CC13854_225    
 
  avgbs_217721    avgbs_43249    avgbs_98869    avgbs_16315    avgbs_47734    avgbs_66642    avgbs_72184    avgbs_72185    avgbs_94036    avgbs_113985    avgbs_115014    avgbs_118602    avgbs_118603    avgbs_123060    avgbs_200455    avgbs_232474    avgbs_86359    avgbs_231461    avgbs_6364    avgbs_11140    avgbs_11141    avgbs_34586    avgbs_35340    avgbs_35401    avgbs_56262    avgbs_56263    avgbs_62906    avgbs_72741    avgbs_73789    avgbs_78548    avgbs_82407    avgbs_86297    avgbs_87973    avgbs_89734    avgbs_89735    avgbs_89736    avgbs_90523    avgbs_90862    avgbs_90921    avgbs_90922    avgbs_95680    avgbs_98571    avgbs_98888    avgbs_98889    avgbs_98890    avgbs_98891    avgbs_105738    avgbs_107404    avgbs_110249    avgbs_121673    avgbs_200238    avgbs_230432    avgbs_230896    avgbs_200556    avgbs_231317    avgbs_224953    avgbs_231500    avgbs_231637    avgbs_200826    avgbs_232429    avgbs_232502    avgbs_216072    avgbs_225806    avgbs_378    avgbs_6K_6338    avgbs_33261    avgbs_45113    avgbs_51381    avgbs_51382    avgbs_54201    avgbs_72818    avgbs_77721    avgbs_84954    avgbs_116768    avgbs_212689    avgbs_913    avgbs_16502    avgbs_34692    avgbs_41196    avgbs_41197    avgbs_81524    avgbs_81525    avgbs_10358    avgbs_20760    avgbs_46760    avgbs_60017    avgbs_62298    avgbs_71562    avgbs_72894    avgbs_85401    avgbs_122348    avgbs_234076    avgbs_202499    avgbs_203471    avgbs_203960    avgbs_205198    avgbs_205268    avgbs_74728    avgbs_47715    avgbs_66492    avgbs_62523    avgbs_233334    avgbs_8169    avgbs_51642    avgbs_114766    avgbs_125970    avgbs_207284    avgbs_18575    avgbs_36688    avgbs_121076    avgbs_121077    avgbs_95999    avgbs_202914    avgbs_214819    avgbs_27939    avgbs_203864    avgbs_110680    avgbs_204445    avgbs_215785   
   1C 27 
  GMI_ES15_c16679_330    
 
  avgbs_213704    avgbs_26491    avgbs_27863    avgbs_37495    avgbs_37496    avgbs_37497    avgbs_40901    avgbs_111703    avgbs_114633    avgbs_213025    avgbs_213668    avgbs_13195    avgbs_97902    avgbs_215441    avgbs_39489    avgbs_39490    avgbs_62759    avgbs_75633    avgbs_92375    avgbs_100027    avgbs_114246    avgbs_218697    avgbs_222555    avgbs_90649    avgbs_121142    avgbs_121143    avgbs_218980    avgbs_219287    avgbs_20266    avgbs_68013    avgbs_68014    avgbs_10633    avgbs_108446    avgbs_119007    avgbs_14568    avgbs_24706    avgbs_45500    avgbs_217887    avgbs_26797    avgbs_66552    avgbs_25731    avgbs_36826    avgbs_36827    avgbs_55568    avgbs_72745    avgbs_94076    avgbs_96887    avgbs_115734    avgbs_124581    avgbs_200369    avgbs_225631    avgbs_212968    avgbs_3635    avgbs_8093    avgbs_55528    avgbs_203840    avgbs_242704    avgbs_39270    avgbs_46411    avgbs_46412    avgbs_71563    avgbs_76183    avgbs_115349    avgbs_125067    avgbs_125068    avgbs_241581    avgbs_78105    avgbs_78106    avgbs_201161    avgbs_203018    avgbs_21563    avgbs_122431    avgbs_208030    avgbs_100602    avgbs_85419    avgbs_23632    avgbs_94075    avgbs_225199    avgbs_76071    avgbs_76072    avgbs_119214    avgbs_213646    avgbs_85453    avgbs_85454    avgbs_86534    avgbs_205316    avgbs_39810    avgbs_225646    avgbs_6269    avgbs_113055    avgbs_214022    avgbs_21876    avgbs_21877    avgbs_39243    avgbs_76780    avgbs_116322    avgbs_203178    avgbs_213964    avgbs_225127    avgbs_225642    avgbs_39639    avgbs_88149    avgbs_225063    avgbs_206191    avgbs_36473    avgbs_99564    avgbs_109648    avgbs_112129    avgbs_215530    avgbs_6K_97720    avgbs_122163    avgbs_10013    avgbs_77054    avgbs_103002    avgbs_102617    avgbs_102618    avgbs_10782    avgbs_94719    avgbs_50895    avgbs_213478    avgbs_215406    avgbs_47762    avgbs_115577    avgbs_215481    avgbs_216059    avgbs_76637    avgbs_6K_97971   
   1C 28 
  GMI_ES_CC11019_290     GMI_ES15_c10106_179    
 
  avgbs_84336    avgbs_39683    avgbs_214809    avgbs_205272    avgbs_215158    avgbs_19299    avgbs_20716    avgbs_20719    avgbs_48585    avgbs_48586    avgbs_213537    avgbs_204550    avgbs_221993    avgbs_97989    avgbs_13212    avgbs_13213    avgbs_91051    avgbs_221197    avgbs_92882    avgbs_110163    avgbs_126213    avgbs_205035    avgbs_208393    avgbs_7230    avgbs_33940    avgbs_44053    avgbs_49070    avgbs_51795    avgbs_77476    avgbs_6K_77720    avgbs_94262    avgbs_107777    avgbs_112237    avgbs_120358    avgbs_125840    avgbs_237811    avgbs_205295    avgbs_206279    avgbs_29872    avgbs_92206    avgbs_99592    avgbs_99593    avgbs_99594    avgbs_105933    avgbs_110156    avgbs_204120    avgbs_206350    avgbs_66613    avgbs_66614    avgbs_120919    avgbs_202186    avgbs_240292    avgbs_206975    avgbs_236751    avgbs_10045    avgbs_10046    avgbs_18058    avgbs_40764    avgbs_56210    avgbs_74613    avgbs_74614    avgbs_240722    avgbs_243791    avgbs_58792    avgbs_101349    avgbs_113782    avgbs_208279    avgbs_16397    avgbs_16399    avgbs_73041    avgbs_89838    avgbs_40745    avgbs_110605    avgbs_2376    avgbs_203232    avgbs_108623    avgbs_237275    avgbs_26930    avgbs_236744    avgbs_206636    avgbs_51007    avgbs_237191    avgbs_206906    avgbs_207113    avgbs_31075    avgbs_235977    avgbs_25364    avgbs_71269    avgbs_74903    avgbs_24143    avgbs_202673    avgbs_85400    avgbs_205169    avgbs_29497    avgbs_29498    avgbs_54572    avgbs_79706    avgbs_204444    avgbs_68381    avgbs_214680    avgbs_214109    avgbs_12812    avgbs_4734    avgbs_12436    avgbs_12437    avgbs_15135    avgbs_121669    avgbs_121670    avgbs_84337    avgbs_84338    avgbs_81485    avgbs_215036    avgbs_108137    avgbs_119452    avgbs_51851    avgbs_100734    avgbs_201105    avgbs_113990    avgbs_115098   
   1C 29 
 
 
  avgbs_26334    avgbs_26335    avgbs_28690    avgbs_28691    avgbs_57462    avgbs_57463    avgbs_71520    avgbs_221276    avgbs_221705    avgbs_1421    avgbs_75716    avgbs_124736    avgbs_221474    avgbs_223106    avgbs_52604    avgbs_107276    avgbs_218728    avgbs_17828    avgbs_55159    avgbs_55161    avgbs_217173    avgbs_217194    avgbs_7287    avgbs_117943    avgbs_117944    avgbs_90386    avgbs_45702    avgbs_218625    avgbs_23474    avgbs_18267    avgbs_44827    avgbs_45018    avgbs_88321    avgbs_203736    avgbs_35947    avgbs_203078    avgbs_203152    avgbs_6000    avgbs_7606    avgbs_10181    avgbs_15871    avgbs_18871    avgbs_21193    avgbs_21194    avgbs_23813    avgbs_25856    avgbs_29264    avgbs_34622    avgbs_39761    avgbs_80502    avgbs_84151    avgbs_89796    avgbs_92888    avgbs_95291    avgbs_97957    avgbs_99556    avgbs_101051    avgbs_101052    avgbs_106795    avgbs_110125    avgbs_111167    avgbs_112458    avgbs_112771    avgbs_116117    avgbs_118270    avgbs_201653    avgbs_238600    avgbs_204959    avgbs_205139    avgbs_205430    avgbs_205468    avgbs_205966    avgbs_205970    avgbs_242286    avgbs_206862    avgbs_242751    avgbs_39763    avgbs_88400    avgbs_204243    avgbs_205039    avgbs_239889    avgbs_207549    avgbs_54156    avgbs_61699    avgbs_81619    avgbs_204004    avgbs_64649    avgbs_203209    avgbs_37934    avgbs_81076    avgbs_206470    avgbs_51788    avgbs_126068    avgbs_206917    avgbs_204999    avgbs_238393    avgbs_119220    avgbs_106266   
   1C 30 
 
 
  avgbs_46869    avgbs_218674    avgbs_220673    avgbs_8465    avgbs_75696    avgbs_220936    avgbs_222216    avgbs_25258    avgbs_222184    avgbs_15969    avgbs_68012    avgbs_219958    avgbs_46734    avgbs_46735    avgbs_217467    avgbs_7288    avgbs_218810    avgbs_204652    avgbs_125093    avgbs_125094   
   1C 31 
  GMI_DS_CC9736_146    
 
  avgbs_219117    avgbs_2565    avgbs_8200    avgbs_111249    avgbs_218385    avgbs_30367    avgbs_62758    avgbs_39701    avgbs_209891    avgbs_5220    avgbs_5221    avgbs_10224    avgbs_14332    avgbs_25280    avgbs_27937    avgbs_37914    avgbs_38141    avgbs_55114    avgbs_55115    avgbs_66084    avgbs_121516    avgbs_51685    avgbs_201309    avgbs_1721    avgbs_3577    avgbs_4154    avgbs_5951    avgbs_6764    avgbs_13639    avgbs_16480    avgbs_16485    avgbs_17158    avgbs_17328    avgbs_20039    avgbs_21890    avgbs_23706    avgbs_23707    avgbs_26814    avgbs_27936    avgbs_30182    avgbs_30183    avgbs_30614    avgbs_31039    avgbs_31604    avgbs_34623    avgbs_35730    avgbs_35844    avgbs_35845    avgbs_35846    avgbs_36679    avgbs_44994    avgbs_48327    avgbs_53034    avgbs_60378    avgbs_62376    avgbs_62840    avgbs_62841    avgbs_63644    avgbs_63645    avgbs_65477    avgbs_65478    avgbs_68711    avgbs_72420    avgbs_74734    avgbs_78763    avgbs_80438    avgbs_80614    avgbs_80861    avgbs_82019    avgbs_85213    avgbs_86145    avgbs_87591    avgbs_96106    avgbs_96714    avgbs_97126    avgbs_104659    avgbs_104660    avgbs_105580    avgbs_105680    avgbs_105868    avgbs_106031    avgbs_106739    avgbs_107899    avgbs_107900    avgbs_110085    avgbs_111372    avgbs_112786    avgbs_114478    avgbs_114480    avgbs_115640    avgbs_119949    avgbs_121976    avgbs_122521    avgbs_122931    avgbs_124221    avgbs_201205    avgbs_201644    avgbs_202605    avgbs_202765    avgbs_236241    avgbs_203054    avgbs_203223    avgbs_203904    avgbs_203964    avgbs_204028    avgbs_204298    avgbs_204314    avgbs_205427    avgbs_205437    avgbs_205687    avgbs_240948    avgbs_206442    avgbs_207199    avgbs_207739    avgbs_244017    avgbs_208058    avgbs_208094    avgbs_431    avgbs_6070    avgbs_24132    avgbs_24134    avgbs_24296    avgbs_24297    avgbs_39330    avgbs_43709    avgbs_64954    avgbs_102887    avgbs_114146    avgbs_119249    avgbs_202680    avgbs_203995    avgbs_205431    avgbs_4148    avgbs_6756    avgbs_21738    avgbs_32652    avgbs_40419    avgbs_54310    avgbs_85981    avgbs_96160    avgbs_112024    avgbs_120722    avgbs_201203    avgbs_205256    avgbs_206620    avgbs_207188    avgbs_207264    avgbs_207429    avgbs_19932    avgbs_63741    avgbs_98075    avgbs_98076    avgbs_201501    avgbs_203359    avgbs_205698    avgbs_2155    avgbs_23588    avgbs_51559    avgbs_70268    avgbs_70269    avgbs_74685    avgbs_124087    avgbs_206225    avgbs_32697    avgbs_237034    avgbs_206970    avgbs_26580    avgbs_40015    avgbs_52540    avgbs_112814    avgbs_206730    avgbs_207047    avgbs_32653    avgbs_62845    avgbs_62846    avgbs_104505    avgbs_204077    avgbs_8167    avgbs_201507    avgbs_61463    avgbs_8596    avgbs_28357    avgbs_53993    avgbs_84701    avgbs_203259    avgbs_236629    avgbs_203311    avgbs_203813    avgbs_239740    avgbs_27624    avgbs_27625    avgbs_203183    avgbs_204577    avgbs_241916    avgbs_207137    avgbs_208220    avgbs_61377    avgbs_237910    avgbs_204720    avgbs_207873    avgbs_3052    avgbs_58791    avgbs_241816    avgbs_85709    avgbs_101998    avgbs_122161    avgbs_122162    avgbs_203004    avgbs_203195    avgbs_238863    avgbs_208427    avgbs_57084    avgbs_58831    avgbs_67185    avgbs_232898    avgbs_243201    avgbs_74795    avgbs_78953    avgbs_203208    avgbs_204623    avgbs_31156    avgbs_31157    avgbs_103023    avgbs_235723    avgbs_24713    avgbs_28427    avgbs_235767    avgbs_94895    avgbs_235488    avgbs_205877    avgbs_201301    avgbs_236973    avgbs_239773    avgbs_206734    avgbs_207358    avgbs_208403    avgbs_241593    avgbs_24407    avgbs_51499    avgbs_57096    avgbs_78481    avgbs_236056    avgbs_26106    avgbs_235643    avgbs_207174   
   1C 32 
 
 
  avgbs_109645    avgbs_219797    avgbs_33376    avgbs_70732    avgbs_52572    avgbs_106717    avgbs_36584    avgbs_223394    avgbs_224825   
   1C 33 
 
 
  avgbs_218369    avgbs_104803    avgbs_104804    avgbs_9726    avgbs_27311    avgbs_201614    avgbs_16131    avgbs_217263    avgbs_70469    avgbs_4333    avgbs_4335    avgbs_14839    avgbs_34367    avgbs_73096    avgbs_202974    avgbs_16336    avgbs_208266   
   1C 34 
 
 
  avgbs_89343    avgbs_52602    avgbs_59616    avgbs_204010    avgbs_207654    avgbs_40746    avgbs_59015    avgbs_214417   
   1C 35 
 
 
  avgbs_38658    avgbs_12687    avgbs_216964    avgbs_205593    avgbs_58135    avgbs_29990    avgbs_223816    avgbs_6K_77337    avgbs_224973    avgbs_223547   
   1C 36 
  GMI_ES15_c8836_313     GMI_DS_CC2357_196     GMI_ES01_c2338_213     GMI_ES17_c6299_272    
 
  avgbs_13702    avgbs_120262    avgbs_103518    avgbs_1402    avgbs_4173    avgbs_13701    avgbs_15127    avgbs_48193    avgbs_57511    avgbs_67579    avgbs_68615    avgbs_84646    avgbs_87030    avgbs_93935    avgbs_106104    avgbs_106906    avgbs_109644    avgbs_110401    avgbs_111172    avgbs_111173    avgbs_111208    avgbs_111370    avgbs_112306    avgbs_124871    avgbs_202065    avgbs_202708    avgbs_226467    avgbs_203203    avgbs_214162    avgbs_214387    avgbs_204265    avgbs_239734    avgbs_30337    avgbs_68223    avgbs_23256    avgbs_23257    avgbs_15044    avgbs_15045    avgbs_108909    avgbs_6344    avgbs_37094    avgbs_76080    avgbs_6460    avgbs_42137    avgbs_11718    avgbs_28333    avgbs_55133    avgbs_56344    avgbs_56345    avgbs_86082    avgbs_86083    avgbs_201961    avgbs_233714    avgbs_84991    avgbs_40618    avgbs_40619    avgbs_47395    avgbs_207388    avgbs_206504    avgbs_3149    avgbs_236362    avgbs_240450    avgbs_30477    avgbs_30478    avgbs_44337    avgbs_96778    avgbs_237716    avgbs_205669    avgbs_53596    avgbs_84694    avgbs_223598    avgbs_58065    avgbs_225016    avgbs_216046    avgbs_6K_55792    avgbs_115067    avgbs_85309    avgbs_91845    avgbs_60597    avgbs_62819    avgbs_62839    avgbs_62842    avgbs_63720    avgbs_111955    avgbs_112787    avgbs_115391    avgbs_215259    avgbs_15430    avgbs_15431    avgbs_16867    avgbs_23704    avgbs_24269    avgbs_24270    avgbs_27293    avgbs_36251    avgbs_36252    avgbs_36590    avgbs_36591    avgbs_49828    avgbs_51604    avgbs_67452    avgbs_79818    avgbs_110332    avgbs_22312    avgbs_46856    avgbs_46857    avgbs_57051    avgbs_79819    avgbs_893    avgbs_27471    avgbs_27472    avgbs_36432    avgbs_40418    avgbs_46648    avgbs_46649    avgbs_52203    avgbs_84661    avgbs_86179    avgbs_88370    avgbs_100609    avgbs_114169    avgbs_215613    avgbs_3399    avgbs_76845    avgbs_11185    avgbs_11275    avgbs_16212    avgbs_17643    avgbs_17652    avgbs_23708    avgbs_27022    avgbs_36675    avgbs_36676    avgbs_36695    avgbs_47338    avgbs_58787    avgbs_73084    avgbs_77637    avgbs_80337    avgbs_84567    avgbs_92360    avgbs_93092    avgbs_100801    avgbs_103570    avgbs_114635    avgbs_117108    avgbs_213452    avgbs_214416    avgbs_215732    avgbs_215765    avgbs_215886    avgbs_38514    avgbs_213940    avgbs_445    avgbs_4728    avgbs_9214    avgbs_10613    avgbs_11272    avgbs_14680    avgbs_20863    avgbs_21481    avgbs_22560    avgbs_24039    avgbs_35973    avgbs_36805    avgbs_47552    avgbs_49870    avgbs_49871    avgbs_51238    avgbs_56247    avgbs_56248    avgbs_68433    avgbs_72421    avgbs_79866    avgbs_82039    avgbs_82057    avgbs_82118    avgbs_6K_80350    avgbs_92214    avgbs_96836    avgbs_97864    avgbs_100932    avgbs_112459    avgbs_115053    avgbs_117055    avgbs_118220    avgbs_213027    avgbs_213179    avgbs_213250    avgbs_213553    avgbs_213898    avgbs_214485    avgbs_214495    avgbs_215112    avgbs_215497    avgbs_215519    avgbs_81198    avgbs_214239   
   1C 37 
  GMI_ES01_c13467_233     GMI_DS_CC5128_138     GMI_ES01_c3178_185     GMI_ES_CC17647_179     GMI_ES02_c3604_154     GMI_DS_CC4006_319     GMI_ES02_c28832_308     GMI_ES17_c20652_599     GMI_ES17_c13450_151     GMI_ES17_c12492_273     GMI_ES17_c17467_410     GMI_ES17_c4730_358    
 
  avgbs_60200    avgbs_60201    avgbs_219854    avgbs_10776    avgbs_74609    avgbs_99285    avgbs_52391    avgbs_124222    avgbs_17299    avgbs_18717    avgbs_20925    avgbs_38134    avgbs_38135    avgbs_40684    avgbs_43383    avgbs_43789    avgbs_45455    avgbs_46051    avgbs_62437    avgbs_63613    avgbs_101446    avgbs_101447    avgbs_110161    avgbs_112114    avgbs_201641    avgbs_213531    avgbs_213978    avgbs_203414    avgbs_203423    avgbs_204088    avgbs_86136    avgbs_88040    avgbs_95649    avgbs_114011    avgbs_117090    avgbs_240829    avgbs_206330    avgbs_242970    avgbs_74894    avgbs_12060    avgbs_15718    avgbs_236509    avgbs_74869    avgbs_74870    avgbs_108269    avgbs_207951    avgbs_81427    avgbs_203421    avgbs_49471    avgbs_64309    avgbs_103967    avgbs_20844    avgbs_52189    avgbs_87268    avgbs_77044    avgbs_94878    avgbs_17483    avgbs_81371    avgbs_81372    avgbs_6163    avgbs_36141    avgbs_47721    avgbs_63401    avgbs_111128    avgbs_98049    avgbs_225499    avgbs_108166    avgbs_213760    avgbs_213169    avgbs_46645    avgbs_115262    avgbs_35940    avgbs_26942    avgbs_58618    avgbs_40315    avgbs_106866    avgbs_11370    avgbs_46232    avgbs_65805    avgbs_68076    avgbs_95524    avgbs_110999    avgbs_214123    avgbs_85690    avgbs_107398    avgbs_36468    avgbs_45302    avgbs_58181    avgbs_68333    avgbs_88102    avgbs_108750    avgbs_108751    avgbs_17159    avgbs_29375    avgbs_31386    avgbs_36744    avgbs_87519    avgbs_102126    avgbs_102127    avgbs_106885    avgbs_110405    avgbs_215776    avgbs_215930    avgbs_12807    avgbs_36660    avgbs_95648    avgbs_111157    avgbs_2391    avgbs_5933    avgbs_7551    avgbs_7552    avgbs_8617    avgbs_12099    avgbs_13456    avgbs_16404    avgbs_19973    avgbs_19974    avgbs_21441    avgbs_27320    avgbs_51946    avgbs_53425    avgbs_53426    avgbs_56284    avgbs_77477    avgbs_81756    avgbs_85851    avgbs_91944    avgbs_91945    avgbs_92233    avgbs_92234    avgbs_96660    avgbs_110086    avgbs_110087    avgbs_215018    avgbs_96645    avgbs_215437    avgbs_2475    avgbs_10875    avgbs_10876    avgbs_10877    avgbs_12353    avgbs_17751    avgbs_19288    avgbs_23263    avgbs_23264    avgbs_24994    avgbs_28706    avgbs_30210    avgbs_30427    avgbs_34791    avgbs_34793    avgbs_36132    avgbs_36133    avgbs_36447    avgbs_37203    avgbs_42592    avgbs_45678    avgbs_52762    avgbs_56737    avgbs_61535    avgbs_71629    avgbs_71630    avgbs_77618    avgbs_86218    avgbs_89678    avgbs_89803    avgbs_90764    avgbs_92561    avgbs_93216    avgbs_105459    avgbs_109009    avgbs_119506    avgbs_123769    avgbs_123770    avgbs_213077    avgbs_213331    avgbs_213577    avgbs_213586    avgbs_213759    avgbs_213840    avgbs_213983    avgbs_214111    avgbs_214284    avgbs_215019    avgbs_215241    avgbs_215438    avgbs_215655    avgbs_215683    avgbs_215756    avgbs_215997    avgbs_97956    avgbs_215483    avgbs_215801    avgbs_9455    avgbs_10234    avgbs_11367    avgbs_12756    avgbs_18122    avgbs_18448    avgbs_19019    avgbs_19671    avgbs_20245    avgbs_20398    avgbs_21916    avgbs_25056    avgbs_26738    avgbs_29707    avgbs_30744    avgbs_30745    avgbs_30992    avgbs_33663    avgbs_42771    avgbs_45089    avgbs_49742    avgbs_51017    avgbs_51162    avgbs_53009    avgbs_53350    avgbs_60579    avgbs_61993    avgbs_67604    avgbs_71210    avgbs_72881    avgbs_72882    avgbs_73845    avgbs_78573    avgbs_82727    avgbs_83790    avgbs_84831    avgbs_85807    avgbs_87469    avgbs_93277    avgbs_93278    avgbs_99243    avgbs_101269    avgbs_115606    avgbs_116670    avgbs_118267    avgbs_118268    avgbs_118269    avgbs_120532    avgbs_120563    avgbs_122331    avgbs_122332    avgbs_125968    avgbs_213054    avgbs_213272    avgbs_213300    avgbs_213455    avgbs_213466    avgbs_213489    avgbs_213745    avgbs_214063    avgbs_214088    avgbs_214227    avgbs_214230    avgbs_214302    avgbs_214456    avgbs_214660    avgbs_214744    avgbs_215134    avgbs_215177    avgbs_215196    avgbs_215372    avgbs_215781    avgbs_215987    avgbs_216146    avgbs_216184    avgbs_16479    avgbs_16481    avgbs_48144    avgbs_51145    avgbs_54738    avgbs_214161    avgbs_214329    avgbs_214597    avgbs_215668    avgbs_14584    avgbs_14585    avgbs_37416    avgbs_44828    avgbs_46566    avgbs_46567    avgbs_51358    avgbs_52009    avgbs_53846    avgbs_57512    avgbs_58266    avgbs_77925    avgbs_78647    avgbs_81697    avgbs_83791    avgbs_83792    avgbs_84243    avgbs_86659    avgbs_87183    avgbs_92352    avgbs_98182    avgbs_98184    avgbs_6K_91152    avgbs_98834    avgbs_99365    avgbs_101254    avgbs_105644    avgbs_105645    avgbs_107055    avgbs_107774    avgbs_108094    avgbs_109303    avgbs_110147    avgbs_110164    avgbs_110165    avgbs_110684    avgbs_116616    avgbs_116617    avgbs_117027    avgbs_117028    avgbs_118659    avgbs_121306    avgbs_122499    avgbs_213339    avgbs_213391    avgbs_213654    avgbs_213705    avgbs_213908    avgbs_214240    avgbs_214265    avgbs_214877    avgbs_215079    avgbs_215153    avgbs_215170    avgbs_215493    avgbs_215515    avgbs_215728    avgbs_215797    avgbs_215829    avgbs_215995    avgbs_216058    avgbs_216160    avgbs_215434    avgbs_42194    avgbs_81202   
   1C 38 
  GMI_ES15_c7654_479     GMI_ES15_c13526_397     GMI_ES02_c21486_395     GMI_ES17_c7736_565     GMI_ES02_lrc17781_234    
 
  avgbs_112355    avgbs_74295    avgbs_119024    avgbs_125102    avgbs_204563    avgbs_207528    avgbs_119438    avgbs_16075    avgbs_95031    avgbs_11479    avgbs_204689    avgbs_99618    avgbs_109683    avgbs_35775    avgbs_38979    avgbs_38980    avgbs_125557    avgbs_125621    avgbs_27183    avgbs_6016    avgbs_12842    avgbs_12843    avgbs_16509    avgbs_20116    avgbs_20117    avgbs_42418    avgbs_58148    avgbs_61142    avgbs_61469    avgbs_91679    avgbs_111121    avgbs_111823    avgbs_112153    avgbs_120781    avgbs_202163    avgbs_203163    avgbs_203364    avgbs_237915    avgbs_206984    avgbs_72973    avgbs_238744    avgbs_6164    avgbs_10222    avgbs_10223    avgbs_22015    avgbs_40875    avgbs_60171    avgbs_62433    avgbs_65543    avgbs_65797    avgbs_71917    avgbs_78789    avgbs_78790    avgbs_92463    avgbs_93593    avgbs_106898    avgbs_107456    avgbs_108071    avgbs_115834    avgbs_115835    avgbs_118512    avgbs_122330    avgbs_122333    avgbs_122801    avgbs_123600    avgbs_201310    avgbs_202777    avgbs_203501    avgbs_237826    avgbs_204860    avgbs_206293    avgbs_206616    avgbs_207204    avgbs_203829    avgbs_111718    avgbs_122706    avgbs_204345    avgbs_99041    avgbs_108638    avgbs_108639    avgbs_111126    avgbs_126171    avgbs_126172    avgbs_205885    avgbs_38657    avgbs_65808    avgbs_67787    avgbs_67788    avgbs_213942    avgbs_203422    avgbs_74491    avgbs_74492    avgbs_81015    avgbs_6K_60360    avgbs_74607    avgbs_74608    avgbs_201059    avgbs_5947    avgbs_26759    avgbs_113810    avgbs_19565    avgbs_239217    avgbs_34751    avgbs_90148    avgbs_62476    avgbs_65650    avgbs_203021    avgbs_66356    avgbs_85116    avgbs_201951    avgbs_207274    avgbs_207159    avgbs_83129    avgbs_201303    avgbs_208319    avgbs_40147    avgbs_236655    avgbs_242590    avgbs_21816    avgbs_36802    avgbs_72815    avgbs_99631    avgbs_201164    avgbs_92170    avgbs_208316    avgbs_63017    avgbs_102702    avgbs_202253    avgbs_57052    avgbs_78725    avgbs_98618    avgbs_7577    avgbs_220209    avgbs_62547    avgbs_224467    avgbs_43558    avgbs_76965    avgbs_214050    avgbs_200529    avgbs_61317    avgbs_72131    avgbs_25120    avgbs_80197    avgbs_80198    avgbs_215344    avgbs_60741    avgbs_200643    avgbs_111965    avgbs_77408    avgbs_61471    avgbs_214496    avgbs_89227    avgbs_97433    avgbs_97434    avgbs_101251    avgbs_215465    avgbs_215957    avgbs_1427    avgbs_66123   
   1C 39 
  GMI_ES02_c25461_277     GMI_ES17_c10770_123    
 
  avgbs_40874    avgbs_58330    avgbs_58331    avgbs_13486    avgbs_15301    avgbs_55502    avgbs_79217    avgbs_102466    avgbs_113647    avgbs_113649    avgbs_217035    avgbs_203837    avgbs_204880    avgbs_220235    avgbs_34232    avgbs_34233    avgbs_34234    avgbs_34235    avgbs_29989    avgbs_218832    avgbs_207253    avgbs_119329    avgbs_38985    avgbs_50504    avgbs_78134    avgbs_21903    avgbs_21905    avgbs_20817    avgbs_202502    avgbs_216114    avgbs_60202    avgbs_15232    avgbs_42928    avgbs_104491    avgbs_104492    avgbs_201958    avgbs_203186    avgbs_16081    avgbs_16082    avgbs_24065    avgbs_26993    avgbs_38659    avgbs_62307    avgbs_77214    avgbs_77215    avgbs_80057    avgbs_80058    avgbs_110619    avgbs_121629    avgbs_122715    avgbs_122716    avgbs_202669    avgbs_235690    avgbs_203145    avgbs_18091    avgbs_39581    avgbs_39582    avgbs_39583    avgbs_203477    avgbs_106518    avgbs_111932    avgbs_111933    avgbs_55976    avgbs_242014    avgbs_47507    avgbs_125756    avgbs_125757    avgbs_206573    avgbs_21575    avgbs_61964    avgbs_103016    avgbs_97506    avgbs_114342    avgbs_124279    avgbs_65893    avgbs_233029    avgbs_124549    avgbs_208261    avgbs_24294    avgbs_39183    avgbs_39184    avgbs_24265    avgbs_24266    avgbs_241208    avgbs_29185    avgbs_70608    avgbs_207644    avgbs_200957    avgbs_63775    avgbs_220653    avgbs_100889    avgbs_221887    avgbs_100890    avgbs_205283    avgbs_206624    avgbs_106124    avgbs_206259    avgbs_20181    avgbs_47197    avgbs_95002    avgbs_95003    avgbs_108925    avgbs_62063    avgbs_50102    avgbs_15361    avgbs_122707    avgbs_216101    avgbs_11636    avgbs_11635    avgbs_56885    avgbs_215552    avgbs_35953    avgbs_35954    avgbs_78447    avgbs_81194    avgbs_122395    avgbs_213260    avgbs_214283    avgbs_214937    avgbs_215006    avgbs_20182    avgbs_24659    avgbs_43281    avgbs_52603    avgbs_52605    avgbs_66912    avgbs_81197    avgbs_103801    avgbs_214078    avgbs_214824    avgbs_21935    avgbs_31581    avgbs_31582    avgbs_38510    avgbs_55160    avgbs_55162    avgbs_56138    avgbs_60109    avgbs_97186    avgbs_99028    avgbs_101066    avgbs_101067    avgbs_101068    avgbs_110558    avgbs_214511    avgbs_214923    avgbs_820    avgbs_11135    avgbs_9527    avgbs_16130    avgbs_20682    avgbs_22016    avgbs_22017    avgbs_48664    avgbs_63623    avgbs_80901    avgbs_94718    avgbs_113194    avgbs_113195    avgbs_213253    avgbs_213535    avgbs_213755    avgbs_35939    avgbs_8760    avgbs_15539    avgbs_52256    avgbs_52257    avgbs_62064    avgbs_104519    avgbs_110821    avgbs_114291    avgbs_119218    avgbs_215880    avgbs_15632    avgbs_37971    avgbs_49250    avgbs_67526    avgbs_93592    avgbs_117007    avgbs_36441    avgbs_36442   
   1C 40 
  GMI_ES02_c23179_274    
 
  avgbs_25598    avgbs_9749    avgbs_13487    avgbs_99879    avgbs_85333    avgbs_126044    avgbs_217815    avgbs_205004    avgbs_208434    avgbs_91802    avgbs_91803    avgbs_91804    avgbs_5306    avgbs_39197    avgbs_61197    avgbs_18053    avgbs_18054    avgbs_48248    avgbs_104361    avgbs_7985    avgbs_37560    avgbs_37561    avgbs_88048    avgbs_118872    avgbs_123506    avgbs_215195    avgbs_34857    avgbs_205467    avgbs_15072    avgbs_7105    avgbs_29929    avgbs_84759    avgbs_20087    avgbs_34532    avgbs_57656    avgbs_108626    avgbs_108627    avgbs_111518    avgbs_111519    avgbs_124940    avgbs_74857    avgbs_74858    avgbs_5658    avgbs_93635    avgbs_124941    avgbs_126045    avgbs_203217    avgbs_58057    avgbs_101585    avgbs_43680    avgbs_49571    avgbs_34411    avgbs_85317    avgbs_100223    avgbs_113648    avgbs_17118    avgbs_46071    avgbs_48865    avgbs_202222    avgbs_103690    avgbs_118864    avgbs_40863    avgbs_43529    avgbs_94953    avgbs_35210    avgbs_36613    avgbs_104964    avgbs_3256    avgbs_104962    avgbs_118892    avgbs_213876    avgbs_215296    avgbs_213382   
   1C 41 
  GMI_ES02_c6368_605    
 
  avgbs_27960    avgbs_27961    avgbs_27962    avgbs_202778    avgbs_213151    avgbs_40860    avgbs_234868    avgbs_83382    avgbs_125514    avgbs_126243    avgbs_81425    avgbs_81426    avgbs_207543    avgbs_103602    avgbs_103603    avgbs_103604    avgbs_235530    avgbs_238659    avgbs_204786    avgbs_203492    avgbs_206451    avgbs_113915    avgbs_207140    avgbs_12684    avgbs_12685    avgbs_12686   
   1C 42 
  GMI_ES_CC12765_141    
 
  avgbs_105    avgbs_106    avgbs_107    avgbs_2931    avgbs_3647    avgbs_56738    avgbs_56739    avgbs_115659    avgbs_216344    avgbs_217345    avgbs_18295    avgbs_222618    avgbs_29658    avgbs_115958    avgbs_35665    avgbs_75822    avgbs_75823    avgbs_7459    avgbs_69677    avgbs_222572    avgbs_89834    avgbs_95595    avgbs_95596    avgbs_95597    avgbs_110391    avgbs_61198    avgbs_13443    avgbs_13136    avgbs_207008    avgbs_89256    avgbs_206746    avgbs_52118    avgbs_52119    avgbs_14948    avgbs_14949    avgbs_14950    avgbs_25161    avgbs_223581    avgbs_106043    avgbs_200454    avgbs_12880    avgbs_12881    avgbs_70576    avgbs_89214    avgbs_89215    avgbs_200478    avgbs_83799    avgbs_84526    avgbs_99047    avgbs_39590    avgbs_39591    avgbs_39592    avgbs_92257    avgbs_92258    avgbs_200642    avgbs_59742    avgbs_60688    avgbs_224450    avgbs_115112    avgbs_89451    avgbs_70893    avgbs_70941    avgbs_11754   
   1C 43 
  GMI_ES15_c1370_537    
 
  avgbs_63098    avgbs_214559    avgbs_2787    avgbs_21684    avgbs_83383    avgbs_84841    avgbs_217489    avgbs_66872    avgbs_82174    avgbs_82175    avgbs_99340    avgbs_122900    avgbs_219791    avgbs_216929    avgbs_27769    avgbs_82238    avgbs_102026    avgbs_91912    avgbs_91913    avgbs_5790    avgbs_5791    avgbs_83728    avgbs_83729    avgbs_21904    avgbs_21907    avgbs_38151    avgbs_92891    avgbs_54768    avgbs_205243    avgbs_106136    avgbs_87351    avgbs_87352    avgbs_25492    avgbs_52174    avgbs_240542    avgbs_102402    avgbs_102403    avgbs_102404   
   1C 44 
  GMI_ES15_c6229_566     GMI_ES15_c5216_593    
 
  avgbs_3482    avgbs_48612    avgbs_74972    avgbs_96263    avgbs_106254    avgbs_115048    avgbs_200214    avgbs_218041    avgbs_219589    avgbs_220504    avgbs_220824    avgbs_222856    avgbs_112896    avgbs_6K_29011    avgbs_39198    avgbs_221040    avgbs_18914    avgbs_117709    avgbs_117710    avgbs_117552    avgbs_1962    avgbs_213064    avgbs_102739    avgbs_57778    avgbs_63271    avgbs_86454    avgbs_44160   
   1C 45 
  GMI_DS_CC9627_146    
 
  avgbs_51138    avgbs_100108    avgbs_100109    avgbs_100110    avgbs_1406    avgbs_6K_2268    avgbs_3438    avgbs_8645    avgbs_14762    avgbs_16058    avgbs_18993    avgbs_19510    avgbs_28826    avgbs_35197    avgbs_42861    avgbs_42862    avgbs_46776    avgbs_49611    avgbs_54994    avgbs_60175    avgbs_68724    avgbs_73009    avgbs_90518    avgbs_92306    avgbs_93306    avgbs_93307    avgbs_97801    avgbs_98931    avgbs_105798    avgbs_114007    avgbs_114921    avgbs_216804    avgbs_213398    avgbs_217178    avgbs_217334    avgbs_217773    avgbs_217812    avgbs_205031    avgbs_220815    avgbs_221176    avgbs_221225    avgbs_221886    avgbs_222376    avgbs_222626    avgbs_222788    avgbs_223155    avgbs_87600    avgbs_23550    avgbs_23551    avgbs_74450    avgbs_110752    avgbs_26398    avgbs_117332    avgbs_70894    avgbs_220036    avgbs_48100    avgbs_76989    avgbs_74448    avgbs_74449    avgbs_12536    avgbs_40794    avgbs_229755    avgbs_58724    avgbs_230534    avgbs_19460    avgbs_19461    avgbs_44941    avgbs_17235    avgbs_17236    avgbs_17237    avgbs_73452    avgbs_47993    avgbs_47994    avgbs_236211    avgbs_236942   
   1C 46 
  GMI_ES_CC12738_121     GMI_ES_CC10812_102     GMI_ES17_c1918_529    
 
  avgbs_8679    avgbs_25324    avgbs_25325    avgbs_37690    avgbs_63202    avgbs_99600    avgbs_99601    avgbs_107825    avgbs_107856    avgbs_119410    avgbs_201717    avgbs_217159    avgbs_218012    avgbs_203476    avgbs_219191    avgbs_222381    avgbs_222694    avgbs_9621    avgbs_12719    avgbs_42502    avgbs_45859    avgbs_57813    avgbs_74329    avgbs_75454    avgbs_79849    avgbs_87288    avgbs_88907    avgbs_94636    avgbs_102380    avgbs_113287    avgbs_203323    avgbs_218611    avgbs_203934    avgbs_205109    avgbs_205447    avgbs_221205    avgbs_222153    avgbs_16304    avgbs_37135    avgbs_57498    avgbs_64043    avgbs_202131    avgbs_219247    avgbs_219639    avgbs_116137    avgbs_44601    avgbs_64433    avgbs_98298    avgbs_219666    avgbs_81537    avgbs_71932    avgbs_220093    avgbs_20012    avgbs_83939    avgbs_217392    avgbs_210278    avgbs_87747    avgbs_21032    avgbs_17778    avgbs_17487    avgbs_40551    avgbs_105075    avgbs_125210    avgbs_59185    avgbs_81384    avgbs_81385    avgbs_237712    avgbs_111194    avgbs_111834    avgbs_92905    avgbs_104576    avgbs_239007    avgbs_125177    avgbs_6928    avgbs_6929    avgbs_6930    avgbs_32218    avgbs_63817    avgbs_79373    avgbs_79374    avgbs_122692    avgbs_28271    avgbs_30810    avgbs_209566    avgbs_76458    avgbs_76459    avgbs_95236    avgbs_112042    avgbs_220746    avgbs_68521    avgbs_74523    avgbs_74524    avgbs_74525    avgbs_86148    avgbs_205207    avgbs_79714    avgbs_116233    avgbs_201609    avgbs_236881    avgbs_94439    avgbs_114900    avgbs_47925    avgbs_201172    avgbs_119892    avgbs_243751    avgbs_54296    avgbs_6690    avgbs_106044    avgbs_34340    avgbs_82787    avgbs_82788   
   1C 47 
  GMI_DS_A3_242_303     GMI_ES01_c3447_952    
 
  avgbs_2185    avgbs_2186    avgbs_642    avgbs_643    avgbs_8798    avgbs_21828    avgbs_57425    avgbs_65866    avgbs_84947    avgbs_84948    avgbs_108338    avgbs_110290    avgbs_217034    avgbs_204193    avgbs_205535    avgbs_222360    avgbs_215876    avgbs_223176    avgbs_5276    avgbs_8815    avgbs_9153    avgbs_11688    avgbs_18036    avgbs_18623    avgbs_19201    avgbs_21348    avgbs_35506    avgbs_56119    avgbs_64347    avgbs_72951    avgbs_74204    avgbs_74205    avgbs_75497    avgbs_75641    avgbs_80742    avgbs_81416    avgbs_98027    avgbs_107953    avgbs_108959    avgbs_112159    avgbs_124243    avgbs_125421    avgbs_125422    avgbs_126143    avgbs_216504    avgbs_201564    avgbs_217423    avgbs_219152    avgbs_204973    avgbs_220654    avgbs_205234    avgbs_221711    avgbs_206658    avgbs_208447    avgbs_438    avgbs_439    avgbs_27003    avgbs_43336    avgbs_43337    avgbs_43338    avgbs_90076    avgbs_120553    avgbs_222878    avgbs_33430    avgbs_33431    avgbs_43873    avgbs_55325    avgbs_55326    avgbs_73861    avgbs_84747    avgbs_203271    avgbs_6K_11503    avgbs_19876    avgbs_41398    avgbs_80364    avgbs_220602    avgbs_55512    avgbs_219131    avgbs_70127    avgbs_14647    avgbs_42400    avgbs_111165    avgbs_15524    avgbs_15525    avgbs_103984    avgbs_23952    avgbs_43719    avgbs_217572    avgbs_12750    avgbs_44817    avgbs_44818    avgbs_44819    avgbs_228925    avgbs_85437    avgbs_124244    avgbs_228681    avgbs_200930    avgbs_18037    avgbs_15380    avgbs_69831    avgbs_69832    avgbs_101794    avgbs_101795    avgbs_116320    avgbs_118009    avgbs_26950    avgbs_15565    avgbs_34702    avgbs_34703    avgbs_28724    avgbs_85538    avgbs_109969    avgbs_239864    avgbs_26192    avgbs_107984    avgbs_244025    avgbs_74161    avgbs_238824    avgbs_57262    avgbs_225276    avgbs_20043    avgbs_13325    avgbs_9447    avgbs_34341    avgbs_88097   
   1C 48 
 
 
  avgbs_34430    avgbs_123254    avgbs_123255    avgbs_33991    avgbs_200437    avgbs_81874    avgbs_6K_109558    avgbs_119752    avgbs_28217    avgbs_34551    avgbs_38148    avgbs_49506    avgbs_59688    avgbs_84998    avgbs_102445    avgbs_104663    avgbs_205152    avgbs_85706    avgbs_87332    avgbs_1737    avgbs_104648    avgbs_104649    avgbs_242193    avgbs_1535    avgbs_26021    avgbs_55747    avgbs_6K_94269    avgbs_121072    avgbs_28943    avgbs_54335    avgbs_63868    avgbs_237432    avgbs_10351    avgbs_4102    avgbs_86072    avgbs_225025    avgbs_244051    avgbs_125438    avgbs_223135    avgbs_52416    avgbs_52417    avgbs_241956    avgbs_236334    avgbs_6K_47245    avgbs_37669    avgbs_28873    avgbs_209583    avgbs_203890    avgbs_18060    avgbs_51182    avgbs_223845    avgbs_214232    avgbs_3609    avgbs_78748    avgbs_224227    avgbs_224248    avgbs_5977    avgbs_83110    avgbs_70932    avgbs_70934    avgbs_79968    avgbs_79969    avgbs_224676   
   1C 49 
  GMI_ES02_c12621_204     GMI_ES02_c4091_462    
  GMI_ES02_c19578_292    GMI_ES17_c3051_143   
  avgbs_216626    avgbs_21059    avgbs_63350    avgbs_95139    avgbs_120742    avgbs_122155    avgbs_122156    avgbs_217441    avgbs_222891    avgbs_34549    avgbs_45996    avgbs_46003    avgbs_48974    avgbs_48975    avgbs_48976    avgbs_49601    avgbs_49602    avgbs_51135    avgbs_53609    avgbs_58640    avgbs_73622    avgbs_73729    avgbs_95278    avgbs_97123    avgbs_120950    avgbs_218067    avgbs_200292    avgbs_214309    avgbs_214411    avgbs_222678    avgbs_222897    avgbs_7861    avgbs_9294    avgbs_12334    avgbs_13142    avgbs_35507    avgbs_58202    avgbs_74005    avgbs_90290    avgbs_93205    avgbs_98665    avgbs_98666    avgbs_102257    avgbs_102258    avgbs_114119    avgbs_114120    avgbs_114121    avgbs_216647    avgbs_201764    avgbs_203038    avgbs_218763    avgbs_29574    avgbs_97110    avgbs_97111    avgbs_205683    avgbs_890    avgbs_87658    avgbs_220715    avgbs_223009    avgbs_82090    avgbs_82091    avgbs_1824    avgbs_51720    avgbs_117554    avgbs_117555    avgbs_83597    avgbs_101786    avgbs_28067    avgbs_12552    avgbs_27004    avgbs_5425    avgbs_18191    avgbs_22568    avgbs_22569    avgbs_31428    avgbs_71171    avgbs_97451    avgbs_122935    avgbs_122936    avgbs_210456    avgbs_224673    avgbs_204410    avgbs_232687    avgbs_83786    avgbs_122367    avgbs_107935    avgbs_201116    avgbs_86469    avgbs_84780    avgbs_84781    avgbs_6K_21750    avgbs_57766    avgbs_237563    avgbs_117556    avgbs_36388    avgbs_36389    avgbs_77359    avgbs_239088    avgbs_609    avgbs_38247    avgbs_52743    avgbs_122697    avgbs_71169    avgbs_71170    avgbs_206527    avgbs_87856    avgbs_44241    avgbs_92007    avgbs_72720    avgbs_239654    avgbs_121355    avgbs_223878    avgbs_79406    avgbs_61820    avgbs_118005    avgbs_69517    avgbs_223360    avgbs_33449    avgbs_19016    avgbs_35335    avgbs_205306    avgbs_225659    avgbs_64879    avgbs_99565    avgbs_105329    avgbs_16228   
   1C 50 
 
  GMI_DS_CC7541_122   
  avgbs_75743    avgbs_204640    avgbs_4346    avgbs_32986    avgbs_86071    avgbs_497    avgbs_25015    avgbs_46950    avgbs_122120    avgbs_200976    avgbs_202582    avgbs_219600    avgbs_113118    avgbs_81717    avgbs_220668    avgbs_2275    avgbs_120901    avgbs_120902    avgbs_120903    avgbs_108120    avgbs_1521    avgbs_1522    avgbs_110092    avgbs_10569    avgbs_231764    avgbs_14551    avgbs_63349    avgbs_52210    avgbs_52211    avgbs_74418    avgbs_74419    avgbs_117557    avgbs_117558    avgbs_1673    avgbs_20691    avgbs_20692    avgbs_95886    avgbs_124462    avgbs_126021    avgbs_126022    avgbs_238666    avgbs_80426    avgbs_80427    avgbs_44662    avgbs_125103    avgbs_203576    avgbs_34241    avgbs_59687    avgbs_78615    avgbs_202628   
   1C 51 
  GMI_ES02_lrc13788_346    
 
  avgbs_62483    avgbs_118417    avgbs_215980    avgbs_83384    avgbs_220756    avgbs_50054    avgbs_81148    avgbs_67141    avgbs_71561    avgbs_25897    avgbs_40525    avgbs_228930    avgbs_83538    avgbs_240947    avgbs_70292    avgbs_204379    avgbs_53390    avgbs_95047    avgbs_95048    avgbs_73157    avgbs_122693    avgbs_85235    avgbs_236867    avgbs_224573    avgbs_24066    avgbs_9281    avgbs_35943    avgbs_18684    avgbs_77614   
   1C 52 
  GMI_ES01_c9472_428    
 
  avgbs_85043    avgbs_69339    avgbs_228854    avgbs_228919    avgbs_74343    avgbs_58099    avgbs_58100    avgbs_13610    avgbs_13611    avgbs_117522    avgbs_3983    avgbs_241449    avgbs_52778    avgbs_236521    avgbs_17658    avgbs_225831    avgbs_50309    avgbs_224669   
   1C 53 
 
 
  avgbs_34705    avgbs_68854    avgbs_219886    avgbs_60192    avgbs_53518    avgbs_214308    avgbs_63225    avgbs_75424    avgbs_223617    avgbs_51198    avgbs_51199    avgbs_51200    avgbs_17722    avgbs_17723    avgbs_17724    avgbs_121636    avgbs_121637    avgbs_121725    avgbs_121726    avgbs_38001    avgbs_53964    avgbs_120255    avgbs_101591   
   1C 54 
 
 
  avgbs_11228    avgbs_201723    avgbs_117385    avgbs_221488    avgbs_82075    avgbs_82076    avgbs_119827    avgbs_21603    avgbs_100708    avgbs_72950    avgbs_101865    avgbs_232183    avgbs_234640    avgbs_223707    avgbs_55566   
   1C 55 
 
 
  avgbs_219640    avgbs_15275    avgbs_23078    avgbs_23079    avgbs_49220    avgbs_58866    avgbs_73088    avgbs_77465    avgbs_100653    avgbs_217770    avgbs_219332    avgbs_204519    avgbs_221302    avgbs_221943    avgbs_222890    avgbs_82225    avgbs_72969    avgbs_99266    avgbs_70019    avgbs_114975    avgbs_229015    avgbs_231171    avgbs_76219    avgbs_56489    avgbs_90710    avgbs_12022    avgbs_13700    avgbs_19202    avgbs_19203    avgbs_48670    avgbs_48671    avgbs_111523    avgbs_229136    avgbs_232361    avgbs_25227    avgbs_25442    avgbs_107952    avgbs_100709    avgbs_96780    avgbs_231227    avgbs_229505    avgbs_87105    avgbs_238035    avgbs_34003    avgbs_223809    avgbs_224167    avgbs_86463   
   1C 56 
  GMI_ES17_c3217_588     GMI_ES_CC12297_189    
 
  avgbs_12870    avgbs_12931    avgbs_107421    avgbs_87436    avgbs_87437    avgbs_218799    avgbs_26417    avgbs_85518    avgbs_26214    avgbs_45147    avgbs_45148    avgbs_45149    avgbs_45150    avgbs_51313    avgbs_97313    avgbs_231006    avgbs_231879    avgbs_909    avgbs_3636    avgbs_3637    avgbs_29750    avgbs_93912    avgbs_108240    avgbs_228782    avgbs_232702    avgbs_9330    avgbs_9331    avgbs_23380    avgbs_23381    avgbs_30372    avgbs_106299    avgbs_229390    avgbs_56228    avgbs_230431    avgbs_231406    avgbs_202703    avgbs_24913    avgbs_87985    avgbs_87986    avgbs_14043    avgbs_14044    avgbs_22094    avgbs_89922    avgbs_48004    avgbs_112307    avgbs_84156    avgbs_107715    avgbs_107717    avgbs_93245   
   1C 57 
 
 
  avgbs_85098    avgbs_85099    avgbs_105743    avgbs_6539    avgbs_28872   
   1C 58 
 
 
  avgbs_33607    avgbs_28158    avgbs_28159    avgbs_28160    avgbs_58232    avgbs_95025    avgbs_221486    avgbs_33146    avgbs_99833    avgbs_242404    avgbs_118719    avgbs_108266   
   1C 59 
 
 
  avgbs_221288    avgbs_218458    avgbs_24748    avgbs_72130    avgbs_39918    avgbs_93568    avgbs_93569    avgbs_216505    avgbs_12799    avgbs_12800    avgbs_40975    avgbs_118044    avgbs_223593    avgbs_26989    avgbs_26990    avgbs_34102   
   1C 60 
 
 
  avgbs_7999    avgbs_87696    avgbs_22554    avgbs_22555    avgbs_40148    avgbs_79295    avgbs_125143    avgbs_125144    avgbs_219487    avgbs_1515    avgbs_35357    avgbs_35358    avgbs_42024    avgbs_49851    avgbs_81373    avgbs_86861    avgbs_86862    avgbs_90243    avgbs_109620    avgbs_124709    avgbs_125595    avgbs_229993    avgbs_230592    avgbs_232746    avgbs_232798    avgbs_71320    avgbs_32433    avgbs_32434    avgbs_6902    avgbs_233617    avgbs_82750    avgbs_82751   
   1C 61 
  GMI_ES17_c7997_871    
 
  avgbs_57284    avgbs_110738    avgbs_30029    avgbs_48370    avgbs_96752    avgbs_96916    avgbs_217878    avgbs_219031    avgbs_219242    avgbs_221629    avgbs_11147    avgbs_23931    avgbs_23932    avgbs_47164    avgbs_2349    avgbs_28116    avgbs_90244    avgbs_232790    avgbs_114325    avgbs_228519    avgbs_35356    avgbs_59580    avgbs_1136    avgbs_25625    avgbs_104684    avgbs_244608    avgbs_62801    avgbs_40974    avgbs_52445    avgbs_52446    avgbs_224301    avgbs_243996    avgbs_42762    avgbs_42763    avgbs_243739    avgbs_63102    avgbs_63103    avgbs_10958    avgbs_47007    avgbs_98787    avgbs_103326    avgbs_103327    avgbs_104198    avgbs_239917    avgbs_48957    avgbs_48958    avgbs_74169    avgbs_241374    avgbs_233841   
   1C 62 
 
 
  avgbs_1107    avgbs_22452    avgbs_22453    avgbs_38726    avgbs_56768    avgbs_219210    avgbs_220347    avgbs_36367    avgbs_73936    avgbs_78542    avgbs_210994    avgbs_220490    avgbs_42377    avgbs_15988    avgbs_213420    avgbs_108942    avgbs_222292    avgbs_236366   
   1C 63 
  GMI_ES02_lrc13446_328     GMI_ES17_c4700_516    
 
  avgbs_202719    avgbs_221614    avgbs_96094    avgbs_90074    avgbs_117423    avgbs_222702    avgbs_50928    avgbs_63598    avgbs_1208    avgbs_1209    avgbs_108759    avgbs_5012    avgbs_5013    avgbs_205418    avgbs_111688    avgbs_63863    avgbs_63864    avgbs_78261    avgbs_241826    avgbs_242101   
   1C 64 
 
 
  avgbs_13345    avgbs_55795    avgbs_14430    avgbs_14477    avgbs_36150    avgbs_230649    avgbs_100280    avgbs_48953    avgbs_49443    avgbs_203701    avgbs_49442    avgbs_241221   
   1C 65 
  GMI_ES01_lrc11151_273    
 
  avgbs_70865    avgbs_63521    avgbs_70302   
   1C 66 
  GMI_ES_CC4633_339     GMI_ES15_c12794_114     GMI_ES17_c2917_533    
 
  avgbs_58864    avgbs_7925    avgbs_53848    avgbs_218216    avgbs_121984    avgbs_22737    avgbs_218689    avgbs_95161    avgbs_35502    avgbs_108512    avgbs_53847    avgbs_66484    avgbs_122879    avgbs_19998    avgbs_19999    avgbs_108649    avgbs_209476    avgbs_40426    avgbs_40427    avgbs_108953    avgbs_15775    avgbs_15776    avgbs_15777    avgbs_15792    avgbs_15793    avgbs_15794    avgbs_82937    avgbs_82938    avgbs_82939    avgbs_103749    avgbs_225397    avgbs_66076    avgbs_106353    avgbs_108794    avgbs_223356    avgbs_201794    avgbs_223501    avgbs_224797    avgbs_5782    avgbs_84356    avgbs_224204    avgbs_224156   
   1C 67 
  GMI_ES15_c7125_354    
 
  avgbs_116240    avgbs_116243    avgbs_119063    avgbs_119064    avgbs_218855    avgbs_61460    avgbs_23606    avgbs_55139    avgbs_55140    avgbs_59824    avgbs_228916    avgbs_228996    avgbs_230401    avgbs_23604    avgbs_75208    avgbs_108103    avgbs_120824    avgbs_231083    avgbs_232511    avgbs_86013    avgbs_76441    avgbs_76444    avgbs_82470    avgbs_6318    avgbs_6319    avgbs_71537   
   1C 68 
  GMI_ES17_c2826_360     GMI_ES17_c20752_1084     GMI_ES02_c11747_563    
 
  avgbs_9697    avgbs_9698    avgbs_65771    avgbs_86069    avgbs_86070    avgbs_219735    avgbs_104028    avgbs_120817    avgbs_120819    avgbs_4252    avgbs_5493    avgbs_7893    avgbs_62354    avgbs_72654    avgbs_73045    avgbs_73046    avgbs_73047    avgbs_88984    avgbs_108177    avgbs_216421    avgbs_216513    avgbs_13264    avgbs_216024    avgbs_223102    avgbs_1520    avgbs_9320    avgbs_9321    avgbs_6K_21923    avgbs_74182    avgbs_86324    avgbs_92154    avgbs_109589    avgbs_200039    avgbs_200239    avgbs_218714    avgbs_200495    avgbs_200936    avgbs_21187    avgbs_21196    avgbs_57118    avgbs_19636    avgbs_65575    avgbs_88477    avgbs_94838    avgbs_24878    avgbs_40559    avgbs_213976    avgbs_12238    avgbs_79794    avgbs_33299    avgbs_74181    avgbs_92448    avgbs_114680    avgbs_114681    avgbs_114682    avgbs_224171    avgbs_23241    avgbs_63251    avgbs_200912    avgbs_213843    avgbs_111602    avgbs_206910    avgbs_58663    avgbs_58664    avgbs_87128    avgbs_4466    avgbs_4467    avgbs_232229    avgbs_44737    avgbs_3081    avgbs_14700    avgbs_14701    avgbs_79044    avgbs_125721    avgbs_204867    avgbs_40698    avgbs_56356    avgbs_73061    avgbs_80009    avgbs_106686    avgbs_106687    avgbs_7875    avgbs_87581    avgbs_209457    avgbs_9132    avgbs_9133    avgbs_15753    avgbs_15754    avgbs_22117    avgbs_22118    avgbs_24075    avgbs_71331    avgbs_71332    avgbs_85681    avgbs_6K_79511    avgbs_98852    avgbs_105725    avgbs_111647    avgbs_121038    avgbs_233574    avgbs_239507    avgbs_212035    avgbs_85423    avgbs_85425    avgbs_10571    avgbs_18166    avgbs_18772    avgbs_21185    avgbs_21186    avgbs_30617    avgbs_38826    avgbs_44397    avgbs_44745    avgbs_71918    avgbs_85682    avgbs_96145    avgbs_108948    avgbs_115421    avgbs_115757    avgbs_120430    avgbs_120551    avgbs_120552    avgbs_120820    avgbs_210740    avgbs_204888    avgbs_215311    avgbs_45398    avgbs_61439    avgbs_94917    avgbs_107854    avgbs_119857    avgbs_221825    avgbs_6127    avgbs_47348    avgbs_48448    avgbs_48449    avgbs_61896    avgbs_124261    avgbs_200310    avgbs_775    avgbs_15987    avgbs_18776    avgbs_37052    avgbs_64161    avgbs_99778    avgbs_99779    avgbs_238154    avgbs_76629    avgbs_100107    avgbs_18622    avgbs_74290    avgbs_238837    avgbs_24837    avgbs_57438    avgbs_68624    avgbs_40826    avgbs_243079    avgbs_25948    avgbs_44744    avgbs_26329    avgbs_26330    avgbs_26331    avgbs_55212    avgbs_55213    avgbs_34735    avgbs_73379    avgbs_242017    avgbs_240535    avgbs_211232    avgbs_13905    avgbs_30215    avgbs_30216   
   1C 69 
  GMI_ES15_c8975_729     GMI_ES02_c13712_383     GMI_ES_CC15404_67     GMI_ES15_c5289_317     GMI_ES17_c15844_381     GMI_ES01_c8777_202    
 
  avgbs_208880    avgbs_3621    avgbs_13472    avgbs_59275    avgbs_80428    avgbs_93386    avgbs_93387    avgbs_93388    avgbs_101888    avgbs_108663    avgbs_216498    avgbs_200095    avgbs_218250    avgbs_219353    avgbs_73319    avgbs_83516    avgbs_83517    avgbs_44255    avgbs_117662    avgbs_200620    avgbs_19207    avgbs_42869    avgbs_16663    avgbs_66090    avgbs_121886    avgbs_212871    avgbs_12968    avgbs_7892    avgbs_121155    avgbs_229821    avgbs_216061    avgbs_29248    avgbs_6K_66317    avgbs_81240    avgbs_81241    avgbs_228932    avgbs_232660    avgbs_106450    avgbs_14073    avgbs_14074    avgbs_33033    avgbs_55576    avgbs_230416    avgbs_72120    avgbs_71559    avgbs_109575    avgbs_7154    avgbs_16666    avgbs_106451    avgbs_102651    avgbs_102652    avgbs_15502    avgbs_114172    avgbs_114173    avgbs_81987    avgbs_848    avgbs_19274    avgbs_19275    avgbs_20640    avgbs_23714    avgbs_23715    avgbs_96748    avgbs_200660    avgbs_200687    avgbs_200884    avgbs_18689    avgbs_7801    avgbs_42378    avgbs_213162    avgbs_215659    avgbs_28066    avgbs_36135    avgbs_36136    avgbs_36137    avgbs_113712    avgbs_50529    avgbs_94443    avgbs_215373    avgbs_25867    avgbs_97997    avgbs_104711    avgbs_105513    avgbs_2271    avgbs_2272    avgbs_77466    avgbs_77469    avgbs_79298    avgbs_16840    avgbs_16841    avgbs_16842    avgbs_86779    avgbs_120312    avgbs_33837    avgbs_33839    avgbs_18061    avgbs_213463    avgbs_216141   
   1C 70 
  GMI_ES_CC16289_111     GMI_ES02_c19974_185     GMI_ES15_c276_367    
 
  avgbs_7466    avgbs_7467    avgbs_9891    avgbs_53903    avgbs_97314    avgbs_97315    avgbs_99942    avgbs_103972    avgbs_116353    avgbs_216533    avgbs_217753    avgbs_200759    avgbs_3049    avgbs_25107    avgbs_59269    avgbs_70596    avgbs_70597    avgbs_101260    avgbs_216271    avgbs_217616    avgbs_17470    avgbs_31649    avgbs_31650    avgbs_54289    avgbs_70908    avgbs_77523    avgbs_77524    avgbs_101261    avgbs_123568    avgbs_217436    avgbs_9744    avgbs_9747    avgbs_18899    avgbs_19000    avgbs_43011    avgbs_46100    avgbs_46101    avgbs_111503    avgbs_217513    avgbs_124612    avgbs_11778    avgbs_11779    avgbs_12124    avgbs_18887    avgbs_32530    avgbs_32531    avgbs_46098    avgbs_46099    avgbs_72843    avgbs_89248    avgbs_220706    avgbs_1823    avgbs_220382    avgbs_1459    avgbs_229269    avgbs_232176    avgbs_6K_4876    avgbs_90114    avgbs_230078    avgbs_232755    avgbs_2255    avgbs_17386    avgbs_200114    avgbs_90113   
   1C 71 
  GMI_ES15_c5908_278     GMI_ES01_c22545_242     GMI_DS_CC9424_119    
 
  avgbs_90505    avgbs_99801    avgbs_99802    avgbs_200632    avgbs_17403    avgbs_99798    avgbs_99799    avgbs_203125    avgbs_17175    avgbs_17176    avgbs_38149    avgbs_75862    avgbs_116857    avgbs_116858    avgbs_7393    avgbs_7394    avgbs_111485    avgbs_110970   
   1C 72 
  GMI_ES17_c1315_660    
 
  avgbs_119202    avgbs_222819    avgbs_18900    avgbs_74469    avgbs_229307    avgbs_20169    avgbs_84270    avgbs_229275    avgbs_229347    avgbs_231434    avgbs_86995    avgbs_5071    avgbs_213140    avgbs_224781    avgbs_224309    avgbs_224920    avgbs_224016    avgbs_224438    avgbs_224231   
   1C 73 
  GMI_ES01_c25270_71     GMI_ES01_c17183_318    
 
  avgbs_35612    avgbs_35613   
   1C 74 
 
 
  avgbs_243110    avgbs_212889    avgbs_223711    avgbs_59808    avgbs_59809    avgbs_225349    avgbs_225652   
   1C 75 
  GMI_ES15_c6153_392    
 
  avgbs_19   
   1C 76 
 
 
  avgbs_98647    avgbs_221767   
   1C 77 
 
 
  avgbs_224281   
   1C 79 
 
 
  avgbs_38905    avgbs_223976   
   1C 86 
 
 
  avgbs_216890   
 
 
   Chromosome 2C    Chr   Pos   Framework   Placed SNP  Placed GBS  
   2C -8 
 
 
  avgbs_107206   
   2C -5 
 
 
  avgbs_119799   
   2C -3 
 
 
  avgbs_56558    avgbs_88545    avgbs_225076    avgbs_68674    avgbs_223417   
   2C -2 
 
 
  avgbs_12999    avgbs_201788    avgbs_224979   
   2C -1 
 
 
  avgbs_124283   
   2C 0 
  GMI_ES_CC4334_304    
 
  avgbs_51888    avgbs_218047    avgbs_218937    avgbs_68673    avgbs_219880    avgbs_12729   
   2C 1 
 
 
  avgbs_7862   
   2C 2 
 
 
  avgbs_6K_81139   
   2C 3 
  GMI_ES15_c7706_583    
 
  avgbs_85300    avgbs_205189    avgbs_20238    avgbs_20239    avgbs_7386    avgbs_7387    avgbs_115109    avgbs_115110    avgbs_115715    avgbs_115716    avgbs_70126   
   2C 4 
 
 
  avgbs_19638    avgbs_45910    avgbs_29207    avgbs_49129    avgbs_49130    avgbs_111701    avgbs_203539    avgbs_222427    avgbs_30966    avgbs_213769    avgbs_60526    avgbs_225272    avgbs_82982    avgbs_66797    avgbs_15070    avgbs_15071    avgbs_10284    avgbs_86457    avgbs_223406    avgbs_224000    avgbs_215145    avgbs_7900   
   2C 5 
  GMI_ES01_c13403_102    
 
  avgbs_113681    avgbs_203675    avgbs_222525    avgbs_101418    avgbs_221916    avgbs_214268    avgbs_214526    avgbs_109216    avgbs_111952    avgbs_22216    avgbs_124064    avgbs_124065    avgbs_124066    avgbs_244001    avgbs_241494    avgbs_91528    avgbs_14    avgbs_19866    avgbs_103296    avgbs_219249    avgbs_101594    avgbs_225357    avgbs_17637    avgbs_114363    avgbs_1440    avgbs_78668    avgbs_111212    avgbs_125677    avgbs_4983    avgbs_7898    avgbs_18478    avgbs_33398    avgbs_33399    avgbs_6K_31405    avgbs_65751    avgbs_78852    avgbs_104550    avgbs_204858    avgbs_244627    avgbs_108999    avgbs_72074    avgbs_235579    avgbs_91680    avgbs_233581    avgbs_239343    avgbs_212480    avgbs_34543    avgbs_34544    avgbs_34545    avgbs_236460    avgbs_39899    avgbs_40705    avgbs_44220    avgbs_207647    avgbs_76521    avgbs_99843    avgbs_99844    avgbs_208173    avgbs_208953    avgbs_109285    avgbs_109286    avgbs_119002    avgbs_219019    avgbs_28676    avgbs_61473    avgbs_121559    avgbs_121569    avgbs_235782    avgbs_108900    avgbs_93212    avgbs_117068    avgbs_118669    avgbs_204457    avgbs_60900    avgbs_72559    avgbs_112569    avgbs_234914    avgbs_212541    avgbs_36830    avgbs_6K_70341    avgbs_98882    avgbs_241119    avgbs_244410    avgbs_40197    avgbs_57684    avgbs_238984    avgbs_238864    avgbs_26770    avgbs_72153    avgbs_98576    avgbs_98577    avgbs_95331   
   2C 6 
  GMI_ES15_c690_324    
 
  avgbs_52284    avgbs_70945    avgbs_48838    avgbs_116573    avgbs_215928    avgbs_7624    avgbs_201463    avgbs_21576    avgbs_44800    avgbs_42405    avgbs_224111    avgbs_65108    avgbs_116572   
   2C 8 
 
 
  avgbs_108901    avgbs_200609   
   2C 9 
 
 
  avgbs_86707    avgbs_73886    avgbs_225393   
   2C 10 
 
 
  avgbs_57533   
   2C 11 
 
 
  avgbs_203121   
   2C 12 
 
 
  avgbs_21577    avgbs_214943   
   2C 13 
 
 
  avgbs_80348    avgbs_8024   
   2C 14 
 
 
  avgbs_202772    avgbs_223859   
   2C 15 
 
 
  avgbs_6820   
   2C 16 
 
 
  avgbs_52212   
   2C 18 
 
 
  avgbs_38006   
   2C 20 
 
 
  avgbs_231623    avgbs_62975    avgbs_62976   
   2C 21 
 
 
  avgbs_34407   
   2C 22 
  GMI_ES_CC12360_189     GMI_DS_CC9187_141    
 
  avgbs_19204    avgbs_92091    avgbs_106692    avgbs_33023    avgbs_59811    avgbs_230595   
   2C 27 
  GMI_ES17_c3418_95    
 
 
   2C 28 
  GMI_ES17_c3291_859    
 
  avgbs_48605    avgbs_21882    avgbs_34483    avgbs_34484    avgbs_219818    avgbs_10862    avgbs_12941    avgbs_26843    avgbs_44715    avgbs_44716    avgbs_67467    avgbs_72162    avgbs_82488    avgbs_95635    avgbs_99280    avgbs_99281    avgbs_124129    avgbs_217004    avgbs_204253    avgbs_220702    avgbs_6K_2633    avgbs_22068    avgbs_35297    avgbs_124786    avgbs_124788    avgbs_223108    avgbs_48070    avgbs_52450    avgbs_52451    avgbs_109359    avgbs_9260    avgbs_219792    avgbs_106493    avgbs_1226    avgbs_5181    avgbs_10209    avgbs_10210    avgbs_11101    avgbs_24451    avgbs_32387    avgbs_68010    avgbs_68077    avgbs_117257    avgbs_201004    avgbs_228698    avgbs_228850    avgbs_229047    avgbs_229112    avgbs_232015    avgbs_43610    avgbs_233825    avgbs_203210    avgbs_233126    avgbs_216551    avgbs_90289    avgbs_237127    avgbs_89488    avgbs_123214    avgbs_114618    avgbs_100126    avgbs_11293    avgbs_217582    avgbs_39045    avgbs_42061    avgbs_124533    avgbs_235694    avgbs_115469    avgbs_207362    avgbs_6205    avgbs_36428    avgbs_36429    avgbs_36430    avgbs_20151    avgbs_67369    avgbs_67370    avgbs_92211    avgbs_205603    avgbs_241676    avgbs_17101    avgbs_26365    avgbs_35474    avgbs_113526    avgbs_113527    avgbs_125935    avgbs_27307    avgbs_77478    avgbs_237840    avgbs_46102    avgbs_16246    avgbs_73277    avgbs_200494    avgbs_118277    avgbs_240686    avgbs_210121    avgbs_74641    avgbs_201637   
   2C 30 
 
 
  avgbs_51160    avgbs_205354    avgbs_238366    avgbs_110273    avgbs_124795   
   2C 31 
  GMI_ES02_c3181_680    
 
  avgbs_116251    avgbs_116252    avgbs_35473    avgbs_69241    avgbs_62464    avgbs_205938    avgbs_204856    avgbs_17565    avgbs_3095    avgbs_3096    avgbs_86684    avgbs_240826    avgbs_118135    avgbs_837    avgbs_52131    avgbs_53037    avgbs_53038    avgbs_71691    avgbs_76206    avgbs_31726    avgbs_71690   
   2C 32 
 
 
  avgbs_16245    avgbs_60041    avgbs_87648    avgbs_87649    avgbs_221934    avgbs_95736    avgbs_34812    avgbs_7922    avgbs_12489    avgbs_15420    avgbs_15422    avgbs_32096    avgbs_34496    avgbs_38662    avgbs_48928    avgbs_53373    avgbs_78001    avgbs_83634    avgbs_89718    avgbs_92630    avgbs_116438    avgbs_119311    avgbs_6K_113427    avgbs_229704    avgbs_230864    avgbs_207512    avgbs_1747    avgbs_205789    avgbs_102464    avgbs_122062    avgbs_244092    avgbs_118707    avgbs_203603    avgbs_28101    avgbs_87725    avgbs_17389    avgbs_52130    avgbs_32299    avgbs_32298    avgbs_38687    avgbs_235503   
   2C 33 
  GMI_ES01_c13657_337     GMI_ES02_c33014_195    
 
  avgbs_57484    avgbs_72346    avgbs_48214    avgbs_40171    avgbs_106272    avgbs_204472    avgbs_27054    avgbs_51344    avgbs_105759    avgbs_218188    avgbs_4805    avgbs_15419    avgbs_15421    avgbs_16084    avgbs_26919    avgbs_63284    avgbs_94350    avgbs_109583    avgbs_116250    avgbs_116253    avgbs_124238    avgbs_124239    avgbs_124240    avgbs_125384    avgbs_125385    avgbs_232311    avgbs_207198    avgbs_43235    avgbs_70701    avgbs_230027    avgbs_9597    avgbs_30456    avgbs_55418    avgbs_71113    avgbs_85590    avgbs_94014    avgbs_120731    avgbs_56744    avgbs_125626    avgbs_122522    avgbs_76121    avgbs_76122    avgbs_76123    avgbs_5546    avgbs_12488    avgbs_17749    avgbs_29542    avgbs_33790    avgbs_35694    avgbs_35695    avgbs_35696    avgbs_35697    avgbs_58543    avgbs_66270    avgbs_66271    avgbs_78000    avgbs_107719    avgbs_112053    avgbs_112514    avgbs_121110    avgbs_202810    avgbs_202860    avgbs_237753    avgbs_204783    avgbs_204789    avgbs_206010    avgbs_1861    avgbs_97170    avgbs_97171    avgbs_108895    avgbs_115051    avgbs_125426    avgbs_109503    avgbs_203000    avgbs_21918    avgbs_65260    avgbs_74167    avgbs_238822    avgbs_8414    avgbs_29780    avgbs_105548    avgbs_105549    avgbs_77416    avgbs_208354    avgbs_40536   
   2C 34 
 
 
  avgbs_217400    avgbs_38272    avgbs_200260    avgbs_23941    avgbs_23942    avgbs_34917    avgbs_49273    avgbs_63578    avgbs_63579    avgbs_81248    avgbs_81249    avgbs_218805   
   2C 35 
  GMI_DS_CC8468_91     GMI_ES_CC4978_509     GMI_ES01_c8470_599    
 
  avgbs_24574    avgbs_78462    avgbs_213610    avgbs_49965    avgbs_48189    avgbs_55749    avgbs_216743    avgbs_4790    avgbs_11633    avgbs_33819    avgbs_56139    avgbs_83027    avgbs_213310    avgbs_230564    avgbs_75739    avgbs_97504    avgbs_120567    avgbs_123914    avgbs_57147    avgbs_229473    avgbs_232739    avgbs_4708    avgbs_4709    avgbs_15271    avgbs_107669    avgbs_107670    avgbs_17074    avgbs_33935    avgbs_47017    avgbs_53159    avgbs_63606    avgbs_81480    avgbs_81481    avgbs_81832    avgbs_98478    avgbs_98480    avgbs_106935    avgbs_106936    avgbs_111816    avgbs_202955    avgbs_206503    avgbs_112397    avgbs_241931    avgbs_37329    avgbs_37330    avgbs_89939    avgbs_54834   
   2C 36 
  GMI_ES_CC6708_301     GMI_ES_CC11290_204     GMI_ES_CC8700_285     GMI_ES01_c1635_353    
 
  avgbs_1705    avgbs_4912    avgbs_13466    avgbs_18760    avgbs_20315    avgbs_20466    avgbs_20918    avgbs_20919    avgbs_21573    avgbs_21574    avgbs_23357    avgbs_23614    avgbs_36915    avgbs_40807    avgbs_48782    avgbs_50167    avgbs_50168    avgbs_51411    avgbs_53158    avgbs_56376    avgbs_58167    avgbs_62961    avgbs_66177    avgbs_68361    avgbs_69752    avgbs_73825    avgbs_74047    avgbs_75381    avgbs_82649    avgbs_87774    avgbs_87881    avgbs_88293    avgbs_88888    avgbs_93119    avgbs_95151    avgbs_101388    avgbs_102528    avgbs_105110    avgbs_106012    avgbs_107295    avgbs_107297    avgbs_107950    avgbs_117101    avgbs_118540    avgbs_119238    avgbs_119723    avgbs_122027    avgbs_122972    avgbs_123145    avgbs_123174    avgbs_124685    avgbs_124686    avgbs_216469    avgbs_201283    avgbs_216920    avgbs_217324    avgbs_218204    avgbs_214206    avgbs_219859    avgbs_220293    avgbs_205424    avgbs_221077    avgbs_221191    avgbs_221305    avgbs_221362    avgbs_222443    avgbs_222881    avgbs_114722    avgbs_18812    avgbs_22159    avgbs_33721    avgbs_48351    avgbs_51170    avgbs_68406    avgbs_69242    avgbs_70598    avgbs_72139    avgbs_79399    avgbs_96075    avgbs_118448    avgbs_123915    avgbs_218766    avgbs_214682    avgbs_219914    avgbs_200680    avgbs_212754    avgbs_212944    avgbs_38202    avgbs_47764    avgbs_51158    avgbs_53992    avgbs_63207    avgbs_67838    avgbs_119649    avgbs_51735    avgbs_51736    avgbs_73821    avgbs_74654    avgbs_94707    avgbs_94708    avgbs_100251    avgbs_100252    avgbs_117163    avgbs_120924    avgbs_220200    avgbs_217427    avgbs_206602    avgbs_77537    avgbs_60353    avgbs_60355    avgbs_63909    avgbs_76515    avgbs_95577    avgbs_219630    avgbs_86029    avgbs_3008    avgbs_13636    avgbs_55991    avgbs_59729    avgbs_74245    avgbs_74246    avgbs_81610    avgbs_107281    avgbs_109002    avgbs_119075    avgbs_201113    avgbs_204638    avgbs_225221    avgbs_205905    avgbs_38203    avgbs_107293    avgbs_107294    avgbs_201095    avgbs_93466    avgbs_93467    avgbs_52335    avgbs_104199    avgbs_232132    avgbs_108285    avgbs_77538    avgbs_20577    avgbs_37304    avgbs_20861    avgbs_96697    avgbs_96698    avgbs_103095    avgbs_206147    avgbs_50776    avgbs_3819    avgbs_5704    avgbs_42146    avgbs_79330    avgbs_93232    avgbs_98784    avgbs_105684    avgbs_105706    avgbs_105707    avgbs_114548    avgbs_115193    avgbs_122358    avgbs_125564    avgbs_125565    avgbs_200550    avgbs_98596    avgbs_11312    avgbs_39217    avgbs_66139    avgbs_66140    avgbs_106122    avgbs_93002    avgbs_93003    avgbs_93004    avgbs_67335    avgbs_50270    avgbs_50271    avgbs_11400    avgbs_96644    avgbs_59659    avgbs_119115    avgbs_200071    avgbs_79642    avgbs_113262    avgbs_113398    avgbs_225566    avgbs_225572   
   2C 37 
  GMI_ES17_lrc14708_436     GMI_DS_CC4033_368    
 
  avgbs_61898    avgbs_219520    avgbs_220500    avgbs_205732    avgbs_6K_64994    avgbs_70599    avgbs_210128    avgbs_38200    avgbs_38201    avgbs_82053    avgbs_8717    avgbs_56995    avgbs_56996    avgbs_113767    avgbs_113769    avgbs_115976    avgbs_212578    avgbs_5590    avgbs_210329    avgbs_207151    avgbs_34919    avgbs_8073    avgbs_60354    avgbs_32535    avgbs_40892    avgbs_47353    avgbs_49678    avgbs_78620    avgbs_230156    avgbs_230221    avgbs_68359    avgbs_68360    avgbs_25178    avgbs_31364    avgbs_31378    avgbs_118291    avgbs_203097    avgbs_685    avgbs_9327    avgbs_35226    avgbs_47911    avgbs_59727    avgbs_79605    avgbs_87466    avgbs_110628    avgbs_114369    avgbs_117621    avgbs_125975    avgbs_202103    avgbs_202121    avgbs_203139    avgbs_237799    avgbs_204905    avgbs_205933    avgbs_206379    avgbs_29123    avgbs_50998    avgbs_77177    avgbs_85806    avgbs_93036    avgbs_112089    avgbs_112090    avgbs_114954    avgbs_233413    avgbs_18174    avgbs_207939    avgbs_9644    avgbs_52097    avgbs_59722    avgbs_105770    avgbs_42239    avgbs_47464    avgbs_77535    avgbs_77536    avgbs_83910    avgbs_83911    avgbs_119749    avgbs_15710    avgbs_15711    avgbs_102900    avgbs_125572    avgbs_234938    avgbs_239257    avgbs_206395    avgbs_36268    avgbs_62638    avgbs_41193    avgbs_54876    avgbs_76573    avgbs_99398    avgbs_113078    avgbs_74638    avgbs_47491    avgbs_67497    avgbs_204255    avgbs_43224    avgbs_93694    avgbs_80951    avgbs_243613    avgbs_20082    avgbs_102892    avgbs_28736    avgbs_108236    avgbs_123489    avgbs_88472    avgbs_57154    avgbs_57155    avgbs_57156    avgbs_64468    avgbs_22786    avgbs_22787    avgbs_74247    avgbs_77399    avgbs_77400    avgbs_97943    avgbs_103298    avgbs_238724   
   2C 38 
  GMI_ES02_c17596_199     GMI_ES_CC9730_217     GMI_DS_CC6030_255     GMI_ES02_c38444_270     GMI_ES01_c3327_180     GMI_ES02_lrc37378_471     GMI_ES01_c24681_389    
 
  avgbs_109    avgbs_673    avgbs_674    avgbs_1250    avgbs_10108    avgbs_19300    avgbs_24874    avgbs_46630    avgbs_71082    avgbs_6K_73687    avgbs_109605    avgbs_118019    avgbs_118946    avgbs_216264    avgbs_221716    avgbs_206705    avgbs_222741    avgbs_60549    avgbs_73743    avgbs_92582    avgbs_113399    avgbs_210531    avgbs_221287    avgbs_222515    avgbs_28918    avgbs_28920    avgbs_45949    avgbs_7085    avgbs_7086    avgbs_29122    avgbs_2038    avgbs_2991    avgbs_25448    avgbs_30954    avgbs_30955    avgbs_30956    avgbs_88333    avgbs_88334    avgbs_200108    avgbs_221910    avgbs_124991    avgbs_34920    avgbs_95571    avgbs_27080    avgbs_28300    avgbs_34273    avgbs_61914    avgbs_98764    avgbs_98765    avgbs_103817    avgbs_231508    avgbs_205615    avgbs_215366    avgbs_119602    avgbs_12517    avgbs_6K_14790    avgbs_28919    avgbs_40046    avgbs_40047    avgbs_40048    avgbs_40049    avgbs_58573    avgbs_74798    avgbs_75684    avgbs_100222    avgbs_96990    avgbs_22063    avgbs_35375    avgbs_60715    avgbs_63208    avgbs_88339    avgbs_229999    avgbs_231531    avgbs_229099    avgbs_26332    avgbs_40404    avgbs_45950    avgbs_56287    avgbs_122336    avgbs_1016    avgbs_1017    avgbs_9177    avgbs_22062    avgbs_25261    avgbs_63571    avgbs_86521    avgbs_99492    avgbs_107613    avgbs_112097    avgbs_118932    avgbs_118933    avgbs_124834    avgbs_201523    avgbs_234641    avgbs_202720    avgbs_203475    avgbs_204086    avgbs_205446    avgbs_205864    avgbs_96440    avgbs_105341    avgbs_105342    avgbs_64469    avgbs_78114    avgbs_104498    avgbs_114453    avgbs_116826    avgbs_211145    avgbs_240944    avgbs_39318    avgbs_41610    avgbs_103492    avgbs_203474    avgbs_57480    avgbs_238164    avgbs_208318    avgbs_61867    avgbs_61868    avgbs_208430    avgbs_98602    avgbs_221798    avgbs_52485    avgbs_237310    avgbs_108976    avgbs_61849    avgbs_68840    avgbs_28970   
   2C 39 
  GMI_ES15_c2110_730     GMI_DS_CC9934_185    
 
  avgbs_12440    avgbs_59493    avgbs_45951    avgbs_208620    avgbs_100221    avgbs_117543    avgbs_18188    avgbs_11797    avgbs_18761    avgbs_20107    avgbs_73673    avgbs_73674    avgbs_85606    avgbs_125802    avgbs_204222    avgbs_205220    avgbs_240344    avgbs_244481    avgbs_91869    avgbs_115618    avgbs_243236    avgbs_67377    avgbs_47382    avgbs_60351    avgbs_60352    avgbs_107552    avgbs_107553    avgbs_95199    avgbs_241842    avgbs_54044    avgbs_241436   
   2C 40 
  GMI_ES02_c8676_360     GMI_ES01_c3302_178    
 
  avgbs_700    avgbs_5009    avgbs_10668    avgbs_12981    avgbs_12982    avgbs_12983    avgbs_36040    avgbs_51277    avgbs_51278    avgbs_52692    avgbs_66046    avgbs_66664    avgbs_94560    avgbs_218170    avgbs_219959    avgbs_200545    avgbs_222046    avgbs_222408    avgbs_59492    avgbs_106947    avgbs_209489    avgbs_87092    avgbs_93198    avgbs_12126    avgbs_71852    avgbs_97904    avgbs_2463    avgbs_71989    avgbs_71990    avgbs_54731    avgbs_12798    avgbs_25458    avgbs_81488    avgbs_86781    avgbs_118718    avgbs_121567    avgbs_121568    avgbs_16464    avgbs_22009    avgbs_24770    avgbs_24771    avgbs_35350    avgbs_35376    avgbs_39655    avgbs_44141    avgbs_52637    avgbs_52638    avgbs_56765    avgbs_58168    avgbs_64437    avgbs_68097    avgbs_74087    avgbs_77663    avgbs_79795    avgbs_97436    avgbs_97437    avgbs_101755    avgbs_104517    avgbs_106966    avgbs_107369    avgbs_107769    avgbs_110170    avgbs_112107    avgbs_114711    avgbs_115426    avgbs_115427    avgbs_201308    avgbs_203027    avgbs_238178    avgbs_204287    avgbs_25429    avgbs_25430    avgbs_236728    avgbs_3783    avgbs_42195    avgbs_42196    avgbs_39912    avgbs_52832    avgbs_118199    avgbs_118200    avgbs_205889    avgbs_36670    avgbs_23786    avgbs_106141    avgbs_117307    avgbs_203274    avgbs_99446    avgbs_113422    avgbs_201097    avgbs_44712    avgbs_8849    avgbs_8850    avgbs_8851    avgbs_483    avgbs_237676    avgbs_233754    avgbs_241542    avgbs_207335   
   2C 41 
  GMI_DS_CC2679_57     GMI_ES01_c12277_1252     GMI_ES01_c3298_226    
 
  avgbs_7178    avgbs_14426    avgbs_15685    avgbs_30137    avgbs_30138    avgbs_32122    avgbs_79344    avgbs_83112    avgbs_85054    avgbs_88269    avgbs_95525    avgbs_112947    avgbs_200018    avgbs_208776    avgbs_217911    avgbs_200220    avgbs_220744    avgbs_25824    avgbs_3975    avgbs_93197    avgbs_12125    avgbs_24427    avgbs_24428    avgbs_26094    avgbs_46798    avgbs_75987    avgbs_108930    avgbs_200521    avgbs_4017    avgbs_32103    avgbs_32104    avgbs_45058    avgbs_45059    avgbs_64373    avgbs_64620    avgbs_12252    avgbs_15556    avgbs_42178    avgbs_208960    avgbs_35349    avgbs_36279    avgbs_46451    avgbs_48944    avgbs_59491    avgbs_61497    avgbs_75264    avgbs_81124    avgbs_231094    avgbs_231639    avgbs_68095    avgbs_68096    avgbs_3974    avgbs_28334    avgbs_58648    avgbs_66258    avgbs_66939    avgbs_68017    avgbs_68018    avgbs_71665    avgbs_72971    avgbs_97295    avgbs_110167    avgbs_27079    avgbs_58997    avgbs_58998    avgbs_232304    avgbs_49625    avgbs_49626    avgbs_93252    avgbs_1391    avgbs_4263    avgbs_6385    avgbs_6964    avgbs_7381    avgbs_7607    avgbs_7640    avgbs_7641    avgbs_8194    avgbs_9472    avgbs_11814    avgbs_16546    avgbs_18881    avgbs_25758    avgbs_25783    avgbs_30392    avgbs_30782    avgbs_37200    avgbs_37201    avgbs_38474    avgbs_38475    avgbs_39702    avgbs_51275    avgbs_51724    avgbs_52597    avgbs_61928    avgbs_61929    avgbs_68032    avgbs_68034    avgbs_68040    avgbs_75133    avgbs_75134    avgbs_78803    avgbs_79211    avgbs_80031    avgbs_80148    avgbs_80149    avgbs_83242    avgbs_86940    avgbs_87810    avgbs_87811    avgbs_88807    avgbs_88808    avgbs_89819    avgbs_96548    avgbs_96549    avgbs_101094    avgbs_106478    avgbs_110166    avgbs_111307    avgbs_111962    avgbs_111972    avgbs_113364    avgbs_115860    avgbs_120640    avgbs_121609    avgbs_124546    avgbs_124901    avgbs_124957    avgbs_126012    avgbs_126013    avgbs_126014    avgbs_201600    avgbs_201647    avgbs_202643    avgbs_200178    avgbs_202811    avgbs_202883    avgbs_203611    avgbs_203686    avgbs_204021    avgbs_239457    avgbs_205584    avgbs_205866    avgbs_205888    avgbs_241720    avgbs_206599    avgbs_1412    avgbs_2369    avgbs_20602    avgbs_27248    avgbs_49729    avgbs_75809    avgbs_87079    avgbs_105921    avgbs_115545    avgbs_120571    avgbs_121161    avgbs_240036    avgbs_206467    avgbs_3661    avgbs_20040    avgbs_23843    avgbs_25296    avgbs_29044    avgbs_54183    avgbs_54184    avgbs_54185    avgbs_64028    avgbs_64030    avgbs_74853    avgbs_100866    avgbs_100867    avgbs_120732    avgbs_120733    avgbs_121965    avgbs_244701    avgbs_25685    avgbs_68772    avgbs_81218    avgbs_86163    avgbs_8930    avgbs_53750    avgbs_73669    avgbs_74852    avgbs_88952    avgbs_90015    avgbs_111904    avgbs_111905    avgbs_113214    avgbs_116275    avgbs_116276    avgbs_116277    avgbs_201916    avgbs_50911    avgbs_78330    avgbs_78331    avgbs_103188    avgbs_203604    avgbs_25273    avgbs_25274    avgbs_49902    avgbs_59083    avgbs_59084    avgbs_67674    avgbs_236905    avgbs_31100    avgbs_65115    avgbs_65370    avgbs_88119    avgbs_97518    avgbs_116368    avgbs_201559    avgbs_9506    avgbs_9507    avgbs_34145    avgbs_119287    avgbs_206422    avgbs_207993    avgbs_8249    avgbs_64026    avgbs_64029    avgbs_99458    avgbs_205668    avgbs_36290    avgbs_84233    avgbs_233706    avgbs_91930    avgbs_91931    avgbs_46820    avgbs_49509    avgbs_105593    avgbs_8608    avgbs_74726    avgbs_74727    avgbs_201297    avgbs_34892    avgbs_34893    avgbs_241395    avgbs_10068    avgbs_19984    avgbs_68423    avgbs_68424    avgbs_244147    avgbs_205967    avgbs_206328    avgbs_93001    avgbs_203780    avgbs_241383    avgbs_29328    avgbs_29329    avgbs_35501    avgbs_124995    avgbs_24198    avgbs_235510   
   2C 42 
  GMI_ES02_c37525_294    
 
  avgbs_100067    avgbs_114085    avgbs_121323    avgbs_207150    avgbs_13153    avgbs_26505    avgbs_32675    avgbs_41307    avgbs_52548    avgbs_59730    avgbs_87862    avgbs_121589    avgbs_210265    avgbs_47828    avgbs_26946    avgbs_81199    avgbs_102459    avgbs_104458    avgbs_105166    avgbs_105167    avgbs_109076    avgbs_115032    avgbs_115033    avgbs_121246    avgbs_212271    avgbs_38104    avgbs_38105    avgbs_99033    avgbs_35089    avgbs_10256    avgbs_10257    avgbs_12061    avgbs_19524    avgbs_62496    avgbs_103966    avgbs_126056    avgbs_231252    avgbs_231815    avgbs_205831    avgbs_232125    avgbs_46522    avgbs_90014    avgbs_204210    avgbs_6K_4371    avgbs_84611    avgbs_232325    avgbs_6184    avgbs_6185    avgbs_62330    avgbs_42508    avgbs_2894    avgbs_19404    avgbs_19530    avgbs_20967    avgbs_55396    avgbs_61378    avgbs_73656    avgbs_103323    avgbs_106546    avgbs_107539    avgbs_111308    avgbs_114858    avgbs_118871    avgbs_122048    avgbs_201568    avgbs_202521    avgbs_203202    avgbs_214345    avgbs_200905    avgbs_17895    avgbs_75576    avgbs_84234    avgbs_124477    avgbs_202289    avgbs_22326    avgbs_22327    avgbs_69843    avgbs_243939    avgbs_207258   
   2C 43 
  GMI_ES01_c9044_416     GMI_ES01_c11537_64     GMI_ES01_c14226_61     GMI_DS_CC2032_276     GMI_ES01_c24758_394     GMI_ES15_c112_624    
 
  avgbs_12062    avgbs_91267    avgbs_91586    avgbs_231636    avgbs_42681    avgbs_47549    avgbs_52047    avgbs_103512    avgbs_36139    avgbs_207950    avgbs_67134    avgbs_67135   
   2C 44 
  GMI_ES15_c4387_160     GMI_ES15_c4231_158     GMI_ES_CC13637_173     GMI_DS_CC5529_175     GMI_ES15_c7666_522     GMI_ES_CC3989_242     GMI_ES15_c2996_540     GMI_ES02_c8845_199     GMI_ES_CC7315_292     GMI_ES15_c6925_619     GMI_ES17_lrc20311_707     GMI_ES17_c4200_911     GMI_ES_CC10804_287     GMI_ES17_c7296_694     GMI_ES_CC3284_453     GMI_ES02_c4655_137    
 
  avgbs_54905    avgbs_54906    avgbs_54907    avgbs_1576    avgbs_1896    avgbs_2323    avgbs_2325    avgbs_6K_2200    avgbs_2682    avgbs_3299    avgbs_7385    avgbs_10096    avgbs_11043    avgbs_11488    avgbs_11948    avgbs_12813    avgbs_14286    avgbs_15691    avgbs_16098    avgbs_16720    avgbs_16845    avgbs_16848    avgbs_17011    avgbs_17618    avgbs_18694    avgbs_19296    avgbs_19297    avgbs_20796    avgbs_21764    avgbs_21910    avgbs_23792    avgbs_24956    avgbs_25045    avgbs_26172    avgbs_26175    avgbs_26686    avgbs_26740    avgbs_27637    avgbs_28829    avgbs_29803    avgbs_29804    avgbs_30052    avgbs_31722    avgbs_32364    avgbs_32489    avgbs_32490    avgbs_33961    avgbs_35601    avgbs_35602    avgbs_36146    avgbs_36469    avgbs_38529    avgbs_38766    avgbs_39561    avgbs_40035    avgbs_44567    avgbs_44717    avgbs_45724    avgbs_45725    avgbs_45924    avgbs_45925    avgbs_46604    avgbs_49736    avgbs_49737    avgbs_52253    avgbs_54938    avgbs_54939    avgbs_55194    avgbs_55552    avgbs_55931    avgbs_59337    avgbs_59338    avgbs_59850    avgbs_60003    avgbs_61523    avgbs_62396    avgbs_62775    avgbs_65615    avgbs_67546    avgbs_68346    avgbs_69747    avgbs_70217    avgbs_72454    avgbs_77222    avgbs_77223    avgbs_77693    avgbs_78694    avgbs_78695    avgbs_78696    avgbs_78697    avgbs_79193    avgbs_79194    avgbs_80385    avgbs_80751    avgbs_80752    avgbs_80755    avgbs_83224    avgbs_85285    avgbs_87373    avgbs_87646    avgbs_88216    avgbs_88928    avgbs_89738    avgbs_90129    avgbs_90130    avgbs_90137    avgbs_90138    avgbs_91541    avgbs_91998    avgbs_95953    avgbs_96968    avgbs_97399    avgbs_97429    avgbs_97982    avgbs_100065    avgbs_100066    avgbs_100498    avgbs_101378    avgbs_101897    avgbs_102056    avgbs_102057    avgbs_103955    avgbs_103956    avgbs_104020    avgbs_104021    avgbs_104352    avgbs_104353    avgbs_106482    avgbs_106928    avgbs_107077    avgbs_109196    avgbs_110827    avgbs_111248    avgbs_111469    avgbs_114611    avgbs_116924    avgbs_117219    avgbs_117220    avgbs_118367    avgbs_118498    avgbs_118703    avgbs_120252    avgbs_120758    avgbs_120759    avgbs_122487    avgbs_123140    avgbs_123691    avgbs_200013    avgbs_216628    avgbs_216849    avgbs_208929    avgbs_217161    avgbs_200117    avgbs_217463    avgbs_200162    avgbs_200173    avgbs_209463    avgbs_217614    avgbs_217993    avgbs_218123    avgbs_218332    avgbs_218348    avgbs_218355    avgbs_218532    avgbs_218589    avgbs_200302    avgbs_218654    avgbs_218662    avgbs_218765    avgbs_200352    avgbs_219200    avgbs_219544    avgbs_219633    avgbs_219663    avgbs_219693    avgbs_210862    avgbs_204401    avgbs_220132    avgbs_220139    avgbs_220171    avgbs_220316    avgbs_220369    avgbs_200540    avgbs_220522    avgbs_220551    avgbs_204904    avgbs_220652    avgbs_220870    avgbs_205379    avgbs_221137    avgbs_200628    avgbs_221513    avgbs_221602    avgbs_221738    avgbs_205850    avgbs_200715    avgbs_212136    avgbs_221963    avgbs_221992    avgbs_222185    avgbs_200782    avgbs_222282    avgbs_200797    avgbs_222341    avgbs_200834    avgbs_222603    avgbs_222616    avgbs_200875    avgbs_207612    avgbs_222776    avgbs_207759    avgbs_9128    avgbs_40034    avgbs_86054    avgbs_99483    avgbs_99484    avgbs_103394    avgbs_209267    avgbs_200746    avgbs_200754    avgbs_4222    avgbs_9056    avgbs_9057    avgbs_10784    avgbs_13044    avgbs_16379    avgbs_20231    avgbs_21867    avgbs_28936    avgbs_31377    avgbs_34786    avgbs_35569    avgbs_35570    avgbs_36450    avgbs_36674    avgbs_43887    avgbs_48878    avgbs_54316    avgbs_54682    avgbs_57417    avgbs_58402    avgbs_60228    avgbs_60504    avgbs_61536    avgbs_63980    avgbs_75185    avgbs_82099    avgbs_86549    avgbs_86550    avgbs_86551    avgbs_88528    avgbs_92761    avgbs_94023    avgbs_94024    avgbs_95082    avgbs_98898    avgbs_98899    avgbs_100518    avgbs_106700    avgbs_119126    avgbs_122447    avgbs_124794    avgbs_208702    avgbs_209884    avgbs_200358    avgbs_210521    avgbs_219422    avgbs_210733    avgbs_211036    avgbs_200779    avgbs_212783    avgbs_45923    avgbs_63333    avgbs_109756    avgbs_210071    avgbs_7316    avgbs_9042    avgbs_9043    avgbs_20701    avgbs_21929    avgbs_23620    avgbs_25392    avgbs_32674    avgbs_35178    avgbs_35772    avgbs_46490    avgbs_47662    avgbs_47809    avgbs_54064    avgbs_57243    avgbs_57921    avgbs_61173    avgbs_65891    avgbs_65960    avgbs_67898    avgbs_70320    avgbs_70832    avgbs_70833    avgbs_73768    avgbs_81499    avgbs_81596    avgbs_88507    avgbs_93361    avgbs_93362    avgbs_98740    avgbs_98741    avgbs_110327    avgbs_123548    avgbs_125035    avgbs_125643    avgbs_3319    avgbs_210866    avgbs_200507    avgbs_22546    avgbs_22547    avgbs_6K_70684    avgbs_110986    avgbs_6513    avgbs_10073    avgbs_15590    avgbs_16503    avgbs_16504    avgbs_16530    avgbs_18438    avgbs_19647    avgbs_19648    avgbs_21899    avgbs_24051    avgbs_24441    avgbs_25025    avgbs_25582    avgbs_27948    avgbs_28075    avgbs_28076    avgbs_30420    avgbs_33854    avgbs_40019    avgbs_40020    avgbs_43611    avgbs_46850    avgbs_47035    avgbs_47394    avgbs_51440    avgbs_53754    avgbs_53977    avgbs_56005    avgbs_56309    avgbs_59450    avgbs_59671    avgbs_59672    avgbs_59673    avgbs_59723    avgbs_60621    avgbs_61553    avgbs_68345    avgbs_73368    avgbs_73626    avgbs_80252    avgbs_91156    avgbs_98837    avgbs_103612    avgbs_109930    avgbs_111680    avgbs_116798    avgbs_209226    avgbs_36453    avgbs_210097    avgbs_109301    avgbs_200801    avgbs_24196    avgbs_24641    avgbs_38737    avgbs_39902    avgbs_47332    avgbs_47908    avgbs_58316    avgbs_71260    avgbs_86180    avgbs_115611    avgbs_207382    avgbs_3827    avgbs_3841    avgbs_4065    avgbs_8605    avgbs_10775    avgbs_16423    avgbs_16427    avgbs_17121    avgbs_22862    avgbs_23441    avgbs_32350    avgbs_38364    avgbs_38717    avgbs_40389    avgbs_40787    avgbs_41493    avgbs_47760    avgbs_51624    avgbs_62555    avgbs_62556    avgbs_65673    avgbs_67505    avgbs_71334    avgbs_73625    avgbs_77529    avgbs_78934    avgbs_81420    avgbs_81421    avgbs_83177    avgbs_83178    avgbs_88397    avgbs_98057    avgbs_101206    avgbs_101353    avgbs_209689    avgbs_210113    avgbs_211129    avgbs_19847    avgbs_27222    avgbs_66842    avgbs_67702    avgbs_90895    avgbs_241    avgbs_50412    avgbs_71477    avgbs_89133    avgbs_101170    avgbs_14329    avgbs_91730    avgbs_201839    avgbs_66472    avgbs_70480    avgbs_62195    avgbs_243152    avgbs_107732    avgbs_242285    avgbs_70404    avgbs_44669    avgbs_112067    avgbs_112068    avgbs_49567    avgbs_84091    avgbs_105474    avgbs_105475    avgbs_205108    avgbs_243530    avgbs_10421    avgbs_3111    avgbs_5955    avgbs_31382    avgbs_41910    avgbs_41911    avgbs_41912    avgbs_235785    avgbs_79075    avgbs_124819    avgbs_124820    avgbs_6K_85    avgbs_1609    avgbs_1688    avgbs_1851    avgbs_2060    avgbs_2141    avgbs_2324    avgbs_2790    avgbs_4110    avgbs_6038    avgbs_6060    avgbs_6082    avgbs_6374    avgbs_6516    avgbs_6996    avgbs_7397    avgbs_8430    avgbs_8625    avgbs_9937    avgbs_10753    avgbs_11225    avgbs_11371    avgbs_14730    avgbs_14731    avgbs_14732    avgbs_14866    avgbs_14871    avgbs_14935    avgbs_15395    avgbs_15563    avgbs_15564    avgbs_17010    avgbs_17012    avgbs_17018    avgbs_17122    avgbs_17882    avgbs_18135    avgbs_18437    avgbs_19138    avgbs_19281    avgbs_19365    avgbs_19957    avgbs_20113    avgbs_20417    avgbs_20426    avgbs_21175    avgbs_21176    avgbs_21216    avgbs_21321    avgbs_21449    avgbs_21543    avgbs_21617    avgbs_23779    avgbs_24148    avgbs_24149    avgbs_24696    avgbs_24697    avgbs_24907    avgbs_24955    avgbs_25674    avgbs_25829    avgbs_27090    avgbs_28126    avgbs_29885    avgbs_29948    avgbs_29949    avgbs_29951    avgbs_29952    avgbs_29984    avgbs_30540    avgbs_31674    avgbs_31675    avgbs_32092    avgbs_32106    avgbs_32107    avgbs_32327    avgbs_33076    avgbs_34987    avgbs_35015    avgbs_35106    avgbs_35107    avgbs_35650    avgbs_36973    avgbs_37395    avgbs_38313    avgbs_38314    avgbs_38315    avgbs_38704    avgbs_39216    avgbs_39294    avgbs_39675    avgbs_39676    avgbs_40151    avgbs_40378    avgbs_40639    avgbs_41550    avgbs_41551    avgbs_42197    avgbs_42386    avgbs_42442    avgbs_42486    avgbs_42487    avgbs_42507    avgbs_43417    avgbs_44012    avgbs_45076    avgbs_45077    avgbs_45797    avgbs_45853    avgbs_46621    avgbs_46836    avgbs_46843    avgbs_47147    avgbs_48925    avgbs_49860    avgbs_50799    avgbs_50843    avgbs_50844    avgbs_51608    avgbs_51609    avgbs_52549    avgbs_54063    avgbs_54970    avgbs_55406    avgbs_55553    avgbs_56282    avgbs_56317    avgbs_56595    avgbs_59214    avgbs_60956    avgbs_61037    avgbs_61038    avgbs_62590    avgbs_62822    avgbs_62871    avgbs_62874    avgbs_63049    avgbs_63192    avgbs_63365    avgbs_63646    avgbs_64914    avgbs_65144    avgbs_65356    avgbs_66452    avgbs_67389    avgbs_67445    avgbs_67825    avgbs_67841    avgbs_68299    avgbs_71288    avgbs_71551    avgbs_71874    avgbs_72645    avgbs_72755    avgbs_73793    avgbs_74011    avgbs_74371    avgbs_74372    avgbs_76325    avgbs_76502    avgbs_76674    avgbs_76924    avgbs_77008    avgbs_77322    avgbs_77324    avgbs_78120    avgbs_78196    avgbs_79483    avgbs_80408    avgbs_80698    avgbs_81353    avgbs_81497    avgbs_83762    avgbs_84563    avgbs_85200    avgbs_85323    avgbs_85324    avgbs_85407    avgbs_85408    avgbs_85804    avgbs_85871    avgbs_86790    avgbs_6K_81921    avgbs_88570    avgbs_88594    avgbs_89949    avgbs_90897    avgbs_91809    avgbs_91935    avgbs_91937    avgbs_92343    avgbs_93122    avgbs_93481    avgbs_93700    avgbs_95376    avgbs_96212    avgbs_96463    avgbs_97699    avgbs_98760    avgbs_99491    avgbs_99949    avgbs_101970    avgbs_102010    avgbs_103098    avgbs_103395    avgbs_104457    avgbs_105365    avgbs_105366    avgbs_105383    avgbs_105384    avgbs_106093    avgbs_106543    avgbs_106964    avgbs_107078    avgbs_107309    avgbs_107310    avgbs_107464    avgbs_107497    avgbs_107596    avgbs_108819    avgbs_108931    avgbs_109395    avgbs_109489    avgbs_109633    avgbs_110011    avgbs_110012    avgbs_110113    avgbs_110114    avgbs_110115    avgbs_110116    avgbs_110464    avgbs_110505    avgbs_110508    avgbs_110576    avgbs_110710    avgbs_111153    avgbs_111154    avgbs_111155    avgbs_111222    avgbs_111344    avgbs_111842    avgbs_111956    avgbs_112036    avgbs_112096    avgbs_112241    avgbs_112563    avgbs_112564    avgbs_112706    avgbs_112707    avgbs_112889    avgbs_113235    avgbs_113751    avgbs_113831    avgbs_113832    avgbs_114239    avgbs_114407    avgbs_114484    avgbs_114491    avgbs_114636    avgbs_114671    avgbs_114872    avgbs_114873    avgbs_114972    avgbs_115082    avgbs_115084    avgbs_115350    avgbs_115351    avgbs_115455    avgbs_115473    avgbs_115612    avgbs_115702    avgbs_115703    avgbs_116057    avgbs_117477    avgbs_118481    avgbs_119098    avgbs_119099    avgbs_119129    avgbs_119244    avgbs_119440    avgbs_119593    avgbs_119677    avgbs_120159    avgbs_120160    avgbs_120475    avgbs_120521    avgbs_120522    avgbs_120799    avgbs_121409    avgbs_121524    avgbs_122244    avgbs_122366    avgbs_122586    avgbs_122746    avgbs_123177    avgbs_123399    avgbs_123408    avgbs_124027    avgbs_124169    avgbs_124225    avgbs_124226    avgbs_124227    avgbs_124443    avgbs_124449    avgbs_124634    avgbs_124638    avgbs_124640    avgbs_124831    avgbs_124872    avgbs_124893    avgbs_125697    avgbs_125838    avgbs_125839    avgbs_125991    avgbs_126010    avgbs_126011    avgbs_126097    avgbs_126217    avgbs_126259    avgbs_201037    avgbs_201047    avgbs_201121    avgbs_201415    avgbs_201702    avgbs_201810    avgbs_202116    avgbs_235121    avgbs_235698    avgbs_235753    avgbs_236067    avgbs_202978    avgbs_203005    avgbs_203091    avgbs_236665    avgbs_203388    avgbs_236860    avgbs_50481    avgbs_203623    avgbs_203748    avgbs_203841    avgbs_237790    avgbs_203975    avgbs_238012    avgbs_238097    avgbs_204090    avgbs_238414    avgbs_204296    avgbs_204440    avgbs_204441    avgbs_204469    avgbs_204584    avgbs_239022    avgbs_204713    avgbs_204803    avgbs_205062    avgbs_205071    avgbs_205347    avgbs_205445    avgbs_205455    avgbs_205585    avgbs_205612    avgbs_205744    avgbs_205764    avgbs_205769    avgbs_241348    avgbs_242129    avgbs_206613    avgbs_206688    avgbs_206694    avgbs_206962    avgbs_207233    avgbs_207272    avgbs_207320    avgbs_207530    avgbs_243451    avgbs_207689    avgbs_207706    avgbs_207716    avgbs_207760    avgbs_207844    avgbs_207949    avgbs_208194    avgbs_244501    avgbs_208388    avgbs_208453    avgbs_244723    avgbs_6768    avgbs_19336    avgbs_21572    avgbs_24569    avgbs_25782    avgbs_26792    avgbs_29806    avgbs_33414    avgbs_36595    avgbs_36596    avgbs_36597    avgbs_43483    avgbs_51949    avgbs_51950    avgbs_52734    avgbs_52735    avgbs_52833    avgbs_53549    avgbs_54061    avgbs_55331    avgbs_55332    avgbs_55385    avgbs_56565    avgbs_60423    avgbs_60424    avgbs_61970    avgbs_61971    avgbs_62755    avgbs_65140    avgbs_66127    avgbs_66128    avgbs_72735    avgbs_86746    avgbs_88000    avgbs_88001    avgbs_88431    avgbs_6K_85179    avgbs_92586    avgbs_95767    avgbs_102964    avgbs_102965    avgbs_103756    avgbs_112031    avgbs_112040    avgbs_114187    avgbs_121291    avgbs_121292    avgbs_202587    avgbs_202816    avgbs_203706    avgbs_204705    avgbs_200678    avgbs_241555    avgbs_206812    avgbs_206971    avgbs_207805    avgbs_332    avgbs_5611    avgbs_6059    avgbs_6289    avgbs_6290    avgbs_7039    avgbs_8606    avgbs_15549    avgbs_21294    avgbs_21309    avgbs_24690    avgbs_24735    avgbs_26850    avgbs_28672    avgbs_28932    avgbs_28935    avgbs_29427    avgbs_29428    avgbs_29876    avgbs_34109    avgbs_36319    avgbs_36692    avgbs_37371    avgbs_39308    avgbs_39415    avgbs_39713    avgbs_47861    avgbs_49591    avgbs_54387    avgbs_55122    avgbs_55137    avgbs_58835    avgbs_63568    avgbs_64814    avgbs_64815    avgbs_67977    avgbs_67978    avgbs_68021    avgbs_68022    avgbs_71409    avgbs_72469    avgbs_85219    avgbs_86182    avgbs_86491    avgbs_86835    avgbs_88522    avgbs_89929    avgbs_89961    avgbs_92899    avgbs_100127    avgbs_101325    avgbs_104508    avgbs_108190    avgbs_108194    avgbs_112054    avgbs_114551    avgbs_118183    avgbs_122564    avgbs_122565    avgbs_124478    avgbs_124742    avgbs_202603    avgbs_204072    avgbs_205288    avgbs_242675    avgbs_206956    avgbs_206982    avgbs_207701    avgbs_208370    avgbs_471    avgbs_20083    avgbs_22083    avgbs_22084    avgbs_26496    avgbs_32326    avgbs_33174    avgbs_35712    avgbs_35713    avgbs_38409    avgbs_47654    avgbs_49310    avgbs_49473    avgbs_50445    avgbs_59196    avgbs_68133    avgbs_68134    avgbs_68135    avgbs_72601    avgbs_78372    avgbs_78996    avgbs_86406    avgbs_86408    avgbs_101013    avgbs_115629    avgbs_115630    avgbs_201881    avgbs_202580    avgbs_202938    avgbs_203552    avgbs_204862    avgbs_206671    avgbs_206922    avgbs_180    avgbs_3826    avgbs_15173    avgbs_15174    avgbs_19561    avgbs_26825    avgbs_28131    avgbs_29033    avgbs_29472    avgbs_35782    avgbs_37679    avgbs_42876    avgbs_45120    avgbs_45697    avgbs_47633    avgbs_53163    avgbs_67651    avgbs_67652    avgbs_69441    avgbs_69442    avgbs_70023    avgbs_71261    avgbs_78325    avgbs_87861    avgbs_88388    avgbs_91395    avgbs_91396    avgbs_93539    avgbs_98570    avgbs_99681    avgbs_100245    avgbs_100638    avgbs_100892    avgbs_102120    avgbs_119342    avgbs_119745    avgbs_119746    avgbs_122747    avgbs_124078    avgbs_201154    avgbs_201305    avgbs_34682    avgbs_236703    avgbs_230400    avgbs_237827    avgbs_240107    avgbs_241361    avgbs_207315    avgbs_12394    avgbs_17630    avgbs_28509    avgbs_30116    avgbs_51059    avgbs_52820    avgbs_58013    avgbs_66730    avgbs_93811    avgbs_94344    avgbs_111125    avgbs_112883    avgbs_119629    avgbs_202404    avgbs_238334    avgbs_2064    avgbs_3789    avgbs_11857    avgbs_11894    avgbs_12450    avgbs_21143    avgbs_21939    avgbs_25050    avgbs_26416    avgbs_26725    avgbs_34108    avgbs_36691    avgbs_37100    avgbs_39377    avgbs_41957    avgbs_41958    avgbs_43677    avgbs_54677    avgbs_63615    avgbs_67118    avgbs_73866    avgbs_78333    avgbs_79266    avgbs_79267    avgbs_92143    avgbs_92621    avgbs_92956    avgbs_92957    avgbs_94385    avgbs_96620    avgbs_115196    avgbs_120943    avgbs_201355    avgbs_202262    avgbs_202608    avgbs_235661    avgbs_202848    avgbs_203117    avgbs_237746    avgbs_238880    avgbs_207900    avgbs_244432    avgbs_6348    avgbs_6491    avgbs_9372    avgbs_20539    avgbs_25168    avgbs_25169    avgbs_34381    avgbs_39346    avgbs_40761    avgbs_40762    avgbs_60418    avgbs_66548    avgbs_67446    avgbs_72549    avgbs_97043    avgbs_107703    avgbs_108020    avgbs_109044    avgbs_122932    avgbs_123193    avgbs_202200    avgbs_205630    avgbs_206744    avgbs_208442    avgbs_19952    avgbs_19959    avgbs_22389    avgbs_23579    avgbs_40590    avgbs_40591    avgbs_40902    avgbs_43475    avgbs_48478    avgbs_48479    avgbs_49822    avgbs_63938    avgbs_66570    avgbs_70058    avgbs_74429    avgbs_75170    avgbs_79145    avgbs_79979    avgbs_82796    avgbs_82797    avgbs_93591    avgbs_93687    avgbs_99414    avgbs_103769    avgbs_105928    avgbs_105929    avgbs_106158    avgbs_113438    avgbs_202427    avgbs_238854    avgbs_241614    avgbs_207029    avgbs_207383    avgbs_2368    avgbs_2640    avgbs_6815    avgbs_9206    avgbs_26487    avgbs_28868    avgbs_30049    avgbs_39417    avgbs_40475    avgbs_40476    avgbs_43212    avgbs_43213    avgbs_91974    avgbs_113799    avgbs_122735    avgbs_122736    avgbs_233258    avgbs_203194    avgbs_236569    avgbs_206415    avgbs_207316    avgbs_208011    avgbs_10168    avgbs_19962    avgbs_22637    avgbs_22638    avgbs_25668    avgbs_25669    avgbs_29532    avgbs_37535    avgbs_37536    avgbs_54991    avgbs_66696    avgbs_72638    avgbs_77588    avgbs_78337    avgbs_99031    avgbs_100693    avgbs_105527    avgbs_105528    avgbs_105529    avgbs_111357    avgbs_116142    avgbs_123896    avgbs_123897    avgbs_203873    avgbs_237887    avgbs_238333    avgbs_205632    avgbs_242613    avgbs_208465    avgbs_5530    avgbs_22157    avgbs_29239    avgbs_30400    avgbs_36971    avgbs_39969    avgbs_42273    avgbs_47840    avgbs_51666    avgbs_54828    avgbs_58021    avgbs_58022    avgbs_75529    avgbs_76416    avgbs_80462    avgbs_114405    avgbs_233599    avgbs_236314    avgbs_238861    avgbs_205590    avgbs_207232    avgbs_243902    avgbs_8636    avgbs_8637    avgbs_8638    avgbs_10514    avgbs_21306    avgbs_26928    avgbs_27073    avgbs_27074    avgbs_27095    avgbs_27096    avgbs_38217    avgbs_54642    avgbs_56281    avgbs_67584    avgbs_75580    avgbs_82506    avgbs_86189    avgbs_86190    avgbs_86191    avgbs_94912    avgbs_94968    avgbs_96104    avgbs_96105    avgbs_103455    avgbs_203979    avgbs_204266    avgbs_204624    avgbs_207024    avgbs_208431    avgbs_33138    avgbs_33139    avgbs_35363    avgbs_35364    avgbs_37756    avgbs_45310    avgbs_45426    avgbs_51227    avgbs_61514    avgbs_63301    avgbs_63962    avgbs_75846    avgbs_84989    avgbs_89831    avgbs_111131    avgbs_202473    avgbs_204054    avgbs_204079    avgbs_240407    avgbs_206793    avgbs_1648    avgbs_17748    avgbs_17842    avgbs_18095    avgbs_18096    avgbs_18097    avgbs_23324    avgbs_23325    avgbs_28151    avgbs_28152    avgbs_34377    avgbs_34378    avgbs_40496    avgbs_45464    avgbs_55861    avgbs_59339    avgbs_64635    avgbs_68375    avgbs_71383    avgbs_74619    avgbs_76910    avgbs_86428    avgbs_86429    avgbs_89604    avgbs_89605    avgbs_96575    avgbs_100810    avgbs_102640    avgbs_107412    avgbs_107413    avgbs_107414    avgbs_107415    avgbs_111388    avgbs_111389    avgbs_113738    avgbs_118224    avgbs_121363    avgbs_201210    avgbs_201623    avgbs_202384    avgbs_203971    avgbs_206449    avgbs_242359    avgbs_110577    avgbs_669    avgbs_17841    avgbs_21307    avgbs_30543    avgbs_30544    avgbs_34854    avgbs_44685    avgbs_44899    avgbs_78383    avgbs_93010    avgbs_102107    avgbs_117571    avgbs_119628    avgbs_202600    avgbs_203188    avgbs_237745    avgbs_205569    avgbs_205674    avgbs_206797    avgbs_208428    avgbs_1139    avgbs_1140    avgbs_1141    avgbs_2025    avgbs_4105    avgbs_24334    avgbs_26101    avgbs_32210    avgbs_37678    avgbs_47606    avgbs_53842    avgbs_53843    avgbs_63796    avgbs_71446    avgbs_74696    avgbs_96791    avgbs_100327    avgbs_103092    avgbs_239077    avgbs_4669    avgbs_22102    avgbs_22103    avgbs_22104    avgbs_26719    avgbs_26720    avgbs_27723    avgbs_29561    avgbs_29719    avgbs_35608    avgbs_43869    avgbs_44894    avgbs_46164    avgbs_46165    avgbs_46166    avgbs_58535    avgbs_58536    avgbs_60417    avgbs_62314    avgbs_80005    avgbs_80006    avgbs_104612    avgbs_111772    avgbs_233059    avgbs_201096    avgbs_233263    avgbs_233562    avgbs_234091    avgbs_201687    avgbs_235460    avgbs_241770    avgbs_241885    avgbs_207303    avgbs_624    avgbs_26694    avgbs_26695    avgbs_43465    avgbs_48809    avgbs_60410    avgbs_62756    avgbs_66457    avgbs_71384    avgbs_79466    avgbs_103249    avgbs_104296    avgbs_106853    avgbs_106854    avgbs_124434    avgbs_202228    avgbs_202237    avgbs_238093    avgbs_242313    avgbs_206842    avgbs_242766    avgbs_208410    avgbs_4695    avgbs_4696    avgbs_14463    avgbs_25609    avgbs_25610    avgbs_32018    avgbs_58124    avgbs_58125    avgbs_62848    avgbs_88787    avgbs_90682    avgbs_100295    avgbs_103215    avgbs_103714    avgbs_103715    avgbs_104227    avgbs_201856    avgbs_235302    avgbs_203179    avgbs_237226    avgbs_4076    avgbs_46883    avgbs_49446    avgbs_50306    avgbs_54060    avgbs_55411    avgbs_55684    avgbs_68733    avgbs_70304    avgbs_93795    avgbs_235141    avgbs_235835    avgbs_203834    avgbs_204380    avgbs_240237    avgbs_12050    avgbs_26855    avgbs_33782    avgbs_47902    avgbs_54102    avgbs_56464    avgbs_58328    avgbs_60861    avgbs_81223    avgbs_96927    avgbs_96928    avgbs_106619    avgbs_201209    avgbs_237098    avgbs_206464    avgbs_8292    avgbs_11376    avgbs_11855    avgbs_41701    avgbs_52129    avgbs_74174    avgbs_74175    avgbs_92922    avgbs_94852    avgbs_94853    avgbs_117353    avgbs_117986    avgbs_117987    avgbs_201740    avgbs_207329    avgbs_15849    avgbs_27763    avgbs_27899    avgbs_39042    avgbs_40114    avgbs_44302    avgbs_44303    avgbs_53199    avgbs_57077    avgbs_69773    avgbs_81325    avgbs_81326    avgbs_89988    avgbs_94810    avgbs_116479    avgbs_116480    avgbs_233505    avgbs_205483    avgbs_205503    avgbs_240643    avgbs_244670    avgbs_10846    avgbs_23339    avgbs_50999    avgbs_62102    avgbs_68226    avgbs_69913    avgbs_69914    avgbs_73846    avgbs_75697    avgbs_90132    avgbs_238365    avgbs_6137    avgbs_32611    avgbs_40457    avgbs_45982    avgbs_47508    avgbs_53490    avgbs_75600    avgbs_97564    avgbs_106163    avgbs_106164    avgbs_202259    avgbs_238959    avgbs_2718    avgbs_27030    avgbs_29713    avgbs_31171    avgbs_53201    avgbs_235930    avgbs_2341    avgbs_26735    avgbs_58008    avgbs_79561    avgbs_235189    avgbs_236749    avgbs_17847    avgbs_236747    avgbs_44059    avgbs_44060   
   2C 45 
 
 
  avgbs_54463    avgbs_97193   
   2C 46 
 
 
  avgbs_90217    avgbs_53295    avgbs_117858    avgbs_202660    avgbs_9055    avgbs_107794    avgbs_243850    avgbs_205407   
   2C 47 
 
 
  avgbs_56268    avgbs_56269    avgbs_56270    avgbs_112051    avgbs_112052    avgbs_126240    avgbs_36121    avgbs_36122   
   2C 48 
 
 
  avgbs_243077    avgbs_216637    avgbs_28559    avgbs_28560   
   2C 50 
 
 
  avgbs_81408    avgbs_81409   
   2C 51 
 
 
  avgbs_47190   
   2C 53 
 
 
  avgbs_97889    avgbs_112528    avgbs_74327   
   2C 54 
 
 
  avgbs_4022    avgbs_92365    avgbs_115398    avgbs_115399    avgbs_52166    avgbs_36171    avgbs_36172    avgbs_103320    avgbs_103321    avgbs_39409    avgbs_112992    avgbs_46513    avgbs_29671    avgbs_29672    avgbs_202060    avgbs_121481    avgbs_38502   
   2C 55 
 
 
  avgbs_56271    avgbs_215404    avgbs_7522    avgbs_47191    avgbs_110991    avgbs_203466    avgbs_101791    avgbs_52360    avgbs_52361    avgbs_104715    avgbs_31592    avgbs_87403    avgbs_111273   
   2C 56 
 
 
  avgbs_204910    avgbs_51472    avgbs_218914    avgbs_115497    avgbs_201814    avgbs_31620    avgbs_222389    avgbs_202574    avgbs_85849   
   2C 57 
 
 
  avgbs_67437    avgbs_125180    avgbs_51473    avgbs_203632    avgbs_201830    avgbs_104638    avgbs_206246    avgbs_5107    avgbs_39325    avgbs_39326    avgbs_52592    avgbs_86295    avgbs_105924    avgbs_113835    avgbs_201258    avgbs_204369    avgbs_85850   
   2C 58 
 
 
  avgbs_109488    avgbs_53449    avgbs_53450    avgbs_108514    avgbs_108515    avgbs_13073    avgbs_13074    avgbs_14187    avgbs_70123   
   2C 59 
 
 
  avgbs_12178    avgbs_86294    avgbs_99308    avgbs_33390    avgbs_15851    avgbs_207007    avgbs_238629    avgbs_95955   
   2C 60 
 
 
  avgbs_38328    avgbs_38329    avgbs_43257    avgbs_99651    avgbs_103382    avgbs_64789    avgbs_64790    avgbs_73089   
   2C 61 
 
 
  avgbs_238807   
   2C 62 
 
 
  avgbs_207517    avgbs_207925   
 
 
   Chromosome 3C    Chr   Pos   Framework   Placed SNP  Placed GBS  
   3C -14 
 
 
  avgbs_10406   
   3C -9 
 
 
  avgbs_10912   
   3C -7 
 
 
  avgbs_119945    avgbs_216027   
   3C -6 
 
 
  avgbs_19115    avgbs_19116   
   3C -5 
 
 
  avgbs_70621    avgbs_200479   
   3C -4 
 
 
  avgbs_93965    avgbs_6K_2913   
   3C -3 
 
 
  avgbs_126111    avgbs_107275    avgbs_64749    avgbs_102724    avgbs_93549   
   3C -2 
 
 
  avgbs_7345    avgbs_75654    avgbs_24432    avgbs_50047    avgbs_50048    avgbs_214866   
   3C -1 
 
 
  avgbs_219999    avgbs_2995   
   3C 0 
  GMI_ES17_c3320_786    
  GMI_ES17_c17442_334   
  avgbs_83268    avgbs_93296    avgbs_94162    avgbs_211858    avgbs_13445    avgbs_200086    avgbs_225512    avgbs_52680    avgbs_106332    avgbs_106333    avgbs_111463    avgbs_111464    avgbs_214858    avgbs_124146    avgbs_80798    avgbs_20157    avgbs_6K_26325    avgbs_9752    avgbs_9753    avgbs_72126    avgbs_214769   
   3C 1 
 
 
  avgbs_47547    avgbs_56493    avgbs_56494    avgbs_123016    avgbs_19233    avgbs_120649    avgbs_120650    avgbs_120651    avgbs_213499    avgbs_108309   
   3C 2 
  GMI_ES02_c10749_342    
 
 
   3C 3 
 
 
  avgbs_80223    avgbs_220596    avgbs_221774    avgbs_216075    avgbs_200903    avgbs_21936    avgbs_226366   
   3C 4 
 
 
  avgbs_15850    avgbs_215312    avgbs_114084    avgbs_413    avgbs_414    avgbs_537    avgbs_71942    avgbs_71943   
   3C 5 
 
 
  avgbs_123850    avgbs_92989    avgbs_95607   
   3C 6 
 
 
  avgbs_82128    avgbs_82129   
   3C 7 
 
 
  avgbs_3785    avgbs_41918    avgbs_126020    avgbs_121971    avgbs_25104    avgbs_210634    avgbs_204611    avgbs_123368    avgbs_8950    avgbs_8951    avgbs_234369   
   3C 8 
 
 
  avgbs_42741    avgbs_65560    avgbs_24248    avgbs_68860    avgbs_202678    avgbs_219887    avgbs_121970    avgbs_18009    avgbs_47786    avgbs_115353    avgbs_118023    avgbs_232835    avgbs_207579    avgbs_17511    avgbs_64388    avgbs_44661   
   3C 9 
 
 
  avgbs_42117    avgbs_57322    avgbs_100362    avgbs_8953   
   3C 10 
 
 
  avgbs_14309    avgbs_25449    avgbs_99962    avgbs_77802    avgbs_95975    avgbs_68578    avgbs_238454    avgbs_72711    avgbs_109627   
   3C 11 
  GMI_ES01_c11827_414    
 
  avgbs_5434    avgbs_93096    avgbs_211335    avgbs_215476    avgbs_22197    avgbs_22198    avgbs_22199    avgbs_83524    avgbs_213642    avgbs_88067    avgbs_227715    avgbs_90708    avgbs_70671    avgbs_83925   
   3C 12 
  GMI_ES_CC5758_340    
 
  avgbs_5920    avgbs_82635    avgbs_89693    avgbs_204615    avgbs_26239    avgbs_9437    avgbs_20975    avgbs_82306    avgbs_90473    avgbs_121347    avgbs_202523    avgbs_215033    avgbs_15039    avgbs_36380    avgbs_36381    avgbs_201725    avgbs_215239    avgbs_215894    avgbs_32342    avgbs_72486    avgbs_77803    avgbs_124153    avgbs_227430    avgbs_81469    avgbs_46692    avgbs_46693    avgbs_14432    avgbs_90863   
   3C 13 
 
 
  avgbs_95947    avgbs_215681    avgbs_51921   
   3C 14 
  GMI_ES15_c605_638     GMI_ES01_c2827_649     GMI_ES17_c125_448    
 
  avgbs_19333    avgbs_93846    avgbs_116062    avgbs_125333    avgbs_213104    avgbs_221383    avgbs_6626    avgbs_62828    avgbs_79    avgbs_47910    avgbs_57673    avgbs_203491    avgbs_244667    avgbs_64118    avgbs_200431    avgbs_122374    avgbs_39073    avgbs_39078    avgbs_97686    avgbs_200263    avgbs_200701    avgbs_80505    avgbs_53297    avgbs_99438    avgbs_202341    avgbs_7532    avgbs_19539    avgbs_125337    avgbs_235057    avgbs_235064    avgbs_7533    avgbs_117886    avgbs_117888    avgbs_46299    avgbs_46300    avgbs_16774    avgbs_119448    avgbs_31685    avgbs_200889    avgbs_34479    avgbs_39113    avgbs_87112    avgbs_241154    avgbs_33646    avgbs_33647    avgbs_33648    avgbs_64119    avgbs_7535    avgbs_47066    avgbs_203462    avgbs_99553    avgbs_213955   
   3C 15 
 
 
  avgbs_22612    avgbs_22613    avgbs_63608    avgbs_18720    avgbs_75396    avgbs_202281   
   3C 16 
  GMI_DS_CC1149_344    
 
  avgbs_6890    avgbs_60087    avgbs_60088    avgbs_63115    avgbs_79566    avgbs_105544    avgbs_117192    avgbs_216595    avgbs_219572    avgbs_220550    avgbs_96847    avgbs_221740    avgbs_208209    avgbs_106414    avgbs_88738    avgbs_55223    avgbs_65156    avgbs_98601   
   3C 17 
 
 
  avgbs_74452    avgbs_84785    avgbs_94811    avgbs_215113   
   3C 18 
  GMI_DS_CC10993_70    
 
  avgbs_14533    avgbs_213388   
   3C 19 
  GMI_ES_CC15057_51     GMI_DS_CC5134_229     GMI_ES01_c461_1288    
 
  avgbs_4547    avgbs_13155    avgbs_21933    avgbs_23535    avgbs_25530    avgbs_26532    avgbs_40231    avgbs_44846    avgbs_47595    avgbs_56588    avgbs_62600    avgbs_63711    avgbs_65537    avgbs_67582    avgbs_72442    avgbs_72443    avgbs_75886    avgbs_76940    avgbs_77096    avgbs_77564    avgbs_84298    avgbs_84299    avgbs_84665    avgbs_95163    avgbs_113206    avgbs_118754    avgbs_123763    avgbs_200279    avgbs_218444    avgbs_200295    avgbs_219197    avgbs_200422    avgbs_200448    avgbs_220331    avgbs_200524    avgbs_221361    avgbs_221530    avgbs_221546    avgbs_200688    avgbs_200860    avgbs_59    avgbs_60    avgbs_760    avgbs_1287    avgbs_1919    avgbs_2151    avgbs_2281    avgbs_2287    avgbs_2721    avgbs_2722    avgbs_3565    avgbs_6746    avgbs_13894    avgbs_18266    avgbs_24307    avgbs_24308    avgbs_26961    avgbs_28453    avgbs_28454    avgbs_29931    avgbs_30778    avgbs_30819    avgbs_34619    avgbs_36177    avgbs_36582    avgbs_36696    avgbs_36758    avgbs_37588    avgbs_38692    avgbs_40028    avgbs_40246    avgbs_41395    avgbs_42736    avgbs_46139    avgbs_47747    avgbs_50499    avgbs_51546    avgbs_52363    avgbs_52426    avgbs_59243    avgbs_59244    avgbs_59267    avgbs_62168    avgbs_64712    avgbs_66663    avgbs_69811    avgbs_70620    avgbs_71873    avgbs_73109    avgbs_74414    avgbs_80826    avgbs_82434    avgbs_83551    avgbs_86863    avgbs_87399    avgbs_88226    avgbs_89741    avgbs_89810    avgbs_92720    avgbs_93032    avgbs_93033    avgbs_93034    avgbs_96248    avgbs_100897    avgbs_101587    avgbs_103043    avgbs_109534    avgbs_109776    avgbs_114082    avgbs_115947    avgbs_118827    avgbs_121335    avgbs_125553    avgbs_208509    avgbs_200010    avgbs_216735    avgbs_216762    avgbs_216863    avgbs_217100    avgbs_200148    avgbs_200226    avgbs_200244    avgbs_218211    avgbs_218383    avgbs_218471    avgbs_200330    avgbs_214271    avgbs_200384    avgbs_200410    avgbs_200487    avgbs_220232    avgbs_220294    avgbs_200571    avgbs_220986    avgbs_220988    avgbs_200684    avgbs_200712    avgbs_200803    avgbs_200837    avgbs_200856    avgbs_200937    avgbs_92634    avgbs_109874    avgbs_112193    avgbs_5671    avgbs_22158    avgbs_26833    avgbs_36209    avgbs_36210    avgbs_42572    avgbs_42573    avgbs_45417    avgbs_56280    avgbs_72528    avgbs_78432    avgbs_78433    avgbs_86017    avgbs_91430    avgbs_101467    avgbs_104529    avgbs_104530    avgbs_121819    avgbs_209435    avgbs_219573    avgbs_222504    avgbs_9062    avgbs_112581    avgbs_216733    avgbs_7267    avgbs_9748    avgbs_13187    avgbs_22789    avgbs_26951    avgbs_29008    avgbs_29009    avgbs_30385    avgbs_30387    avgbs_36666    avgbs_38404    avgbs_38405    avgbs_42222    avgbs_43826    avgbs_43827    avgbs_43828    avgbs_54601    avgbs_56999    avgbs_57000    avgbs_59115    avgbs_59716    avgbs_59717    avgbs_59718    avgbs_63844    avgbs_67440    avgbs_74655    avgbs_78453    avgbs_81158    avgbs_84578    avgbs_86785    avgbs_86786    avgbs_87146    avgbs_87791    avgbs_93141    avgbs_93142    avgbs_97380    avgbs_98677    avgbs_101804    avgbs_101805    avgbs_102477    avgbs_105559    avgbs_108478    avgbs_110202    avgbs_123808    avgbs_209514    avgbs_209958    avgbs_210674    avgbs_220241    avgbs_200780    avgbs_212400    avgbs_38781    avgbs_38782    avgbs_57863    avgbs_75612    avgbs_91948    avgbs_219068    avgbs_50527    avgbs_52475    avgbs_53777    avgbs_71564    avgbs_74618    avgbs_84305    avgbs_93058    avgbs_96164    avgbs_68810    avgbs_115337    avgbs_123075    avgbs_206159    avgbs_36598    avgbs_63064    avgbs_63331    avgbs_110648    avgbs_219584    avgbs_224517    avgbs_6360    avgbs_202034    avgbs_63651    avgbs_117186    avgbs_35465    avgbs_209064    avgbs_53889    avgbs_93082    avgbs_200652    avgbs_225516    avgbs_222519    avgbs_2306    avgbs_18100    avgbs_21946    avgbs_24985    avgbs_34616    avgbs_34617    avgbs_42646    avgbs_47578    avgbs_54604    avgbs_60145    avgbs_64248    avgbs_64249    avgbs_68354    avgbs_68355    avgbs_68368    avgbs_70644    avgbs_80029    avgbs_80030    avgbs_99551    avgbs_100210    avgbs_106037    avgbs_109642    avgbs_113247    avgbs_113248    avgbs_113249    avgbs_114653    avgbs_115378    avgbs_116663    avgbs_118958    avgbs_119673    avgbs_121312    avgbs_122051    avgbs_124983    avgbs_213074    avgbs_223264    avgbs_224047    avgbs_200313    avgbs_224212    avgbs_219397    avgbs_210732    avgbs_200653    avgbs_200769    avgbs_225523    avgbs_225649    avgbs_222836    avgbs_200906    avgbs_1041    avgbs_7689    avgbs_29742    avgbs_36609    avgbs_38740    avgbs_42671    avgbs_43805    avgbs_46287    avgbs_50824    avgbs_71086    avgbs_86603    avgbs_92041    avgbs_113604    avgbs_113704    avgbs_114175    avgbs_125452    avgbs_125453    avgbs_209877    avgbs_211184    avgbs_200639    avgbs_749    avgbs_750    avgbs_8356    avgbs_8357    avgbs_20984    avgbs_51355    avgbs_51356    avgbs_56993    avgbs_67642    avgbs_69511    avgbs_72591    avgbs_96109    avgbs_209441    avgbs_210378    avgbs_224472    avgbs_219826    avgbs_6K_84241    avgbs_112835    avgbs_215839    avgbs_36697    avgbs_1040    avgbs_71361    avgbs_61682    avgbs_35020    avgbs_70798    avgbs_76926    avgbs_213894    avgbs_50337    avgbs_54006    avgbs_46472    avgbs_214130    avgbs_119968    avgbs_213121    avgbs_216000    avgbs_1626    avgbs_3752    avgbs_11558    avgbs_27373    avgbs_27894    avgbs_36317    avgbs_55006    avgbs_64713    avgbs_76161    avgbs_76542    avgbs_91765    avgbs_123354    avgbs_125556    avgbs_213057    avgbs_213107    avgbs_213308    avgbs_213707    avgbs_214209    avgbs_215081    avgbs_215182    avgbs_215415    avgbs_215689    avgbs_1096    avgbs_25031    avgbs_36165    avgbs_41092    avgbs_44730    avgbs_50560    avgbs_70799    avgbs_70800    avgbs_76012    avgbs_78230    avgbs_92539    avgbs_94089    avgbs_94164    avgbs_109242    avgbs_112730    avgbs_213628    avgbs_215664    avgbs_212640    avgbs_215940    avgbs_216037    avgbs_75703    avgbs_75704    avgbs_9070    avgbs_23925    avgbs_36898    avgbs_36900    avgbs_43175    avgbs_43239    avgbs_52993    avgbs_62508    avgbs_76075    avgbs_89809    avgbs_106682    avgbs_116469    avgbs_120868    avgbs_213752    avgbs_214110    avgbs_214878    avgbs_10833    avgbs_21909    avgbs_125129    avgbs_213288    avgbs_213797    avgbs_7444    avgbs_53136    avgbs_53137    avgbs_99979    avgbs_99980    avgbs_74381    avgbs_214833   
   3C 20 
  BA_grs_c8269_158     GMI_ES_CC3491_422     GMI_ES17_c2398_610     GMI_ES17_c10984_690    
 
  avgbs_61    avgbs_33899    avgbs_41705    avgbs_41706    avgbs_49010    avgbs_51352    avgbs_72066    avgbs_96767    avgbs_96768    avgbs_200035    avgbs_217122    avgbs_1155    avgbs_5508    avgbs_21410    avgbs_30767    avgbs_33678    avgbs_36855    avgbs_38882    avgbs_41451    avgbs_46835    avgbs_56050    avgbs_61362    avgbs_61363    avgbs_61663    avgbs_66583    avgbs_74116    avgbs_90401    avgbs_98089    avgbs_100651    avgbs_100838    avgbs_102221    avgbs_106211    avgbs_114402    avgbs_116693    avgbs_200027    avgbs_217909    avgbs_209932    avgbs_200373    avgbs_200386    avgbs_220501    avgbs_221187    avgbs_221871    avgbs_39690    avgbs_39691    avgbs_39692    avgbs_39693    avgbs_11106    avgbs_28290    avgbs_37778    avgbs_41297    avgbs_42729    avgbs_42867    avgbs_44100    avgbs_112853    avgbs_122024    avgbs_221718    avgbs_222957    avgbs_40455    avgbs_89223    avgbs_32296    avgbs_37611    avgbs_40227    avgbs_40229    avgbs_44677    avgbs_47856    avgbs_52887    avgbs_53154    avgbs_53156    avgbs_53988    avgbs_57323    avgbs_57324    avgbs_57325    avgbs_62952    avgbs_72225    avgbs_89895    avgbs_95560    avgbs_102770    avgbs_104101    avgbs_104102    avgbs_218986    avgbs_211843    avgbs_222417    avgbs_37336    avgbs_4115    avgbs_68735    avgbs_68736    avgbs_118749    avgbs_208631    avgbs_222781    avgbs_102003    avgbs_212167    avgbs_46495    avgbs_77276    avgbs_77277    avgbs_224444    avgbs_13227    avgbs_49011    avgbs_88516    avgbs_92204    avgbs_110481    avgbs_110482    avgbs_122105    avgbs_223827    avgbs_219811    avgbs_200641    avgbs_225770    avgbs_5469    avgbs_6K_69655    avgbs_90403    avgbs_120597    avgbs_121839    avgbs_123813    avgbs_223302    avgbs_224481    avgbs_23393    avgbs_86308    avgbs_90396    avgbs_90397    avgbs_24425   
   3C 21 
  GMI_ES02_c13608_538     GMI_ES15_c1806_90     GMI_ES17_c5784_752    
 
  avgbs_203420    avgbs_17635    avgbs_25847    avgbs_74532    avgbs_93582    avgbs_99294    avgbs_107605    avgbs_36192    avgbs_203062    avgbs_220384    avgbs_87098    avgbs_52886    avgbs_59619    avgbs_99663    avgbs_83097    avgbs_6089    avgbs_10027    avgbs_10028    avgbs_14208    avgbs_16513    avgbs_20738    avgbs_20739    avgbs_20740    avgbs_22666    avgbs_25727    avgbs_25730    avgbs_26846    avgbs_30728    avgbs_32200    avgbs_33815    avgbs_35095    avgbs_36831    avgbs_39211    avgbs_40282    avgbs_45822    avgbs_62458    avgbs_73024    avgbs_88861    avgbs_99840    avgbs_99841    avgbs_109532    avgbs_123215    avgbs_226615    avgbs_36106    avgbs_42937    avgbs_117258    avgbs_47833    avgbs_227242    avgbs_9910    avgbs_10051    avgbs_10052    avgbs_15913    avgbs_17200    avgbs_17201    avgbs_21350    avgbs_25893    avgbs_29805    avgbs_42188    avgbs_48966    avgbs_60090    avgbs_60985    avgbs_68163    avgbs_71246    avgbs_78956    avgbs_78957    avgbs_81146    avgbs_81147    avgbs_85963    avgbs_88739    avgbs_91128    avgbs_101672    avgbs_104397    avgbs_106474    avgbs_106475    avgbs_109021    avgbs_112940    avgbs_115286    avgbs_116390    avgbs_120646    avgbs_120744    avgbs_120779    avgbs_125601    avgbs_125803    avgbs_126227    avgbs_126228    avgbs_237875    avgbs_204716    avgbs_205762    avgbs_241421    avgbs_206845    avgbs_29211    avgbs_65313    avgbs_78733    avgbs_79684    avgbs_105227    avgbs_110495    avgbs_123849    avgbs_206753    avgbs_103451    avgbs_112082    avgbs_202077    avgbs_202361    avgbs_202410    avgbs_205173    avgbs_206174    avgbs_47769    avgbs_52683    avgbs_52686    avgbs_52888    avgbs_53946    avgbs_74622    avgbs_203754    avgbs_6066    avgbs_19397    avgbs_88307    avgbs_93175    avgbs_95771    avgbs_114959    avgbs_122180    avgbs_122181    avgbs_201956    avgbs_205544    avgbs_207012    avgbs_10383    avgbs_33296    avgbs_66487    avgbs_16175    avgbs_16176    avgbs_29210    avgbs_35974    avgbs_58671    avgbs_66591    avgbs_91925    avgbs_125217    avgbs_227215    avgbs_225773    avgbs_56379    avgbs_60429    avgbs_60430    avgbs_4136    avgbs_26840    avgbs_36255    avgbs_36256    avgbs_45938    avgbs_54393    avgbs_58254    avgbs_60496    avgbs_60501    avgbs_67430    avgbs_74526    avgbs_84197    avgbs_84199    avgbs_124389    avgbs_203777    avgbs_219421    avgbs_204578    avgbs_208245    avgbs_2562    avgbs_51343    avgbs_106747    avgbs_103458    avgbs_90794    avgbs_93183    avgbs_3411    avgbs_236832    avgbs_80632    avgbs_92298    avgbs_82352    avgbs_100677    avgbs_240532    avgbs_58810    avgbs_58812    avgbs_69597    avgbs_61939    avgbs_103026    avgbs_203020    avgbs_24694    avgbs_113822    avgbs_226417    avgbs_204996    avgbs_58808    avgbs_58811    avgbs_58819    avgbs_105207    avgbs_204855    avgbs_240224    avgbs_70555    avgbs_236192    avgbs_346    avgbs_347    avgbs_36436    avgbs_201997    avgbs_235161    avgbs_235850    avgbs_59219    avgbs_210485    avgbs_39026    avgbs_28937    avgbs_28938    avgbs_66124   
   3C 22 
  GMI_DS_CC8554_381    
 
  avgbs_63492    avgbs_63935    avgbs_74899    avgbs_27632    avgbs_32179    avgbs_32180    avgbs_34580    avgbs_61506    avgbs_36731    avgbs_50215    avgbs_88324    avgbs_91654    avgbs_4844    avgbs_35975    avgbs_52590    avgbs_67466    avgbs_115118    avgbs_200996    avgbs_201248    avgbs_206513    avgbs_56302    avgbs_81154    avgbs_100685    avgbs_101024    avgbs_106132    avgbs_54886    avgbs_95657    avgbs_220481    avgbs_241934    avgbs_123632    avgbs_203550    avgbs_119360    avgbs_124969    avgbs_202984    avgbs_240243    avgbs_65829    avgbs_204190    avgbs_24806    avgbs_33817    avgbs_63811    avgbs_79167    avgbs_98772    avgbs_64101    avgbs_202476    avgbs_6554    avgbs_22348    avgbs_72734    avgbs_76455    avgbs_4604    avgbs_240600    avgbs_45220    avgbs_45714    avgbs_202559    avgbs_57105    avgbs_81560    avgbs_236452    avgbs_113414    avgbs_62594   
   3C 23 
 
 
  avgbs_222253    avgbs_88558    avgbs_110483    avgbs_115070    avgbs_218908    avgbs_222370    avgbs_200846    avgbs_120525    avgbs_45572    avgbs_35576    avgbs_35577    avgbs_51777    avgbs_55678    avgbs_74877    avgbs_76834    avgbs_90724    avgbs_90725    avgbs_96762    avgbs_104996    avgbs_203041    avgbs_30054    avgbs_61507    avgbs_76736    avgbs_107706    avgbs_11637    avgbs_11638    avgbs_18016    avgbs_50888    avgbs_55677    avgbs_111717    avgbs_124354    avgbs_124355    avgbs_201175    avgbs_202946    avgbs_203585    avgbs_204170    avgbs_204442    avgbs_205819    avgbs_58833    avgbs_120494    avgbs_45573    avgbs_4113    avgbs_233282    avgbs_63618    avgbs_107366    avgbs_79028    avgbs_45219    avgbs_233671    avgbs_40782    avgbs_47919    avgbs_36435    avgbs_92467    avgbs_205621    avgbs_50737    avgbs_5362    avgbs_5861    avgbs_9007    avgbs_41392    avgbs_39681    avgbs_207092   
   3C 24 
  GMI_DS_CC5751_111    
 
  avgbs_90140    avgbs_221157    avgbs_122385    avgbs_88468    avgbs_88469   
   3C 25 
 
 
  avgbs_37138    avgbs_37139   
   3C 26 
  GMI_ES01_c23001_372     GMI_ES_CC8805_161     GMI_ES01_c10120_303    
 
  avgbs_58562    avgbs_58563    avgbs_7666    avgbs_14004    avgbs_85583    avgbs_90402    avgbs_125066    avgbs_216639    avgbs_36610    avgbs_36611    avgbs_36612    avgbs_46871    avgbs_30030    avgbs_30031    avgbs_58652    avgbs_60696    avgbs_60697    avgbs_25841    avgbs_219413    avgbs_203606    avgbs_6474    avgbs_6475    avgbs_111219    avgbs_7430    avgbs_9566    avgbs_11491    avgbs_16680    avgbs_20932    avgbs_23394    avgbs_23395    avgbs_32208    avgbs_34089    avgbs_35242    avgbs_35341    avgbs_44405    avgbs_48230    avgbs_52011    avgbs_52072    avgbs_52073    avgbs_52074    avgbs_52080    avgbs_52499    avgbs_55268    avgbs_76917    avgbs_77273    avgbs_77274    avgbs_77406    avgbs_77944    avgbs_78892    avgbs_78893    avgbs_87838    avgbs_91113    avgbs_92915    avgbs_96737    avgbs_100830    avgbs_100922    avgbs_120766    avgbs_125622    avgbs_125623    avgbs_236079    avgbs_205410    avgbs_240925    avgbs_15772    avgbs_38288    avgbs_52843    avgbs_59218    avgbs_62169    avgbs_112087    avgbs_112088    avgbs_125497    avgbs_239413    avgbs_206587    avgbs_936    avgbs_1405    avgbs_3175    avgbs_4241    avgbs_6886    avgbs_7231    avgbs_8185    avgbs_8186    avgbs_8321    avgbs_9995    avgbs_10416    avgbs_10932    avgbs_12086    avgbs_12087    avgbs_16070    avgbs_16071    avgbs_17834    avgbs_21277    avgbs_23199    avgbs_23798    avgbs_23799    avgbs_24469    avgbs_24470    avgbs_25346    avgbs_25413    avgbs_30097    avgbs_31193    avgbs_36431    avgbs_38533    avgbs_39196    avgbs_41304    avgbs_41305    avgbs_41944    avgbs_44406    avgbs_47102    avgbs_47256    avgbs_50905    avgbs_50906    avgbs_51644    avgbs_59086    avgbs_61866    avgbs_63525    avgbs_63912    avgbs_66632    avgbs_66953    avgbs_66976    avgbs_66977    avgbs_67855    avgbs_70223    avgbs_71597    avgbs_71598    avgbs_71599    avgbs_72463    avgbs_74967    avgbs_74968    avgbs_74969    avgbs_76512    avgbs_76993    avgbs_77855    avgbs_78845    avgbs_78846    avgbs_78847    avgbs_80838    avgbs_81020    avgbs_81021    avgbs_81022    avgbs_86049    avgbs_86657    avgbs_90239    avgbs_91672    avgbs_91834    avgbs_92536    avgbs_94297    avgbs_96506    avgbs_99835    avgbs_105134    avgbs_105135    avgbs_107363    avgbs_107382    avgbs_110943    avgbs_110944    avgbs_111334    avgbs_113760    avgbs_114949    avgbs_114950    avgbs_114951    avgbs_119376    avgbs_120097    avgbs_120098    avgbs_120099    avgbs_121402    avgbs_121439    avgbs_122083    avgbs_122608    avgbs_122609    avgbs_124347    avgbs_201214    avgbs_201500    avgbs_201708    avgbs_202555    avgbs_204233    avgbs_204277    avgbs_204376    avgbs_205063    avgbs_85067    avgbs_205332    avgbs_205573    avgbs_205792    avgbs_205964    avgbs_205974    avgbs_242329    avgbs_206703    avgbs_206888    avgbs_207223    avgbs_208002    avgbs_208269    avgbs_11569    avgbs_34621    avgbs_35647    avgbs_35648    avgbs_56043    avgbs_68472    avgbs_89921    avgbs_116791    avgbs_121417    avgbs_202969    avgbs_202988    avgbs_27849    avgbs_37672    avgbs_40970    avgbs_40971    avgbs_74966    avgbs_78118    avgbs_78119    avgbs_78848    avgbs_92294    avgbs_93808    avgbs_111546    avgbs_111547    avgbs_122847    avgbs_125045    avgbs_207774    avgbs_12568    avgbs_12569    avgbs_21527    avgbs_34283    avgbs_43641    avgbs_58262    avgbs_72022    avgbs_72023    avgbs_78237    avgbs_105600    avgbs_237696    avgbs_208386    avgbs_8862    avgbs_36730    avgbs_41501    avgbs_41502    avgbs_50205    avgbs_67479    avgbs_67765    avgbs_112283    avgbs_122583    avgbs_201417    avgbs_235244    avgbs_236644    avgbs_25299    avgbs_30903    avgbs_30904    avgbs_51999    avgbs_116856    avgbs_172    avgbs_173    avgbs_25157    avgbs_25158    avgbs_41660    avgbs_43686    avgbs_67199    avgbs_80070    avgbs_95587    avgbs_101582    avgbs_109309    avgbs_122606    avgbs_122607    avgbs_237772    avgbs_204933    avgbs_2638    avgbs_93398    avgbs_108072    avgbs_205470    avgbs_35592    avgbs_49655    avgbs_50171    avgbs_113997    avgbs_117443    avgbs_204294    avgbs_207075    avgbs_2600    avgbs_11839    avgbs_39376    avgbs_69634    avgbs_80615    avgbs_86074    avgbs_91801    avgbs_106154    avgbs_46515    avgbs_58679    avgbs_74723    avgbs_90610    avgbs_95463    avgbs_205230    avgbs_207376    avgbs_18459    avgbs_92578    avgbs_11299    avgbs_62863    avgbs_66914    avgbs_84819    avgbs_84820    avgbs_114224    avgbs_80203    avgbs_94864    avgbs_234456    avgbs_119119    avgbs_6560    avgbs_29934    avgbs_43471    avgbs_45604    avgbs_64695    avgbs_5765    avgbs_5766    avgbs_29407    avgbs_54322    avgbs_120400    avgbs_237431    avgbs_17078    avgbs_32545    avgbs_59247    avgbs_242892    avgbs_244369    avgbs_49269    avgbs_210166    avgbs_215343   
   3C 28 
  GMI_ES_CC14261_141     GMI_ES01_c11741_182    
 
  avgbs_83544    avgbs_218803    avgbs_13696    avgbs_14002    avgbs_14003    avgbs_49670    avgbs_58639    avgbs_114206    avgbs_126109    avgbs_8691    avgbs_8692    avgbs_11295    avgbs_12084    avgbs_14190    avgbs_26668    avgbs_58580    avgbs_62170    avgbs_66827    avgbs_88275    avgbs_88276    avgbs_106374    avgbs_217690    avgbs_217814    avgbs_219316    avgbs_219537    avgbs_220811    avgbs_200835    avgbs_66450    avgbs_86637    avgbs_93564    avgbs_117089    avgbs_219150    avgbs_222763    avgbs_3825    avgbs_13947    avgbs_32219    avgbs_33394    avgbs_33395    avgbs_35965    avgbs_43373    avgbs_67391    avgbs_68196    avgbs_124755    avgbs_223104    avgbs_15833    avgbs_18049    avgbs_25111    avgbs_40294    avgbs_54104    avgbs_119333   
   3C 29 
 
 
  avgbs_49238    avgbs_49239   
   3C 30 
 
 
  avgbs_16438    avgbs_219602   
   3C 31 
 
 
  avgbs_219064   
   3C 32 
  GMI_ES01_c109_982    
 
  avgbs_7961    avgbs_94702    avgbs_92078    avgbs_21034    avgbs_93676    avgbs_93677    avgbs_83356    avgbs_224406   
   3C 33 
 
 
  avgbs_12172   
   3C 34 
 
 
  avgbs_27354    avgbs_217720    avgbs_40308    avgbs_72279    avgbs_72280    avgbs_86473    avgbs_72260    avgbs_220122    avgbs_51632    avgbs_90209    avgbs_216434    avgbs_200337    avgbs_40611    avgbs_77601    avgbs_77568    avgbs_26385    avgbs_28735   
   3C 35 
 
 
  avgbs_86695    avgbs_21750    avgbs_21751    avgbs_87652    avgbs_106863    avgbs_106864    avgbs_95670    avgbs_95671    avgbs_35508    avgbs_94562    avgbs_94564    avgbs_96515    avgbs_218675    avgbs_29284    avgbs_38641    avgbs_105257    avgbs_227172    avgbs_64505    avgbs_201698    avgbs_72993   
   3C 36 
 
 
  avgbs_200333    avgbs_43298    avgbs_43299    avgbs_72900    avgbs_28502    avgbs_231788    avgbs_67032   
   3C 37 
 
 
  avgbs_72901    avgbs_231002    avgbs_87110    avgbs_225620   
   3C 38 
 
 
  avgbs_24815    avgbs_24816    avgbs_20626    avgbs_225532    avgbs_44775    avgbs_11203    avgbs_118413    avgbs_23072   
   3C 39 
 
 
  avgbs_46488    avgbs_200308    avgbs_20362    avgbs_79550    avgbs_200554    avgbs_47872    avgbs_117426   
   3C 40 
  GMI_ES15_c6870_218    
 
  avgbs_1679    avgbs_216283    avgbs_10199    avgbs_20595    avgbs_47590    avgbs_119680    avgbs_216802    avgbs_104951    avgbs_4620    avgbs_88224    avgbs_10149    avgbs_25849    avgbs_20594    avgbs_22385    avgbs_22386    avgbs_32853    avgbs_50028    avgbs_52681    avgbs_100961    avgbs_112713    avgbs_117427    avgbs_117428    avgbs_126095    avgbs_232167    avgbs_232397    avgbs_232521    avgbs_232809    avgbs_48514    avgbs_226832    avgbs_230387    avgbs_232160    avgbs_17007    avgbs_210141    avgbs_17008    avgbs_45934    avgbs_45936    avgbs_229243    avgbs_48550    avgbs_19585    avgbs_223596    avgbs_25090    avgbs_26730    avgbs_26734    avgbs_2660    avgbs_18804    avgbs_65717    avgbs_69172    avgbs_229974    avgbs_120630    avgbs_109902    avgbs_109903    avgbs_95007    avgbs_77779    avgbs_5321   
   3C 41 
 
 
  avgbs_8360    avgbs_51087    avgbs_216683    avgbs_200118    avgbs_10058    avgbs_31483    avgbs_48322    avgbs_49758    avgbs_54427    avgbs_54428    avgbs_54429    avgbs_67055    avgbs_74465    avgbs_82237    avgbs_123266    avgbs_216993    avgbs_217521    avgbs_217940    avgbs_222582    avgbs_223029    avgbs_220049    avgbs_220961    avgbs_43623    avgbs_107662    avgbs_209991    avgbs_81835    avgbs_72171   
   3C 42 
 
 
  avgbs_19673    avgbs_75416    avgbs_75417    avgbs_10146    avgbs_19324    avgbs_21323    avgbs_28257    avgbs_37856    avgbs_37857    avgbs_55208    avgbs_78246    avgbs_217357    avgbs_217455    avgbs_200187    avgbs_218452    avgbs_13839    avgbs_84946    avgbs_104410    avgbs_217378    avgbs_16455    avgbs_16456    avgbs_38586    avgbs_67294    avgbs_80677    avgbs_33126   
   3C 43 
 
 
  avgbs_88609    avgbs_116923   
   3C 44 
  GMI_ES02_c1819_259    
 
  avgbs_243503    avgbs_76321    avgbs_239981    avgbs_244581    avgbs_28503    avgbs_98174    avgbs_102354    avgbs_225530   
   3C 45 
 
 
  avgbs_29058    avgbs_114097    avgbs_115454   
   3C 46 
 
 
  avgbs_115104    avgbs_60013    avgbs_63042    avgbs_78003    avgbs_105550    avgbs_228680    avgbs_230574    avgbs_75768    avgbs_231111    avgbs_240006    avgbs_223517    avgbs_105258    avgbs_19737    avgbs_223603    avgbs_224988   
   3C 47 
 
 
  avgbs_101258    avgbs_221902    avgbs_61400    avgbs_61401    avgbs_61402    avgbs_15082    avgbs_11359    avgbs_43318    avgbs_67607    avgbs_109505    avgbs_109506    avgbs_17250    avgbs_62730    avgbs_54363    avgbs_224302    avgbs_49928    avgbs_83966    avgbs_224984    avgbs_240890    avgbs_16576    avgbs_121745    avgbs_244040    avgbs_67608    avgbs_84885    avgbs_84886    avgbs_84887    avgbs_26590    avgbs_6K_23770    avgbs_34080    avgbs_34081    avgbs_223523    avgbs_123117   
   3C 48 
 
 
  avgbs_81242    avgbs_106559    avgbs_106560    avgbs_37903    avgbs_125361    avgbs_200257    avgbs_16739    avgbs_16740    avgbs_243202    avgbs_243525    avgbs_11698    avgbs_76571    avgbs_224802    avgbs_86702    avgbs_225036    avgbs_118122    avgbs_118123    avgbs_118124    avgbs_21503    avgbs_223297    avgbs_113724    avgbs_225683   
   3C 49 
 
 
  avgbs_26464    avgbs_117494   
   3C 50 
  GMI_ES01_c20367_331    
 
  avgbs_122386    avgbs_121423    avgbs_119170    avgbs_119171    avgbs_90984    avgbs_90985    avgbs_242231    avgbs_59932    avgbs_48538    avgbs_242599    avgbs_53351   
   3C 51 
 
 
  avgbs_218473    avgbs_37902    avgbs_37904    avgbs_37906    avgbs_68781    avgbs_116287    avgbs_216881    avgbs_125668    avgbs_124647    avgbs_124648    avgbs_27164    avgbs_27165    avgbs_107395    avgbs_107396    avgbs_6K_102428   
   3C 52 
  GMI_ES01_lrc22461_468    
 
  avgbs_221695    avgbs_1380   
   3C 53 
  GMI_ES_CC8697_53    
 
  avgbs_1135    avgbs_8661    avgbs_21508    avgbs_21509    avgbs_64300    avgbs_77763    avgbs_93924    avgbs_111202    avgbs_111203    avgbs_114258    avgbs_122080    avgbs_122081    avgbs_221393    avgbs_222556    avgbs_628    avgbs_629    avgbs_4316    avgbs_4317    avgbs_4563    avgbs_8565    avgbs_17902    avgbs_23886    avgbs_38213    avgbs_45930    avgbs_46936    avgbs_52845    avgbs_57027    avgbs_60615    avgbs_65191    avgbs_65192    avgbs_65193    avgbs_67861    avgbs_67885    avgbs_69488    avgbs_74719    avgbs_78002    avgbs_78004    avgbs_79808    avgbs_79829    avgbs_81623    avgbs_82699    avgbs_82700    avgbs_98096    avgbs_98097    avgbs_6K_101811    avgbs_110374    avgbs_114419    avgbs_114420    avgbs_114575    avgbs_114576    avgbs_116769    avgbs_116770    avgbs_118280    avgbs_213041    avgbs_216362    avgbs_217188    avgbs_218016    avgbs_218620    avgbs_218681    avgbs_218842    avgbs_218976    avgbs_219144    avgbs_219224    avgbs_220247    avgbs_220320    avgbs_222916    avgbs_25150    avgbs_38501    avgbs_49876    avgbs_56236    avgbs_57542    avgbs_57543    avgbs_79469    avgbs_218834    avgbs_221190    avgbs_123860    avgbs_200924    avgbs_214787    avgbs_26718    avgbs_101298    avgbs_15976    avgbs_222959    avgbs_69239    avgbs_221164    avgbs_221196    avgbs_96978    avgbs_28416    avgbs_28417    avgbs_83285    avgbs_124128    avgbs_232727    avgbs_119436    avgbs_73143    avgbs_237564    avgbs_237043    avgbs_93500    avgbs_11182    avgbs_11183    avgbs_22144    avgbs_213461    avgbs_57360    avgbs_63218    avgbs_79807    avgbs_89201    avgbs_126039    avgbs_6986    avgbs_233653    avgbs_45890    avgbs_92963   
   3C 54 
  GMI_ES17_c16539_472    
 
  avgbs_37664    avgbs_218237    avgbs_1148    avgbs_12199    avgbs_22694    avgbs_22695    avgbs_41969    avgbs_62325    avgbs_87425    avgbs_94313    avgbs_94768    avgbs_105744    avgbs_218244    avgbs_200277    avgbs_218469    avgbs_219209    avgbs_219212    avgbs_219541    avgbs_220103    avgbs_221430    avgbs_10274    avgbs_25851    avgbs_25852    avgbs_42954    avgbs_49799    avgbs_51024    avgbs_94974    avgbs_95992    avgbs_216816    avgbs_65659    avgbs_21737    avgbs_37865    avgbs_49842    avgbs_49843    avgbs_49844    avgbs_91543    avgbs_99796    avgbs_110270    avgbs_218246    avgbs_46057    avgbs_46058    avgbs_19738    avgbs_86910    avgbs_111759    avgbs_221141    avgbs_222757    avgbs_1756    avgbs_55462    avgbs_219127    avgbs_62896    avgbs_105981    avgbs_107406    avgbs_220192    avgbs_31708    avgbs_209923    avgbs_84562    avgbs_105633    avgbs_216996    avgbs_12170    avgbs_38013    avgbs_89196    avgbs_96444    avgbs_117959    avgbs_24060    avgbs_118652    avgbs_86957    avgbs_53291    avgbs_224271    avgbs_77253   
   3C 55 
  GMI_ES17_lrc19617_111     GMI_DS_CC1800_254    
 
  avgbs_48364    avgbs_55110    avgbs_55111    avgbs_55112    avgbs_6K_58282    avgbs_77020    avgbs_77021    avgbs_77766    avgbs_77767    avgbs_95308    avgbs_122014    avgbs_122942    avgbs_219510    avgbs_1409    avgbs_3588    avgbs_7442    avgbs_19310    avgbs_19311    avgbs_21976    avgbs_55467    avgbs_91821    avgbs_216773    avgbs_217583    avgbs_221254    avgbs_97189    avgbs_8049    avgbs_40583    avgbs_122375    avgbs_60863    avgbs_101249    avgbs_205320    avgbs_241390    avgbs_3981    avgbs_3982    avgbs_13007    avgbs_13008    avgbs_63896    avgbs_94710    avgbs_98299    avgbs_233245    avgbs_215419    avgbs_93138    avgbs_240201    avgbs_243825    avgbs_120071    avgbs_39903    avgbs_39904    avgbs_18055    avgbs_26647    avgbs_60614    avgbs_100674    avgbs_237854    avgbs_241346    avgbs_234192    avgbs_41418    avgbs_67375    avgbs_236612   
   3C 56 
 
 
  avgbs_10939    avgbs_29346    avgbs_66291    avgbs_106255    avgbs_116873    avgbs_116874    avgbs_216564    avgbs_217843    avgbs_221291    avgbs_81766    avgbs_22627    avgbs_45424    avgbs_26269    avgbs_202750    avgbs_95985    avgbs_79668    avgbs_239311    avgbs_79667    avgbs_100099    avgbs_122389    avgbs_122390   
   3C 57 
  GMI_ES17_c4051_315    
 
  avgbs_19758    avgbs_21833    avgbs_21834    avgbs_42921    avgbs_42922    avgbs_42923    avgbs_45507    avgbs_59312    avgbs_62154    avgbs_86552    avgbs_87590    avgbs_90637    avgbs_90638    avgbs_95428    avgbs_102662    avgbs_220946    avgbs_215395    avgbs_7796    avgbs_40307    avgbs_84836    avgbs_93744    avgbs_58323    avgbs_119802    avgbs_123627    avgbs_123628    avgbs_7166    avgbs_7167    avgbs_7168    avgbs_95970    avgbs_95971    avgbs_8317    avgbs_44891    avgbs_13157    avgbs_77252    avgbs_77470    avgbs_95286    avgbs_231485    avgbs_14582    avgbs_55129    avgbs_229184    avgbs_229464    avgbs_37957    avgbs_37958    avgbs_34107    avgbs_31522    avgbs_91454    avgbs_109843    avgbs_104679    avgbs_100262    avgbs_241316    avgbs_206439    avgbs_63895    avgbs_70818   
   3C 58 
 
 
  avgbs_30837    avgbs_34007    avgbs_34280    avgbs_48155    avgbs_48156    avgbs_95307    avgbs_95309    avgbs_96484    avgbs_98229    avgbs_100596    avgbs_111300    avgbs_221733    avgbs_222406    avgbs_112554    avgbs_218406    avgbs_21736    avgbs_15237    avgbs_44312    avgbs_65703    avgbs_65704    avgbs_73792    avgbs_97176    avgbs_97177    avgbs_98273    avgbs_98274    avgbs_106256    avgbs_6K_109589    avgbs_229206    avgbs_223777    avgbs_200453    avgbs_224738    avgbs_200686    avgbs_231926    avgbs_50384    avgbs_50385    avgbs_50386    avgbs_79877    avgbs_79878    avgbs_122166    avgbs_122167    avgbs_123833    avgbs_9209    avgbs_27496    avgbs_107049    avgbs_202497    avgbs_202775    avgbs_206482    avgbs_244349    avgbs_10565    avgbs_45809    avgbs_52804   
   3C 59 
  GMI_ES15_c7272_387     GMI_ES01_c14397_365    
 
  avgbs_2290    avgbs_24258    avgbs_34412    avgbs_68227    avgbs_83226    avgbs_216310    avgbs_2124    avgbs_7508    avgbs_11042    avgbs_14202    avgbs_14203    avgbs_14204    avgbs_27601    avgbs_27603    avgbs_28193    avgbs_31239    avgbs_32564    avgbs_32856    avgbs_45808    avgbs_56222    avgbs_87748    avgbs_87765    avgbs_99782    avgbs_99783    avgbs_110539    avgbs_112632    avgbs_112869    avgbs_119765    avgbs_119766    avgbs_201062    avgbs_201811    avgbs_213703    avgbs_217928    avgbs_218853    avgbs_219174    avgbs_220419    avgbs_221007    avgbs_200674    avgbs_221597    avgbs_206981    avgbs_215840    avgbs_24253    avgbs_40687    avgbs_40688    avgbs_40689    avgbs_62632    avgbs_70768    avgbs_71104    avgbs_71872    avgbs_108654    avgbs_108655    avgbs_108656    avgbs_209436    avgbs_200414    avgbs_33300    avgbs_67810    avgbs_80596    avgbs_86573    avgbs_114691    avgbs_118331    avgbs_218040    avgbs_3603    avgbs_80595    avgbs_87967    avgbs_201107    avgbs_77455    avgbs_12808    avgbs_12809    avgbs_18872    avgbs_18873    avgbs_18874    avgbs_42396    avgbs_46047    avgbs_116520    avgbs_116521    avgbs_70766    avgbs_86885    avgbs_93995    avgbs_110904    avgbs_120667    avgbs_207832    avgbs_217459    avgbs_65340    avgbs_204167    avgbs_7845    avgbs_7846    avgbs_8301    avgbs_9992    avgbs_12137    avgbs_18661    avgbs_43990    avgbs_81244    avgbs_82816    avgbs_85335    avgbs_95348    avgbs_101130    avgbs_101131    avgbs_108388    avgbs_108389    avgbs_111217    avgbs_116303    avgbs_116304    avgbs_229072    avgbs_229153    avgbs_229299    avgbs_200289    avgbs_224374    avgbs_219691    avgbs_231023    avgbs_205065    avgbs_206523    avgbs_232572    avgbs_1100    avgbs_1462    avgbs_12404    avgbs_18830    avgbs_19452    avgbs_21835    avgbs_51370    avgbs_67258    avgbs_67259    avgbs_67260    avgbs_85336    avgbs_89470    avgbs_94979    avgbs_104726    avgbs_120660    avgbs_120926    avgbs_201013    avgbs_229082    avgbs_205192    avgbs_206256    avgbs_207829    avgbs_103705    avgbs_103706    avgbs_7422    avgbs_9831    avgbs_23071    avgbs_23793    avgbs_50951    avgbs_50952    avgbs_65339    avgbs_80342    avgbs_89807    avgbs_96170    avgbs_112483    avgbs_112484    avgbs_118657    avgbs_118658    avgbs_124493    avgbs_66978    avgbs_89202    avgbs_56805    avgbs_56806    avgbs_115773    avgbs_239109    avgbs_243952    avgbs_238404    avgbs_120703    avgbs_120705    avgbs_67957    avgbs_82868    avgbs_74272    avgbs_47774   
   3C 60 
  GMI_ES02_c17906_415    
 
  avgbs_4483    avgbs_18503    avgbs_26618    avgbs_46503    avgbs_49522    avgbs_79189    avgbs_208519    avgbs_202760    avgbs_205460    avgbs_1410    avgbs_11887    avgbs_29928    avgbs_14151    avgbs_34958    avgbs_46788    avgbs_109952    avgbs_93176    avgbs_114421    avgbs_114577    avgbs_243067    avgbs_124306   
   3C 61 
 
 
  avgbs_111736    avgbs_244473    avgbs_99154    avgbs_108935    avgbs_18660    avgbs_18662    avgbs_66493    avgbs_119023    avgbs_119025    avgbs_62861    avgbs_38228    avgbs_119764   
   3C 62 
 
 
  avgbs_109601    avgbs_109628    avgbs_66213    avgbs_66214    avgbs_235683    avgbs_29000    avgbs_123105    avgbs_236142    avgbs_3605    avgbs_7157    avgbs_10904    avgbs_23278    avgbs_23279    avgbs_23280    avgbs_38730    avgbs_53820    avgbs_88481    avgbs_104725    avgbs_113717    avgbs_116456    avgbs_233912    avgbs_234198    avgbs_234362    avgbs_236431    avgbs_239001    avgbs_212116    avgbs_241727    avgbs_215867    avgbs_243933    avgbs_18828    avgbs_26364    avgbs_78035    avgbs_81642    avgbs_106608    avgbs_110557    avgbs_235569    avgbs_235656    avgbs_102040    avgbs_235551    avgbs_244068    avgbs_28996    avgbs_85988    avgbs_85989    avgbs_237411    avgbs_240493    avgbs_235852   
   3C 63 
 
 
  avgbs_6850    avgbs_29854    avgbs_115786    avgbs_61834    avgbs_99677    avgbs_104546    avgbs_119351    avgbs_120481    avgbs_123104    avgbs_237935    avgbs_244258    avgbs_18811    avgbs_215606    avgbs_233594    avgbs_239609   
   3C 64 
 
 
  avgbs_13232   
   3C 65 
  GMI_ES01_c13233_204    
 
  avgbs_3585    avgbs_3587    avgbs_61715    avgbs_91927    avgbs_91928    avgbs_91929    avgbs_94203    avgbs_118161    avgbs_125679    avgbs_200019    avgbs_215967    avgbs_17277    avgbs_200112    avgbs_4671    avgbs_4672    avgbs_22359    avgbs_22360    avgbs_22361    avgbs_22363    avgbs_25108    avgbs_46055    avgbs_60209    avgbs_82975    avgbs_87484    avgbs_101815    avgbs_107012    avgbs_109085    avgbs_109456    avgbs_116164    avgbs_216457    avgbs_213208    avgbs_201699    avgbs_213571    avgbs_218079    avgbs_218627    avgbs_218854    avgbs_219402    avgbs_211310    avgbs_215179    avgbs_215570    avgbs_200781    avgbs_126052    avgbs_56276    avgbs_72557    avgbs_2143    avgbs_106040    avgbs_200406    avgbs_7278    avgbs_124813    avgbs_124814    avgbs_61556    avgbs_26956    avgbs_232021    avgbs_90026    avgbs_212325    avgbs_24747    avgbs_43094    avgbs_43095    avgbs_43096    avgbs_94713    avgbs_209456    avgbs_65368    avgbs_241906    avgbs_101610    avgbs_101611    avgbs_244493    avgbs_11142    avgbs_11143    avgbs_111743    avgbs_122805    avgbs_122806    avgbs_122807    avgbs_201452    avgbs_4277    avgbs_4278    avgbs_9685    avgbs_9688    avgbs_17597    avgbs_20930    avgbs_20931    avgbs_68852    avgbs_100943    avgbs_100944    avgbs_100945    avgbs_125396    avgbs_234771    avgbs_203252    avgbs_244574    avgbs_241617    avgbs_20618    avgbs_65367    avgbs_106301    avgbs_75898    avgbs_15810    avgbs_17064    avgbs_118359    avgbs_74660    avgbs_56072   
   3C 66 
  GMI_ES_CC7714_103    
 
  avgbs_79112    avgbs_220520    avgbs_11705    avgbs_25720    avgbs_27468    avgbs_31168    avgbs_65638    avgbs_65639    avgbs_88764    avgbs_106084    avgbs_213772    avgbs_221071    avgbs_222944    avgbs_5332    avgbs_51494    avgbs_106039    avgbs_108069    avgbs_31984    avgbs_74201    avgbs_42555    avgbs_30930    avgbs_118078    avgbs_8597    avgbs_65369    avgbs_240234    avgbs_17017    avgbs_92841    avgbs_240515    avgbs_21130    avgbs_25077    avgbs_56429    avgbs_64935    avgbs_237572    avgbs_99712    avgbs_126051    avgbs_202725    avgbs_214038    avgbs_242890    avgbs_120529    avgbs_64514    avgbs_3611   
   3C 67 
  GMI_ES17_c13962_600    
 
  avgbs_982    avgbs_38914    avgbs_44080    avgbs_62735    avgbs_76021    avgbs_76022    avgbs_81630    avgbs_81631    avgbs_82032    avgbs_87553    avgbs_98384    avgbs_100044    avgbs_101583    avgbs_103420    avgbs_202617    avgbs_203379    avgbs_200348    avgbs_214821    avgbs_215183    avgbs_4408    avgbs_4656    avgbs_7068    avgbs_7592    avgbs_44723    avgbs_73975    avgbs_86073    avgbs_97085    avgbs_116044    avgbs_117093    avgbs_120807    avgbs_213201    avgbs_205269    avgbs_211472    avgbs_26617    avgbs_97084    avgbs_106192    avgbs_115302    avgbs_212607    avgbs_77317    avgbs_10616    avgbs_48183    avgbs_49956    avgbs_119168    avgbs_121494    avgbs_208894    avgbs_210189    avgbs_19701    avgbs_19702    avgbs_13091    avgbs_19230    avgbs_209774    avgbs_210631    avgbs_77955    avgbs_42808    avgbs_42809    avgbs_42810    avgbs_94714    avgbs_94715    avgbs_29663    avgbs_29664    avgbs_29665    avgbs_13036    avgbs_11916    avgbs_21501    avgbs_22877    avgbs_44724    avgbs_44725    avgbs_44726    avgbs_65845    avgbs_65846    avgbs_99020    avgbs_99023    avgbs_100761    avgbs_109439    avgbs_117478    avgbs_201755    avgbs_204930    avgbs_232526    avgbs_24239    avgbs_24240    avgbs_26032    avgbs_84207    avgbs_101523    avgbs_101524    avgbs_104580    avgbs_107821    avgbs_45634    avgbs_63161    avgbs_77956    avgbs_78758    avgbs_116719    avgbs_231468    avgbs_86633    avgbs_239989    avgbs_41046    avgbs_4633    avgbs_80617    avgbs_80618    avgbs_123821    avgbs_123822    avgbs_207518    avgbs_243452    avgbs_68732    avgbs_12098    avgbs_15799    avgbs_20634    avgbs_20635    avgbs_25717    avgbs_25718    avgbs_34853    avgbs_75510    avgbs_78572    avgbs_80378    avgbs_89649    avgbs_89650    avgbs_89651    avgbs_91843    avgbs_100001    avgbs_100002    avgbs_104436    avgbs_104437    avgbs_104438    avgbs_105391    avgbs_113330    avgbs_113331    avgbs_117348    avgbs_118574    avgbs_118575    avgbs_121493    avgbs_125148    avgbs_125656    avgbs_233742    avgbs_240219    avgbs_240405    avgbs_241786    avgbs_244019    avgbs_244562    avgbs_208385    avgbs_52813    avgbs_237332    avgbs_92141    avgbs_238742    avgbs_25934    avgbs_25935    avgbs_40924    avgbs_237020    avgbs_118353    avgbs_240469    avgbs_26809    avgbs_78255    avgbs_22817    avgbs_114205    avgbs_47886    avgbs_47887    avgbs_85232    avgbs_122059    avgbs_6628    avgbs_6629    avgbs_37289    avgbs_37290    avgbs_234372    avgbs_35606    avgbs_61698    avgbs_238803   
   3C 69 
  GMI_ES01_c17319_464     GMI_ES02_c12942_675    
 
  avgbs_3327    avgbs_7803    avgbs_7804    avgbs_82954    avgbs_86124    avgbs_107700    avgbs_112519    avgbs_112520    avgbs_222151    avgbs_222740    avgbs_12082    avgbs_35040    avgbs_42092    avgbs_70717    avgbs_76086    avgbs_76087    avgbs_86595    avgbs_113232    avgbs_113233    avgbs_113234    avgbs_118008    avgbs_124201    avgbs_200078    avgbs_218129    avgbs_214017    avgbs_205070    avgbs_205324    avgbs_12692    avgbs_88182    avgbs_123815    avgbs_201977    avgbs_35017    avgbs_63312    avgbs_92588    avgbs_124567    avgbs_105294    avgbs_85874    avgbs_82441    avgbs_90192    avgbs_116895    avgbs_240608    avgbs_98046    avgbs_98047    avgbs_63870    avgbs_107909    avgbs_124749    avgbs_33877    avgbs_33878    avgbs_78785    avgbs_78787    avgbs_108283    avgbs_109403    avgbs_237184    avgbs_238775    avgbs_242350    avgbs_70177    avgbs_87843    avgbs_101243    avgbs_107828    avgbs_107830    avgbs_62085    avgbs_6K_105061    avgbs_7309    avgbs_10751    avgbs_18071    avgbs_30565    avgbs_6K_68515    avgbs_75408    avgbs_75410    avgbs_78608    avgbs_104722    avgbs_104723    avgbs_124536    avgbs_202175    avgbs_239748    avgbs_242275    avgbs_1222    avgbs_53298    avgbs_64131    avgbs_68530    avgbs_75511    avgbs_83812    avgbs_94298    avgbs_119994    avgbs_225205    avgbs_6739    avgbs_6904    avgbs_6905    avgbs_12373    avgbs_17451    avgbs_19410    avgbs_27017    avgbs_35016    avgbs_44997    avgbs_45987    avgbs_45988    avgbs_51180    avgbs_56524    avgbs_56686    avgbs_76605    avgbs_76992    avgbs_80267    avgbs_80268    avgbs_81695    avgbs_88869    avgbs_89594    avgbs_92633    avgbs_98638    avgbs_98639    avgbs_100043    avgbs_100181    avgbs_106082    avgbs_108074    avgbs_116195    avgbs_121983    avgbs_122478    avgbs_122479    avgbs_124203    avgbs_126334    avgbs_203674    avgbs_240222    avgbs_241307    avgbs_206497    avgbs_242331    avgbs_212873    avgbs_15904    avgbs_15905    avgbs_15906    avgbs_64980    avgbs_88909    avgbs_109188    avgbs_205317    avgbs_699    avgbs_1077    avgbs_7340    avgbs_9438    avgbs_9439    avgbs_9440    avgbs_10437    avgbs_12164    avgbs_15180    avgbs_15768    avgbs_17703    avgbs_20643    avgbs_24417    avgbs_33324    avgbs_34187    avgbs_36347    avgbs_36348    avgbs_41050    avgbs_53150    avgbs_62305    avgbs_62306    avgbs_62670    avgbs_72988    avgbs_79010    avgbs_80567    avgbs_80568    avgbs_90012    avgbs_93167    avgbs_93919    avgbs_96408    avgbs_108985    avgbs_108993    avgbs_110601    avgbs_115848    avgbs_116060    avgbs_116513    avgbs_118559    avgbs_118560    avgbs_124373    avgbs_124566    avgbs_125964    avgbs_232896    avgbs_233702    avgbs_234631    avgbs_235464    avgbs_235469    avgbs_235630    avgbs_236030    avgbs_236421    avgbs_236500    avgbs_214954    avgbs_200586    avgbs_240559    avgbs_240887    avgbs_241873    avgbs_242037    avgbs_215807    avgbs_243368    avgbs_244673    avgbs_30521    avgbs_38179    avgbs_40652    avgbs_40653    avgbs_50427    avgbs_76757    avgbs_76758    avgbs_237144    avgbs_242698    avgbs_17808    avgbs_32014    avgbs_78561    avgbs_12008    avgbs_27319    avgbs_94504    avgbs_235703    avgbs_10920    avgbs_45844    avgbs_98425    avgbs_45577    avgbs_47770    avgbs_233347    avgbs_236748    avgbs_237385    avgbs_208400    avgbs_241697    avgbs_15914    avgbs_233593    avgbs_234593    avgbs_1635    avgbs_60456    avgbs_67300    avgbs_67301    avgbs_3387    avgbs_4937    avgbs_40701    avgbs_100132    avgbs_100133    avgbs_100134    avgbs_122281    avgbs_240083   
   3C 70 
 
 
  avgbs_4164    avgbs_221533    avgbs_2882    avgbs_2883    avgbs_91229    avgbs_225136   
   3C 71 
  GMI_ES_CC11028_196    
 
  avgbs_4961    avgbs_6700    avgbs_35457    avgbs_99128    avgbs_99129    avgbs_118202    avgbs_216478    avgbs_204334    avgbs_68320    avgbs_112778    avgbs_200467    avgbs_220161    avgbs_215837    avgbs_68546    avgbs_99767    avgbs_105102    avgbs_59119    avgbs_4740    avgbs_22878    avgbs_29775    avgbs_61409    avgbs_66956    avgbs_71236    avgbs_77826    avgbs_90214    avgbs_204232    avgbs_23464    avgbs_23465    avgbs_1664    avgbs_93181    avgbs_93182    avgbs_28682    avgbs_33038    avgbs_54186    avgbs_87946    avgbs_87947    avgbs_56310    avgbs_56311    avgbs_56312    avgbs_61546    avgbs_93940    avgbs_93941    avgbs_47177    avgbs_236947    avgbs_505    avgbs_2884    avgbs_7252    avgbs_8806    avgbs_8807    avgbs_10617    avgbs_10969    avgbs_12841    avgbs_14879    avgbs_20133    avgbs_20352    avgbs_20353    avgbs_22239    avgbs_32317    avgbs_62202    avgbs_62203    avgbs_62204    avgbs_62268    avgbs_68689    avgbs_68690    avgbs_75357    avgbs_77963    avgbs_97372    avgbs_101069    avgbs_105535    avgbs_105536    avgbs_123328    avgbs_123469    avgbs_123745    avgbs_124607    avgbs_126218    avgbs_202407    avgbs_235923    avgbs_240939    avgbs_244284    avgbs_19427    avgbs_29797    avgbs_76497    avgbs_78948    avgbs_201932    avgbs_237717    avgbs_12887    avgbs_13308    avgbs_14235    avgbs_14236    avgbs_14237    avgbs_21744    avgbs_24241    avgbs_50949    avgbs_87100    avgbs_107327    avgbs_108208    avgbs_116720    avgbs_125460    avgbs_201176    avgbs_208994    avgbs_235515    avgbs_239404    avgbs_240807    avgbs_241227    avgbs_243387    avgbs_244094    avgbs_168    avgbs_50471    avgbs_50472    avgbs_105860    avgbs_105861    avgbs_111190    avgbs_111191    avgbs_161    avgbs_11710    avgbs_72118    avgbs_87088    avgbs_240912    avgbs_244117    avgbs_239329    avgbs_64610    avgbs_208248    avgbs_94667    avgbs_95262    avgbs_234447    avgbs_237971    avgbs_14155    avgbs_14420    avgbs_35159    avgbs_38995    avgbs_52469    avgbs_58872    avgbs_58873    avgbs_59814    avgbs_63785    avgbs_83341    avgbs_1438    avgbs_69139    avgbs_217166    avgbs_7395    avgbs_4064    avgbs_104399    avgbs_91437    avgbs_91438    avgbs_30958    avgbs_16849   
   3C 72 
 
 
  avgbs_87272    avgbs_33266    avgbs_36374    avgbs_236284   
   3C 73 
 
 
  avgbs_102602    avgbs_110712    avgbs_56    avgbs_57    avgbs_58    avgbs_27466    avgbs_27467    avgbs_71348    avgbs_71349    avgbs_111907    avgbs_36794    avgbs_50256    avgbs_50257    avgbs_50258    avgbs_83149    avgbs_239619    avgbs_240988    avgbs_11226    avgbs_121504    avgbs_234292    avgbs_216068    avgbs_239055    avgbs_26002    avgbs_26003    avgbs_81685    avgbs_73342    avgbs_238777    avgbs_241633   
   3C 74 
  GMI_ES02_c841_728    
 
  avgbs_110711    avgbs_6K_86839    avgbs_118912    avgbs_2396    avgbs_110841    avgbs_110842    avgbs_229159    avgbs_17631    avgbs_46761    avgbs_46762    avgbs_48465    avgbs_201208    avgbs_238645    avgbs_234686    avgbs_40594    avgbs_235131    avgbs_207356    avgbs_74244    avgbs_107844    avgbs_107801    avgbs_242445    avgbs_51524    avgbs_223675    avgbs_23122    avgbs_107713   
   3C 75 
  GMI_ES01_c16727_290    
 
  avgbs_23210    avgbs_32864    avgbs_42986    avgbs_42987    avgbs_42988    avgbs_68755    avgbs_77413    avgbs_80701    avgbs_87554    avgbs_96407    avgbs_201020    avgbs_217140    avgbs_204752    avgbs_220422    avgbs_220461    avgbs_221593    avgbs_222576    avgbs_11130    avgbs_11794    avgbs_19850    avgbs_23123    avgbs_34569    avgbs_34570    avgbs_34571    avgbs_63392    avgbs_68656    avgbs_68657    avgbs_90555    avgbs_92514    avgbs_96678    avgbs_96679    avgbs_220418    avgbs_220733    avgbs_94808    avgbs_18069    avgbs_18070    avgbs_106314    avgbs_113611    avgbs_217271    avgbs_206380    avgbs_222523    avgbs_5222    avgbs_12009    avgbs_14026    avgbs_41262    avgbs_41263    avgbs_50944    avgbs_61692    avgbs_2425    avgbs_100504    avgbs_315    avgbs_79454    avgbs_85873    avgbs_15179    avgbs_43717    avgbs_125401    avgbs_72553    avgbs_203561    avgbs_107845    avgbs_111460    avgbs_111465    avgbs_237202    avgbs_63003    avgbs_43510    avgbs_75983   
   3C 76 
  GMI_ES15_c14533_341    
 
  avgbs_5157    avgbs_5158    avgbs_79936    avgbs_218792    avgbs_14050    avgbs_14051    avgbs_20852    avgbs_49053    avgbs_34706    avgbs_96826    avgbs_96827    avgbs_21050    avgbs_72758    avgbs_122038    avgbs_224007   
   3C 77 
 
 
  avgbs_207732   
   3C 78 
 
 
  avgbs_11512    avgbs_11513    avgbs_116898    avgbs_94311    avgbs_240758    avgbs_235832   
   3C 79 
 
 
  avgbs_221979    avgbs_47522    avgbs_47523    avgbs_15332    avgbs_116691    avgbs_37899   
   3C 80 
 
 
  avgbs_63497    avgbs_63498    avgbs_63499   
   3C 81 
 
 
  avgbs_18626    avgbs_11219    avgbs_234155    avgbs_224006   
   3C 82 
 
 
  avgbs_3003    avgbs_11843    avgbs_20525    avgbs_46596   
   3C 83 
 
 
  avgbs_122855    avgbs_223001    avgbs_233855   
   3C 84 
 
 
  avgbs_10137    avgbs_213270    avgbs_10194    avgbs_17360    avgbs_78065   
   3C 85 
 
 
  avgbs_87637   
   3C 86 
 
 
  avgbs_19352    avgbs_63871    avgbs_115129    avgbs_2114    avgbs_243327    avgbs_70915   
   3C 87 
  BA_grs_c10318_236    
 
  avgbs_101014    avgbs_574    avgbs_711    avgbs_1474    avgbs_1475    avgbs_6482    avgbs_8097    avgbs_9151    avgbs_10331    avgbs_17126    avgbs_18598    avgbs_21137    avgbs_21138    avgbs_21139    avgbs_27016    avgbs_29197    avgbs_32338    avgbs_40844    avgbs_41948    avgbs_42965    avgbs_6K_40569    avgbs_46454    avgbs_48596    avgbs_51649    avgbs_51650    avgbs_52815    avgbs_57223    avgbs_57225    avgbs_59189    avgbs_59454    avgbs_67564    avgbs_67565    avgbs_69782    avgbs_71157    avgbs_71158    avgbs_72533    avgbs_75982    avgbs_82258    avgbs_84761    avgbs_86296    avgbs_91831    avgbs_95311    avgbs_96834    avgbs_97566    avgbs_101127    avgbs_101128    avgbs_104156    avgbs_105811    avgbs_107668    avgbs_109852    avgbs_119891    avgbs_121271    avgbs_122986    avgbs_122987    avgbs_216221    avgbs_216834    avgbs_216884    avgbs_216914    avgbs_213353    avgbs_218463    avgbs_218733    avgbs_214419    avgbs_219360    avgbs_219957    avgbs_220069    avgbs_204531    avgbs_204594    avgbs_220604    avgbs_205130    avgbs_220925    avgbs_221510    avgbs_221641    avgbs_206340    avgbs_13114    avgbs_13115    avgbs_33325    avgbs_40702    avgbs_1038    avgbs_4737    avgbs_4738    avgbs_11842    avgbs_21318    avgbs_21319    avgbs_21712    avgbs_22112    avgbs_25838    avgbs_27093    avgbs_35484    avgbs_44854    avgbs_50272    avgbs_50350    avgbs_54354    avgbs_57400    avgbs_57401    avgbs_57402    avgbs_74230    avgbs_79695    avgbs_86823    avgbs_88870    avgbs_88871    avgbs_98713    avgbs_105565    avgbs_105808    avgbs_201012    avgbs_216899    avgbs_217657    avgbs_217702    avgbs_219258    avgbs_220218    avgbs_205351    avgbs_29251    avgbs_29252    avgbs_29253    avgbs_40843    avgbs_5473    avgbs_57404    avgbs_62669    avgbs_76830    avgbs_76831    avgbs_104652    avgbs_104695    avgbs_115566    avgbs_115597    avgbs_115598    avgbs_124535    avgbs_124537    avgbs_214548    avgbs_220983    avgbs_215635    avgbs_7208    avgbs_21689    avgbs_14523    avgbs_63084    avgbs_63085    avgbs_124484    avgbs_223093    avgbs_6115    avgbs_9884    avgbs_223314    avgbs_223723    avgbs_91121    avgbs_91122    avgbs_23595    avgbs_23596    avgbs_34320    avgbs_34319    avgbs_200902    avgbs_86031    avgbs_200331    avgbs_224458    avgbs_14982    avgbs_79620    avgbs_79621    avgbs_79622    avgbs_113778    avgbs_7022    avgbs_7023   
   3C 88 
  GMI_ES17_c8729_764    
 
  avgbs_108155    avgbs_17043    avgbs_17044    avgbs_41086    avgbs_45884    avgbs_48512    avgbs_50977    avgbs_73079    avgbs_113906    avgbs_218773    avgbs_220163    avgbs_221890    avgbs_222188    avgbs_222407    avgbs_222716    avgbs_102359    avgbs_102360   
   3C 89 
 
 
  avgbs_21376   
   3C 90 
 
 
  avgbs_32119    avgbs_32120    avgbs_32121    avgbs_229969    avgbs_94101   
   3C 91 
 
 
  avgbs_104794    avgbs_225423    avgbs_7437   
   3C 92 
 
 
  avgbs_200407   
   3C 93 
 
 
  avgbs_202109   
   3C 94 
  GMI_ES_CC16445_119    
 
  avgbs_93704    avgbs_222125    avgbs_14083    avgbs_17163    avgbs_17164    avgbs_31985    avgbs_67302    avgbs_102384    avgbs_102385    avgbs_105463    avgbs_110433    avgbs_110434    avgbs_35300    avgbs_9466    avgbs_22003    avgbs_25311    avgbs_109922    avgbs_118476    avgbs_11202    avgbs_32908    avgbs_56178    avgbs_56179    avgbs_56180    avgbs_58623    avgbs_119460    avgbs_119461    avgbs_119462    avgbs_230528    avgbs_35238    avgbs_35239    avgbs_113311   
   3C 95 
 
 
  avgbs_100882    avgbs_101105   
   3C 96 
 
 
  avgbs_42915    avgbs_49714    avgbs_49715    avgbs_32319   
   3C 97 
 
 
  avgbs_200843   
   3C 99 
 
 
  avgbs_1669    avgbs_26441    avgbs_26442    avgbs_217682    avgbs_117977    avgbs_117978    avgbs_118671    avgbs_216428    avgbs_53902   
   3C 100 
 
 
  avgbs_121778    avgbs_3226    avgbs_3227   
   3C 101 
 
 
  avgbs_217401    avgbs_91846    avgbs_109393    avgbs_109394    avgbs_213814   
   3C 102 
 
 
  avgbs_91847    avgbs_93560    avgbs_109812    avgbs_109820    avgbs_23509    avgbs_23510    avgbs_5980    avgbs_50011   
   3C 103 
 
 
  avgbs_91403    avgbs_115433    avgbs_218166    avgbs_221897    avgbs_104444    avgbs_104445    avgbs_68020   
   3C 104 
 
 
  avgbs_93849    avgbs_94464    avgbs_124720    avgbs_221360    avgbs_77009   
   3C 105 
 
 
  avgbs_218013    avgbs_218273    avgbs_38286    avgbs_50855    avgbs_41342   
   3C 106 
 
 
  avgbs_6K_104364    avgbs_71404    avgbs_71405    avgbs_71406    avgbs_216887    avgbs_223000    avgbs_51432   
 
 
   Chromosome 4C    Chr   Pos   Framework   Placed SNP  Placed GBS  
   4C 0 
  GMI_ES15_c12434_433    
 
  avgbs_230704    avgbs_10459    avgbs_63267    avgbs_72464    avgbs_85915    avgbs_113378    avgbs_113379    avgbs_231397    avgbs_231452    avgbs_41332    avgbs_54293   
   4C 7 
 
 
  avgbs_19313    avgbs_68557    avgbs_37263    avgbs_37264    avgbs_49217    avgbs_47690    avgbs_84051   
   4C 8 
 
 
  avgbs_36456    avgbs_75557    avgbs_222322    avgbs_15869   
   4C 9 
 
 
  avgbs_107945    avgbs_222262    avgbs_113933    avgbs_222539    avgbs_218196    avgbs_117380   
   4C 10 
 
 
  avgbs_115387    avgbs_5586    avgbs_40402    avgbs_35313   
   4C 11 
 
 
  avgbs_12190    avgbs_74478    avgbs_49730    avgbs_63439    avgbs_214107    avgbs_234689   
   4C 12 
 
 
  avgbs_41659    avgbs_45626    avgbs_87609    avgbs_91855    avgbs_91856    avgbs_124206    avgbs_52685    avgbs_99608    avgbs_9601    avgbs_109427    avgbs_109428    avgbs_123123   
   4C 13 
 
 
  avgbs_107847    avgbs_217585    avgbs_77282    avgbs_220405    avgbs_74591    avgbs_53912   
   4C 14 
 
 
  avgbs_202409    avgbs_66031   
   4C 15 
 
 
  avgbs_118143    avgbs_222513    avgbs_207585   
   4C 16 
  GMI_ES15_c337_508    
 
  avgbs_114542    avgbs_114543    avgbs_218798    avgbs_18561    avgbs_18578    avgbs_78042    avgbs_217742    avgbs_12154    avgbs_12155    avgbs_30091    avgbs_9725    avgbs_213261    avgbs_231497    avgbs_2197    avgbs_7905    avgbs_13799    avgbs_13800    avgbs_36080    avgbs_36081    avgbs_37253    avgbs_40499    avgbs_44620    avgbs_61889    avgbs_77853    avgbs_91775    avgbs_91776    avgbs_113023    avgbs_230066    avgbs_230790    avgbs_225663    avgbs_230884    avgbs_231097    avgbs_224020    avgbs_47205    avgbs_66916    avgbs_86375    avgbs_86376    avgbs_100146    avgbs_110277    avgbs_22262    avgbs_225481    avgbs_78012    avgbs_224841    avgbs_225071    avgbs_224863    avgbs_67364    avgbs_78294   
   4C 17 
 
 
  avgbs_48656    avgbs_67442    avgbs_67443    avgbs_233225    avgbs_242988   
   4C 19 
 
 
  avgbs_29335    avgbs_53763    avgbs_217839    avgbs_217214    avgbs_6K_2822   
   4C 20 
 
 
  avgbs_33383    avgbs_107944    avgbs_113931    avgbs_113932   
   4C 21 
 
 
  avgbs_115789    avgbs_233779   
   4C 24 
  GMI_ES17_c7355_434    
 
  avgbs_236656    avgbs_15392    avgbs_19491    avgbs_35183    avgbs_35184    avgbs_35185    avgbs_205287    avgbs_50861    avgbs_11943    avgbs_206550    avgbs_49819    avgbs_82500    avgbs_82502   
   4C 25 
 
  GMI_ES01_c11917_356   
  avgbs_111522    avgbs_64703    avgbs_219680    avgbs_9971    avgbs_83421    avgbs_216791    avgbs_7054    avgbs_7055    avgbs_55596    avgbs_59071    avgbs_203074    avgbs_122223    avgbs_124003    avgbs_66579    avgbs_6K_26418    avgbs_2264    avgbs_201119   
   4C 26 
 
 
  avgbs_111770    avgbs_93856    avgbs_46956    avgbs_50862    avgbs_50863    avgbs_50864    avgbs_50865    avgbs_33382    avgbs_36497    avgbs_36498    avgbs_98908    avgbs_93710    avgbs_17177    avgbs_17178    avgbs_33384    avgbs_33385    avgbs_73595    avgbs_116899    avgbs_224380    avgbs_204039    avgbs_59301    avgbs_54877    avgbs_54878    avgbs_99931    avgbs_106555    avgbs_236291    avgbs_119709    avgbs_6K_27541    avgbs_107282    avgbs_107283    avgbs_107284    avgbs_41157    avgbs_235261    avgbs_60136    avgbs_60137    avgbs_67501    avgbs_238612    avgbs_109626    avgbs_108765   
   4C 27 
 
 
  avgbs_25369    avgbs_25370    avgbs_46301    avgbs_15263    avgbs_73557   
   4C 28 
 
 
  avgbs_36945    avgbs_36946    avgbs_72837    avgbs_220154    avgbs_222690    avgbs_5721    avgbs_21477    avgbs_216529    avgbs_202577    avgbs_17897    avgbs_95655    avgbs_78868    avgbs_68339    avgbs_43712    avgbs_70984    avgbs_70985    avgbs_81063   
   4C 29 
  GMI_ES02_c933_584    
 
  avgbs_123384    avgbs_123385    avgbs_29721    avgbs_54015    avgbs_61526    avgbs_202813    avgbs_47471    avgbs_47472    avgbs_115111    avgbs_17975    avgbs_17978    avgbs_19608    avgbs_19609    avgbs_6K_21554    avgbs_36888    avgbs_36889    avgbs_52269    avgbs_62028    avgbs_62987    avgbs_76918    avgbs_104945    avgbs_110548    avgbs_202648    avgbs_236702    avgbs_203671    avgbs_224512    avgbs_242681    avgbs_65426    avgbs_120451    avgbs_67471    avgbs_73642    avgbs_120869    avgbs_236620    avgbs_32377    avgbs_81064    avgbs_204982    avgbs_115583    avgbs_115584    avgbs_115585    avgbs_124082    avgbs_124083    avgbs_126231    avgbs_201892    avgbs_102441    avgbs_39663    avgbs_490    avgbs_240599    avgbs_125985    avgbs_201877    avgbs_59920    avgbs_45985    avgbs_27270   
   4C 31 
 
 
  avgbs_122429    avgbs_81203   
   4C 32 
 
 
  avgbs_58681   
   4C 33 
 
 
  avgbs_219455    avgbs_69894    avgbs_48302    avgbs_101943    avgbs_3985    avgbs_3986    avgbs_88560    avgbs_7008    avgbs_7009    avgbs_100217    avgbs_125984    avgbs_20914    avgbs_64917    avgbs_69711    avgbs_85186    avgbs_85187    avgbs_90740    avgbs_102157    avgbs_114495    avgbs_114496    avgbs_201260    avgbs_204448    avgbs_86417    avgbs_125657    avgbs_57217    avgbs_221031    avgbs_71116    avgbs_109998    avgbs_109999    avgbs_15184    avgbs_18587   
   4C 34 
 
 
  avgbs_26828    avgbs_26829    avgbs_20754    avgbs_35458    avgbs_111162    avgbs_59970    avgbs_203954    avgbs_43474    avgbs_201426   
   4C 35 
 
 
  avgbs_2916    avgbs_2917    avgbs_2918    avgbs_88603    avgbs_24777    avgbs_46025    avgbs_36229    avgbs_36230    avgbs_50441    avgbs_75216    avgbs_75221    avgbs_115438    avgbs_115439    avgbs_2810    avgbs_2811   
   4C 36 
 
  GMI_DS_oPt-15903_270   
  avgbs_11991    avgbs_17996    avgbs_120757    avgbs_87664    avgbs_87665    avgbs_7655    avgbs_85023    avgbs_216939    avgbs_217873    avgbs_86418    avgbs_35128    avgbs_35129    avgbs_80902    avgbs_218122    avgbs_27309    avgbs_32903    avgbs_217717    avgbs_94336    avgbs_236859    avgbs_28440    avgbs_8981    avgbs_11680    avgbs_11681    avgbs_11682    avgbs_15515    avgbs_18268    avgbs_35706    avgbs_102511    avgbs_120983    avgbs_122237    avgbs_55538    avgbs_237521    avgbs_16    avgbs_24    avgbs_2400    avgbs_3954    avgbs_4677    avgbs_4730    avgbs_6069    avgbs_8530    avgbs_10752    avgbs_11818    avgbs_11819    avgbs_13383    avgbs_15035    avgbs_15336    avgbs_16808    avgbs_19217    avgbs_20129    avgbs_20209    avgbs_20759    avgbs_20808    avgbs_21526    avgbs_21564    avgbs_21565    avgbs_21599    avgbs_25043    avgbs_25426    avgbs_25739    avgbs_28306    avgbs_30226    avgbs_30227    avgbs_30366    avgbs_37423    avgbs_37614    avgbs_37615    avgbs_39660    avgbs_40214    avgbs_40629    avgbs_42586    avgbs_42748    avgbs_43203    avgbs_47851    avgbs_49671    avgbs_49962    avgbs_49963    avgbs_50593    avgbs_51309    avgbs_51800    avgbs_52098    avgbs_53208    avgbs_58937    avgbs_60404    avgbs_60553    avgbs_60554    avgbs_62110    avgbs_62132    avgbs_62133    avgbs_63453    avgbs_65676    avgbs_65695    avgbs_65735    avgbs_66840    avgbs_66841    avgbs_67406    avgbs_68113    avgbs_68451    avgbs_68452    avgbs_68935    avgbs_69509    avgbs_70761    avgbs_70763    avgbs_76534    avgbs_77157    avgbs_77539    avgbs_77867    avgbs_79709    avgbs_80474    avgbs_80645    avgbs_81073    avgbs_81144    avgbs_81338    avgbs_82454    avgbs_82539    avgbs_82908    avgbs_82909    avgbs_84045    avgbs_84519    avgbs_85833    avgbs_86358    avgbs_88442    avgbs_89995    avgbs_90344    avgbs_90345    avgbs_91109    avgbs_92371    avgbs_92372    avgbs_92390    avgbs_94241    avgbs_94706    avgbs_100691    avgbs_102323    avgbs_105073    avgbs_105368    avgbs_105374    avgbs_105495    avgbs_108249    avgbs_108251    avgbs_109237    avgbs_109498    avgbs_109545    avgbs_110006    avgbs_110007    avgbs_110666    avgbs_111333    avgbs_111845    avgbs_112136    avgbs_112194    avgbs_112195    avgbs_113349    avgbs_113350    avgbs_113360    avgbs_113733    avgbs_113734    avgbs_113805    avgbs_115613    avgbs_116013    avgbs_118522    avgbs_119805    avgbs_121941    avgbs_124821    avgbs_124822    avgbs_125592    avgbs_126145    avgbs_201009    avgbs_233377    avgbs_201536    avgbs_201608    avgbs_234483    avgbs_202080    avgbs_236278    avgbs_203405    avgbs_219335    avgbs_204184    avgbs_204302    avgbs_238649    avgbs_204604    avgbs_239553    avgbs_205099    avgbs_240200    avgbs_205721    avgbs_205815    avgbs_205894    avgbs_241782    avgbs_206701    avgbs_206763    avgbs_242472    avgbs_207717    avgbs_243891    avgbs_6073    avgbs_6074    avgbs_15245    avgbs_15247    avgbs_25882    avgbs_25883    avgbs_39371    avgbs_43670    avgbs_50594    avgbs_51539    avgbs_60856    avgbs_63852    avgbs_64806    avgbs_107422    avgbs_108645    avgbs_118300    avgbs_120798    avgbs_124205    avgbs_204096    avgbs_1925    avgbs_1926    avgbs_4013    avgbs_16562    avgbs_21687    avgbs_21688    avgbs_27823    avgbs_28168    avgbs_28169    avgbs_30519    avgbs_34261    avgbs_34303    avgbs_34304    avgbs_39730    avgbs_42091    avgbs_50921    avgbs_50922    avgbs_54072    avgbs_59997    avgbs_61555    avgbs_62329    avgbs_65005    avgbs_66285    avgbs_66798    avgbs_67718    avgbs_67719    avgbs_69632    avgbs_71937    avgbs_72856    avgbs_73314    avgbs_77286    avgbs_79493    avgbs_80581    avgbs_80582    avgbs_81514    avgbs_82701    avgbs_87096    avgbs_99406    avgbs_103495    avgbs_103496    avgbs_105607    avgbs_106011    avgbs_106117    avgbs_108789    avgbs_109497    avgbs_110333    avgbs_6K_102362    avgbs_111453    avgbs_111454    avgbs_112034    avgbs_113486    avgbs_114044    avgbs_114877    avgbs_114878    avgbs_115320    avgbs_115580    avgbs_115581    avgbs_116869    avgbs_116870    avgbs_119069    avgbs_119804    avgbs_120472    avgbs_121708    avgbs_121709    avgbs_121851    avgbs_201593    avgbs_236292    avgbs_237958    avgbs_204226    avgbs_242861    avgbs_207797    avgbs_208059    avgbs_225814    avgbs_244681    avgbs_399    avgbs_14885    avgbs_23970    avgbs_23971    avgbs_28790    avgbs_28791    avgbs_38416    avgbs_41437    avgbs_41438    avgbs_49547    avgbs_78121    avgbs_93134    avgbs_203118    avgbs_244578    avgbs_10289    avgbs_14776    avgbs_14777    avgbs_26773    avgbs_45091    avgbs_45971    avgbs_55665    avgbs_55666    avgbs_74550    avgbs_96177    avgbs_103413    avgbs_106919    avgbs_20684    avgbs_203175    avgbs_239031    avgbs_376    avgbs_51006    avgbs_69810    avgbs_71668    avgbs_77387    avgbs_94588    avgbs_201677    avgbs_206967    avgbs_5589    avgbs_10726    avgbs_14783    avgbs_32614    avgbs_34147    avgbs_36524    avgbs_37282    avgbs_41473    avgbs_61554    avgbs_82069    avgbs_87094    avgbs_111487    avgbs_114286    avgbs_114287    avgbs_115406    avgbs_242149    avgbs_242663    avgbs_705    avgbs_706    avgbs_14614    avgbs_87804    avgbs_6K_105459    avgbs_234068    avgbs_236132    avgbs_242657    avgbs_22594    avgbs_47596    avgbs_47597    avgbs_62620    avgbs_62621    avgbs_63778    avgbs_64900    avgbs_66072    avgbs_73916    avgbs_79731    avgbs_79732    avgbs_86415    avgbs_92652    avgbs_94337    avgbs_108608    avgbs_123019    avgbs_206245    avgbs_244121    avgbs_31588    avgbs_32257    avgbs_69543    avgbs_80455    avgbs_102682    avgbs_229487    avgbs_217946    avgbs_236171    avgbs_239220    avgbs_26577    avgbs_31554    avgbs_42358    avgbs_42363    avgbs_55258    avgbs_55527    avgbs_63658    avgbs_74520    avgbs_85123    avgbs_96785    avgbs_121272    avgbs_121273    avgbs_121274    avgbs_124867    avgbs_235947    avgbs_219617    avgbs_25    avgbs_5559    avgbs_20053    avgbs_20054    avgbs_43531    avgbs_43676    avgbs_52022    avgbs_52023    avgbs_80740    avgbs_89797    avgbs_234334    avgbs_236478    avgbs_197    avgbs_198    avgbs_22369    avgbs_26070    avgbs_36031    avgbs_42976    avgbs_81177    avgbs_81178    avgbs_84387    avgbs_84388    avgbs_104130    avgbs_115223    avgbs_117676    avgbs_235401    avgbs_208204    avgbs_208445    avgbs_1188    avgbs_15863    avgbs_66071    avgbs_75507    avgbs_75508    avgbs_78194    avgbs_95206    avgbs_105123    avgbs_26620    avgbs_54037    avgbs_69624    avgbs_103540    avgbs_37107    avgbs_60049    avgbs_80225    avgbs_80226    avgbs_80227    avgbs_81698    avgbs_104646    avgbs_114475    avgbs_244376    avgbs_28505    avgbs_39558    avgbs_39559    avgbs_71487    avgbs_80142    avgbs_94592    avgbs_94593    avgbs_109244    avgbs_114892    avgbs_114893    avgbs_233682    avgbs_235772    avgbs_222308    avgbs_834    avgbs_29595    avgbs_49108    avgbs_103785    avgbs_2053    avgbs_78302    avgbs_78303    avgbs_78304    avgbs_95407    avgbs_220234    avgbs_241989    avgbs_40003    avgbs_43472    avgbs_233240    avgbs_236179    avgbs_206992    avgbs_8872    avgbs_29423    avgbs_51008    avgbs_75250    avgbs_236699    avgbs_3525    avgbs_35325    avgbs_35486    avgbs_81366    avgbs_106168    avgbs_118238    avgbs_118239    avgbs_2232    avgbs_26626    avgbs_35221    avgbs_43126    avgbs_54210    avgbs_91741    avgbs_103459    avgbs_201965    avgbs_238437    avgbs_242040    avgbs_98297    avgbs_1593    avgbs_32352    avgbs_124072    avgbs_229710    avgbs_8362    avgbs_47605    avgbs_235172    avgbs_241893    avgbs_54079    avgbs_78359    avgbs_88365    avgbs_22681    avgbs_22682    avgbs_32539    avgbs_242254    avgbs_52998    avgbs_52999    avgbs_10600    avgbs_10663    avgbs_82505    avgbs_63783    avgbs_97562   
   4C 37 
 
 
  avgbs_203007    avgbs_51694    avgbs_52876    avgbs_22853    avgbs_29905    avgbs_108056    avgbs_120802    avgbs_235752    avgbs_237376    avgbs_206581    avgbs_6555    avgbs_11998    avgbs_67219    avgbs_123773    avgbs_123775    avgbs_201348    avgbs_207601    avgbs_50031    avgbs_206745    avgbs_28002    avgbs_208141    avgbs_65325    avgbs_80010    avgbs_109940    avgbs_116761    avgbs_243391    avgbs_41075    avgbs_51973    avgbs_72433    avgbs_3897    avgbs_71249    avgbs_82259    avgbs_5872    avgbs_5873    avgbs_11767    avgbs_240233    avgbs_72434    avgbs_111170    avgbs_18027    avgbs_18028    avgbs_18029   
   4C 38 
 
 
  avgbs_21421    avgbs_74896    avgbs_74897    avgbs_219083    avgbs_220378    avgbs_65444    avgbs_88604    avgbs_88935    avgbs_88936    avgbs_15527    avgbs_34772    avgbs_43170    avgbs_53537    avgbs_232988    avgbs_51665    avgbs_50336    avgbs_80377    avgbs_80606    avgbs_100694    avgbs_112023    avgbs_202094    avgbs_237394    avgbs_238865    avgbs_244652    avgbs_238564    avgbs_23130    avgbs_52066    avgbs_52067    avgbs_24584    avgbs_65258    avgbs_28729    avgbs_28730    avgbs_36987    avgbs_95019    avgbs_95020    avgbs_29599   
   4C 39 
 
 
  avgbs_218907   
   4C 40 
 
 
  avgbs_38796   
   4C 42 
  GMI_ES01_c515_543    
 
  avgbs_68347    avgbs_8375    avgbs_53521    avgbs_201269    avgbs_219012    avgbs_219135    avgbs_65432    avgbs_6116    avgbs_212127    avgbs_81590    avgbs_1680    avgbs_69196    avgbs_80200    avgbs_80282    avgbs_96076    avgbs_96077    avgbs_204328    avgbs_4367    avgbs_32894    avgbs_36904    avgbs_46024    avgbs_61212    avgbs_79520    avgbs_200305    avgbs_230601    avgbs_200427    avgbs_231096    avgbs_224911    avgbs_66392    avgbs_82271    avgbs_97764    avgbs_65434    avgbs_36728    avgbs_105930    avgbs_14605    avgbs_14878    avgbs_39989    avgbs_51321    avgbs_54545    avgbs_62081    avgbs_62082    avgbs_110003    avgbs_217125    avgbs_32193    avgbs_26830    avgbs_55563    avgbs_98080    avgbs_98081    avgbs_86404    avgbs_202286    avgbs_205793    avgbs_39239    avgbs_65699    avgbs_206154    avgbs_204357    avgbs_25160    avgbs_25368    avgbs_34870    avgbs_34871    avgbs_45290    avgbs_45291    avgbs_47469    avgbs_47470    avgbs_31687    avgbs_39040    avgbs_203283    avgbs_103916    avgbs_236390    avgbs_208051    avgbs_66336    avgbs_66337    avgbs_201302    avgbs_204500    avgbs_81721    avgbs_100216    avgbs_207181    avgbs_237978    avgbs_24158    avgbs_233691    avgbs_201875    avgbs_243210    avgbs_53536    avgbs_76860    avgbs_101190    avgbs_237375    avgbs_237798    avgbs_26832    avgbs_45253    avgbs_105606    avgbs_123936    avgbs_225440    avgbs_225385   
   4C 43 
 
  GMI_ES_CC8945_103    GMI_DS_CC1661_180   
  avgbs_200264    avgbs_17972    avgbs_18791    avgbs_66051    avgbs_66052    avgbs_94565    avgbs_13024   
   4C 44 
 
 
  avgbs_205429   
   4C 45 
 
 
  avgbs_5834    avgbs_523    avgbs_122567   
   4C 46 
 
 
  avgbs_217478    avgbs_39533    avgbs_46523   
   4C 47 
 
 
  avgbs_2422    avgbs_6K_30360    avgbs_35234    avgbs_218137    avgbs_104484    avgbs_219718    avgbs_75626   
   4C 48 
 
 
  avgbs_82903    avgbs_224949   
   4C 49 
 
 
  avgbs_61515    avgbs_9244    avgbs_9245    avgbs_16517   
   4C 50 
 
 
  avgbs_68454    avgbs_111455    avgbs_36648    avgbs_123869    avgbs_9034    avgbs_42375    avgbs_228982    avgbs_209964    avgbs_229950    avgbs_36477   
   4C 51 
 
 
  avgbs_36649    avgbs_45070    avgbs_201838    avgbs_4760    avgbs_221389    avgbs_43597    avgbs_36478    avgbs_40233    avgbs_117523    avgbs_43530    avgbs_35301    avgbs_236163    avgbs_67164    avgbs_67165    avgbs_124664   
   4C 52 
 
 
  avgbs_112930    avgbs_79708    avgbs_221042    avgbs_66799    avgbs_93885    avgbs_7684    avgbs_32628    avgbs_77567    avgbs_204763    avgbs_63660    avgbs_64001    avgbs_68802    avgbs_113169    avgbs_113170    avgbs_238139    avgbs_24412    avgbs_27730    avgbs_64522    avgbs_68449    avgbs_68450   
   4C 53 
 
 
  avgbs_43687    avgbs_53315   
   4C 54 
 
 
  avgbs_6K_34931    avgbs_95943    avgbs_74309    avgbs_111321    avgbs_89727    avgbs_240227    avgbs_41897    avgbs_236643   
   4C 55 
 
 
  avgbs_112805    avgbs_40156    avgbs_121763    avgbs_4100    avgbs_2625    avgbs_30946   
   4C 57 
  GMI_ES_CC2132_361    
 
  avgbs_859    avgbs_4774    avgbs_4842    avgbs_9394    avgbs_9396    avgbs_12114    avgbs_14964    avgbs_14968    avgbs_21497    avgbs_22185    avgbs_22641    avgbs_25188    avgbs_25618    avgbs_36924    avgbs_41219    avgbs_42249    avgbs_48121    avgbs_53878    avgbs_58905    avgbs_65135    avgbs_67347    avgbs_68478    avgbs_68832    avgbs_69701    avgbs_70342    avgbs_74224    avgbs_80792    avgbs_94066    avgbs_94168    avgbs_96111    avgbs_97969    avgbs_101497    avgbs_101498    avgbs_105293    avgbs_110312    avgbs_113279    avgbs_115371    avgbs_115559    avgbs_115560    avgbs_116045    avgbs_117846    avgbs_123082    avgbs_123083    avgbs_124135    avgbs_124230    avgbs_124331    avgbs_126181    avgbs_216463    avgbs_201363    avgbs_216592    avgbs_216751    avgbs_202057    avgbs_217509    avgbs_202748    avgbs_217751    avgbs_217772    avgbs_202886    avgbs_218751    avgbs_219034    avgbs_203783    avgbs_219176    avgbs_219917    avgbs_219952    avgbs_220199    avgbs_220217    avgbs_220253    avgbs_205053    avgbs_221262    avgbs_221508    avgbs_221637    avgbs_221752    avgbs_221844    avgbs_221927    avgbs_221960    avgbs_222100    avgbs_222197    avgbs_222229    avgbs_222317    avgbs_206759    avgbs_207433    avgbs_223084    avgbs_223168    avgbs_3521    avgbs_23681    avgbs_28307    avgbs_37233    avgbs_58579    avgbs_71869    avgbs_71870    avgbs_76980    avgbs_85537    avgbs_85868    avgbs_101358    avgbs_108902    avgbs_113640    avgbs_113641    avgbs_124041    avgbs_217030    avgbs_220903    avgbs_87467    avgbs_16449    avgbs_19345    avgbs_38923    avgbs_48252    avgbs_49961    avgbs_115289    avgbs_218760    avgbs_208197    avgbs_55307    avgbs_89753    avgbs_89754    avgbs_93179    avgbs_221139    avgbs_221326    avgbs_65308    avgbs_219710    avgbs_10735    avgbs_10736    avgbs_37937    avgbs_37939    avgbs_47458    avgbs_34591    avgbs_34592    avgbs_36131    avgbs_58795    avgbs_78874    avgbs_78875    avgbs_101820    avgbs_45132    avgbs_45133    avgbs_3156    avgbs_30093    avgbs_35980    avgbs_36492    avgbs_36493    avgbs_43394    avgbs_53955    avgbs_53956    avgbs_71004    avgbs_72942    avgbs_6K_73993    avgbs_112689    avgbs_113029    avgbs_121703    avgbs_122239    avgbs_220041    avgbs_46086    avgbs_53822    avgbs_218628    avgbs_1929    avgbs_6005    avgbs_8071    avgbs_43755    avgbs_47772    avgbs_48899    avgbs_62829    avgbs_87913    avgbs_95708    avgbs_96732    avgbs_111822    avgbs_216406    avgbs_4075    avgbs_40641    avgbs_40642    avgbs_99277    avgbs_211453    avgbs_62111    avgbs_120790    avgbs_72079    avgbs_120661   
   4C 58 
 
 
  avgbs_3365    avgbs_11077    avgbs_15616    avgbs_15617    avgbs_17331    avgbs_18624    avgbs_18625    avgbs_23274    avgbs_26010    avgbs_36297    avgbs_41166    avgbs_42274    avgbs_45071    avgbs_46271    avgbs_48674    avgbs_48675    avgbs_49574    avgbs_57318    avgbs_57660    avgbs_61658    avgbs_65859    avgbs_66770    avgbs_67282    avgbs_68455    avgbs_69893    avgbs_70425    avgbs_75677    avgbs_82318    avgbs_90832    avgbs_97083    avgbs_111821    avgbs_112523    avgbs_118085    avgbs_124246    avgbs_200016    avgbs_216367    avgbs_201909    avgbs_217533    avgbs_217824    avgbs_217833    avgbs_203264    avgbs_218493    avgbs_218636    avgbs_203888    avgbs_219349    avgbs_219812    avgbs_221189    avgbs_221314    avgbs_221735    avgbs_223085    avgbs_34956    avgbs_91980    avgbs_2415    avgbs_13656    avgbs_17022    avgbs_17023    avgbs_32297    avgbs_32300    avgbs_36175    avgbs_40050    avgbs_40809    avgbs_63400    avgbs_72963    avgbs_76503    avgbs_76581    avgbs_86208    avgbs_120125    avgbs_216322    avgbs_220158    avgbs_222859    avgbs_53209    avgbs_53210    avgbs_115288    avgbs_124643    avgbs_218195    avgbs_36328    avgbs_36329    avgbs_69983    avgbs_222072    avgbs_207033    avgbs_15514    avgbs_28800    avgbs_36407    avgbs_37636    avgbs_44832    avgbs_44833    avgbs_66593    avgbs_76928    avgbs_76933    avgbs_77629    avgbs_112721    avgbs_217150    avgbs_219717    avgbs_36130    avgbs_38316    avgbs_51778    avgbs_51779    avgbs_51780    avgbs_69493    avgbs_115778    avgbs_115779    avgbs_119817    avgbs_119818    avgbs_217834    avgbs_8090    avgbs_42893    avgbs_59732    avgbs_65006    avgbs_31010    avgbs_53487    avgbs_66433    avgbs_66597    avgbs_67672    avgbs_89817    avgbs_89818    avgbs_89945    avgbs_110152    avgbs_110153    avgbs_119563    avgbs_219925    avgbs_6231    avgbs_6232    avgbs_12989    avgbs_26677    avgbs_49778    avgbs_22940    avgbs_27403    avgbs_49440    avgbs_49441    avgbs_62616    avgbs_77180    avgbs_77320    avgbs_102761    avgbs_65662    avgbs_15239    avgbs_15240    avgbs_119910    avgbs_119911    avgbs_119912    avgbs_21115    avgbs_21116    avgbs_37595    avgbs_6264    avgbs_3051   
   4C 59 
  GMI_ES15_c6993_447    
 
  avgbs_11205    avgbs_15971    avgbs_25487    avgbs_29720    avgbs_71709    avgbs_88908    avgbs_220224    avgbs_67669    avgbs_94194    avgbs_94195    avgbs_38632    avgbs_74735    avgbs_76601    avgbs_76602    avgbs_217564    avgbs_42476    avgbs_111381    avgbs_41472    avgbs_75160    avgbs_125666    avgbs_218399    avgbs_12639    avgbs_79239    avgbs_105122    avgbs_10056    avgbs_208875    avgbs_233389    avgbs_244279   
   4C 60 
 
 
  avgbs_12134    avgbs_12136    avgbs_53421    avgbs_98795    avgbs_117631    avgbs_106488    avgbs_106489    avgbs_124257    avgbs_232732    avgbs_49589    avgbs_9029    avgbs_84355    avgbs_215102   
   4C 61 
  GMI_DS_A3_213_352     GMI_ES17_c4295_432     GMI_ES17_c4096_846     BA_grs_c10611_259    
 
  avgbs_2269    avgbs_9518    avgbs_11956    avgbs_12317    avgbs_12563    avgbs_12564    avgbs_38666    avgbs_48660    avgbs_49507    avgbs_57030    avgbs_67543    avgbs_70559    avgbs_70574    avgbs_77185    avgbs_89661    avgbs_6K_86477    avgbs_94604    avgbs_96614    avgbs_98592    avgbs_98593    avgbs_100477    avgbs_109220    avgbs_115524    avgbs_116683    avgbs_119403    avgbs_119995    avgbs_122028    avgbs_216753    avgbs_216760    avgbs_216906    avgbs_217079    avgbs_217134    avgbs_219036    avgbs_219324    avgbs_219996    avgbs_220528    avgbs_220835    avgbs_200716    avgbs_222307    avgbs_222615    avgbs_200891    avgbs_57647    avgbs_33859    avgbs_88538    avgbs_105363    avgbs_120870    avgbs_221151    avgbs_222895    avgbs_34769    avgbs_34770    avgbs_3214    avgbs_14059    avgbs_33942    avgbs_69450    avgbs_73850    avgbs_120743    avgbs_124281    avgbs_218181    avgbs_223086    avgbs_7137    avgbs_7138    avgbs_29810    avgbs_36295    avgbs_36296    avgbs_121073    avgbs_16463    avgbs_53320    avgbs_82027    avgbs_88761    avgbs_117223    avgbs_217917    avgbs_19331    avgbs_71474    avgbs_119721    avgbs_24054    avgbs_38341    avgbs_50360    avgbs_54424    avgbs_63063    avgbs_69449    avgbs_77726    avgbs_83345    avgbs_92680    avgbs_92681    avgbs_105149    avgbs_105150    avgbs_43710    avgbs_86178    avgbs_2125    avgbs_20536    avgbs_36018    avgbs_36021    avgbs_46677    avgbs_46680    avgbs_60419    avgbs_60420    avgbs_62039    avgbs_73241    avgbs_74746    avgbs_84032    avgbs_94174    avgbs_218661    avgbs_220249    avgbs_113056    avgbs_24281    avgbs_61694    avgbs_61695    avgbs_66189    avgbs_84678    avgbs_80583    avgbs_80584    avgbs_92955    avgbs_101507    avgbs_25844    avgbs_55003    avgbs_55271    avgbs_219120    avgbs_44668    avgbs_77710    avgbs_80745    avgbs_96615    avgbs_96616    avgbs_230323    avgbs_224274    avgbs_224315    avgbs_224974    avgbs_225803    avgbs_229873    avgbs_36189    avgbs_46676    avgbs_229813    avgbs_69771    avgbs_76274    avgbs_76275    avgbs_87834    avgbs_106927    avgbs_4376    avgbs_13624    avgbs_216429    avgbs_231678    avgbs_87768    avgbs_117224    avgbs_49245    avgbs_11941    avgbs_15694    avgbs_15695    avgbs_39201    avgbs_90428    avgbs_82    avgbs_242592    avgbs_21360    avgbs_89539    avgbs_115457    avgbs_94204    avgbs_8927    avgbs_8928    avgbs_18020    avgbs_18021    avgbs_24576    avgbs_32881    avgbs_36628    avgbs_62857    avgbs_6K_70389    avgbs_77985    avgbs_105369    avgbs_107377    avgbs_107378    avgbs_111980    avgbs_114145    avgbs_120831    avgbs_122921    avgbs_126211    avgbs_209709    avgbs_230654    avgbs_224827    avgbs_223005    avgbs_244520    avgbs_49552    avgbs_107728    avgbs_120500    avgbs_17629    avgbs_40251    avgbs_89752    avgbs_89850    avgbs_99904    avgbs_221832    avgbs_49096    avgbs_67499    avgbs_69653    avgbs_107364    avgbs_219947    avgbs_20762    avgbs_28359    avgbs_67206    avgbs_20543    avgbs_57907    avgbs_57908    avgbs_209327    avgbs_241960    avgbs_102080    avgbs_102081    avgbs_4314    avgbs_114638    avgbs_235855    avgbs_22140    avgbs_52311    avgbs_52312    avgbs_95562    avgbs_115222    avgbs_243174    avgbs_68241    avgbs_85264    avgbs_85265    avgbs_85266    avgbs_111625    avgbs_111626    avgbs_39272    avgbs_42919    avgbs_78162    avgbs_106138    avgbs_54509    avgbs_75700    avgbs_24373    avgbs_41593    avgbs_242516    avgbs_39990    avgbs_39991    avgbs_53664    avgbs_53665    avgbs_53666    avgbs_106188    avgbs_234954    avgbs_43287    avgbs_231110    avgbs_243363    avgbs_26615    avgbs_26616    avgbs_85931    avgbs_89255    avgbs_244220    avgbs_56167    avgbs_67338    avgbs_38010    avgbs_66686    avgbs_229872    avgbs_222723    avgbs_43495    avgbs_43496    avgbs_68596    avgbs_37367    avgbs_50358    avgbs_50359    avgbs_78124    avgbs_242567    avgbs_31105    avgbs_96099    avgbs_25687    avgbs_56215    avgbs_58637    avgbs_58638    avgbs_86223    avgbs_103902    avgbs_40013    avgbs_102387    avgbs_241488    avgbs_68291    avgbs_373    avgbs_374    avgbs_375    avgbs_93514    avgbs_22241    avgbs_75671    avgbs_79401    avgbs_57854    avgbs_47477    avgbs_118435    avgbs_222758    avgbs_125087    avgbs_61509    avgbs_16992    avgbs_16993    avgbs_225344    avgbs_69506    avgbs_69508    avgbs_223441    avgbs_114250    avgbs_225597    avgbs_119987    avgbs_119988    avgbs_119989    avgbs_119990    avgbs_117225    avgbs_36526    avgbs_38012    avgbs_223290    avgbs_48423    avgbs_67139    avgbs_72816    avgbs_81667    avgbs_110380    avgbs_116605    avgbs_116606    avgbs_223791    avgbs_225228    avgbs_225452    avgbs_4432    avgbs_15696    avgbs_55002    avgbs_88137    avgbs_100476    avgbs_223270    avgbs_223463    avgbs_224188    avgbs_224340    avgbs_224599    avgbs_225338    avgbs_32857    avgbs_34902    avgbs_66967    avgbs_75078    avgbs_2943   
   4C 62 
  GMI_ES_CC9014_257     GMI_ES01_c19480_559    
 
  avgbs_13009    avgbs_16432    avgbs_20900    avgbs_36876    avgbs_6K_48174    avgbs_67895    avgbs_92953    avgbs_95520    avgbs_107170    avgbs_107397    avgbs_108904    avgbs_109487    avgbs_124047    avgbs_200340    avgbs_200465    avgbs_200783    avgbs_7418    avgbs_9382    avgbs_12012    avgbs_21824    avgbs_23188    avgbs_23268    avgbs_57985    avgbs_59871    avgbs_101347    avgbs_216629    avgbs_200077    avgbs_219385    avgbs_200539    avgbs_221911    avgbs_12294    avgbs_39882    avgbs_39883    avgbs_71653    avgbs_75115    avgbs_75116    avgbs_78501    avgbs_78502    avgbs_97244    avgbs_103286    avgbs_121537    avgbs_121553    avgbs_122257    avgbs_219403    avgbs_24462    avgbs_113869    avgbs_30228    avgbs_89760    avgbs_123424    avgbs_123425    avgbs_55125    avgbs_55126    avgbs_89966    avgbs_35563    avgbs_35566    avgbs_108911    avgbs_108912    avgbs_117495    avgbs_222706    avgbs_30300    avgbs_54065    avgbs_54066    avgbs_29799    avgbs_105362    avgbs_17560    avgbs_21822    avgbs_42637    avgbs_56396    avgbs_57986    avgbs_62360    avgbs_97003    avgbs_117301    avgbs_229261    avgbs_230262    avgbs_230571    avgbs_231920    avgbs_4392    avgbs_8644    avgbs_9516    avgbs_19224    avgbs_30806    avgbs_35265    avgbs_35266    avgbs_57321    avgbs_58159    avgbs_200038    avgbs_200268    avgbs_7322    avgbs_9755    avgbs_9756    avgbs_44732    avgbs_5416    avgbs_5419    avgbs_15185    avgbs_54898    avgbs_94697    avgbs_221528    avgbs_16168    avgbs_20627    avgbs_21354    avgbs_25153    avgbs_27841    avgbs_36022    avgbs_39845    avgbs_39846    avgbs_39847    avgbs_90429    avgbs_111322    avgbs_230624    avgbs_231159    avgbs_4391    avgbs_35635    avgbs_64140    avgbs_6K_108402    avgbs_208642    avgbs_244090    avgbs_93275    avgbs_1743    avgbs_35636    avgbs_108903    avgbs_108905    avgbs_111433    avgbs_111434    avgbs_209469    avgbs_200569    avgbs_10674    avgbs_16373    avgbs_16417    avgbs_25212    avgbs_88433    avgbs_107340    avgbs_123311    avgbs_123312    avgbs_124972    avgbs_223274    avgbs_223411    avgbs_223115    avgbs_243854    avgbs_34748    avgbs_84911    avgbs_84912    avgbs_47404    avgbs_112508    avgbs_234880    avgbs_54806    avgbs_87790    avgbs_18682    avgbs_8022    avgbs_45704    avgbs_45705    avgbs_78498   
   4C 63 
  GMI_ES15_c222_568     GMI_ES01_lrc10967_551    
 
  avgbs_10909    avgbs_13835    avgbs_21311    avgbs_35036    avgbs_36494    avgbs_51265    avgbs_55929    avgbs_73558    avgbs_73713    avgbs_99999    avgbs_105705    avgbs_108690    avgbs_112456    avgbs_117382    avgbs_216928    avgbs_200146    avgbs_200458    avgbs_219861    avgbs_200538    avgbs_200635    avgbs_200655    avgbs_3242    avgbs_3243    avgbs_22344    avgbs_26666    avgbs_26671    avgbs_31131    avgbs_57984    avgbs_88181    avgbs_65196    avgbs_55602    avgbs_102447    avgbs_209304    avgbs_19810    avgbs_54537    avgbs_120184    avgbs_125683    avgbs_57875    avgbs_49845    avgbs_62716    avgbs_62717    avgbs_90154    avgbs_112329    avgbs_112330    avgbs_3513    avgbs_14344    avgbs_217792    avgbs_28750    avgbs_92890    avgbs_697    avgbs_2074    avgbs_55094    avgbs_59094    avgbs_59095    avgbs_65675    avgbs_76688    avgbs_78129    avgbs_79594    avgbs_79595    avgbs_95600    avgbs_211147    avgbs_211457    avgbs_6544    avgbs_74194    avgbs_78554    avgbs_95601    avgbs_124590    avgbs_216581    avgbs_219060    avgbs_220491    avgbs_33422    avgbs_33423    avgbs_45669    avgbs_66850    avgbs_119952    avgbs_219790    avgbs_102417    avgbs_25655    avgbs_223917    avgbs_14913    avgbs_35100    avgbs_58714    avgbs_230531    avgbs_54860    avgbs_47869    avgbs_17530    avgbs_54840    avgbs_93350    avgbs_36020    avgbs_12133    avgbs_12135    avgbs_15140    avgbs_19259    avgbs_20781    avgbs_26477    avgbs_68779    avgbs_76332    avgbs_84625    avgbs_84626    avgbs_228501    avgbs_113721    avgbs_2    avgbs_1595    avgbs_1998    avgbs_1999    avgbs_4184    avgbs_5574    avgbs_6006    avgbs_6007    avgbs_6606    avgbs_10167    avgbs_14828    avgbs_14829    avgbs_20136    avgbs_20137    avgbs_21009    avgbs_21322    avgbs_21346    avgbs_21788    avgbs_23732    avgbs_24487    avgbs_33630    avgbs_39890    avgbs_40610    avgbs_45595    avgbs_55391    avgbs_64493    avgbs_66423    avgbs_67296    avgbs_68043    avgbs_71714    avgbs_72767    avgbs_75632    avgbs_75913    avgbs_76333    avgbs_77283    avgbs_77917    avgbs_78877    avgbs_78878    avgbs_80694    avgbs_86092    avgbs_86299    avgbs_87530    avgbs_91997    avgbs_104545    avgbs_105382    avgbs_105443    avgbs_106870    avgbs_111123    avgbs_111366    avgbs_112005    avgbs_112085    avgbs_113639    avgbs_113718    avgbs_113783    avgbs_114687    avgbs_115843    avgbs_115844    avgbs_117267    avgbs_121021    avgbs_121022    avgbs_121028    avgbs_121790    avgbs_124150    avgbs_124151    avgbs_124199    avgbs_124680    avgbs_125962    avgbs_233051    avgbs_233297    avgbs_229379    avgbs_202602    avgbs_202686    avgbs_237558    avgbs_238186    avgbs_238962    avgbs_239351    avgbs_239419    avgbs_243082    avgbs_243224    avgbs_21600    avgbs_37299    avgbs_39642    avgbs_77918    avgbs_88890    avgbs_93780    avgbs_112346    avgbs_121281    avgbs_123688    avgbs_240669    avgbs_243057    avgbs_20831    avgbs_20897    avgbs_80637    avgbs_86114    avgbs_111186    avgbs_111337    avgbs_111646    avgbs_122996    avgbs_124380    avgbs_124381    avgbs_124446    avgbs_235230    avgbs_208246    avgbs_34590    avgbs_53021    avgbs_104687    avgbs_118432    avgbs_204793    avgbs_182    avgbs_1913    avgbs_8172    avgbs_8173    avgbs_50519    avgbs_60707    avgbs_70760    avgbs_70762    avgbs_75910    avgbs_77986    avgbs_201343    avgbs_37285    avgbs_81636    avgbs_84568    avgbs_205898    avgbs_2621    avgbs_30424    avgbs_30425    avgbs_30426    avgbs_39466    avgbs_47144    avgbs_47145    avgbs_65694    avgbs_102813    avgbs_107266    avgbs_234041    avgbs_38631    avgbs_39358    avgbs_41390    avgbs_88435    avgbs_89847    avgbs_89848    avgbs_114686    avgbs_202187    avgbs_3144    avgbs_9926    avgbs_15589    avgbs_38922    avgbs_42143    avgbs_85073    avgbs_105722    avgbs_234016    avgbs_236654    avgbs_205281    avgbs_200    avgbs_27731    avgbs_47397    avgbs_49572    avgbs_81245    avgbs_81247    avgbs_242969    avgbs_33768    avgbs_33769    avgbs_76535    avgbs_123147    avgbs_31601    avgbs_49573    avgbs_85051    avgbs_88527    avgbs_62913    avgbs_83620    avgbs_94508    avgbs_38911    avgbs_80616    avgbs_234875    avgbs_10029    avgbs_48797    avgbs_48798    avgbs_57962    avgbs_112550    avgbs_112551    avgbs_233803    avgbs_239468    avgbs_22653    avgbs_22654    avgbs_61741    avgbs_233733    avgbs_32540    avgbs_108254    avgbs_44008    avgbs_72960    avgbs_72961    avgbs_107961    avgbs_236701    avgbs_79163    avgbs_100326    avgbs_116948    avgbs_236587    avgbs_243283    avgbs_222680    avgbs_34177    avgbs_41387    avgbs_83308    avgbs_119639    avgbs_218550    avgbs_239512    avgbs_45594    avgbs_92994    avgbs_235194    avgbs_58715    avgbs_8591    avgbs_18928    avgbs_207879    avgbs_86077    avgbs_235508    avgbs_45408    avgbs_45409    avgbs_4568    avgbs_4569    avgbs_244091    avgbs_236223    avgbs_4109   
   4C 64 
  GMI_DS_CC11029_65    
 
  avgbs_17503    avgbs_18018    avgbs_21789    avgbs_200120    avgbs_118589    avgbs_57265    avgbs_2768    avgbs_21155    avgbs_35037    avgbs_52452    avgbs_15622    avgbs_30776    avgbs_41208    avgbs_41209    avgbs_44055    avgbs_77744    avgbs_596    avgbs_18498    avgbs_56059    avgbs_71814    avgbs_6K_112664    avgbs_6035    avgbs_30114    avgbs_51438    avgbs_200336    avgbs_16328    avgbs_75635    avgbs_102086    avgbs_51013    avgbs_72230    avgbs_49297    avgbs_210167    avgbs_9954    avgbs_9955    avgbs_236275    avgbs_92998    avgbs_233731    avgbs_74687    avgbs_119574    avgbs_119575    avgbs_123443    avgbs_16102    avgbs_47557    avgbs_101049    avgbs_241311    avgbs_6749    avgbs_68067    avgbs_230553    avgbs_35035    avgbs_106980    avgbs_235185    avgbs_87952    avgbs_244002    avgbs_88273    avgbs_118060    avgbs_233800    avgbs_234103    avgbs_27977    avgbs_38215    avgbs_38725    avgbs_235150    avgbs_237388    avgbs_21358    avgbs_242979    avgbs_242604    avgbs_236157    avgbs_117503    avgbs_16830    avgbs_17077    avgbs_240931   
   4C 65 
 
 
  avgbs_27942    avgbs_225545   
   4C 67 
 
 
  avgbs_20723    avgbs_20724    avgbs_448    avgbs_449    avgbs_32283    avgbs_32284    avgbs_47602    avgbs_114507    avgbs_6079    avgbs_233510    avgbs_203658    avgbs_235205    avgbs_59678    avgbs_88546    avgbs_102436    avgbs_102437    avgbs_76552    avgbs_38472    avgbs_38473    avgbs_233211    avgbs_238286    avgbs_233237    avgbs_242884    avgbs_94575    avgbs_36266    avgbs_29324    avgbs_243848   
   4C 68 
 
 
  avgbs_111030    avgbs_8261    avgbs_50446    avgbs_75295   
   4C 69 
 
 
  avgbs_224113   
   4C 70 
  GMI_ES01_c5419_407    
 
  avgbs_200385    avgbs_64168    avgbs_200433    avgbs_225833    avgbs_29065    avgbs_29067    avgbs_67486    avgbs_15499    avgbs_6K_57848    avgbs_6830    avgbs_122448    avgbs_222976    avgbs_37620    avgbs_216054    avgbs_66362    avgbs_44924    avgbs_44925    avgbs_39881   
   4C 72 
 
 
  avgbs_104583    avgbs_12516    avgbs_72446    avgbs_35987    avgbs_60219   
   4C 73 
 
 
  avgbs_120996   
   4C 74 
 
 
  avgbs_6134    avgbs_6135    avgbs_51823    avgbs_49840    avgbs_49841    avgbs_62678   
   4C 75 
  GMI_DS_CC7482_102     GMI_DS_CC9295_235    
 
  avgbs_21741    avgbs_6K_86251    avgbs_218621    avgbs_215413    avgbs_9099    avgbs_50311    avgbs_224588    avgbs_116510    avgbs_37462    avgbs_47778    avgbs_60871    avgbs_85340    avgbs_85341    avgbs_97264    avgbs_97265    avgbs_97369    avgbs_103444    avgbs_108983    avgbs_200252    avgbs_205098    avgbs_224985    avgbs_200658    avgbs_205734    avgbs_200753    avgbs_200878    avgbs_20045    avgbs_34205    avgbs_34437    avgbs_68148    avgbs_125423    avgbs_200053    avgbs_202979    avgbs_8134    avgbs_108731    avgbs_1930    avgbs_6K_17395    avgbs_59674    avgbs_90007    avgbs_90008    avgbs_102446    avgbs_114294    avgbs_37479    avgbs_213911    avgbs_109781    avgbs_204037    avgbs_98808    avgbs_15111    avgbs_214673    avgbs_156    avgbs_21740    avgbs_200954    avgbs_206165    avgbs_9569    avgbs_42832    avgbs_93699    avgbs_108820    avgbs_203341    avgbs_73695    avgbs_3207    avgbs_41088   
   4C 76 
 
 
  avgbs_216860    avgbs_200251    avgbs_200535    avgbs_46758    avgbs_64990    avgbs_72292    avgbs_30874    avgbs_229670    avgbs_9092    avgbs_11114    avgbs_225835    avgbs_203692   
   4C 77 
 
 
  avgbs_6682    avgbs_32026    avgbs_32027    avgbs_42926    avgbs_200284    avgbs_200590    avgbs_222053    avgbs_200218    avgbs_111941    avgbs_17454    avgbs_81055    avgbs_22470    avgbs_100590    avgbs_47776    avgbs_46464    avgbs_117747    avgbs_225672    avgbs_97069   
   4C 78 
 
 
  avgbs_9038    avgbs_9091    avgbs_38509    avgbs_213939    avgbs_84974    avgbs_46778    avgbs_236242    avgbs_119439    avgbs_13265   
   4C 79 
 
 
  avgbs_1404    avgbs_50312    avgbs_50313    avgbs_103135    avgbs_103282    avgbs_221421    avgbs_200679    avgbs_1780    avgbs_10733    avgbs_76983    avgbs_216624    avgbs_62055    avgbs_219531    avgbs_225653    avgbs_32025   
   4C 80 
 
 
  avgbs_19909    avgbs_55644    avgbs_57704    avgbs_84999    avgbs_85000    avgbs_6K_92039    avgbs_221623    avgbs_110823    avgbs_114692    avgbs_122784    avgbs_38285    avgbs_90543    avgbs_104509    avgbs_7140    avgbs_124776    avgbs_84930    avgbs_84932    avgbs_206733    avgbs_225671    avgbs_73306    avgbs_8464   
   4C 81 
  GMI_ES02_c2988_293     GMI_ES15_c3169_222    
 
  avgbs_200110    avgbs_459    avgbs_42119    avgbs_83016    avgbs_83017    avgbs_122760    avgbs_71250    avgbs_95605    avgbs_122571    avgbs_217977    avgbs_200676    avgbs_17649    avgbs_14813    avgbs_14814    avgbs_41125    avgbs_80766    avgbs_217925    avgbs_6681    avgbs_93612    avgbs_117009    avgbs_31965    avgbs_31966    avgbs_31967    avgbs_56838    avgbs_6572    avgbs_14149    avgbs_48562    avgbs_944    avgbs_47744    avgbs_117109    avgbs_117110    avgbs_39290    avgbs_109613    avgbs_109614    avgbs_126319    avgbs_14787    avgbs_116153    avgbs_235370    avgbs_14788    avgbs_76621    avgbs_236156    avgbs_11693    avgbs_11694    avgbs_15719    avgbs_241454    avgbs_232327    avgbs_36402    avgbs_48561    avgbs_12515    avgbs_234245    avgbs_21765    avgbs_200587    avgbs_9268    avgbs_9392    avgbs_17698    avgbs_19908    avgbs_35607    avgbs_42360    avgbs_48592    avgbs_48593    avgbs_81232    avgbs_94169    avgbs_96082    avgbs_97180    avgbs_97181    avgbs_107664    avgbs_109850    avgbs_115450    avgbs_120047    avgbs_223207    avgbs_200050    avgbs_201595    avgbs_223606    avgbs_200216    avgbs_200281    avgbs_224158    avgbs_200662    avgbs_200681    avgbs_200708    avgbs_200800    avgbs_200893    avgbs_97806    avgbs_111264    avgbs_3208    avgbs_8669    avgbs_98407    avgbs_107597    avgbs_107221    avgbs_210112    avgbs_12037    avgbs_25579    avgbs_63014    avgbs_63015    avgbs_224254   
   4C 82 
 
 
  avgbs_116128    avgbs_74697    avgbs_211021    avgbs_46525    avgbs_18713    avgbs_232508    avgbs_123809    avgbs_7109    avgbs_7110    avgbs_55971   
   4C 83 
 
 
  avgbs_15288    avgbs_57641    avgbs_78102    avgbs_101162    avgbs_115377    avgbs_7992    avgbs_20822    avgbs_70585    avgbs_121353    avgbs_121354    avgbs_101690    avgbs_200734    avgbs_103762    avgbs_95543    avgbs_122783    avgbs_213652    avgbs_66434    avgbs_66435    avgbs_115709    avgbs_243247    avgbs_4830    avgbs_116834    avgbs_200246    avgbs_108044    avgbs_46020    avgbs_31540   
   4C 84 
 
 
  avgbs_14815    avgbs_64183    avgbs_64414    avgbs_64822    avgbs_77377    avgbs_87936    avgbs_94495    avgbs_113632    avgbs_125110    avgbs_217860    avgbs_219337    avgbs_200436    avgbs_219687    avgbs_220989    avgbs_200619    avgbs_223121    avgbs_14615    avgbs_20143    avgbs_23012    avgbs_44667    avgbs_110750    avgbs_110751    avgbs_217399    avgbs_23013    avgbs_115465    avgbs_26841    avgbs_27954    avgbs_121776    avgbs_80750    avgbs_9072    avgbs_98997    avgbs_98998    avgbs_98999    avgbs_200541    avgbs_13444    avgbs_101210    avgbs_54887    avgbs_100759    avgbs_84899    avgbs_9279   
   4C 85 
  GMI_ES15_c7179_388    
 
  avgbs_9486    avgbs_90170    avgbs_96604    avgbs_200450    avgbs_755    avgbs_12068    avgbs_33119    avgbs_90645    avgbs_90646    avgbs_200607    avgbs_3993    avgbs_3994    avgbs_3995    avgbs_52111    avgbs_97442    avgbs_218344    avgbs_114514    avgbs_200811    avgbs_225854    avgbs_57246    avgbs_82428    avgbs_10734    avgbs_215534   
   4C 86 
 
 
  avgbs_29855    avgbs_109100    avgbs_1591    avgbs_29564    avgbs_54503    avgbs_6K_78024    avgbs_84659    avgbs_105842    avgbs_217680    avgbs_200183    avgbs_218023    avgbs_214762    avgbs_46224    avgbs_32461    avgbs_30388    avgbs_220703    avgbs_115741    avgbs_117003    avgbs_109101    avgbs_14683    avgbs_49593    avgbs_64976   
   4C 87 
 
 
  avgbs_66993    avgbs_99729    avgbs_108537    avgbs_112972    avgbs_215174    avgbs_221823    avgbs_222007    avgbs_20474    avgbs_20475    avgbs_97665    avgbs_12971    avgbs_12972    avgbs_12973    avgbs_81580    avgbs_100594    avgbs_216766    avgbs_59192    avgbs_11316    avgbs_79460    avgbs_87260    avgbs_92931    avgbs_92932    avgbs_115174    avgbs_43296    avgbs_67324    avgbs_74317    avgbs_86257    avgbs_108790    avgbs_108791    avgbs_108792    avgbs_225350    avgbs_117221    avgbs_122572    avgbs_36662    avgbs_122882    avgbs_98003    avgbs_44666    avgbs_200293    avgbs_6228   
   4C 88 
 
 
  avgbs_12761    avgbs_19713    avgbs_39658    avgbs_62784    avgbs_75325    avgbs_80351    avgbs_86258    avgbs_99707    avgbs_99708    avgbs_99784    avgbs_106955    avgbs_121068    avgbs_121069    avgbs_216583    avgbs_200083    avgbs_200195    avgbs_200890    avgbs_6K_29354    avgbs_53548    avgbs_83185    avgbs_83503    avgbs_85522    avgbs_22417    avgbs_95559    avgbs_119400    avgbs_12357    avgbs_21757    avgbs_21758    avgbs_21759    avgbs_83256    avgbs_94184    avgbs_6277    avgbs_10518    avgbs_38414    avgbs_38415    avgbs_60530    avgbs_200396    avgbs_214750    avgbs_225621    avgbs_54998    avgbs_1897    avgbs_61490    avgbs_86868    avgbs_227683    avgbs_39659    avgbs_200266    avgbs_1934    avgbs_47335    avgbs_224705    avgbs_57687    avgbs_57689   
   4C 89 
 
 
  avgbs_12872    avgbs_41886    avgbs_44679    avgbs_115135    avgbs_99061    avgbs_99062    avgbs_111214    avgbs_67293    avgbs_38116    avgbs_58322    avgbs_93193    avgbs_108219    avgbs_108220    avgbs_8629    avgbs_126317    avgbs_126318    avgbs_4995    avgbs_4996    avgbs_99945    avgbs_26318    avgbs_2351    avgbs_111545    avgbs_26533    avgbs_120124    avgbs_223539   
   4C 90 
 
 
  avgbs_2800    avgbs_3228    avgbs_3229    avgbs_3230    avgbs_11235    avgbs_11236    avgbs_117568    avgbs_117569    avgbs_45593    avgbs_15186   
   4C 91 
  GMI_ES_CC11649_76    
  GMI_DS_CC10009_162   
  avgbs_16450    avgbs_32581    avgbs_99390    avgbs_213803    avgbs_112319    avgbs_30381    avgbs_231048    avgbs_206038    avgbs_51053    avgbs_66401    avgbs_214521    avgbs_214324    avgbs_14529    avgbs_98562    avgbs_86730    avgbs_123741    avgbs_65700    avgbs_200945    avgbs_215605    avgbs_10127    avgbs_19011    avgbs_75499    avgbs_89172    avgbs_215221   
   4C 92 
 
 
  avgbs_43233    avgbs_87199    avgbs_108022    avgbs_117855    avgbs_200761    avgbs_213788    avgbs_6K_58274    avgbs_62932    avgbs_62933    avgbs_118082    avgbs_48501    avgbs_48502    avgbs_6K_85746    avgbs_229536    avgbs_200345   
   4C 93 
 
 
  avgbs_10128    avgbs_19010    avgbs_60441    avgbs_103360    avgbs_111033    avgbs_111034    avgbs_114398    avgbs_13642    avgbs_101806    avgbs_206073    avgbs_64821   
   4C 94 
 
 
  avgbs_1165    avgbs_10589    avgbs_10590    avgbs_10591    avgbs_10598    avgbs_10599    avgbs_18179    avgbs_23203    avgbs_53894    avgbs_76543    avgbs_91936    avgbs_200006    avgbs_217074    avgbs_200156    avgbs_200361    avgbs_200368    avgbs_214896    avgbs_204859    avgbs_200729    avgbs_120018    avgbs_222851    avgbs_113883    avgbs_113884    avgbs_119953    avgbs_119954    avgbs_99019    avgbs_70241   
   4C 95 
 
  GMI_ES_CC9220_300   
  avgbs_2850    avgbs_108450    avgbs_108451    avgbs_121518    avgbs_100429    avgbs_100430    avgbs_55186    avgbs_107532    avgbs_107533    avgbs_21804    avgbs_113006   
   4C 96 
 
 
  avgbs_44783    avgbs_63930    avgbs_40295   
   4C 97 
  GMI_ES02_c13068_328    
 
  avgbs_39104    avgbs_224133    avgbs_8017    avgbs_81486    avgbs_205854    avgbs_30800    avgbs_91525    avgbs_225146    avgbs_79970    avgbs_79971    avgbs_53602    avgbs_20244    avgbs_32904    avgbs_200136    avgbs_200230    avgbs_224280    avgbs_224843    avgbs_96255   
   4C 98 
 
 
  avgbs_224982    avgbs_99730    avgbs_200044   
   4C 100 
 
 
  avgbs_94690   
   4C 101 
 
 
  avgbs_42890    avgbs_222071    avgbs_88567    avgbs_42874   
   4C 102 
 
 
  avgbs_72437    avgbs_72438    avgbs_75500    avgbs_109186    avgbs_111284    avgbs_111285    avgbs_121512    avgbs_218004    avgbs_220303    avgbs_98648    avgbs_98649    avgbs_60011    avgbs_210515   
   4C 103 
 
 
  avgbs_11475    avgbs_11476    avgbs_87520    avgbs_204926    avgbs_67963    avgbs_120947    avgbs_12547    avgbs_89695    avgbs_8178    avgbs_8179    avgbs_49214   
   4C 104 
 
 
  avgbs_43262    avgbs_43263    avgbs_45563    avgbs_45564    avgbs_123845   
   4C 105 
 
 
  avgbs_28721    avgbs_28722    avgbs_200827    avgbs_118604    avgbs_16324   
   4C 106 
 
 
  avgbs_113908    avgbs_200059    avgbs_102824    avgbs_33091    avgbs_59273    avgbs_119420    avgbs_72978    avgbs_72979   
   4C 107 
 
 
  avgbs_11846    avgbs_85097    avgbs_200597    avgbs_83303    avgbs_19880    avgbs_68079    avgbs_83305    avgbs_42789    avgbs_1374    avgbs_57931    avgbs_100815    avgbs_100816   
   4C 108 
 
 
  avgbs_16846    avgbs_16847    avgbs_200725    avgbs_8897    avgbs_80575    avgbs_217032    avgbs_86093    avgbs_114938    avgbs_114939    avgbs_119783    avgbs_11081    avgbs_57637    avgbs_7613    avgbs_26004   
   4C 109 
 
 
  avgbs_57537    avgbs_9432    avgbs_14103    avgbs_200092    avgbs_55130    avgbs_55131    avgbs_27930    avgbs_200700   
   4C 110 
 
 
  avgbs_84620    avgbs_84621    avgbs_84622    avgbs_14807    avgbs_14808    avgbs_72756    avgbs_110939    avgbs_121326    avgbs_200921    avgbs_79489    avgbs_91064    avgbs_91065    avgbs_200391    avgbs_10918    avgbs_10919   
   4C 111 
 
 
  avgbs_49658    avgbs_200188    avgbs_79490    avgbs_200278    avgbs_14475    avgbs_57635    avgbs_57636    avgbs_76382    avgbs_76383    avgbs_110891    avgbs_110892   
   4C 112 
 
 
  avgbs_122179    avgbs_78867    avgbs_114309    avgbs_114310   
   4C 113 
 
 
  avgbs_106026    avgbs_112775   
 
 
   Chromosome 5C    Chr   Pos   Framework   Placed SNP  Placed GBS  
   5C -16 
 
 
  avgbs_52659    avgbs_21618   
   5C -15 
 
 
  avgbs_102165    avgbs_32759    avgbs_15178   
   5C -14 
 
 
  avgbs_71135   
   5C -13 
 
 
  avgbs_213052   
   5C -12 
 
 
  avgbs_7199    avgbs_63268    avgbs_73244    avgbs_73245    avgbs_224566   
   5C -11 
 
 
  avgbs_87990    avgbs_235486   
   5C -10 
 
 
  avgbs_110756    avgbs_6K_57836    avgbs_9090    avgbs_74977    avgbs_105828   
   5C -9 
 
 
  avgbs_76024    avgbs_76025   
   5C -8 
 
  GMI_ES02_c5703_238   
  avgbs_11096    avgbs_49703   
   5C -7 
 
 
  avgbs_14040    avgbs_109203    avgbs_223349    avgbs_34978   
   5C -5 
 
 
  avgbs_38194    avgbs_81402    avgbs_14041    avgbs_14042    avgbs_225390    avgbs_38195    avgbs_48059   
   5C -4 
 
 
  avgbs_116483   
   5C -3 
 
 
  avgbs_215925    avgbs_123169    avgbs_12619    avgbs_223454   
   5C -2 
 
 
  avgbs_204673   
   5C -1 
 
 
  avgbs_94591    avgbs_221460    avgbs_78713    avgbs_78714    avgbs_73544    avgbs_102575   
   5C 0 
  GMI_ES17_c5197_503    
 
  avgbs_103180    avgbs_4806    avgbs_16044    avgbs_86859    avgbs_85698    avgbs_225441    avgbs_100158    avgbs_210609   
   5C 3 
  GMI_ES02_c10836_312    
 
  avgbs_73381    avgbs_65094    avgbs_65096    avgbs_238076    avgbs_43772    avgbs_43773   
   5C 4 
 
 
  avgbs_203370    avgbs_21700    avgbs_107043    avgbs_21698    avgbs_10562    avgbs_1228    avgbs_223195    avgbs_214958   
   5C 5 
 
 
  avgbs_6K_15811    avgbs_23865    avgbs_23866    avgbs_54357    avgbs_54358    avgbs_75646    avgbs_103695    avgbs_216380    avgbs_220891    avgbs_18479    avgbs_86121   
   5C 6 
  GMI_ES02_c26223_268     GMI_ES17_c1186_142     GMI_ES15_c6191_370    
  GMI_ES15_c11166_228    GMI_ES01_c7940_496   
  avgbs_7198    avgbs_7200    avgbs_17340    avgbs_17341    avgbs_23745    avgbs_33904    avgbs_41343    avgbs_63269    avgbs_96443    avgbs_103126    avgbs_110713    avgbs_119048    avgbs_120934    avgbs_201229    avgbs_202656    avgbs_218511    avgbs_204523    avgbs_216052    avgbs_65093    avgbs_220054    avgbs_124601    avgbs_116598    avgbs_42733    avgbs_6982    avgbs_9670    avgbs_9671    avgbs_50847    avgbs_55733    avgbs_115231    avgbs_218883    avgbs_50656    avgbs_53030    avgbs_53031    avgbs_79186    avgbs_61216    avgbs_73805    avgbs_81618    avgbs_203981    avgbs_69365    avgbs_47272    avgbs_47273    avgbs_14855    avgbs_216034    avgbs_102094    avgbs_123870    avgbs_123871    avgbs_244699    avgbs_21701    avgbs_238377    avgbs_116076    avgbs_30069    avgbs_107116    avgbs_4798    avgbs_20467    avgbs_20468    avgbs_20469    avgbs_48840    avgbs_107598    avgbs_35319    avgbs_69382    avgbs_242496    avgbs_42072    avgbs_110811    avgbs_124913    avgbs_71616    avgbs_239067    avgbs_95391    avgbs_95392    avgbs_52733    avgbs_80810    avgbs_80811    avgbs_113776    avgbs_113777    avgbs_124395    avgbs_219709    avgbs_37949    avgbs_37950    avgbs_37951    avgbs_40350    avgbs_51876    avgbs_51877    avgbs_106055    avgbs_235178    avgbs_65214    avgbs_107273    avgbs_243231    avgbs_50655    avgbs_71925    avgbs_33289    avgbs_43928    avgbs_43929    avgbs_78393    avgbs_44098    avgbs_65297    avgbs_42297    avgbs_23852    avgbs_74915    avgbs_216793    avgbs_16261    avgbs_68089    avgbs_68090    avgbs_53540    avgbs_53541    avgbs_53542    avgbs_102830    avgbs_32369    avgbs_89705    avgbs_78152    avgbs_78153    avgbs_9126    avgbs_10018    avgbs_85762    avgbs_21699   
   5C 7 
  GMI_ES02_c15089_196    
 
  avgbs_73875    avgbs_15951    avgbs_122622    avgbs_208047    avgbs_28190    avgbs_28191    avgbs_37979    avgbs_37980    avgbs_40248    avgbs_100354    avgbs_108172    avgbs_114524    avgbs_203236    avgbs_203580    avgbs_109074    avgbs_53033    avgbs_55119    avgbs_121169    avgbs_32726    avgbs_117667    avgbs_219545    avgbs_22390    avgbs_65334    avgbs_37732    avgbs_75413    avgbs_75414    avgbs_75415    avgbs_228446    avgbs_125504    avgbs_5485    avgbs_5486    avgbs_244697    avgbs_207422    avgbs_85937    avgbs_16247    avgbs_43107    avgbs_17952    avgbs_99238    avgbs_99239    avgbs_244489    avgbs_60472    avgbs_122754    avgbs_241148    avgbs_64948    avgbs_10273    avgbs_238089    avgbs_206752    avgbs_86693    avgbs_233707   
   5C 8 
  GMI_ES01_c1223_200    
 
  avgbs_6K_73494    avgbs_125420    avgbs_204775    avgbs_113864    avgbs_65529    avgbs_231481    avgbs_3211    avgbs_50294    avgbs_58542    avgbs_96206    avgbs_108574    avgbs_214211    avgbs_108473    avgbs_67348    avgbs_67349    avgbs_8080    avgbs_44176    avgbs_77869   
   5C 9 
 
 
  avgbs_78500    avgbs_204829    avgbs_26530    avgbs_26531    avgbs_50746    avgbs_98266    avgbs_205840    avgbs_70540    avgbs_113536    avgbs_242902    avgbs_243461    avgbs_236423    avgbs_126130    avgbs_237682    avgbs_91646    avgbs_235441   
   5C 10 
 
 
  avgbs_21517    avgbs_21519    avgbs_230325    avgbs_7809    avgbs_7810    avgbs_7811    avgbs_100919    avgbs_224563    avgbs_76400   
   5C 11 
  GMI_ES15_c12818_361     GMI_ES15_c6652_253     GMI_ES02_c15112_271     GMI_ES_CC16529_138    
 
  avgbs_22423    avgbs_22425    avgbs_54362    avgbs_229167    avgbs_230365    avgbs_115261    avgbs_41225    avgbs_235293    avgbs_214139    avgbs_66852    avgbs_10913    avgbs_97250    avgbs_103201    avgbs_6K_107491    avgbs_241561    avgbs_16748    avgbs_16749    avgbs_121654    avgbs_121655    avgbs_9539    avgbs_9540    avgbs_65309    avgbs_13481    avgbs_121566    avgbs_206650    avgbs_47876    avgbs_66864    avgbs_66865    avgbs_111740    avgbs_205765    avgbs_206927    avgbs_120103    avgbs_10942    avgbs_76592    avgbs_4504    avgbs_63341    avgbs_69537    avgbs_205558    avgbs_15257    avgbs_15258    avgbs_79082    avgbs_79083    avgbs_2286    avgbs_16125    avgbs_16126    avgbs_21561    avgbs_201068    avgbs_200150    avgbs_221088    avgbs_2002    avgbs_30169    avgbs_120405    avgbs_125885    avgbs_205820    avgbs_203793    avgbs_7733    avgbs_104574    avgbs_104575    avgbs_114941    avgbs_201011    avgbs_13399    avgbs_33080    avgbs_204073    avgbs_20152    avgbs_20153    avgbs_20687    avgbs_29625    avgbs_63607    avgbs_108681    avgbs_115650    avgbs_69900    avgbs_96308    avgbs_205795    avgbs_243631    avgbs_5367    avgbs_204530    avgbs_57442    avgbs_60053    avgbs_60054    avgbs_202277    avgbs_24705    avgbs_201848    avgbs_100983    avgbs_208309    avgbs_83946    avgbs_105830    avgbs_240213    avgbs_241336    avgbs_48169    avgbs_57375    avgbs_57376    avgbs_22702   
   5C 12 
  GMI_ES15_c6914_663     GMI_ES01_c11975_322     GMI_ES15_c12600_230    
 
  avgbs_11643    avgbs_87840    avgbs_215228    avgbs_120122    avgbs_67011    avgbs_204234    avgbs_36037    avgbs_36038    avgbs_36039    avgbs_30522    avgbs_30523    avgbs_30524    avgbs_101784    avgbs_223451    avgbs_224530    avgbs_39723    avgbs_237479    avgbs_78482    avgbs_78483    avgbs_66879    avgbs_30250    avgbs_91850    avgbs_123018    avgbs_225794    avgbs_20798    avgbs_20805    avgbs_14408   
   5C 13 
  GMI_ES01_c12564_210     GMI_ES_CC11658_395     GMI_ES01_c9170_468     GMI_DS_CC892_260    
 
  avgbs_26873    avgbs_30570    avgbs_30571    avgbs_78208    avgbs_98692    avgbs_98693    avgbs_200207    avgbs_223993    avgbs_95028    avgbs_211602    avgbs_232462    avgbs_25390    avgbs_202727    avgbs_67816    avgbs_27983    avgbs_93349    avgbs_41676    avgbs_56770    avgbs_69289    avgbs_78741    avgbs_1788    avgbs_64543    avgbs_201044    avgbs_224543    avgbs_224931    avgbs_4525    avgbs_82148    avgbs_16072    avgbs_16073    avgbs_25187    avgbs_82518    avgbs_112268    avgbs_58699    avgbs_58700    avgbs_90726    avgbs_224398    avgbs_61346    avgbs_214489    avgbs_223801    avgbs_79464    avgbs_23701    avgbs_52980    avgbs_58206    avgbs_85086    avgbs_85087    avgbs_118092    avgbs_224266    avgbs_224520    avgbs_225002    avgbs_225180    avgbs_225682    avgbs_4251    avgbs_57392    avgbs_114943    avgbs_223580    avgbs_225614    avgbs_3089    avgbs_13965    avgbs_13966    avgbs_69290    avgbs_77350    avgbs_211117    avgbs_82289    avgbs_101817    avgbs_52271   
   5C 14 
  GMI_ES01_c6298_257     GMI_ES02_c28204_255     GMI_ES01_c22998_155     GMI_ES15_c5451_344     GMI_ES01_lrc8457_64     GMI_ES01_c11126_277     GMI_ES15_c12436_395     GMI_ES02_c4756_515     GMI_ES15_c3159_412     GMI_ES01_c4174_228     GMI_DS_CC6107_131    
 
  avgbs_31532    avgbs_39844    avgbs_53971    avgbs_79364    avgbs_80466    avgbs_96757    avgbs_102422    avgbs_121552    avgbs_124125    avgbs_231224    avgbs_200562    avgbs_206115    avgbs_110854    avgbs_230171    avgbs_125691    avgbs_2420    avgbs_98412    avgbs_21300    avgbs_83347    avgbs_104758    avgbs_122679    avgbs_48341    avgbs_122680    avgbs_55078    avgbs_203785    avgbs_86663    avgbs_86664    avgbs_82071    avgbs_122434    avgbs_18632    avgbs_71835    avgbs_60880    avgbs_85475    avgbs_121455    avgbs_121456    avgbs_236109    avgbs_210545    avgbs_211427    avgbs_216    avgbs_85395    avgbs_8467    avgbs_69899    avgbs_75803    avgbs_201532    avgbs_203715    avgbs_120450    avgbs_316    avgbs_1874    avgbs_32468    avgbs_32835    avgbs_96004    avgbs_113192    avgbs_113193    avgbs_118081    avgbs_125255    avgbs_235982    avgbs_206072    avgbs_200939    avgbs_9834    avgbs_11097    avgbs_18633    avgbs_52931    avgbs_204138    avgbs_4488    avgbs_18136    avgbs_33010    avgbs_66997    avgbs_74146    avgbs_74210    avgbs_89158    avgbs_96467    avgbs_108502    avgbs_112697    avgbs_115199    avgbs_119341    avgbs_201220    avgbs_201244    avgbs_202183    avgbs_223592    avgbs_200231    avgbs_204558    avgbs_5541    avgbs_21592    avgbs_26296    avgbs_26297    avgbs_31407    avgbs_33988    avgbs_33989    avgbs_2348    avgbs_4751    avgbs_7020    avgbs_7038    avgbs_7076    avgbs_7078    avgbs_8297    avgbs_6K_9676    avgbs_15925    avgbs_32356    avgbs_33441    avgbs_33442    avgbs_34489    avgbs_37065    avgbs_49502    avgbs_49883    avgbs_49884    avgbs_75106    avgbs_83647    avgbs_85901    avgbs_85902    avgbs_91787    avgbs_96955    avgbs_100130    avgbs_101757    avgbs_103123    avgbs_103124    avgbs_104666    avgbs_107637    avgbs_107638    avgbs_107656    avgbs_107657    avgbs_115254    avgbs_115954    avgbs_116012    avgbs_121692    avgbs_121693    avgbs_121694    avgbs_122908    avgbs_234595    avgbs_202700    avgbs_202898    avgbs_240258    avgbs_205781    avgbs_205813    avgbs_241132    avgbs_206040    avgbs_241987    avgbs_215770    avgbs_207521    avgbs_13423    avgbs_13424    avgbs_20826    avgbs_20827    avgbs_104759    avgbs_9848    avgbs_13972    avgbs_20797    avgbs_20799    avgbs_30979    avgbs_31417    avgbs_36117    avgbs_36118    avgbs_39849    avgbs_44357    avgbs_47533    avgbs_52542    avgbs_60811    avgbs_64265    avgbs_67272    avgbs_69231    avgbs_69232    avgbs_69756    avgbs_71428    avgbs_83026    avgbs_85005    avgbs_85006    avgbs_86510    avgbs_96682    avgbs_97041    avgbs_97664    avgbs_104952    avgbs_104953    avgbs_107936    avgbs_109678    avgbs_114245    avgbs_114531    avgbs_114915    avgbs_115374    avgbs_116284    avgbs_116511    avgbs_117216    avgbs_121869    avgbs_125477    avgbs_201821    avgbs_235739    avgbs_202839    avgbs_202892    avgbs_200316    avgbs_237030    avgbs_237868    avgbs_224534    avgbs_205073    avgbs_215101    avgbs_240694    avgbs_200694    avgbs_99133    avgbs_207195    avgbs_207634    avgbs_208169    avgbs_12129    avgbs_12130    avgbs_12131    avgbs_31089    avgbs_34501    avgbs_6K_103122    avgbs_112477    avgbs_200365    avgbs_206261    avgbs_2411    avgbs_4343    avgbs_18949    avgbs_19012    avgbs_21719    avgbs_23229    avgbs_24036    avgbs_35078    avgbs_50396    avgbs_52028    avgbs_59046    avgbs_59047    avgbs_65470    avgbs_65471    avgbs_68937    avgbs_68939    avgbs_70651    avgbs_73498    avgbs_75137    avgbs_75138    avgbs_76528    avgbs_86697    avgbs_98893    avgbs_107512    avgbs_115589    avgbs_115590    avgbs_117548    avgbs_119235    avgbs_123947    avgbs_124482    avgbs_125193    avgbs_232928    avgbs_233573    avgbs_202304    avgbs_202644    avgbs_203010    avgbs_237005    avgbs_224159    avgbs_203757    avgbs_238597    avgbs_205335    avgbs_242464    avgbs_243132    avgbs_243232    avgbs_204211    avgbs_6K_19478    avgbs_31538    avgbs_62581    avgbs_62582    avgbs_61368    avgbs_205536    avgbs_586    avgbs_33849    avgbs_58054    avgbs_96974    avgbs_111868    avgbs_111869    avgbs_33411    avgbs_44808    avgbs_13375    avgbs_235514    avgbs_13333    avgbs_237956    avgbs_206334    avgbs_54    avgbs_208281    avgbs_107033    avgbs_109189    avgbs_3136    avgbs_3137    avgbs_243964    avgbs_51392    avgbs_202032    avgbs_204651    avgbs_50377    avgbs_55005    avgbs_237138    avgbs_97033    avgbs_98664    avgbs_79223    avgbs_224148    avgbs_224674    avgbs_223432    avgbs_48784    avgbs_224163    avgbs_223281    avgbs_4697    avgbs_28165    avgbs_90421    avgbs_225115    avgbs_5048    avgbs_5049    avgbs_33313    avgbs_40081    avgbs_115010    avgbs_122490    avgbs_122491    avgbs_223875    avgbs_224333    avgbs_224509    avgbs_1422    avgbs_31855    avgbs_68936    avgbs_223201    avgbs_223586    avgbs_223908    avgbs_214731    avgbs_225637    avgbs_19192   
   5C 15 
 
 
  avgbs_77683    avgbs_223293    avgbs_224697    avgbs_57293    avgbs_108043    avgbs_35324    avgbs_75421    avgbs_109491    avgbs_116512    avgbs_6133   
   5C 16 
 
  GMI_ES_CC6322_820   
  avgbs_75917    avgbs_22292    avgbs_232769    avgbs_109854    avgbs_60454    avgbs_224851    avgbs_224892    avgbs_225027    avgbs_4206    avgbs_223261    avgbs_26434    avgbs_70604    avgbs_223730   
   5C 17 
  GMI_DS_oPt-1466_323    
  GMI_ES_CC13394_332   
  avgbs_17479    avgbs_46529    avgbs_46547    avgbs_91084    avgbs_118755    avgbs_118764    avgbs_230125    avgbs_231348    avgbs_7933    avgbs_20555    avgbs_34756    avgbs_67982    avgbs_72730    avgbs_90835    avgbs_90853    avgbs_90854    avgbs_91001    avgbs_91085    avgbs_112377    avgbs_230996    avgbs_225130    avgbs_8274    avgbs_223522    avgbs_207402    avgbs_27564    avgbs_29151    avgbs_59045    avgbs_78193    avgbs_37272    avgbs_99003    avgbs_205881    avgbs_37015    avgbs_37016    avgbs_61907    avgbs_72282    avgbs_110285    avgbs_116210    avgbs_203602    avgbs_224480    avgbs_118198    avgbs_234142    avgbs_37271    avgbs_223465    avgbs_225805    avgbs_73932    avgbs_223659   
   5C 18 
  GMI_ES15_c9085_462     GMI_ES15_c8238_156    
 
  avgbs_38254    avgbs_120234    avgbs_1445    avgbs_41255    avgbs_71208    avgbs_72729    avgbs_74364    avgbs_74365    avgbs_1444    avgbs_74459    avgbs_846    avgbs_84922    avgbs_232142    avgbs_31440    avgbs_120233    avgbs_237756    avgbs_235091    avgbs_87382    avgbs_87383    avgbs_119760    avgbs_25535    avgbs_25536    avgbs_75284    avgbs_75291    avgbs_81491    avgbs_83167    avgbs_91573    avgbs_103265    avgbs_112825    avgbs_224914    avgbs_13557    avgbs_204539    avgbs_15158    avgbs_27117    avgbs_27118    avgbs_31011    avgbs_42833    avgbs_95409    avgbs_113499    avgbs_114277    avgbs_121555    avgbs_123028    avgbs_123029    avgbs_201905    avgbs_203100    avgbs_81687    avgbs_100825    avgbs_110122    avgbs_110123    avgbs_239504    avgbs_20140    avgbs_29087    avgbs_29088    avgbs_51262    avgbs_58437    avgbs_64419    avgbs_66949    avgbs_77173    avgbs_77174    avgbs_87498    avgbs_89408    avgbs_89409    avgbs_201943    avgbs_237707    avgbs_204131    avgbs_238247    avgbs_204373    avgbs_205092    avgbs_80871    avgbs_121641    avgbs_62907    avgbs_73066    avgbs_87618    avgbs_90554    avgbs_204439    avgbs_204954    avgbs_234911    avgbs_205133    avgbs_206822    avgbs_205704    avgbs_111353    avgbs_111354    avgbs_22131    avgbs_115178    avgbs_215253    avgbs_29781    avgbs_29782    avgbs_223681    avgbs_117162    avgbs_6K_113240    avgbs_206757   
   5C 19 
 
 
  avgbs_60642    avgbs_5465    avgbs_230268    avgbs_230992    avgbs_90759    avgbs_224120   
   5C 20 
 
 
  avgbs_84145    avgbs_84146    avgbs_231385    avgbs_228709    avgbs_51329    avgbs_2177    avgbs_65264    avgbs_83091    avgbs_113064    avgbs_61418    avgbs_225145    avgbs_10337    avgbs_21518    avgbs_21520    avgbs_85686    avgbs_121144    avgbs_121145    avgbs_223407    avgbs_200601    avgbs_225680    avgbs_17322    avgbs_17323    avgbs_17324    avgbs_52713    avgbs_94603    avgbs_223582    avgbs_224261    avgbs_74370   
   5C 21 
 
 
  avgbs_117838    avgbs_117839    avgbs_10353    avgbs_87470    avgbs_225053    avgbs_225038    avgbs_10986   
   5C 22 
  GMI_ES02_c11855_528     GMI_ES01_c25986_126    
 
  avgbs_27596    avgbs_9557    avgbs_213207    avgbs_213256    avgbs_56289    avgbs_56290    avgbs_84956    avgbs_84957    avgbs_1443    avgbs_120699    avgbs_224453    avgbs_223337    avgbs_225788    avgbs_94201    avgbs_10976    avgbs_223418    avgbs_105944    avgbs_1174   
   5C 23 
 
 
  avgbs_3703    avgbs_228906   
   5C 24 
 
 
  avgbs_44119    avgbs_214065    avgbs_215090    avgbs_45490    avgbs_109824    avgbs_87852    avgbs_16046    avgbs_126061    avgbs_40336    avgbs_40337    avgbs_1382    avgbs_117542    avgbs_223198    avgbs_96617   
   5C 25 
  GMI_ES17_c18582_193    
 
  avgbs_89982    avgbs_122251    avgbs_243220    avgbs_44156    avgbs_40126    avgbs_88721    avgbs_88722    avgbs_217954    avgbs_91956    avgbs_76683    avgbs_110783    avgbs_4743    avgbs_225188   
   5C 26 
 
 
  avgbs_241691    avgbs_203303    avgbs_723    avgbs_223310    avgbs_223481    avgbs_13673    avgbs_224180    avgbs_224397    avgbs_225226    avgbs_97445    avgbs_101741    avgbs_225279    avgbs_31942   
   5C 27 
  GMI_ES02_lrc23878_108    
 
  avgbs_92639    avgbs_225166    avgbs_70025    avgbs_76785    avgbs_224649    avgbs_225483    avgbs_105041    avgbs_24590    avgbs_122883    avgbs_122884    avgbs_122885    avgbs_46506    avgbs_46507    avgbs_33121    avgbs_244238    avgbs_223605   
   5C 28 
 
 
  avgbs_101476    avgbs_213657    avgbs_215160    avgbs_10594    avgbs_100163    avgbs_38542    avgbs_96223    avgbs_13160    avgbs_60015    avgbs_24232    avgbs_223687    avgbs_225655    avgbs_225239    avgbs_224387    avgbs_224749   
   5C 29 
  GMI_ES17_c3625_404    
 
  avgbs_88844    avgbs_22566    avgbs_113070    avgbs_121137    avgbs_235024    avgbs_107866    avgbs_53264    avgbs_22951    avgbs_22952    avgbs_118767    avgbs_124770    avgbs_225696   
   5C 30 
  GMI_ES02_c13236_178    
 
  avgbs_225362    avgbs_230747    avgbs_11227    avgbs_17689    avgbs_213298    avgbs_225220    avgbs_210134    avgbs_118531    avgbs_81954    avgbs_81955    avgbs_115926    avgbs_234052    avgbs_201576    avgbs_233652    avgbs_111565    avgbs_65618    avgbs_5792    avgbs_5793    avgbs_42310    avgbs_24524    avgbs_24525    avgbs_115942    avgbs_115943    avgbs_16486    avgbs_19527    avgbs_19528    avgbs_28078    avgbs_122699    avgbs_124714    avgbs_126262    avgbs_119261    avgbs_123937    avgbs_123938    avgbs_123939    avgbs_216013    avgbs_7273    avgbs_7274    avgbs_34992    avgbs_63476    avgbs_224518    avgbs_46969    avgbs_118393    avgbs_118394    avgbs_243340    avgbs_243686    avgbs_224686    avgbs_2381    avgbs_2382    avgbs_2106   
   5C 31 
  GMI_ES01_c16767_69     GMI_ES02_c22115_682     GMI_ES02_c3374_73    
 
  avgbs_16222    avgbs_16227    avgbs_67920    avgbs_108142    avgbs_225490    avgbs_15363    avgbs_30246    avgbs_56313    avgbs_70568    avgbs_88767    avgbs_94295    avgbs_107580    avgbs_116260    avgbs_116261    avgbs_118132    avgbs_119345    avgbs_119346    avgbs_229181    avgbs_230921    avgbs_232631    avgbs_11543    avgbs_97154    avgbs_228830    avgbs_226526    avgbs_232278    avgbs_17492    avgbs_51041    avgbs_51042    avgbs_96224    avgbs_96225    avgbs_209197    avgbs_79031    avgbs_14123    avgbs_98190    avgbs_98191    avgbs_98192    avgbs_19509    avgbs_111773    avgbs_111774    avgbs_78844    avgbs_225139   
   5C 32 
 
 
  avgbs_60796   
   5C 33 
 
 
  avgbs_121549    avgbs_121550    avgbs_13677    avgbs_13678   
   5C 34 
 
 
  avgbs_77365    avgbs_28521    avgbs_28522    avgbs_87269    avgbs_87270    avgbs_6K_9614   
   5C 35 
  GMI_ES01_c12952_349    
 
  avgbs_64364    avgbs_46005    avgbs_70948    avgbs_6189    avgbs_7867    avgbs_7868    avgbs_17095    avgbs_21695    avgbs_21696    avgbs_88838    avgbs_88839    avgbs_110376    avgbs_110377    avgbs_110378    avgbs_34513    avgbs_19815    avgbs_19816    avgbs_235884    avgbs_122322    avgbs_244124    avgbs_6395    avgbs_114026    avgbs_207155    avgbs_208101   
   5C 36 
 
 
  avgbs_122516    avgbs_13048    avgbs_2853    avgbs_19426    avgbs_244352    avgbs_19695   
   5C 37 
  GMI_ES17_c5744_689    
 
  avgbs_57638    avgbs_9577    avgbs_201606   
   5C 38 
  GMI_ES02_c14691_637    
  GMI_ES15_c3003_520   
  avgbs_114156    avgbs_215989   
   5C 39 
  GMI_ES01_c12117_562     GMI_ES01_c1493_96    
 
  avgbs_2849    avgbs_22438    avgbs_41285    avgbs_41286    avgbs_91933    avgbs_231657    avgbs_201174    avgbs_122561    avgbs_208041    avgbs_10479    avgbs_94825    avgbs_223475    avgbs_6402    avgbs_6403    avgbs_107733    avgbs_74426    avgbs_15498    avgbs_9879    avgbs_75722    avgbs_224430    avgbs_117182    avgbs_224785    avgbs_215938    avgbs_76060    avgbs_100951    avgbs_206023    avgbs_114525    avgbs_214131    avgbs_26039    avgbs_22403    avgbs_224165    avgbs_225089    avgbs_223320    avgbs_13605    avgbs_21776    avgbs_36337    avgbs_37978    avgbs_223963    avgbs_225007    avgbs_84318    avgbs_21777    avgbs_13223    avgbs_11037    avgbs_77067    avgbs_215266   
   5C 40 
 
 
  avgbs_22120    avgbs_41232    avgbs_87737    avgbs_90413    avgbs_92873    avgbs_119385    avgbs_205502    avgbs_213998    avgbs_111365    avgbs_215800    avgbs_8383    avgbs_8389    avgbs_10097    avgbs_65497    avgbs_86976    avgbs_90358    avgbs_114925    avgbs_117022    avgbs_213269    avgbs_230737    avgbs_214631    avgbs_231071    avgbs_205500    avgbs_232269    avgbs_232517    avgbs_48108    avgbs_76098    avgbs_103039    avgbs_119880    avgbs_111499    avgbs_64139    avgbs_70674    avgbs_237047    avgbs_23335    avgbs_108007    avgbs_108008    avgbs_208843    avgbs_239242    avgbs_50112    avgbs_207603    avgbs_214092    avgbs_14283    avgbs_24808    avgbs_242291    avgbs_86476    avgbs_205309   
   5C 41 
  GMI_ES17_c2183_936    
 
  avgbs_15496    avgbs_201946    avgbs_38890    avgbs_116673    avgbs_18882    avgbs_23173    avgbs_24521    avgbs_24522    avgbs_83504    avgbs_215064    avgbs_65990    avgbs_91614    avgbs_122685    avgbs_62109    avgbs_78974    avgbs_108463    avgbs_53462    avgbs_23842    avgbs_13798    avgbs_41268    avgbs_8939    avgbs_94012    avgbs_110493    avgbs_10419    avgbs_21615    avgbs_92039    avgbs_214477    avgbs_205594    avgbs_206421    avgbs_12638    avgbs_229405    avgbs_105084    avgbs_96987    avgbs_96988    avgbs_7416    avgbs_110879    avgbs_110995    avgbs_206505   
   5C 42 
 
 
  avgbs_50111    avgbs_106835    avgbs_21146    avgbs_3439    avgbs_223250   
   5C 43 
 
 
  avgbs_230052    avgbs_24340    avgbs_6772    avgbs_201374    avgbs_124837    avgbs_124838   
   5C 45 
 
 
  avgbs_64138    avgbs_44699   
   5C 46 
 
 
  avgbs_52892    avgbs_80188    avgbs_80189   
   5C 47 
 
 
  avgbs_39069    avgbs_39070    avgbs_119066    avgbs_64752    avgbs_121159    avgbs_121211   
   5C 48 
 
 
  avgbs_43206    avgbs_56918    avgbs_238111    avgbs_3416   
   5C 49 
 
 
  avgbs_80548    avgbs_204962    avgbs_98305    avgbs_98306    avgbs_106836    avgbs_106838    avgbs_16049    avgbs_201999   
   5C 51 
 
 
  avgbs_89729    avgbs_89730    avgbs_55443    avgbs_30266    avgbs_1156    avgbs_1157    avgbs_1158    avgbs_205214    avgbs_202278    avgbs_117339    avgbs_117340    avgbs_105771    avgbs_105772   
   5C 52 
 
 
  avgbs_90527    avgbs_90531    avgbs_234834    avgbs_117260    avgbs_94935   
   5C 53 
 
 
  avgbs_121005    avgbs_116705    avgbs_125018    avgbs_125019    avgbs_114418   
   5C 54 
 
 
  avgbs_227166    avgbs_108539    avgbs_120369    avgbs_54756    avgbs_237448    avgbs_238442    avgbs_51712    avgbs_37880   
   5C 55 
 
 
  avgbs_94823    avgbs_242720    avgbs_36569    avgbs_203076    avgbs_50543    avgbs_203591    avgbs_87202    avgbs_87203   
   5C 56 
 
 
  avgbs_202009    avgbs_86643    avgbs_205329    avgbs_225602    avgbs_90768    avgbs_32516   
   5C 57 
  GMI_ES02_c3531_401    
 
  avgbs_107440    avgbs_204648   
   5C 58 
 
 
  avgbs_53334   
   5C 59 
 
 
  avgbs_22581    avgbs_4684    avgbs_38684    avgbs_51801    avgbs_78100    avgbs_110774    avgbs_214931    avgbs_215787    avgbs_35518    avgbs_35519    avgbs_46584    avgbs_6861    avgbs_14725    avgbs_14726    avgbs_24649    avgbs_77650    avgbs_77651    avgbs_50366   
   5C 60 
  GMI_DS_CC6822_86    
 
  avgbs_14929    avgbs_30224    avgbs_30225    avgbs_202844    avgbs_14653   
   5C 61 
 
 
  avgbs_206665    avgbs_207947   
   5C 62 
 
 
  avgbs_16137    avgbs_63273   
   5C 63 
 
 
  avgbs_22431    avgbs_202623    avgbs_221131    avgbs_28486    avgbs_47949    avgbs_114773    avgbs_220951    avgbs_57722    avgbs_42098    avgbs_214018    avgbs_17316    avgbs_220078    avgbs_113626    avgbs_50005    avgbs_229775    avgbs_12582    avgbs_121256   
   5C 64 
 
 
  avgbs_83387    avgbs_123370    avgbs_223037    avgbs_39176    avgbs_121598    avgbs_85842    avgbs_123371    avgbs_220900    avgbs_108799    avgbs_122639    avgbs_68442    avgbs_79170    avgbs_113230    avgbs_69968    avgbs_29433    avgbs_39939    avgbs_202964   
   5C 65 
 
 
  avgbs_30098    avgbs_30099    avgbs_86506    avgbs_217331    avgbs_220306    avgbs_116491    avgbs_18337   
   5C 66 
  GMI_ES15_c10103_419    
 
  avgbs_26208    avgbs_60039    avgbs_20512    avgbs_54336    avgbs_71584    avgbs_78161   
   5C 67 
 
 
  avgbs_50765    avgbs_216567    avgbs_218881    avgbs_50670    avgbs_72375    avgbs_205390    avgbs_106070    avgbs_15204    avgbs_105476    avgbs_46128   
   5C 68 
 
 
  avgbs_3674    avgbs_47323    avgbs_222592    avgbs_26125    avgbs_67354    avgbs_104641    avgbs_109725    avgbs_220298    avgbs_12786    avgbs_104686    avgbs_206249    avgbs_87304    avgbs_87305    avgbs_37246    avgbs_216258    avgbs_40852    avgbs_229961    avgbs_26944    avgbs_26945    avgbs_74824    avgbs_84783    avgbs_91577    avgbs_3297    avgbs_102415    avgbs_122370    avgbs_225768   
   5C 69 
 
 
  avgbs_98373    avgbs_109629    avgbs_109630    avgbs_231932    avgbs_203025    avgbs_207806    avgbs_22475    avgbs_126029    avgbs_206276    avgbs_15531    avgbs_20873    avgbs_121676    avgbs_206452    avgbs_18307    avgbs_41867    avgbs_207431    avgbs_85616    avgbs_85617    avgbs_48787    avgbs_48788   
   5C 70 
 
 
  avgbs_53366    avgbs_14636    avgbs_90039    avgbs_122012    avgbs_123496    avgbs_17108    avgbs_17109    avgbs_65872    avgbs_104912    avgbs_105079    avgbs_112252    avgbs_114700    avgbs_115004    avgbs_201840    avgbs_204670    avgbs_207807    avgbs_57756    avgbs_39931    avgbs_28439    avgbs_60896    avgbs_65501   
   5C 71 
  GMI_ES02_c21781_486     GMI_ES02_c31937_212    
 
  avgbs_22663    avgbs_82119    avgbs_1176    avgbs_10638    avgbs_41984    avgbs_41985    avgbs_48304    avgbs_99606    avgbs_218460    avgbs_205945    avgbs_54173    avgbs_54174    avgbs_83513    avgbs_39064    avgbs_222748    avgbs_29450    avgbs_90940    avgbs_115282    avgbs_115283    avgbs_202808    avgbs_120679    avgbs_124324    avgbs_113576    avgbs_125028    avgbs_15519    avgbs_15567    avgbs_16356    avgbs_39443    avgbs_65736    avgbs_116735    avgbs_121596    avgbs_123829    avgbs_204185    avgbs_36082    avgbs_36083    avgbs_208315    avgbs_16624    avgbs_208069    avgbs_25544    avgbs_206784    avgbs_46362    avgbs_46366    avgbs_203436    avgbs_6368    avgbs_48708    avgbs_40285    avgbs_46964   
   5C 72 
  GMI_ES01_c4371_166     GMI_ES01_c13663_212     GMI_ES01_c2879_300    
 
  avgbs_23082    avgbs_39095    avgbs_105418    avgbs_218298    avgbs_646    avgbs_12015    avgbs_20271    avgbs_29514    avgbs_30878    avgbs_34635    avgbs_34636    avgbs_48847    avgbs_55961    avgbs_56103    avgbs_70641    avgbs_76549    avgbs_79216    avgbs_82899    avgbs_87976    avgbs_88241    avgbs_91104    avgbs_101443    avgbs_117197    avgbs_122493    avgbs_216910    avgbs_201759    avgbs_203374    avgbs_203730    avgbs_219166    avgbs_204607    avgbs_220593    avgbs_220597    avgbs_205323    avgbs_205543    avgbs_205731    avgbs_222689    avgbs_208037    avgbs_208389    avgbs_27284    avgbs_46591    avgbs_58145    avgbs_65346    avgbs_203450    avgbs_214587    avgbs_222060    avgbs_40069    avgbs_40071    avgbs_46450    avgbs_56102    avgbs_74264    avgbs_111056    avgbs_117470    avgbs_217104    avgbs_45738    avgbs_15788    avgbs_15789    avgbs_15790    avgbs_115756    avgbs_203430    avgbs_219595    avgbs_9024    avgbs_12109    avgbs_65587    avgbs_65588    avgbs_18170    avgbs_27869    avgbs_27870    avgbs_59570    avgbs_120156    avgbs_206645    avgbs_65159    avgbs_124273    avgbs_230222    avgbs_8023    avgbs_11733    avgbs_12913    avgbs_12914    avgbs_12915    avgbs_22983    avgbs_25028    avgbs_31667    avgbs_32943    avgbs_40283    avgbs_40284    avgbs_43930    avgbs_46916    avgbs_46917    avgbs_60984    avgbs_62683    avgbs_66312    avgbs_71675    avgbs_75356    avgbs_80107    avgbs_83083    avgbs_87097    avgbs_90101    avgbs_92926    avgbs_101150    avgbs_117870    avgbs_202710    avgbs_229679    avgbs_202973    avgbs_203226    avgbs_214061    avgbs_230202    avgbs_224241    avgbs_214399    avgbs_203976    avgbs_224798    avgbs_206036    avgbs_18085    avgbs_30173    avgbs_30174    avgbs_58056    avgbs_70002    avgbs_2690    avgbs_11662    avgbs_11663    avgbs_18132    avgbs_23676    avgbs_23677    avgbs_54800    avgbs_93939    avgbs_99110    avgbs_99111    avgbs_99112    avgbs_105419    avgbs_50490    avgbs_54791    avgbs_56891    avgbs_115674    avgbs_4156    avgbs_16618    avgbs_27007    avgbs_27008    avgbs_108244    avgbs_113744    avgbs_118171    avgbs_202040    avgbs_16355    avgbs_16357    avgbs_39065    avgbs_101676    avgbs_122492    avgbs_20171    avgbs_38553    avgbs_104673    avgbs_68785    avgbs_88705    avgbs_49413    avgbs_55688    avgbs_15066    avgbs_36364    avgbs_36365    avgbs_110499    avgbs_124810    avgbs_206801    avgbs_19721    avgbs_206400    avgbs_16271    avgbs_42485    avgbs_114016    avgbs_111707    avgbs_208330    avgbs_47118    avgbs_107107    avgbs_107108    avgbs_107109    avgbs_120682    avgbs_84935    avgbs_84936    avgbs_35156    avgbs_55154    avgbs_76870    avgbs_91576    avgbs_101485    avgbs_112390    avgbs_124357    avgbs_124359    avgbs_125053    avgbs_125054    avgbs_204217    avgbs_208241    avgbs_1308    avgbs_25905    avgbs_25906    avgbs_25907    avgbs_30659    avgbs_40070    avgbs_40072    avgbs_105619    avgbs_121907    avgbs_207824    avgbs_207962    avgbs_85574    avgbs_85576    avgbs_122749    avgbs_125771    avgbs_203138    avgbs_208418    avgbs_4129    avgbs_4130    avgbs_16683    avgbs_78989    avgbs_106765    avgbs_202206    avgbs_202433    avgbs_202480    avgbs_204864    avgbs_206066    avgbs_4541    avgbs_4542    avgbs_16932    avgbs_19674    avgbs_85279    avgbs_85280    avgbs_106793    avgbs_120689    avgbs_125340    avgbs_207823    avgbs_207835    avgbs_17949    avgbs_104886    avgbs_104887    avgbs_107263    avgbs_109971    avgbs_109972    avgbs_89243    avgbs_99090    avgbs_99091    avgbs_121831    avgbs_206397    avgbs_5944    avgbs_105037    avgbs_105429    avgbs_105430    avgbs_30865    avgbs_76159    avgbs_79859    avgbs_86883    avgbs_123165    avgbs_123166    avgbs_202902    avgbs_206155    avgbs_72144    avgbs_87104    avgbs_112235    avgbs_205364    avgbs_38614    avgbs_76284    avgbs_80060    avgbs_104672    avgbs_115794    avgbs_206247    avgbs_1359    avgbs_38610    avgbs_38611    avgbs_55234    avgbs_80132    avgbs_105032    avgbs_114301    avgbs_124384    avgbs_203819    avgbs_206287    avgbs_26561    avgbs_47586    avgbs_55547    avgbs_55548    avgbs_99017    avgbs_206080    avgbs_17676    avgbs_17677    avgbs_80059    avgbs_92129    avgbs_105319    avgbs_112737    avgbs_113328    avgbs_206991    avgbs_208107    avgbs_2419    avgbs_37142    avgbs_41853    avgbs_41854    avgbs_112935    avgbs_112936    avgbs_112937    avgbs_204632    avgbs_206821    avgbs_206887    avgbs_17130    avgbs_80130    avgbs_100512    avgbs_100513    avgbs_106287    avgbs_107306    avgbs_107307    avgbs_71411    avgbs_82645    avgbs_107038    avgbs_207772    avgbs_207899    avgbs_59424    avgbs_87660    avgbs_206102    avgbs_102620    avgbs_207667    avgbs_203895    avgbs_75807    avgbs_126077    avgbs_204644   
   5C 73 
  GMI_ES01_lrc13031_95     GMI_ES15_c3717_245     GMI_ES17_c12869_484     GMI_ES02_c12844_488     GMI_DS_CC9634_105    
 
  avgbs_223022    avgbs_1345    avgbs_40086    avgbs_17426    avgbs_21345    avgbs_29371    avgbs_32456    avgbs_34361    avgbs_47877    avgbs_65079    avgbs_73788    avgbs_76164    avgbs_80254    avgbs_80260    avgbs_84800    avgbs_116447    avgbs_122791    avgbs_229713    avgbs_214816    avgbs_214986    avgbs_205774    avgbs_206029    avgbs_208090    avgbs_1342    avgbs_16283    avgbs_97743    avgbs_232781    avgbs_34462    avgbs_202980    avgbs_13848    avgbs_124210    avgbs_225822    avgbs_78104    avgbs_44418    avgbs_48025    avgbs_56027    avgbs_76133    avgbs_76163    avgbs_86755    avgbs_92442    avgbs_92444    avgbs_118854    avgbs_121251    avgbs_124479    avgbs_216078    avgbs_22924    avgbs_40356    avgbs_40358    avgbs_69927    avgbs_8229    avgbs_23518    avgbs_69462    avgbs_91692    avgbs_101906    avgbs_230869    avgbs_23800    avgbs_60694    avgbs_61393    avgbs_203991    avgbs_17798    avgbs_17799    avgbs_120680    avgbs_110180    avgbs_35580    avgbs_65666    avgbs_113952    avgbs_120999    avgbs_208360    avgbs_15604    avgbs_15605    avgbs_34254    avgbs_120390    avgbs_120391    avgbs_207785    avgbs_4539    avgbs_4540    avgbs_21658    avgbs_106776    avgbs_114345    avgbs_115275    avgbs_119997    avgbs_120017    avgbs_124790    avgbs_124985    avgbs_206407    avgbs_206809    avgbs_7213    avgbs_7214    avgbs_115317    avgbs_99156    avgbs_99157    avgbs_116314    avgbs_116315    avgbs_24716    avgbs_105066    avgbs_31760    avgbs_207779    avgbs_16235    avgbs_33936    avgbs_202956    avgbs_55447    avgbs_120131    avgbs_121876    avgbs_202699    avgbs_85258    avgbs_48711    avgbs_50736    avgbs_41858    avgbs_113726    avgbs_10536    avgbs_48223    avgbs_218758    avgbs_12044    avgbs_224708    avgbs_95581   
   5C 74 
  GMI_ES02_c11794_636     GMI_ES01_lrc8208_413    
 
  avgbs_27642    avgbs_112453    avgbs_114880    avgbs_114881    avgbs_217739    avgbs_218076    avgbs_219170    avgbs_10067    avgbs_91315    avgbs_23671    avgbs_92443    avgbs_92445    avgbs_42402    avgbs_42403    avgbs_83338    avgbs_121252    avgbs_10512    avgbs_61392    avgbs_87991    avgbs_111105    avgbs_216382    avgbs_15735    avgbs_111169    avgbs_38296    avgbs_46078    avgbs_52765    avgbs_59368    avgbs_112878    avgbs_121771    avgbs_218274    avgbs_19925    avgbs_79945    avgbs_110828    avgbs_111635    avgbs_109226    avgbs_109227    avgbs_58276    avgbs_58277    avgbs_80950    avgbs_224905   
   5C 75 
 
 
  avgbs_110183    avgbs_110184    avgbs_218323    avgbs_213725    avgbs_63018    avgbs_72941    avgbs_84528    avgbs_203662    avgbs_119672    avgbs_31112    avgbs_69646    avgbs_217921    avgbs_218296    avgbs_5459    avgbs_46737    avgbs_119195    avgbs_217664   
   5C 76 
  GMI_ES_CC14804_235     GMI_ES17_c7323_831     GMI_ES17_c4115_637     GMI_ES01_c10147_214     GMI_ES15_c3688_477     GMI_ES01_c4041_423     GMI_ES15_c10756_401    
 
  avgbs_62368    avgbs_9608    avgbs_24399    avgbs_35198    avgbs_35199    avgbs_41746    avgbs_41747    avgbs_43881    avgbs_47579    avgbs_61406    avgbs_68395    avgbs_99860    avgbs_100893    avgbs_119172    avgbs_125861    avgbs_126348    avgbs_218679    avgbs_216083    avgbs_223120    avgbs_223178    avgbs_21180    avgbs_31668    avgbs_53788    avgbs_58055    avgbs_60477    avgbs_65251    avgbs_71127    avgbs_71150    avgbs_76151    avgbs_121575    avgbs_18428    avgbs_18429    avgbs_38831    avgbs_38832    avgbs_42498    avgbs_47620    avgbs_53191    avgbs_64033    avgbs_64034    avgbs_116532    avgbs_2408    avgbs_78887    avgbs_115097    avgbs_217950    avgbs_214174    avgbs_21538    avgbs_21539    avgbs_21664    avgbs_21665    avgbs_67222    avgbs_68555    avgbs_204240    avgbs_24575    avgbs_38926    avgbs_61227    avgbs_219472    avgbs_14091    avgbs_17249    avgbs_25817    avgbs_26016    avgbs_27839    avgbs_41428    avgbs_48888    avgbs_48889    avgbs_49877    avgbs_69079    avgbs_69080    avgbs_69439    avgbs_97928    avgbs_97929    avgbs_102973    avgbs_109041    avgbs_121574    avgbs_217553    avgbs_220189    avgbs_26489    avgbs_58667    avgbs_63754    avgbs_521    avgbs_1332    avgbs_3519    avgbs_3600    avgbs_3706    avgbs_16078    avgbs_19915    avgbs_24117    avgbs_24400    avgbs_28422    avgbs_35241    avgbs_37525    avgbs_37526    avgbs_38389    avgbs_40850    avgbs_42135    avgbs_42256    avgbs_43880    avgbs_47082    avgbs_47946    avgbs_50625    avgbs_51015    avgbs_53789    avgbs_55301    avgbs_56832    avgbs_56909    avgbs_57457    avgbs_57458    avgbs_57615    avgbs_58224    avgbs_63123    avgbs_63998    avgbs_68207    avgbs_68208    avgbs_68948    avgbs_68949    avgbs_70906    avgbs_71583    avgbs_72125    avgbs_73358    avgbs_73359    avgbs_73438    avgbs_75929    avgbs_76089    avgbs_76090    avgbs_78639    avgbs_80906    avgbs_90065    avgbs_92104    avgbs_92565    avgbs_93790    avgbs_94177    avgbs_97754    avgbs_102627    avgbs_102807    avgbs_103120    avgbs_105729    avgbs_105730    avgbs_108128    avgbs_108181    avgbs_110611    avgbs_110612    avgbs_111060    avgbs_111064    avgbs_111065    avgbs_114730    avgbs_115737    avgbs_117077    avgbs_117078    avgbs_117472    avgbs_117997    avgbs_117998    avgbs_119173    avgbs_120266    avgbs_121109    avgbs_121475    avgbs_124061    avgbs_126090    avgbs_200979    avgbs_201141    avgbs_202671    avgbs_229568    avgbs_203307    avgbs_230238    avgbs_203594    avgbs_203900    avgbs_204150    avgbs_204395    avgbs_204528    avgbs_231206    avgbs_204866    avgbs_204976    avgbs_231624    avgbs_205596    avgbs_231881    avgbs_206226    avgbs_206281    avgbs_206593    avgbs_232351    avgbs_232524    avgbs_207777    avgbs_232656    avgbs_60478    avgbs_4788    avgbs_5618    avgbs_21493    avgbs_30590    avgbs_37726    avgbs_40087    avgbs_40088    avgbs_40089    avgbs_47103    avgbs_47104    avgbs_54013    avgbs_54014    avgbs_54179    avgbs_60693    avgbs_62369    avgbs_64371    avgbs_68742    avgbs_69778    avgbs_70181    avgbs_70182    avgbs_71606    avgbs_90099    avgbs_121868    avgbs_203773    avgbs_203869    avgbs_204128    avgbs_13945    avgbs_59330    avgbs_62410    avgbs_62411    avgbs_229520    avgbs_43144    avgbs_44261    avgbs_55803    avgbs_58993    avgbs_58994    avgbs_61407    avgbs_73525    avgbs_78028    avgbs_120273    avgbs_120274    avgbs_203850    avgbs_230608    avgbs_38383    avgbs_38384    avgbs_8525    avgbs_8526    avgbs_26015    avgbs_40482    avgbs_40483    avgbs_40484    avgbs_43877    avgbs_50411    avgbs_60920    avgbs_71695    avgbs_80759    avgbs_90325    avgbs_97202    avgbs_123872    avgbs_203558    avgbs_50620    avgbs_50621    avgbs_7598    avgbs_7599    avgbs_7600    avgbs_16650    avgbs_24228    avgbs_40493    avgbs_69077    avgbs_70279    avgbs_72643    avgbs_72644    avgbs_82901    avgbs_89035    avgbs_108245    avgbs_119193    avgbs_121062    avgbs_121063    avgbs_125687    avgbs_202045    avgbs_229525    avgbs_230192    avgbs_230858    avgbs_231340    avgbs_15743    avgbs_17754    avgbs_49097    avgbs_66255    avgbs_99639    avgbs_99640    avgbs_123974    avgbs_1726    avgbs_1727    avgbs_19245    avgbs_23857    avgbs_25815    avgbs_25816    avgbs_41623    avgbs_41698    avgbs_41699    avgbs_48709    avgbs_55982    avgbs_81976    avgbs_88113    avgbs_88114    avgbs_101522    avgbs_115329    avgbs_116550    avgbs_125965    avgbs_22074    avgbs_30237    avgbs_31745    avgbs_33510    avgbs_35698    avgbs_58527    avgbs_58528    avgbs_77758    avgbs_92134    avgbs_204202    avgbs_231174    avgbs_8756    avgbs_8757    avgbs_14597    avgbs_19893    avgbs_23001    avgbs_26199    avgbs_27396    avgbs_29676    avgbs_29677    avgbs_32244    avgbs_37170    avgbs_37171    avgbs_41642    avgbs_42492    avgbs_44485    avgbs_46120    avgbs_47220    avgbs_49631    avgbs_53081    avgbs_66838    avgbs_69355    avgbs_69761    avgbs_73418    avgbs_73419    avgbs_73448    avgbs_73449    avgbs_87707    avgbs_97626    avgbs_97627    avgbs_98856    avgbs_106847    avgbs_107387    avgbs_203099    avgbs_203426    avgbs_203716    avgbs_204228    avgbs_29325    avgbs_29326    avgbs_35689    avgbs_48454    avgbs_59141    avgbs_59194    avgbs_92133    avgbs_229179    avgbs_23696    avgbs_56669    avgbs_78239    avgbs_83936    avgbs_97930    avgbs_97931    avgbs_101920    avgbs_118876    avgbs_118877    avgbs_126076    avgbs_203468    avgbs_22522    avgbs_27509    avgbs_39833    avgbs_42662    avgbs_42663    avgbs_44697    avgbs_44698    avgbs_47496    avgbs_106502    avgbs_106503    avgbs_116952    avgbs_116953    avgbs_116954    avgbs_116955    avgbs_230064    avgbs_204656    avgbs_100160    avgbs_221846    avgbs_39338    avgbs_39339    avgbs_39340    avgbs_6723    avgbs_203688    avgbs_21208    avgbs_201694    avgbs_108915    avgbs_14691    avgbs_17051    avgbs_17052    avgbs_17824    avgbs_17998    avgbs_18263    avgbs_20950    avgbs_22479    avgbs_30645    avgbs_105018    avgbs_110959    avgbs_112265    avgbs_112266    avgbs_113273    avgbs_115003    avgbs_121898    avgbs_121899    avgbs_202426    avgbs_203246    avgbs_206946    avgbs_7215    avgbs_64035    avgbs_75874    avgbs_110720    avgbs_121662    avgbs_121663    avgbs_122089    avgbs_202905    avgbs_204106    avgbs_10326    avgbs_10327    avgbs_18190    avgbs_20236    avgbs_20247    avgbs_20951    avgbs_50194    avgbs_52315    avgbs_65201    avgbs_104914    avgbs_107554    avgbs_110185    avgbs_110226    avgbs_111021    avgbs_111149    avgbs_114994    avgbs_115330    avgbs_121480    avgbs_123080    avgbs_123081    avgbs_124396    avgbs_202403    avgbs_202417    avgbs_202420    avgbs_203677    avgbs_979    avgbs_7183    avgbs_15302    avgbs_16287    avgbs_16288    avgbs_49202    avgbs_65781    avgbs_93402    avgbs_207956    avgbs_208095    avgbs_122559    avgbs_125904    avgbs_207086    avgbs_104814    avgbs_203752    avgbs_112150    avgbs_20641    avgbs_25059    avgbs_57745    avgbs_106630    avgbs_206424    avgbs_113489    avgbs_50642    avgbs_65292    avgbs_22393    avgbs_39263    avgbs_109247    avgbs_206777    avgbs_207834    avgbs_204850    avgbs_38927    avgbs_122088   
   5C 77 
  GMI_ES01_c791_1072    
 
  avgbs_80356    avgbs_117904    avgbs_213888    avgbs_214381    avgbs_15328    avgbs_18075    avgbs_46323    avgbs_46496    avgbs_62799    avgbs_65250    avgbs_70266    avgbs_79221    avgbs_79906    avgbs_83671    avgbs_88688    avgbs_88843    avgbs_96064    avgbs_96741    avgbs_109881    avgbs_112401    avgbs_112919    avgbs_116014    avgbs_120259    avgbs_120279    avgbs_217026    avgbs_217144    avgbs_217579    avgbs_218501    avgbs_214086    avgbs_218719    avgbs_203629    avgbs_219192    avgbs_204337    avgbs_204916    avgbs_215073    avgbs_221074    avgbs_205809    avgbs_222338    avgbs_16972    avgbs_20048    avgbs_46435    avgbs_66194    avgbs_81823    avgbs_81824    avgbs_90327    avgbs_126345    avgbs_217395    avgbs_20927    avgbs_41215    avgbs_43388    avgbs_61593    avgbs_73487    avgbs_115310    avgbs_218541    avgbs_219275    avgbs_219493    avgbs_43796    avgbs_54953    avgbs_66623    avgbs_70571    avgbs_70572    avgbs_70616    avgbs_213659    avgbs_220024    avgbs_16667    avgbs_30722    avgbs_30723    avgbs_37274    avgbs_37571    avgbs_40531    avgbs_71377    avgbs_73485    avgbs_17755    avgbs_217274    avgbs_39257    avgbs_84247    avgbs_114400    avgbs_1316    avgbs_18391    avgbs_18392    avgbs_26153    avgbs_68900    avgbs_90843    avgbs_102556    avgbs_218835    avgbs_215261    avgbs_2240    avgbs_20309    avgbs_125686    avgbs_46328    avgbs_46329    avgbs_110784    avgbs_65294    avgbs_201024    avgbs_207895    avgbs_21433    avgbs_53856    avgbs_85381    avgbs_202878    avgbs_50184    avgbs_50185    avgbs_108344    avgbs_112219    avgbs_202161    avgbs_203649    avgbs_22123    avgbs_202162    avgbs_15453    avgbs_15454    avgbs_61340    avgbs_92662    avgbs_6953    avgbs_123955    avgbs_49492    avgbs_207149    avgbs_125745    avgbs_125746    avgbs_120610    avgbs_202054   
   5C 78 
  GMI_ES01_c15886_573    
  GMI_ES02_c15652_109   
  avgbs_69787    avgbs_70329    avgbs_91255    avgbs_104991    avgbs_125823    avgbs_216732    avgbs_214708    avgbs_204381    avgbs_206283    avgbs_87961    avgbs_214454    avgbs_9374    avgbs_221018    avgbs_70369    avgbs_220001    avgbs_33638    avgbs_81445    avgbs_114862    avgbs_18301    avgbs_18302    avgbs_31274    avgbs_31275    avgbs_48388    avgbs_48825    avgbs_61071    avgbs_69164    avgbs_98106    avgbs_121814    avgbs_218785    avgbs_112625    avgbs_19151    avgbs_39646    avgbs_110500    avgbs_203407   
   5C 79 
 
 
  avgbs_216759    avgbs_219974    avgbs_21456    avgbs_112938    avgbs_203480    avgbs_20274    avgbs_20275    avgbs_18959    avgbs_111090    avgbs_119213    avgbs_78611    avgbs_93439    avgbs_93440    avgbs_202453    avgbs_101991    avgbs_7221    avgbs_112232    avgbs_44145    avgbs_65500   
   5C 80 
 
 
  avgbs_40083    avgbs_104435    avgbs_110829    avgbs_115821    avgbs_8562    avgbs_113745    avgbs_116430    avgbs_122505    avgbs_29894    avgbs_4462   
   5C 81 
 
 
  avgbs_106605    avgbs_48700    avgbs_98929    avgbs_5021    avgbs_102155    avgbs_201292    avgbs_20496    avgbs_206765    avgbs_4264    avgbs_115311    avgbs_200732    avgbs_225626   
   5C 82 
 
 
  avgbs_19407    avgbs_19408    avgbs_97511    avgbs_106446    avgbs_106447    avgbs_121806    avgbs_121807    avgbs_221764    avgbs_60507    avgbs_19920    avgbs_20448    avgbs_110220    avgbs_202443    avgbs_76943    avgbs_76944    avgbs_224787   
   5C 83 
  GMI_ES17_c12067_507    
 
  avgbs_65342   
   5C 84 
 
 
  avgbs_104642   
   5C 85 
 
 
  avgbs_122379    avgbs_101488   
   5C 86 
  GMI_ES17_c2656_146    
 
  avgbs_38216   
   5C 87 
 
 
  avgbs_230081    avgbs_202595    avgbs_125339   
   5C 89 
 
 
  avgbs_217688    avgbs_28   
   5C 90 
 
 
  avgbs_72051    avgbs_220101    avgbs_62788   
   5C 91 
 
 
  avgbs_9015    avgbs_11920    avgbs_101603    avgbs_214020   
   5C 92 
  GMI_ES02_c2554_426    
 
 
   5C 94 
 
 
  avgbs_17901   
   5C 95 
 
 
  avgbs_109185    avgbs_56242    avgbs_56243   
   5C 96 
 
 
  avgbs_105270    avgbs_105271    avgbs_98658    avgbs_98657   
   5C 100 
 
 
  avgbs_44247   
   5C 108 
  GMI_ES17_c3370_293    
 
  avgbs_35344    avgbs_209798    avgbs_12720    avgbs_44018    avgbs_53568    avgbs_109348    avgbs_109349    avgbs_230050    avgbs_230335   
   5C 109 
 
 
  avgbs_13180   
   5C 110 
 
 
  avgbs_85062   
   5C 111 
 
 
  avgbs_123516    avgbs_123517    avgbs_123518    avgbs_212176   
   5C 113 
 
 
  avgbs_229389   
   5C 116 
 
 
  avgbs_106759    avgbs_232638   
   5C 119 
 
 
  avgbs_96648    avgbs_122317    avgbs_122318    avgbs_202889    avgbs_232519   
   5C 121 
 
 
  avgbs_36072    avgbs_36073   
   5C 122 
 
 
  avgbs_65481    avgbs_87042   
   5C 123 
 
 
  avgbs_23733   
   5C 126 
  GMI_ES01_c2481_1101    
 
  avgbs_87885    avgbs_79805    avgbs_87943   
 
 
   Chromosome 6C    Chr   Pos   Framework   Placed SNP  Placed GBS  
   6C -4 
 
 
  avgbs_104702   
   6C -3 
 
 
  avgbs_10566    avgbs_230460   
   6C -2 
 
 
  avgbs_83255   
   6C -1 
 
 
  avgbs_32805    avgbs_32807    avgbs_53716    avgbs_71467    avgbs_219026    avgbs_222624    avgbs_40478    avgbs_40479    avgbs_217220    avgbs_53680    avgbs_27127    avgbs_95768    avgbs_229564    avgbs_231805   
   6C 0 
  GMI_ES01_c3132_376    
 
  avgbs_53714    avgbs_20286   
   6C 1 
 
 
  avgbs_1580   
   6C 2 
 
 
  avgbs_119980    avgbs_217110    avgbs_1581    avgbs_104320    avgbs_116373    avgbs_24404    avgbs_113467    avgbs_120454   
   6C 3 
  GMI_ES17_c11186_555    
 
  avgbs_219889    avgbs_4337    avgbs_48886    avgbs_55481    avgbs_70450    avgbs_106544    avgbs_217254    avgbs_16148    avgbs_19914    avgbs_98822    avgbs_102816    avgbs_122966    avgbs_217221    avgbs_223038    avgbs_7172    avgbs_17671    avgbs_17672    avgbs_26774    avgbs_26775    avgbs_26776    avgbs_33188    avgbs_37501    avgbs_69920    avgbs_69921    avgbs_6K_100295    avgbs_217388    avgbs_218030    avgbs_218164    avgbs_220580    avgbs_222275    avgbs_222938    avgbs_30438    avgbs_72296    avgbs_123231    avgbs_217897    avgbs_20605    avgbs_26927    avgbs_30436    avgbs_94496    avgbs_107352    avgbs_123230    avgbs_123232    avgbs_86505    avgbs_111352    avgbs_119982    avgbs_230271    avgbs_10179    avgbs_32739    avgbs_42614    avgbs_73688    avgbs_73689    avgbs_30435    avgbs_243215    avgbs_91124    avgbs_91125    avgbs_55437    avgbs_21090    avgbs_19362    avgbs_28699    avgbs_8810    avgbs_47657    avgbs_111631    avgbs_117140    avgbs_201550    avgbs_243417    avgbs_71792    avgbs_42320    avgbs_63294    avgbs_92893    avgbs_240527    avgbs_17874    avgbs_39359    avgbs_39360    avgbs_242666    avgbs_86290    avgbs_14735    avgbs_84872    avgbs_93908    avgbs_79345    avgbs_3760    avgbs_3762    avgbs_7753    avgbs_7754    avgbs_7755    avgbs_90671    avgbs_90672    avgbs_244310    avgbs_65344    avgbs_123990    avgbs_78125    avgbs_237042    avgbs_234744    avgbs_29273    avgbs_55438    avgbs_235819    avgbs_67455    avgbs_243424    avgbs_22652    avgbs_35791    avgbs_48922    avgbs_237046    avgbs_30437    avgbs_85149    avgbs_86782    avgbs_242980    avgbs_243532    avgbs_46978    avgbs_234942    avgbs_241613    avgbs_33187    avgbs_99998    avgbs_117347    avgbs_18508   
   6C 5 
 
 
  avgbs_217275    avgbs_57932   
   6C 6 
 
 
  avgbs_59187    avgbs_79628   
   6C 7 
 
 
  avgbs_120564    avgbs_120565    avgbs_120402    avgbs_207786    avgbs_3761    avgbs_60195   
   6C 8 
 
 
  avgbs_205988    avgbs_221391   
   6C 9 
 
 
  avgbs_81624    avgbs_81625    avgbs_81626    avgbs_12458    avgbs_74930   
   6C 10 
 
 
  avgbs_25394    avgbs_122396    avgbs_97400    avgbs_24849    avgbs_218368    avgbs_3631    avgbs_71878   
   6C 11 
 
 
  avgbs_14180    avgbs_14181    avgbs_54599    avgbs_200362    avgbs_66486    avgbs_230779    avgbs_122873    avgbs_6K_77957    avgbs_225047   
   6C 12 
 
 
  avgbs_36661    avgbs_12095    avgbs_46875    avgbs_224131   
   6C 13 
 
 
  avgbs_219233    avgbs_20868   
   6C 14 
 
 
  avgbs_229070    avgbs_88501    avgbs_8130   
   6C 15 
  GMI_ES02_c15952_348    
 
  avgbs_22704   
   6C 16 
 
 
  avgbs_11435    avgbs_213303    avgbs_26782    avgbs_26783    avgbs_121496    avgbs_215309    avgbs_11422    avgbs_64396    avgbs_82107    avgbs_44395   
   6C 17 
 
 
  avgbs_823   
   6C 18 
  GMI_ES02_c28827_474    
 
  avgbs_221984    avgbs_65425    avgbs_219716   
   6C 19 
 
 
  avgbs_222085   
   6C 20 
 
 
  avgbs_11190    avgbs_201722   
   6C 22 
  GMI_DS_oPt-14552_101    
 
 
   6C 23 
  GMI_ES15_c10866_209    
 
  avgbs_4754    avgbs_5581    avgbs_5582    avgbs_9710    avgbs_50081    avgbs_74297    avgbs_32169    avgbs_118572    avgbs_206724    avgbs_44917    avgbs_74069    avgbs_218553    avgbs_117146    avgbs_117147    avgbs_230925    avgbs_231311    avgbs_9787    avgbs_15077    avgbs_27266    avgbs_29526    avgbs_31723    avgbs_42951    avgbs_92283    avgbs_103860    avgbs_116783    avgbs_117375    avgbs_123839    avgbs_124090    avgbs_229009    avgbs_223509    avgbs_229551    avgbs_19110    avgbs_27278    avgbs_40489    avgbs_51060    avgbs_81399    avgbs_83049    avgbs_83050    avgbs_104752    avgbs_231640    avgbs_940    avgbs_67975    avgbs_89941    avgbs_119964    avgbs_119965    avgbs_29814    avgbs_47731    avgbs_47732    avgbs_104118    avgbs_115247    avgbs_21860    avgbs_6545    avgbs_8620    avgbs_11066    avgbs_11067    avgbs_11068    avgbs_16375    avgbs_16376    avgbs_16391    avgbs_16392    avgbs_23331    avgbs_23477    avgbs_39210    avgbs_40280    avgbs_40281    avgbs_41522   
   6C 24 
 
 
  avgbs_44769    avgbs_14850    avgbs_55674    avgbs_6K_100229    avgbs_219757    avgbs_72772   
   6C 25 
  GMI_ES02_c23210_257    
 
  avgbs_6K_100394    avgbs_68382   
   6C 27 
  GMI_ES17_c5666_258     GMI_ES02_c34690_199     GMI_DS_CC9093_95     GMI_DS_CC9448_443     GMI_DS_CC7847_62     GMI_DS_CC1776_314     GMI_ES17_c2308_1026     GMI_ES01_c8337_431     GMI_ES01_c3899_470    
 
  avgbs_4799    avgbs_6K_6566    avgbs_9159    avgbs_11552    avgbs_11864    avgbs_16054    avgbs_16055    avgbs_16714    avgbs_23316    avgbs_24298    avgbs_27349    avgbs_32222    avgbs_34916    avgbs_35002    avgbs_49362    avgbs_49383    avgbs_51769    avgbs_55195    avgbs_55787    avgbs_60965    avgbs_61410    avgbs_64551    avgbs_64552    avgbs_65907    avgbs_66744    avgbs_70099    avgbs_71355    avgbs_78514    avgbs_78516    avgbs_80650    avgbs_86611    avgbs_90433    avgbs_94419    avgbs_98073    avgbs_98216    avgbs_110154    avgbs_110529    avgbs_112600    avgbs_113063    avgbs_115533    avgbs_121340    avgbs_125852    avgbs_216740    avgbs_216754    avgbs_216901    avgbs_217947    avgbs_218128    avgbs_218811    avgbs_218812    avgbs_218933    avgbs_219079    avgbs_219147    avgbs_219783    avgbs_219979    avgbs_220600    avgbs_221169    avgbs_221227    avgbs_221714    avgbs_222324    avgbs_222491    avgbs_222755    avgbs_223150    avgbs_223152    avgbs_56025    avgbs_56026    avgbs_4299    avgbs_4653    avgbs_4654    avgbs_6138    avgbs_9139    avgbs_11009    avgbs_11341    avgbs_12581    avgbs_15886    avgbs_6K_14868    avgbs_21861    avgbs_21862    avgbs_23075    avgbs_24673    avgbs_25595    avgbs_25734    avgbs_26449    avgbs_28220    avgbs_29795    avgbs_35738    avgbs_35992    avgbs_36188    avgbs_36617    avgbs_36709    avgbs_36738    avgbs_46657    avgbs_47222    avgbs_47519    avgbs_47553    avgbs_47698    avgbs_48535    avgbs_54476    avgbs_56260    avgbs_56265    avgbs_58742    avgbs_59428    avgbs_61060    avgbs_61390    avgbs_65671    avgbs_66868    avgbs_67190    avgbs_67410    avgbs_67417    avgbs_71186    avgbs_75978    avgbs_76238    avgbs_77031    avgbs_77061    avgbs_79485    avgbs_81912    avgbs_86126    avgbs_86127    avgbs_88016    avgbs_89830    avgbs_91175    avgbs_96854    avgbs_97573    avgbs_97998    avgbs_98461    avgbs_100279    avgbs_100620    avgbs_101789    avgbs_103663    avgbs_106801    avgbs_107852    avgbs_116040    avgbs_121883    avgbs_123817    avgbs_124656    avgbs_125121    avgbs_125707    avgbs_216378    avgbs_244746    avgbs_213244    avgbs_216847    avgbs_217494    avgbs_217594    avgbs_217683    avgbs_217698    avgbs_217981    avgbs_218032    avgbs_218115    avgbs_218132    avgbs_218420    avgbs_218698    avgbs_218716    avgbs_218740    avgbs_218957    avgbs_219175    avgbs_219773    avgbs_219808    avgbs_219819    avgbs_220053    avgbs_220349    avgbs_220506    avgbs_221142    avgbs_221217    avgbs_221224    avgbs_221547    avgbs_221853    avgbs_222352    avgbs_222632    avgbs_222946    avgbs_223063    avgbs_223149    avgbs_7886    avgbs_48553    avgbs_118180    avgbs_122293    avgbs_125141    avgbs_2803    avgbs_2804    avgbs_5517    avgbs_24312    avgbs_24313    avgbs_24501    avgbs_24502    avgbs_28348    avgbs_36737    avgbs_39006    avgbs_40630    avgbs_40631    avgbs_44629    avgbs_48204    avgbs_62480    avgbs_70461    avgbs_76434    avgbs_79840    avgbs_80450    avgbs_90095    avgbs_94927    avgbs_111390    avgbs_123604    avgbs_124663    avgbs_218537    avgbs_219177    avgbs_220399    avgbs_4101    avgbs_15868    avgbs_16370    avgbs_16371    avgbs_23577    avgbs_23578    avgbs_26834    avgbs_30564    avgbs_36637    avgbs_40637    avgbs_41558    avgbs_47355    avgbs_52753    avgbs_54208    avgbs_59608    avgbs_60158    avgbs_61518    avgbs_63184    avgbs_67062    avgbs_67147    avgbs_69629    avgbs_74682    avgbs_77062    avgbs_80835    avgbs_106209    avgbs_113202    avgbs_113800    avgbs_124689    avgbs_124896    avgbs_125172    avgbs_28614    avgbs_218194    avgbs_219766    avgbs_220256    avgbs_221480    avgbs_28745    avgbs_61461    avgbs_92102    avgbs_92650    avgbs_113268    avgbs_219489    avgbs_221290    avgbs_6K_720    avgbs_18653    avgbs_18654    avgbs_24178    avgbs_24495    avgbs_26284    avgbs_30297    avgbs_35678    avgbs_36183    avgbs_36495    avgbs_40991    avgbs_43292    avgbs_43293    avgbs_43294    avgbs_50995    avgbs_54441    avgbs_58062    avgbs_59334    avgbs_59494    avgbs_68094    avgbs_75689    avgbs_81156    avgbs_105127    avgbs_106429    avgbs_117773    avgbs_117930    avgbs_117931    avgbs_217575    avgbs_218180    avgbs_218380    avgbs_220644    avgbs_96975    avgbs_12606    avgbs_24507    avgbs_31220    avgbs_34877    avgbs_35557    avgbs_36680    avgbs_51760    avgbs_57536    avgbs_67591    avgbs_69558    avgbs_83393    avgbs_95990    avgbs_97559    avgbs_98217    avgbs_98462    avgbs_101262    avgbs_110019    avgbs_110155    avgbs_110617    avgbs_118014    avgbs_119665    avgbs_229304    avgbs_229794    avgbs_229820    avgbs_230342    avgbs_231232    avgbs_231284    avgbs_231517    avgbs_231537    avgbs_231813    avgbs_231936    avgbs_232459    avgbs_232554    avgbs_232711    avgbs_11427    avgbs_11428    avgbs_28225    avgbs_36205    avgbs_60114    avgbs_62117    avgbs_65207    avgbs_65208    avgbs_66513    avgbs_88413    avgbs_88414    avgbs_91126    avgbs_91127    avgbs_95860    avgbs_108029    avgbs_117277    avgbs_229814    avgbs_230632    avgbs_232669    avgbs_23586    avgbs_23636    avgbs_36484    avgbs_36487    avgbs_39613    avgbs_52432    avgbs_75243    avgbs_229444    avgbs_230771    avgbs_3990    avgbs_5277    avgbs_53119    avgbs_95561    avgbs_103330    avgbs_105678    avgbs_111883    avgbs_111884    avgbs_124870    avgbs_223113    avgbs_133    avgbs_24019    avgbs_29815    avgbs_29816    avgbs_37410    avgbs_50388    avgbs_54902    avgbs_106208    avgbs_118304    avgbs_218984    avgbs_232675    avgbs_69336    avgbs_16447    avgbs_39332    avgbs_102097    avgbs_102098    avgbs_117832    avgbs_126277    avgbs_126278    avgbs_232541    avgbs_4149    avgbs_4150    avgbs_75506    avgbs_3176    avgbs_7136    avgbs_16534    avgbs_16880    avgbs_20123    avgbs_24145    avgbs_30516    avgbs_30563    avgbs_30596    avgbs_31480    avgbs_35090    avgbs_38338    avgbs_43588    avgbs_47488    avgbs_47489    avgbs_49282    avgbs_50368    avgbs_52295    avgbs_52547    avgbs_52678    avgbs_55953    avgbs_60431    avgbs_62006    avgbs_62622    avgbs_62942    avgbs_64590    avgbs_66630    avgbs_68706    avgbs_68707    avgbs_70138    avgbs_70783    avgbs_76209    avgbs_76210    avgbs_81518    avgbs_83698    avgbs_83699    avgbs_86196    avgbs_95850    avgbs_103595    avgbs_108797    avgbs_113695    avgbs_115027    avgbs_231544    avgbs_10669    avgbs_10670    avgbs_30180    avgbs_30181    avgbs_35025    avgbs_36483    avgbs_44708    avgbs_61027    avgbs_61028    avgbs_72640    avgbs_72737    avgbs_102779    avgbs_121148    avgbs_30205    avgbs_94053    avgbs_234527    avgbs_43519    avgbs_106800    avgbs_76091    avgbs_4234    avgbs_40571    avgbs_40572    avgbs_26609    avgbs_29944    avgbs_57532    avgbs_269    avgbs_84160    avgbs_84161    avgbs_94705    avgbs_99977    avgbs_70503    avgbs_103741    avgbs_30287    avgbs_49727    avgbs_241696    avgbs_75182    avgbs_34000    avgbs_64241    avgbs_89543    avgbs_92752    avgbs_103874    avgbs_74033    avgbs_82243    avgbs_14459    avgbs_16804    avgbs_34534    avgbs_40696    avgbs_55737    avgbs_66609    avgbs_75536    avgbs_92921    avgbs_105939    avgbs_120826    avgbs_232893    avgbs_238465    avgbs_240530    avgbs_241451    avgbs_30232    avgbs_78959    avgbs_235873    avgbs_10148    avgbs_12115    avgbs_24151    avgbs_27631    avgbs_28866    avgbs_50076    avgbs_52754    avgbs_66169    avgbs_66384    avgbs_66471    avgbs_70735    avgbs_77685    avgbs_79604    avgbs_80753    avgbs_88564    avgbs_95709    avgbs_101292    avgbs_105876    avgbs_115270    avgbs_122145    avgbs_122500    avgbs_122501    avgbs_122599    avgbs_122602    avgbs_235290    avgbs_235525    avgbs_235759    avgbs_236964    avgbs_238161    avgbs_238434    avgbs_238528    avgbs_243183    avgbs_33343    avgbs_47696    avgbs_238846    avgbs_244378    avgbs_4133    avgbs_4789    avgbs_16989    avgbs_17872    avgbs_25559    avgbs_30095    avgbs_30597    avgbs_30599    avgbs_30600    avgbs_31550    avgbs_34528    avgbs_36689    avgbs_39058    avgbs_39760    avgbs_41459    avgbs_41462    avgbs_46661    avgbs_47368    avgbs_47410    avgbs_49283    avgbs_49284    avgbs_50978    avgbs_52184    avgbs_53202    avgbs_58723    avgbs_65750    avgbs_67948    avgbs_71442    avgbs_6K_66708    avgbs_72338    avgbs_73812    avgbs_74482    avgbs_77265    avgbs_77266    avgbs_82274    avgbs_86195    avgbs_86284    avgbs_89672    avgbs_89933    avgbs_92290    avgbs_99214    avgbs_99215    avgbs_101709    avgbs_104218    avgbs_105357    avgbs_106022    avgbs_106023    avgbs_111618    avgbs_112205    avgbs_121534    avgbs_124099    avgbs_125922    avgbs_125923    avgbs_217886    avgbs_218659    avgbs_237289    avgbs_219837    avgbs_238794    avgbs_240244    avgbs_241078    avgbs_241196    avgbs_241966    avgbs_31212    avgbs_33097    avgbs_33098    avgbs_39203    avgbs_40075    avgbs_47695    avgbs_49642    avgbs_49643    avgbs_65535    avgbs_74505    avgbs_93482    avgbs_93483    avgbs_102860    avgbs_102861    avgbs_102862    avgbs_121235    avgbs_217854    avgbs_1939    avgbs_6K_3516    avgbs_8345    avgbs_10681    avgbs_15566    avgbs_17187    avgbs_17388    avgbs_20124    avgbs_21874    avgbs_29589    avgbs_30598    avgbs_34828    avgbs_40574    avgbs_40621    avgbs_46780    avgbs_54057    avgbs_54903    avgbs_56303    avgbs_71895    avgbs_76196    avgbs_77272    avgbs_79007    avgbs_80910    avgbs_88309    avgbs_89926    avgbs_90156    avgbs_100564    avgbs_102465    avgbs_107917    avgbs_109764    avgbs_109765    avgbs_110088    avgbs_110408    avgbs_111993    avgbs_111994    avgbs_118013    avgbs_118599    avgbs_119290    avgbs_119512    avgbs_122856    avgbs_123420    avgbs_123973    avgbs_125393    avgbs_125457    avgbs_125804    avgbs_126173    avgbs_6428    avgbs_233827    avgbs_234112    avgbs_234151    avgbs_234851    avgbs_235269    avgbs_235407    avgbs_236686    avgbs_238119    avgbs_241356    avgbs_242501    avgbs_242600    avgbs_243473    avgbs_243513    avgbs_222821    avgbs_243806    avgbs_244587    avgbs_244648    avgbs_4031    avgbs_8869    avgbs_27296    avgbs_43448    avgbs_46951    avgbs_50590    avgbs_81204    avgbs_83827    avgbs_99552    avgbs_109171    avgbs_115422    avgbs_216415    avgbs_239675    avgbs_240265    avgbs_7143    avgbs_15941    avgbs_15943    avgbs_18292    avgbs_32038    avgbs_37109    avgbs_68763    avgbs_71451    avgbs_99097    avgbs_118989    avgbs_238466    avgbs_205304    avgbs_222103    avgbs_242996    avgbs_8868    avgbs_40853    avgbs_40854    avgbs_47354    avgbs_65989    avgbs_74556    avgbs_95415    avgbs_239073    avgbs_221520    avgbs_157    avgbs_986    avgbs_22405    avgbs_65493    avgbs_74518    avgbs_75074    avgbs_75075    avgbs_90499    avgbs_96923    avgbs_232897    avgbs_234040    avgbs_204101    avgbs_239377    avgbs_242774    avgbs_17876    avgbs_65376    avgbs_89956    avgbs_109409    avgbs_110478    avgbs_5562    avgbs_5563    avgbs_22089    avgbs_33206    avgbs_53138    avgbs_53139    avgbs_63629    avgbs_76211    avgbs_103908    avgbs_108061    avgbs_233695    avgbs_52153    avgbs_52154    avgbs_52178    avgbs_52179    avgbs_66310    avgbs_233221    avgbs_242796    avgbs_6058    avgbs_35235    avgbs_58924    avgbs_58933    avgbs_59316    avgbs_77725    avgbs_78771    avgbs_16374    avgbs_22283    avgbs_26722    avgbs_55033    avgbs_57981    avgbs_75873    avgbs_79268    avgbs_237476    avgbs_238902    avgbs_24546    avgbs_58547    avgbs_242086    avgbs_23841    avgbs_37356    avgbs_61359    avgbs_100244    avgbs_39878    avgbs_64617    avgbs_82582    avgbs_95504    avgbs_236513    avgbs_241128    avgbs_243124    avgbs_55066    avgbs_59315    avgbs_88642    avgbs_99218    avgbs_236884    avgbs_21304    avgbs_89673    avgbs_108097    avgbs_242158    avgbs_242204    avgbs_43297    avgbs_43502    avgbs_94980    avgbs_111089    avgbs_234025    avgbs_238743    avgbs_41074    avgbs_57817    avgbs_92231    avgbs_105128    avgbs_97039    avgbs_2619    avgbs_73778    avgbs_79602    avgbs_107287    avgbs_242057    avgbs_34141    avgbs_34142    avgbs_42406    avgbs_108454    avgbs_234108    avgbs_236349    avgbs_243860    avgbs_36008    avgbs_49311    avgbs_51963    avgbs_54691    avgbs_58026    avgbs_238098    avgbs_204995    avgbs_240603    avgbs_54665    avgbs_78345    avgbs_239296    avgbs_45632    avgbs_242025    avgbs_21562    avgbs_102131    avgbs_71894    avgbs_75847    avgbs_70637    avgbs_70638    avgbs_70639    avgbs_105887    avgbs_105888    avgbs_237994    avgbs_235077    avgbs_237072    avgbs_28520    avgbs_239130    avgbs_24567    avgbs_37624    avgbs_29943    avgbs_52109    avgbs_50773    avgbs_61095    avgbs_61096    avgbs_50774    avgbs_25048    avgbs_25049    avgbs_55377    avgbs_99828    avgbs_115365    avgbs_115366    avgbs_237378    avgbs_235387    avgbs_58722   
   6C 28 
  GMI_DS_CC10035_89    
 
  avgbs_77669    avgbs_220439    avgbs_3721    avgbs_3722    avgbs_6933    avgbs_6934    avgbs_117386    avgbs_121400    avgbs_2870    avgbs_15678    avgbs_213413    avgbs_230667    avgbs_230765    avgbs_125647    avgbs_125648    avgbs_108637    avgbs_29796    avgbs_59958    avgbs_113299    avgbs_222512    avgbs_77736    avgbs_121547    avgbs_106191    avgbs_124903    avgbs_244506    avgbs_49560    avgbs_51698    avgbs_14809    avgbs_20784    avgbs_30394    avgbs_49814    avgbs_69507    avgbs_121399    avgbs_234449    avgbs_219949    avgbs_243956    avgbs_5341    avgbs_14528    avgbs_18536    avgbs_20578    avgbs_20579    avgbs_21302    avgbs_33140    avgbs_36905    avgbs_37122    avgbs_76661    avgbs_76662    avgbs_94922    avgbs_112027    avgbs_114965    avgbs_115296    avgbs_240794    avgbs_96277    avgbs_123696    avgbs_4469    avgbs_76594    avgbs_234454    avgbs_242768    avgbs_242188    avgbs_22243    avgbs_235393    avgbs_38083    avgbs_244663    avgbs_49813    avgbs_112429    avgbs_200823   
   6C 29 
  GMI_ES01_c14139_498     GMI_ES02_c349_407    
 
  avgbs_22948    avgbs_6K_24059    avgbs_29144    avgbs_43366    avgbs_44168    avgbs_44169    avgbs_48755    avgbs_59959    avgbs_59960    avgbs_66173    avgbs_68356    avgbs_84833    avgbs_96270    avgbs_102764    avgbs_118140    avgbs_123526    avgbs_216799    avgbs_216830    avgbs_217831    avgbs_218481    avgbs_221408    avgbs_200747    avgbs_223044    avgbs_4350    avgbs_6017    avgbs_10683    avgbs_20177    avgbs_21677    avgbs_29471    avgbs_51958    avgbs_56003    avgbs_64825    avgbs_67008    avgbs_76986    avgbs_89671    avgbs_89783    avgbs_91691    avgbs_95598    avgbs_105574    avgbs_105581    avgbs_105582    avgbs_216520    avgbs_219460    avgbs_200460    avgbs_222220    avgbs_200869    avgbs_45335    avgbs_41571    avgbs_83947    avgbs_89934    avgbs_95579    avgbs_211360    avgbs_68872    avgbs_10597    avgbs_29608    avgbs_51493    avgbs_67358    avgbs_67367    avgbs_81453    avgbs_90477    avgbs_90478    avgbs_217857    avgbs_39782    avgbs_66205    avgbs_66207    avgbs_105375    avgbs_28738    avgbs_38125    avgbs_39972    avgbs_41498    avgbs_41499    avgbs_43580    avgbs_66575    avgbs_98475    avgbs_208893    avgbs_219775    avgbs_204981    avgbs_120060    avgbs_222399    avgbs_550    avgbs_4285    avgbs_9549    avgbs_10476    avgbs_10933    avgbs_14667    avgbs_16192    avgbs_16193    avgbs_20073    avgbs_20196    avgbs_20559    avgbs_24192    avgbs_24515    avgbs_25105    avgbs_28846    avgbs_32541    avgbs_34625    avgbs_41954    avgbs_42369    avgbs_42597    avgbs_42680    avgbs_45430    avgbs_51068    avgbs_51481    avgbs_52578    avgbs_52579    avgbs_55636    avgbs_56107    avgbs_56941    avgbs_59785    avgbs_61130    avgbs_62046    avgbs_72058    avgbs_72503    avgbs_72706    avgbs_73229    avgbs_74435    avgbs_74943    avgbs_75922    avgbs_75923    avgbs_81219    avgbs_81265    avgbs_88923    avgbs_96002    avgbs_96003    avgbs_101299    avgbs_103077    avgbs_103823    avgbs_104016    avgbs_106959    avgbs_107279    avgbs_107280    avgbs_112433    avgbs_115083    avgbs_118260    avgbs_118940    avgbs_228677    avgbs_228898    avgbs_213255    avgbs_229131    avgbs_229189    avgbs_229360    avgbs_230032    avgbs_230259    avgbs_230264    avgbs_230290    avgbs_230392    avgbs_231320    avgbs_231496    avgbs_231577    avgbs_231685    avgbs_231728    avgbs_232026    avgbs_232388    avgbs_232634    avgbs_17063    avgbs_36908    avgbs_58660    avgbs_63921    avgbs_66364    avgbs_71720    avgbs_83264    avgbs_109691    avgbs_123749    avgbs_123811    avgbs_231365    avgbs_231557    avgbs_231647    avgbs_231781    avgbs_18253    avgbs_74308    avgbs_77192    avgbs_77193    avgbs_84040    avgbs_84043    avgbs_95017    avgbs_95018    avgbs_108131    avgbs_117153    avgbs_231047    avgbs_232272    avgbs_437    avgbs_13846    avgbs_18440    avgbs_28592    avgbs_35952    avgbs_40490    avgbs_53178    avgbs_78644    avgbs_78820    avgbs_117775    avgbs_117981    avgbs_229289    avgbs_230322    avgbs_207557    avgbs_231722    avgbs_6109    avgbs_6110    avgbs_33039    avgbs_35243    avgbs_36465    avgbs_50262    avgbs_71884    avgbs_81959    avgbs_82226    avgbs_125821    avgbs_229949    avgbs_52407    avgbs_9047    avgbs_9048    avgbs_16433    avgbs_16434    avgbs_16435    avgbs_59544    avgbs_60481    avgbs_60482    avgbs_89749    avgbs_98450    avgbs_93087    avgbs_231690    avgbs_549    avgbs_2924    avgbs_5583    avgbs_6977    avgbs_7968    avgbs_22309    avgbs_25246    avgbs_33040    avgbs_36089    avgbs_36464    avgbs_40679    avgbs_40680    avgbs_43747    avgbs_45410    avgbs_45617    avgbs_46775    avgbs_54212    avgbs_57300    avgbs_57302    avgbs_57958    avgbs_57959    avgbs_57960    avgbs_58102    avgbs_58231    avgbs_59626    avgbs_61711    avgbs_67290    avgbs_71885    avgbs_76478    avgbs_76850    avgbs_79008    avgbs_80100    avgbs_81395    avgbs_89143    avgbs_90463    avgbs_90464    avgbs_90479    avgbs_94530    avgbs_95995    avgbs_106052    avgbs_110746    avgbs_114220    avgbs_121541    avgbs_125905    avgbs_230091    avgbs_231008    avgbs_231063    avgbs_231112    avgbs_231140    avgbs_231609    avgbs_232438    avgbs_232473    avgbs_232804    avgbs_91785    avgbs_99911    avgbs_111072    avgbs_117772    avgbs_231200    avgbs_232538    avgbs_4365    avgbs_78384    avgbs_26534    avgbs_2398    avgbs_114463    avgbs_18589    avgbs_234930    avgbs_113916    avgbs_243116    avgbs_24341    avgbs_118815    avgbs_124442    avgbs_240854    avgbs_222784    avgbs_244450    avgbs_9961    avgbs_234023    avgbs_114078    avgbs_236567    avgbs_69639    avgbs_39426    avgbs_1922    avgbs_18485    avgbs_18546    avgbs_40605    avgbs_41365    avgbs_61129    avgbs_76475    avgbs_78916    avgbs_99240    avgbs_99241    avgbs_110764    avgbs_236450    avgbs_237894    avgbs_3723    avgbs_46161    avgbs_91331    avgbs_233557    avgbs_10626    avgbs_15557    avgbs_17930    avgbs_19762    avgbs_20726    avgbs_23092    avgbs_39336    avgbs_49254    avgbs_49255    avgbs_55283    avgbs_65908    avgbs_66120    avgbs_68704    avgbs_76548    avgbs_78950    avgbs_81057    avgbs_82499    avgbs_99212    avgbs_99213    avgbs_108049    avgbs_112393    avgbs_112488    avgbs_114208    avgbs_114913    avgbs_117281    avgbs_117343    avgbs_122241    avgbs_125993    avgbs_237504    avgbs_242647    avgbs_242827    avgbs_243901    avgbs_16491    avgbs_17155    avgbs_59335    avgbs_63320    avgbs_80860    avgbs_86158    avgbs_86159    avgbs_105753    avgbs_5936    avgbs_5937    avgbs_7021    avgbs_16493    avgbs_18269    avgbs_22048    avgbs_28229    avgbs_30399    avgbs_36262    avgbs_38840    avgbs_38841    avgbs_38842    avgbs_39162    avgbs_39319    avgbs_40219    avgbs_47497    avgbs_52069    avgbs_52776    avgbs_52963    avgbs_59762    avgbs_67989    avgbs_68463    avgbs_72307    avgbs_74307    avgbs_74882    avgbs_75918    avgbs_77923    avgbs_79119    avgbs_86423    avgbs_90120    avgbs_99186    avgbs_101984    avgbs_104552    avgbs_106210    avgbs_110867    avgbs_112095    avgbs_112103    avgbs_112434    avgbs_112618    avgbs_115026    avgbs_115546    avgbs_122240    avgbs_122245    avgbs_122614    avgbs_124918    avgbs_236499    avgbs_220196    avgbs_240229    avgbs_240230    avgbs_241666    avgbs_105802    avgbs_242692    avgbs_243225    avgbs_244160    avgbs_16689    avgbs_39619    avgbs_39751    avgbs_58934    avgbs_61519    avgbs_94568    avgbs_116966    avgbs_122114    avgbs_123149    avgbs_239485    avgbs_242116    avgbs_244101    avgbs_8043    avgbs_16100    avgbs_17387    avgbs_20935    avgbs_21265    avgbs_29968    avgbs_30351    avgbs_34520    avgbs_37101    avgbs_37426    avgbs_42598    avgbs_47060    avgbs_47169    avgbs_51435    avgbs_52594    avgbs_54123    avgbs_59386    avgbs_65419    avgbs_65553    avgbs_65767    avgbs_67571    avgbs_73234    avgbs_76620    avgbs_83755    avgbs_88457    avgbs_89462    avgbs_91591    avgbs_92944    avgbs_93054    avgbs_99884    avgbs_99986    avgbs_100261    avgbs_6K_95496    avgbs_105356    avgbs_105932    avgbs_108888    avgbs_110635    avgbs_112073    avgbs_114536    avgbs_115432    avgbs_116301    avgbs_120491    avgbs_121966    avgbs_122087    avgbs_234156    avgbs_234170    avgbs_235260    avgbs_236630    avgbs_236946    avgbs_237315    avgbs_237623    avgbs_238179    avgbs_238770    avgbs_239042    avgbs_240534    avgbs_241628    avgbs_222290    avgbs_242400    avgbs_242669    avgbs_242697    avgbs_244097    avgbs_14928    avgbs_54639    avgbs_76207    avgbs_76208    avgbs_78505    avgbs_93989    avgbs_100140    avgbs_104514    avgbs_107902    avgbs_108065    avgbs_111343    avgbs_120381    avgbs_125751    avgbs_236476    avgbs_219090    avgbs_239085    avgbs_240286    avgbs_241302    avgbs_7911    avgbs_25174    avgbs_59884    avgbs_67542    avgbs_71094    avgbs_78352    avgbs_86362    avgbs_92422    avgbs_108191    avgbs_109833    avgbs_109834    avgbs_114330    avgbs_114336    avgbs_115923    avgbs_123141    avgbs_124785    avgbs_237478    avgbs_241886    avgbs_244293    avgbs_912    avgbs_36638    avgbs_37699    avgbs_37916    avgbs_37917    avgbs_42877    avgbs_57196    avgbs_57197    avgbs_57198    avgbs_105194    avgbs_113463    avgbs_234799    avgbs_236381    avgbs_240772    avgbs_206800    avgbs_242673    avgbs_242745    avgbs_244280    avgbs_244599    avgbs_2615    avgbs_24179    avgbs_36086    avgbs_38126    avgbs_43365    avgbs_51747    avgbs_63374    avgbs_63774    avgbs_70476    avgbs_86207    avgbs_90648    avgbs_103665    avgbs_112347    avgbs_125369    avgbs_235509    avgbs_236449    avgbs_243941    avgbs_244465    avgbs_40503    avgbs_79251    avgbs_80409    avgbs_110694    avgbs_115509    avgbs_236296    avgbs_236885    avgbs_237760    avgbs_243238    avgbs_207938    avgbs_244474    avgbs_8607    avgbs_19344    avgbs_38144    avgbs_43643    avgbs_48245    avgbs_56829    avgbs_59735    avgbs_85810    avgbs_85811    avgbs_89759    avgbs_90970    avgbs_107530    avgbs_111373    avgbs_113561    avgbs_113562    avgbs_115434    avgbs_123199    avgbs_123200    avgbs_125011    avgbs_125130    avgbs_125786    avgbs_233507    avgbs_239196    avgbs_243935    avgbs_16561    avgbs_56320    avgbs_61299    avgbs_64888    avgbs_67964    avgbs_78108    avgbs_78317    avgbs_78492    avgbs_88453    avgbs_100039    avgbs_112708    avgbs_112709    avgbs_234661    avgbs_235859    avgbs_239190    avgbs_241711    avgbs_7162    avgbs_29380    avgbs_35218    avgbs_52304    avgbs_54081    avgbs_54082    avgbs_63799    avgbs_66181    avgbs_66183    avgbs_72226    avgbs_80470    avgbs_108452    avgbs_233229    avgbs_234441    avgbs_239929    avgbs_241368    avgbs_241656    avgbs_243801    avgbs_244133    avgbs_8614    avgbs_28613    avgbs_75808    avgbs_106656    avgbs_116308    avgbs_234452    avgbs_234979    avgbs_237677    avgbs_239027    avgbs_240500    avgbs_241888    avgbs_242605    avgbs_243266    avgbs_243290    avgbs_39132    avgbs_40908    avgbs_41575    avgbs_54549    avgbs_57279    avgbs_74799    avgbs_102873    avgbs_103468    avgbs_125845    avgbs_234660    avgbs_218940    avgbs_239490    avgbs_243840    avgbs_2497    avgbs_29147    avgbs_40224    avgbs_43050    avgbs_57200    avgbs_74023    avgbs_74024    avgbs_74785    avgbs_99697    avgbs_99698    avgbs_119018    avgbs_234903    avgbs_218349    avgbs_239347    avgbs_205051    avgbs_241967    avgbs_243773    avgbs_14817    avgbs_39745    avgbs_39746    avgbs_49281    avgbs_53523    avgbs_55379    avgbs_87478    avgbs_219013    avgbs_237513    avgbs_244695    avgbs_21461    avgbs_29687    avgbs_53966    avgbs_58492    avgbs_67271    avgbs_81452    avgbs_103402    avgbs_236298    avgbs_237533    avgbs_240232    avgbs_212217    avgbs_16896    avgbs_23090    avgbs_25040    avgbs_57098    avgbs_58650    avgbs_64497    avgbs_68873    avgbs_78556    avgbs_237279    avgbs_238184    avgbs_244087    avgbs_235462    avgbs_238868    avgbs_239200    avgbs_244639    avgbs_24787    avgbs_24788    avgbs_34010    avgbs_52187    avgbs_64047    avgbs_88387    avgbs_91359    avgbs_105949    avgbs_115308    avgbs_233232    avgbs_218944    avgbs_1034    avgbs_8931    avgbs_10190    avgbs_10191    avgbs_52012    avgbs_60866    avgbs_233295    avgbs_237262    avgbs_219438    avgbs_242606    avgbs_244096    avgbs_25637    avgbs_29359    avgbs_30332    avgbs_57891    avgbs_80143    avgbs_124219    avgbs_124220    avgbs_236477    avgbs_237727    avgbs_238269    avgbs_238729    avgbs_241493    avgbs_29641    avgbs_43599    avgbs_60464    avgbs_108062    avgbs_236479    avgbs_219477    avgbs_220507    avgbs_240851    avgbs_242155    avgbs_242721    avgbs_3725    avgbs_57343    avgbs_58978    avgbs_58979    avgbs_61530    avgbs_74842    avgbs_109421    avgbs_120755    avgbs_235845    avgbs_238281    avgbs_5747    avgbs_43602    avgbs_51671    avgbs_70042    avgbs_101655    avgbs_116408    avgbs_236614    avgbs_239737    avgbs_39721    avgbs_48105    avgbs_124168    avgbs_124696    avgbs_237000    avgbs_9116    avgbs_36259    avgbs_42130    avgbs_45648    avgbs_72637    avgbs_73938    avgbs_97500    avgbs_97501    avgbs_101852    avgbs_120448    avgbs_120449    avgbs_233519    avgbs_234542    avgbs_219810    avgbs_242945    avgbs_244718    avgbs_29146    avgbs_75767    avgbs_86219    avgbs_114471    avgbs_122959    avgbs_244231    avgbs_10430    avgbs_40686    avgbs_60152    avgbs_78380    avgbs_103477    avgbs_243373    avgbs_25648    avgbs_69659    avgbs_235614    avgbs_238520    avgbs_33257    avgbs_41181    avgbs_56421    avgbs_106094    avgbs_109314    avgbs_236025    avgbs_236322    avgbs_238295    avgbs_240699    avgbs_113446    avgbs_236305    avgbs_242859    avgbs_1234    avgbs_27768    avgbs_63959    avgbs_63960    avgbs_80475    avgbs_234972    avgbs_239969    avgbs_47839    avgbs_112608    avgbs_237499    avgbs_19088    avgbs_30531    avgbs_30532    avgbs_39800    avgbs_61643    avgbs_239174    avgbs_50721    avgbs_53240    avgbs_103818    avgbs_232119    avgbs_242995    avgbs_236026    avgbs_31692    avgbs_48773    avgbs_63505    avgbs_113443    avgbs_118851    avgbs_118852    avgbs_24652    avgbs_64271    avgbs_235831    avgbs_238163    avgbs_215945    avgbs_22372    avgbs_224427    avgbs_53698    avgbs_54475    avgbs_92515    avgbs_224306    avgbs_106246    avgbs_122617    avgbs_215670    avgbs_118916   
   6C 30 
  GMI_ES02_c8298_599     GMI_ES_CC9431_260     GMI_ES02_c17191_397     GMI_DS_CC7795_77    
 
  avgbs_20560    avgbs_48892    avgbs_52593    avgbs_68055    avgbs_99719    avgbs_99720    avgbs_120130    avgbs_120422    avgbs_218671    avgbs_215845    avgbs_222747    avgbs_222870    avgbs_223055    avgbs_2329    avgbs_2330    avgbs_10422    avgbs_16013    avgbs_22930    avgbs_24958    avgbs_42678    avgbs_42679    avgbs_43013    avgbs_45518    avgbs_47168    avgbs_72542    avgbs_75921    avgbs_81530    avgbs_84274    avgbs_97675    avgbs_101595    avgbs_104278    avgbs_115141    avgbs_125175    avgbs_213035    avgbs_217522    avgbs_213626    avgbs_218588    avgbs_200542    avgbs_200733    avgbs_200842    avgbs_200940    avgbs_12669    avgbs_47601    avgbs_50818    avgbs_50819    avgbs_50820    avgbs_51986    avgbs_103291    avgbs_210254    avgbs_5679    avgbs_96500    avgbs_123152    avgbs_214262    avgbs_59930    avgbs_59931    avgbs_6353    avgbs_35302    avgbs_56602    avgbs_56604    avgbs_72953    avgbs_93007    avgbs_96924    avgbs_99183    avgbs_110908    avgbs_110909    avgbs_121401    avgbs_123153    avgbs_200258    avgbs_8108    avgbs_8109    avgbs_8110    avgbs_55067    avgbs_107424    avgbs_105224    avgbs_1533    avgbs_14956    avgbs_20197    avgbs_20198    avgbs_39509    avgbs_59969    avgbs_77973    avgbs_85886    avgbs_99242    avgbs_104892    avgbs_105185    avgbs_109828    avgbs_110564    avgbs_113038    avgbs_237813    avgbs_243492    avgbs_64040    avgbs_238143    avgbs_70552    avgbs_68702    avgbs_15953    avgbs_15954    avgbs_219237    avgbs_21770    avgbs_22608   
   6C 31 
 
 
  avgbs_23115    avgbs_243793    avgbs_30115    avgbs_40218    avgbs_89855    avgbs_93086    avgbs_107901    avgbs_39405    avgbs_54318    avgbs_59968    avgbs_67865    avgbs_84997    avgbs_93044    avgbs_97306    avgbs_234463    avgbs_237248    avgbs_238935    avgbs_16128    avgbs_17298    avgbs_113596    avgbs_125976    avgbs_108278    avgbs_76805    avgbs_236277    avgbs_83543    avgbs_14763    avgbs_58337    avgbs_234438    avgbs_54397    avgbs_54398    avgbs_86593    avgbs_99829    avgbs_234124    avgbs_105171    avgbs_105172    avgbs_239930    avgbs_91774    avgbs_99547    avgbs_99548    avgbs_36260    avgbs_59092    avgbs_234540    avgbs_86079    avgbs_239942    avgbs_236354    avgbs_238991    avgbs_66387   
   6C 32 
  GMI_ES01_c17064_407    
 
  avgbs_65592    avgbs_65593    avgbs_108961    avgbs_51929    avgbs_78224    avgbs_90096    avgbs_90187    avgbs_3844    avgbs_123917    avgbs_233206    avgbs_243229    avgbs_16775    avgbs_234685    avgbs_100318    avgbs_244168    avgbs_123427    avgbs_50542    avgbs_9046    avgbs_225335   
   6C 33 
 
 
  avgbs_233009    avgbs_73764    avgbs_238590    avgbs_57101    avgbs_89377    avgbs_201876    avgbs_103463    avgbs_30090    avgbs_242532    avgbs_22336    avgbs_238938    avgbs_51142    avgbs_51315    avgbs_55282   
   6C 35 
 
 
  avgbs_42993    avgbs_101656    avgbs_7677    avgbs_76617    avgbs_215567   
   6C 36 
 
 
  avgbs_20311    avgbs_21863    avgbs_236934    avgbs_219141   
   6C 37 
 
 
  avgbs_79455    avgbs_207933    avgbs_240410    avgbs_124778   
   6C 38 
 
 
  avgbs_40213    avgbs_106797    avgbs_106798    avgbs_106799    avgbs_238427    avgbs_76619    avgbs_239038   
   6C 39 
 
 
  avgbs_36056    avgbs_200243    avgbs_220257    avgbs_3726    avgbs_220611   
   6C 40 
 
 
  avgbs_60084    avgbs_60086    avgbs_47187    avgbs_16518    avgbs_40841    avgbs_213985    avgbs_69444    avgbs_17721    avgbs_91041    avgbs_215272   
   6C 41 
 
 
  avgbs_205660    avgbs_35936    avgbs_216698    avgbs_235116   
   6C 42 
 
 
  avgbs_74788    avgbs_52837    avgbs_208362    avgbs_242270   
   6C 43 
 
 
  avgbs_53710    avgbs_19590   
   6C 44 
 
 
  avgbs_14876    avgbs_234331   
   6C 46 
  GMI_ES15_c14048_223     GMI_ES02_c2118_202    
 
  avgbs_119    avgbs_120    avgbs_25574    avgbs_64547    avgbs_72083    avgbs_72084    avgbs_74255    avgbs_93566    avgbs_100188    avgbs_121684    avgbs_203313    avgbs_214600    avgbs_200504    avgbs_74925    avgbs_74926    avgbs_92213    avgbs_105315    avgbs_113594    avgbs_120188    avgbs_200580    avgbs_200614    avgbs_212290    avgbs_6568    avgbs_20620    avgbs_42010    avgbs_75872    avgbs_115020    avgbs_115021    avgbs_23111    avgbs_6K_51953    avgbs_208746    avgbs_43561    avgbs_43562    avgbs_72667    avgbs_213859    avgbs_204501    avgbs_34890    avgbs_87398    avgbs_215057    avgbs_2763    avgbs_20293    avgbs_106395    avgbs_106396    avgbs_33003    avgbs_213819    avgbs_83233    avgbs_109139    avgbs_215733    avgbs_20292    avgbs_51540    avgbs_41448    avgbs_215955    avgbs_25322    avgbs_86509    avgbs_70542    avgbs_70543    avgbs_43574    avgbs_43575    avgbs_65269    avgbs_65270    avgbs_77473    avgbs_106343    avgbs_200534    avgbs_14699    avgbs_52955    avgbs_67600    avgbs_115169    avgbs_115170    avgbs_115171   
   6C 47 
 
 
  avgbs_117384    avgbs_118099    avgbs_118100    avgbs_118101    avgbs_118102    avgbs_107478    avgbs_107480   
   6C 48 
 
 
  avgbs_104536    avgbs_206239    avgbs_3672    avgbs_26073    avgbs_26074    avgbs_242298   
   6C 49 
 
 
  avgbs_10936    avgbs_59597    avgbs_83392    avgbs_201709    avgbs_239631    avgbs_57487    avgbs_57488   
   6C 50 
 
 
  avgbs_203634    avgbs_3671    avgbs_36785    avgbs_36786    avgbs_58631    avgbs_116851    avgbs_116852   
   6C 51 
 
 
  avgbs_105311    avgbs_8180    avgbs_91833    avgbs_74467    avgbs_82862    avgbs_200918    avgbs_62194    avgbs_204018    avgbs_207863   
   6C 52 
 
 
  avgbs_77857    avgbs_29004    avgbs_71650    avgbs_204431   
   6C 53 
 
 
  avgbs_92160    avgbs_92162    avgbs_22607   
   6C 54 
 
 
  avgbs_11823    avgbs_119204    avgbs_93117    avgbs_33957   
   6C 55 
 
 
  avgbs_13322    avgbs_13323    avgbs_62193    avgbs_204019    avgbs_34963    avgbs_41442    avgbs_7916    avgbs_76872    avgbs_76873   
   6C 56 
 
 
  avgbs_202418   
   6C 57 
 
 
  avgbs_120490    avgbs_34110    avgbs_112855    avgbs_100032   
   6C 58 
  GMI_ES17_c5637_152    
 
  avgbs_7970    avgbs_11093    avgbs_11094    avgbs_11524    avgbs_19563    avgbs_213295    avgbs_213506    avgbs_70782    avgbs_13402    avgbs_50026    avgbs_57698    avgbs_57699    avgbs_125586    avgbs_201812    avgbs_11994    avgbs_24840    avgbs_124431    avgbs_219568    avgbs_114054    avgbs_57033    avgbs_68132    avgbs_98315    avgbs_119109    avgbs_33656    avgbs_68617    avgbs_102039    avgbs_110982    avgbs_118479    avgbs_4176    avgbs_8571    avgbs_69697    avgbs_69698    avgbs_222038    avgbs_4679    avgbs_4680    avgbs_58047    avgbs_69707    avgbs_73646    avgbs_239428    avgbs_18646    avgbs_80393    avgbs_80394    avgbs_97356    avgbs_200699    avgbs_244707    avgbs_89867    avgbs_37829    avgbs_236373    avgbs_71801    avgbs_207471    avgbs_56053    avgbs_6375    avgbs_94349   
   6C 59 
  GMI_DS_CC3774_51    
 
  avgbs_206883    avgbs_4982    avgbs_120489    avgbs_201250    avgbs_17342    avgbs_103070    avgbs_105313    avgbs_200113    avgbs_206146    avgbs_62515    avgbs_212990    avgbs_5921    avgbs_65619    avgbs_204181    avgbs_52670    avgbs_200772    avgbs_205240    avgbs_108152    avgbs_99932    avgbs_107332    avgbs_107334    avgbs_21547    avgbs_35038    avgbs_35039    avgbs_233420    avgbs_121433    avgbs_207936    avgbs_105701   
   6C 60 
 
 
  avgbs_12289    avgbs_123047    avgbs_29727    avgbs_29728    avgbs_67049    avgbs_123046    avgbs_200691    avgbs_109406    avgbs_203401   
   6C 61 
  GMI_ES01_c2163_409     GMI_ES17_c21168_588     GMI_ES_CC17866_90    
 
  avgbs_57403    avgbs_115301    avgbs_123740    avgbs_215439    avgbs_205839    avgbs_206615    avgbs_222772    avgbs_32110    avgbs_45088    avgbs_77246    avgbs_81708    avgbs_104755    avgbs_104756    avgbs_116686    avgbs_117365    avgbs_200531    avgbs_205022    avgbs_6273    avgbs_3741    avgbs_13084    avgbs_13085    avgbs_111016    avgbs_49874    avgbs_49875    avgbs_91808    avgbs_109295    avgbs_71114    avgbs_7917    avgbs_38892    avgbs_73879    avgbs_99504    avgbs_110091    avgbs_201092    avgbs_224017    avgbs_227022    avgbs_231085    avgbs_215528    avgbs_3382    avgbs_4829    avgbs_25439    avgbs_25440    avgbs_67519    avgbs_67520    avgbs_87689    avgbs_90218    avgbs_92425    avgbs_105659    avgbs_106439    avgbs_109000    avgbs_214163    avgbs_204386    avgbs_205489    avgbs_206291    avgbs_206394    avgbs_206667    avgbs_69538    avgbs_76160    avgbs_59317    avgbs_69187    avgbs_78214    avgbs_106910    avgbs_56253    avgbs_6032    avgbs_108377    avgbs_108378    avgbs_66062    avgbs_106729    avgbs_106730    avgbs_205553    avgbs_3414    avgbs_47929    avgbs_54011    avgbs_54012    avgbs_102355    avgbs_102356    avgbs_121339    avgbs_26876    avgbs_47287    avgbs_58293    avgbs_121424    avgbs_203017    avgbs_11666    avgbs_20712    avgbs_25110    avgbs_27504    avgbs_27505    avgbs_29031    avgbs_53187    avgbs_53188    avgbs_55051    avgbs_55052    avgbs_88745    avgbs_105568    avgbs_110773    avgbs_117366    avgbs_121088    avgbs_202495    avgbs_203135    avgbs_205121    avgbs_221069    avgbs_244583    avgbs_73970    avgbs_25152    avgbs_48919    avgbs_64048    avgbs_103216    avgbs_103217    avgbs_115117    avgbs_234324    avgbs_236977    avgbs_204065    avgbs_242430    avgbs_206963    avgbs_207713    avgbs_17392    avgbs_58495    avgbs_106347    avgbs_229408    avgbs_206384    avgbs_26845    avgbs_111748    avgbs_55584    avgbs_15627    avgbs_95578    avgbs_68228    avgbs_110448    avgbs_235109    avgbs_201950    avgbs_100314    avgbs_242864    avgbs_9456    avgbs_96804    avgbs_96805    avgbs_243271    avgbs_233931    avgbs_86349    avgbs_205749    avgbs_123356    avgbs_113361    avgbs_113362    avgbs_64819    avgbs_64820    avgbs_66070    avgbs_104714    avgbs_202024    avgbs_13276    avgbs_116255    avgbs_200857    avgbs_56854    avgbs_225520    avgbs_86488    avgbs_223932    avgbs_223303    avgbs_62704    avgbs_97323    avgbs_48278    avgbs_19989    avgbs_223610    avgbs_111259    avgbs_5002    avgbs_32823    avgbs_223869   
   6C 62 
 
 
  avgbs_7865    avgbs_102866    avgbs_87221    avgbs_102865    avgbs_110090    avgbs_200802    avgbs_63406    avgbs_122919    avgbs_229348    avgbs_230578    avgbs_70680    avgbs_90914    avgbs_101117    avgbs_48920    avgbs_56254    avgbs_31644    avgbs_94257    avgbs_231750    avgbs_116088   
   6C 63 
  GMI_ES17_c4515_297    
 
  avgbs_124188    avgbs_124190    avgbs_25810    avgbs_79131    avgbs_19594    avgbs_48426    avgbs_91849    avgbs_22597    avgbs_106887    avgbs_108517    avgbs_222650    avgbs_104142    avgbs_104143    avgbs_40325    avgbs_52091    avgbs_52092    avgbs_117056    avgbs_210444    avgbs_46013    avgbs_57490    avgbs_57849    avgbs_65314    avgbs_82766    avgbs_224366    avgbs_231332    avgbs_1497    avgbs_82831    avgbs_109405    avgbs_229709    avgbs_114280    avgbs_228356    avgbs_20743    avgbs_100595    avgbs_6987    avgbs_1496    avgbs_21465    avgbs_16780    avgbs_73285    avgbs_120970    avgbs_120971    avgbs_120972    avgbs_124589    avgbs_25833    avgbs_49105    avgbs_203536    avgbs_4020    avgbs_241287    avgbs_50266    avgbs_204795    avgbs_6K_81876   
   6C 64 
  GMI_ES02_c7632_364     GMI_ES_CC7312_286    
 
  avgbs_15594    avgbs_39012    avgbs_8227    avgbs_100722    avgbs_101122    avgbs_201521    avgbs_6359    avgbs_11056    avgbs_15269    avgbs_6K_32694    avgbs_38674    avgbs_38675    avgbs_38676    avgbs_48707    avgbs_21220    avgbs_44791    avgbs_119886    avgbs_119887    avgbs_52896    avgbs_229677    avgbs_32237    avgbs_111660    avgbs_111661    avgbs_111662   
   6C 65 
 
 
  avgbs_58045    avgbs_58046    avgbs_202329    avgbs_21466    avgbs_24449    avgbs_24450    avgbs_28952    avgbs_28953    avgbs_219796    avgbs_18664    avgbs_66191    avgbs_720    avgbs_47538    avgbs_200312    avgbs_15935    avgbs_96492    avgbs_97324    avgbs_98276    avgbs_230912    avgbs_227986    avgbs_81773    avgbs_119782    avgbs_227867    avgbs_75876    avgbs_116122    avgbs_51968    avgbs_6K_107999    avgbs_56230    avgbs_232113    avgbs_64386    avgbs_6372    avgbs_6373    avgbs_42053    avgbs_7452    avgbs_56855    avgbs_92656    avgbs_104276    avgbs_107368    avgbs_203876    avgbs_205286    avgbs_60752    avgbs_206867    avgbs_86683    avgbs_220953   
   6C 66 
 
 
  avgbs_3300    avgbs_26497    avgbs_28371    avgbs_97062    avgbs_97063    avgbs_229951    avgbs_319    avgbs_320    avgbs_9617    avgbs_14221    avgbs_14222    avgbs_24715    avgbs_6K_29683    avgbs_50708    avgbs_67017    avgbs_67018    avgbs_98950    avgbs_98951    avgbs_98952    avgbs_107513    avgbs_123803    avgbs_202385    avgbs_19325    avgbs_19326    avgbs_65978    avgbs_229817    avgbs_229957    avgbs_42180    avgbs_52543    avgbs_230233    avgbs_230666    avgbs_214095    avgbs_10774    avgbs_92657    avgbs_107718    avgbs_84077    avgbs_84078    avgbs_217070    avgbs_38627   
   6C 67 
 
 
  avgbs_202754    avgbs_9927    avgbs_49218    avgbs_63518    avgbs_107485    avgbs_201632    avgbs_8444    avgbs_84577    avgbs_63283    avgbs_10324    avgbs_28405    avgbs_35408    avgbs_46468    avgbs_84225    avgbs_6K_80617    avgbs_88555    avgbs_96745    avgbs_108516    avgbs_200273    avgbs_230267    avgbs_230504    avgbs_200533    avgbs_205111    avgbs_231860    avgbs_232282    avgbs_65979    avgbs_75782    avgbs_102800    avgbs_20877    avgbs_60231    avgbs_51110    avgbs_104324    avgbs_109308    avgbs_22007   
   6C 68 
  GMI_DS_CC5652_132    
 
  avgbs_32141    avgbs_62153    avgbs_118051    avgbs_219806    avgbs_19764    avgbs_32055    avgbs_213513    avgbs_122920    avgbs_124430    avgbs_4664    avgbs_23639    avgbs_35409    avgbs_56529    avgbs_56530    avgbs_56531    avgbs_67852    avgbs_87058    avgbs_97056    avgbs_103080    avgbs_118142    avgbs_120100    avgbs_229445    avgbs_230941    avgbs_2468    avgbs_2469    avgbs_32056    avgbs_87137    avgbs_231028    avgbs_231844    avgbs_102244    avgbs_230120    avgbs_112424    avgbs_112425    avgbs_232387    avgbs_79109    avgbs_209314    avgbs_20394    avgbs_85431    avgbs_85432    avgbs_10576   
   6C 69 
 
 
  avgbs_10417    avgbs_14689    avgbs_217108    avgbs_1217    avgbs_63146    avgbs_39461    avgbs_64152    avgbs_106933    avgbs_106934    avgbs_48706    avgbs_200126   
   6C 70 
 
 
  avgbs_7781    avgbs_13238    avgbs_38609    avgbs_120415    avgbs_11720    avgbs_213312    avgbs_844    avgbs_50063    avgbs_99726    avgbs_99727    avgbs_235396    avgbs_239588    avgbs_11922    avgbs_11923    avgbs_11538    avgbs_19353    avgbs_213306    avgbs_13214    avgbs_204906   
   6C 71 
 
 
  avgbs_20545    avgbs_93260    avgbs_211722    avgbs_56169    avgbs_51967    avgbs_108786    avgbs_226480    avgbs_15997    avgbs_209146    avgbs_121013    avgbs_118937    avgbs_118938    avgbs_118939    avgbs_95994   
   6C 72 
 
 
  avgbs_7474    avgbs_7475    avgbs_221706    avgbs_67080    avgbs_230797    avgbs_75213    avgbs_61458    avgbs_93021    avgbs_3724    avgbs_10603    avgbs_29726    avgbs_62892   
   6C 73 
  GMI_ES01_c5633_477     GMI_ES15_c3806_357    
 
  avgbs_126333    avgbs_62821    avgbs_63384    avgbs_79573    avgbs_216491    avgbs_218233    avgbs_14655    avgbs_34270    avgbs_25378    avgbs_34271    avgbs_34825    avgbs_8784    avgbs_4114    avgbs_225175    avgbs_86087    avgbs_86612    avgbs_2229    avgbs_20242    avgbs_25875    avgbs_25876    avgbs_52017    avgbs_116460    avgbs_116461    avgbs_228736    avgbs_229349    avgbs_232299    avgbs_105279    avgbs_232812    avgbs_33858    avgbs_220052    avgbs_79446    avgbs_79447    avgbs_105370    avgbs_105371    avgbs_105372    avgbs_39543   
   6C 74 
 
 
  avgbs_62749    avgbs_41236    avgbs_41401    avgbs_41402    avgbs_73233    avgbs_82555    avgbs_123314    avgbs_124189    avgbs_229991    avgbs_231323    avgbs_232463    avgbs_232729    avgbs_65338    avgbs_204515    avgbs_110427    avgbs_222368    avgbs_7216   
   6C 75 
 
 
  avgbs_23216    avgbs_30621    avgbs_30622    avgbs_30623    avgbs_64064    avgbs_64065    avgbs_114887    avgbs_230451    avgbs_231246    avgbs_231256    avgbs_232198    avgbs_232540    avgbs_31697    avgbs_77697    avgbs_95677    avgbs_106195    avgbs_229421    avgbs_229693    avgbs_231170    avgbs_200941   
   6C 76 
 
 
  avgbs_6924    avgbs_6925    avgbs_8016    avgbs_13589    avgbs_84424    avgbs_116846    avgbs_116847    avgbs_216661    avgbs_225734    avgbs_28754    avgbs_28755    avgbs_754    avgbs_21735    avgbs_83076    avgbs_83077    avgbs_83078    avgbs_17791    avgbs_17792    avgbs_91628    avgbs_109548    avgbs_85964    avgbs_15807    avgbs_216170    avgbs_11289   
   6C 77 
  GMI_DS_A3_435_344    
 
  avgbs_7126    avgbs_110761    avgbs_221908    avgbs_206831    avgbs_12391    avgbs_12392    avgbs_14245    avgbs_14246    avgbs_70364    avgbs_115091    avgbs_220000    avgbs_16088    avgbs_16089    avgbs_232543    avgbs_11848    avgbs_76178    avgbs_88605    avgbs_91231    avgbs_97881    avgbs_117782    avgbs_230879    avgbs_32847    avgbs_107602    avgbs_107603    avgbs_90882    avgbs_109737    avgbs_54516    avgbs_117917    avgbs_219081    avgbs_34131    avgbs_105590    avgbs_33172    avgbs_85191    avgbs_231445    avgbs_109664    avgbs_111982    avgbs_103079    avgbs_111945    avgbs_111946    avgbs_200750    avgbs_102006    avgbs_121980    avgbs_121981    avgbs_26780    avgbs_65380    avgbs_93931    avgbs_102728    avgbs_239397    avgbs_15000    avgbs_100431    avgbs_102442    avgbs_102443    avgbs_208006    avgbs_6757    avgbs_6758    avgbs_116872    avgbs_237313    avgbs_27249    avgbs_11039    avgbs_243719   
   6C 78 
  GMI_ES02_c247_241    
 
  avgbs_13389    avgbs_39098    avgbs_59298    avgbs_72623    avgbs_80495    avgbs_95773    avgbs_120585    avgbs_217011    avgbs_200130    avgbs_218485    avgbs_214424    avgbs_219809    avgbs_221372    avgbs_223126    avgbs_2424    avgbs_26383    avgbs_33025    avgbs_33026    avgbs_39485    avgbs_39486    avgbs_53157    avgbs_53161    avgbs_53162    avgbs_70287    avgbs_71471    avgbs_71472    avgbs_75122    avgbs_80238    avgbs_94131    avgbs_105151    avgbs_105152    avgbs_109115    avgbs_110525    avgbs_120456    avgbs_124149    avgbs_124264    avgbs_124265    avgbs_125292    avgbs_218105    avgbs_218363    avgbs_220598    avgbs_200795    avgbs_1909    avgbs_98842    avgbs_11707    avgbs_11708    avgbs_46081    avgbs_49911    avgbs_75366    avgbs_220334    avgbs_110740    avgbs_51509    avgbs_218917    avgbs_70400    avgbs_55120    avgbs_117283    avgbs_29888    avgbs_6K_78949    avgbs_62889    avgbs_115271    avgbs_6836    avgbs_13041    avgbs_13042    avgbs_56589    avgbs_59051    avgbs_59052    avgbs_59053    avgbs_74110    avgbs_79448    avgbs_80496    avgbs_98816    avgbs_100931    avgbs_118290    avgbs_122489    avgbs_122612    avgbs_231999    avgbs_232546    avgbs_232559    avgbs_232689    avgbs_1908    avgbs_2683    avgbs_2684    avgbs_5006    avgbs_8996    avgbs_11532    avgbs_14395    avgbs_16087    avgbs_19800    avgbs_28149    avgbs_58620    avgbs_90819    avgbs_95941    avgbs_101313    avgbs_107328    avgbs_116912    avgbs_124266    avgbs_124267    avgbs_229959    avgbs_204388    avgbs_200740    avgbs_232516    avgbs_91794    avgbs_93728    avgbs_93729    avgbs_117974    avgbs_229759    avgbs_232497    avgbs_25254    avgbs_29213    avgbs_65330    avgbs_65331    avgbs_100033    avgbs_33951    avgbs_38098    avgbs_38099    avgbs_22230    avgbs_22231    avgbs_28366    avgbs_73048    avgbs_119495    avgbs_25432    avgbs_67844    avgbs_4472    avgbs_116069    avgbs_125237    avgbs_125281    avgbs_97822    avgbs_97823    avgbs_241017    avgbs_223974   
   6C 79 
 
 
  avgbs_28288    avgbs_116109    avgbs_116113    avgbs_46226    avgbs_11649    avgbs_11650    avgbs_225758   
   6C 80 
 
 
  avgbs_200377    avgbs_231745    avgbs_241281    avgbs_98336    avgbs_14244    avgbs_106649   
   6C 81 
 
 
  avgbs_95153    avgbs_56231   
   6C 83 
 
 
  avgbs_62893    avgbs_71957    avgbs_75258    avgbs_105578    avgbs_105579    avgbs_122788    avgbs_2437   
   6C 84 
  GMI_ES02_c4066_165    
 
  avgbs_97808    avgbs_222068    avgbs_123136    avgbs_223017    avgbs_8091    avgbs_24687    avgbs_30276    avgbs_43300    avgbs_110673    avgbs_120915    avgbs_217595    avgbs_217890    avgbs_205015    avgbs_16988    avgbs_37652    avgbs_88301    avgbs_85156    avgbs_99497    avgbs_205180    avgbs_18719    avgbs_75820    avgbs_203906    avgbs_231673    avgbs_231674    avgbs_58473    avgbs_75524    avgbs_40411    avgbs_28005    avgbs_82621    avgbs_103883    avgbs_111025    avgbs_82618    avgbs_21351    avgbs_21610    avgbs_82085    avgbs_82086    avgbs_235359    avgbs_68296    avgbs_3606    avgbs_3607    avgbs_100826    avgbs_10161    avgbs_119663    avgbs_82206    avgbs_112003    avgbs_102524    avgbs_113001    avgbs_81348    avgbs_64382   
   6C 85 
  GMI_ES01_c14065_159    
 
  avgbs_74929    avgbs_82920    avgbs_204596    avgbs_6732    avgbs_6733    avgbs_110674    avgbs_201603    avgbs_72560    avgbs_72561    avgbs_72562    avgbs_9211    avgbs_9212    avgbs_98063    avgbs_98064    avgbs_98065    avgbs_125490    avgbs_223136    avgbs_201    avgbs_6147    avgbs_6148    avgbs_49836    avgbs_49837    avgbs_7950    avgbs_7951    avgbs_235248    avgbs_235348    avgbs_241623   
   6C 87 
 
 
  avgbs_67553    avgbs_82287    avgbs_116795    avgbs_120433    avgbs_71237    avgbs_84425    avgbs_84426    avgbs_33930    avgbs_33931    avgbs_106884   
   6C 88 
  GMI_ES15_c858_507     GMI_ES01_c24478_209    
 
  avgbs_16954    avgbs_80993    avgbs_217219    avgbs_5020    avgbs_11564    avgbs_102720    avgbs_51796    avgbs_29616    avgbs_87565    avgbs_3608    avgbs_3610    avgbs_24160    avgbs_95517    avgbs_95518    avgbs_111663    avgbs_78724    avgbs_120010    avgbs_120011    avgbs_37066    avgbs_80994    avgbs_88569    avgbs_106570    avgbs_109541    avgbs_229157    avgbs_229835    avgbs_215790    avgbs_20943    avgbs_20944    avgbs_224917    avgbs_40395    avgbs_13506    avgbs_13507    avgbs_229203    avgbs_120866    avgbs_35368    avgbs_40137    avgbs_6K_24823    avgbs_102664    avgbs_102665    avgbs_9021    avgbs_9022    avgbs_123919    avgbs_208186    avgbs_213526    avgbs_5675    avgbs_46280    avgbs_79939    avgbs_59757    avgbs_210504    avgbs_18927    avgbs_94703   
   6C 89 
 
 
  avgbs_11769   
   6C 90 
 
 
  avgbs_124715    avgbs_92864   
   6C 91 
 
 
  avgbs_123487   
   6C 92 
 
 
  avgbs_93209    avgbs_21159    avgbs_87780    avgbs_201339    avgbs_11424    avgbs_81743   
   6C 93 
 
 
  avgbs_74088   
   6C 94 
 
 
  avgbs_98142    avgbs_49236   
   6C 95 
  GMI_ES15_c1697_192    
 
  avgbs_120526    avgbs_86209   
   6C 96 
 
 
  avgbs_222813    avgbs_92291    avgbs_124756    avgbs_86942   
   6C 97 
 
 
  avgbs_22059    avgbs_110420    avgbs_62647    avgbs_230646   
   6C 98 
 
 
  avgbs_87893    avgbs_87894   
   6C 99 
 
 
  avgbs_6100    avgbs_88788    avgbs_21370    avgbs_100905    avgbs_200191   
   6C 100 
 
 
  avgbs_6K_11246    avgbs_39893    avgbs_62001    avgbs_62785    avgbs_96746    avgbs_78780    avgbs_22174    avgbs_22175   
   6C 101 
 
 
  avgbs_62648    avgbs_71465    avgbs_103691    avgbs_33131   
   6C 102 
 
 
  avgbs_81744    avgbs_81745    avgbs_32850    avgbs_32851    avgbs_86154    avgbs_79184    avgbs_204875    avgbs_85883   
   6C 104 
 
 
  avgbs_32526    avgbs_223683    avgbs_76252    avgbs_107498    avgbs_107499    avgbs_107500    avgbs_7207   
 
 
   Chromosome 7C-17A    Chr   Pos   Framework   Placed SNP  Placed GBS  
   7C-17A -7 
 
 
  avgbs_36246    avgbs_121935   
   7C-17A -6 
 
 
  avgbs_30004    avgbs_30005    avgbs_30006   
   7C-17A -4 
 
 
  avgbs_20298   
   7C-17A -2 
 
 
  avgbs_98527   
   7C-17A -1 
 
 
  avgbs_111055    avgbs_101344    avgbs_222391    avgbs_34774    avgbs_10368    avgbs_79323    avgbs_90102    avgbs_204891    avgbs_206088   
   7C-17A 0 
  GMI_DS_CC5720_167    
 
  avgbs_53849    avgbs_54151    avgbs_121361    avgbs_121362    avgbs_203771    avgbs_205608    avgbs_33099    avgbs_33100    avgbs_84613    avgbs_84614    avgbs_38205    avgbs_38207    avgbs_51409    avgbs_75636    avgbs_80785    avgbs_102489    avgbs_213132    avgbs_229465    avgbs_204628    avgbs_85840    avgbs_47643    avgbs_66995    avgbs_111451    avgbs_111452    avgbs_210764    avgbs_206235    avgbs_111532    avgbs_241930    avgbs_81644    avgbs_6087    avgbs_6088    avgbs_112314    avgbs_112315    avgbs_62331    avgbs_62332    avgbs_29334    avgbs_35575    avgbs_38461    avgbs_111810    avgbs_111812    avgbs_14973    avgbs_103373    avgbs_82564    avgbs_203746    avgbs_102488    avgbs_204348    avgbs_206121    avgbs_203853    avgbs_14974    avgbs_42478    avgbs_52007    avgbs_54499    avgbs_206188    avgbs_3360    avgbs_29135    avgbs_85869    avgbs_42579    avgbs_206989    avgbs_10087    avgbs_200060   
   7C-17A 1 
 
 
  avgbs_86525    avgbs_205315    avgbs_35574   
   7C-17A 2 
 
 
  avgbs_5304    avgbs_217528   
   7C-17A 3 
 
 
  avgbs_66561   
   7C-17A 4 
 
 
  avgbs_219163    avgbs_114429    avgbs_109037    avgbs_109038    avgbs_122955   
   7C-17A 5 
 
 
  avgbs_980    avgbs_981    avgbs_11908    avgbs_27051    avgbs_221891    avgbs_91872   
   7C-17A 6 
 
 
  avgbs_82883    avgbs_42870    avgbs_27524    avgbs_231507    avgbs_25879    avgbs_87713    avgbs_102771    avgbs_229510    avgbs_200617    avgbs_67999    avgbs_87712    avgbs_232742   
   7C-17A 7 
 
 
  avgbs_229732   
   7C-17A 8 
 
 
  avgbs_87457    avgbs_220992    avgbs_18492    avgbs_34227    avgbs_218101    avgbs_112480    avgbs_213612   
   7C-17A 9 
 
 
  avgbs_39636    avgbs_43977    avgbs_6K_71500    avgbs_101665    avgbs_118253    avgbs_207594    avgbs_223097    avgbs_2231    avgbs_125892   
   7C-17A 10 
 
 
  avgbs_88496    avgbs_200622    avgbs_75266    avgbs_220283   
   7C-17A 11 
  GMI_ES17_c19709_452    
 
  avgbs_59478   
   7C-17A 12 
 
 
  avgbs_222063    avgbs_73357    avgbs_119667    avgbs_119668    avgbs_232614   
   7C-17A 13 
 
 
  avgbs_15825    avgbs_55947   
   7C-17A 14 
  GMI_ES01_lrc10014_150    
 
  avgbs_119080    avgbs_119081    avgbs_222803    avgbs_75267    avgbs_224771    avgbs_5781    avgbs_62712    avgbs_62714    avgbs_75268    avgbs_111296    avgbs_117095    avgbs_124562    avgbs_216842    avgbs_223885    avgbs_224464    avgbs_224592    avgbs_224924    avgbs_87022    avgbs_87023    avgbs_87024   
   7C-17A 15 
 
 
  avgbs_29974    avgbs_66152    avgbs_119900    avgbs_207731    avgbs_29620    avgbs_90592    avgbs_102066    avgbs_213249    avgbs_229404    avgbs_206086    avgbs_6267   
   7C-17A 16 
  GMI_ES17_c2767_643    
 
  avgbs_16811    avgbs_39046    avgbs_40319    avgbs_40320    avgbs_217211    avgbs_12593    avgbs_84329    avgbs_91753    avgbs_231651    avgbs_217149   
   7C-17A 17 
 
 
  avgbs_48992    avgbs_48993    avgbs_122870    avgbs_220400    avgbs_223003    avgbs_42560    avgbs_49511    avgbs_109469    avgbs_109470    avgbs_218634    avgbs_218823    avgbs_205726    avgbs_5270    avgbs_216496    avgbs_53468    avgbs_219007    avgbs_230943   
   7C-17A 18 
 
 
  avgbs_51556    avgbs_57625    avgbs_58476    avgbs_69443    avgbs_94785    avgbs_108818    avgbs_124046    avgbs_219310    avgbs_6942    avgbs_19754    avgbs_75168    avgbs_6K_78815    avgbs_99151    avgbs_214012    avgbs_102525    avgbs_7691    avgbs_28230    avgbs_223351    avgbs_223782    avgbs_224249    avgbs_225328   
   7C-17A 19 
 
 
  avgbs_12485    avgbs_41727    avgbs_41728    avgbs_49240    avgbs_49242    avgbs_59622    avgbs_12302    avgbs_57374    avgbs_76020    avgbs_9596    avgbs_71558    avgbs_86754    avgbs_94282    avgbs_122437    avgbs_224691   
   7C-17A 20 
 
 
  avgbs_7493    avgbs_7494    avgbs_401    avgbs_402    avgbs_403    avgbs_116318    avgbs_116450    avgbs_200259    avgbs_5246    avgbs_5247    avgbs_5248    avgbs_44387    avgbs_44388    avgbs_44389    avgbs_34288    avgbs_84460    avgbs_202970    avgbs_224639    avgbs_224881    avgbs_77275    avgbs_224816   
   7C-17A 21 
  GMI_ES17_c7160_500    
 
  avgbs_121351    avgbs_121352    avgbs_40539    avgbs_43039    avgbs_57694    avgbs_99761    avgbs_43007    avgbs_78546    avgbs_78547    avgbs_77959   
   7C-17A 22 
 
 
  avgbs_32956    avgbs_32957    avgbs_50328    avgbs_50329    avgbs_87714    avgbs_112818    avgbs_218017    avgbs_33898    avgbs_45918    avgbs_73063    avgbs_78566    avgbs_96749    avgbs_112203    avgbs_120745    avgbs_217338    avgbs_218074    avgbs_216047    avgbs_111794    avgbs_8657    avgbs_201540   
   7C-17A 23 
 
 
  avgbs_215705    avgbs_62507    avgbs_71188    avgbs_214545    avgbs_200471    avgbs_84738    avgbs_117004    avgbs_117005   
   7C-17A 24 
 
 
  avgbs_51590    avgbs_51591    avgbs_51592    avgbs_69446    avgbs_26642    avgbs_119347    avgbs_119348    avgbs_119349   
   7C-17A 25 
 
 
  avgbs_122755    avgbs_122756    avgbs_17320    avgbs_101357   
   7C-17A 26 
 
 
  avgbs_222311    avgbs_28703    avgbs_13296    avgbs_44054    avgbs_5314   
   7C-17A 28 
 
 
  avgbs_217320    avgbs_104495    avgbs_55655    avgbs_100619    avgbs_219138    avgbs_85299    avgbs_94387    avgbs_94388    avgbs_225008    avgbs_113541    avgbs_113542   
   7C-17A 29 
 
 
  avgbs_56617    avgbs_58397    avgbs_58399    avgbs_91551    avgbs_219201    avgbs_9108    avgbs_225187    avgbs_43038   
   7C-17A 30 
 
 
  avgbs_102067    avgbs_102068    avgbs_100941    avgbs_100942    avgbs_33845    avgbs_35472   
   7C-17A 31 
 
 
  avgbs_224776   
   7C-17A 33 
 
 
  avgbs_4487    avgbs_112671    avgbs_112672   
   7C-17A 34 
 
 
  avgbs_76959   
   7C-17A 35 
 
 
  avgbs_123565   
   7C-17A 38 
 
 
  avgbs_115800    avgbs_115801    avgbs_25923    avgbs_12860    avgbs_12861    avgbs_16956    avgbs_16957    avgbs_111847   
   7C-17A 39 
 
 
  avgbs_78563    avgbs_205321   
   7C-17A 40 
 
 
  avgbs_104787   
   7C-17A 41 
 
 
  avgbs_23988    avgbs_23989    avgbs_34247    avgbs_240185    avgbs_124931   
   7C-17A 42 
 
 
  avgbs_49064    avgbs_203533    avgbs_208807    avgbs_85035    avgbs_80983   
   7C-17A 43 
 
 
  avgbs_96153    avgbs_49680    avgbs_57219    avgbs_57220    avgbs_237804    avgbs_223883   
   7C-17A 44 
 
 
  avgbs_116101   
   7C-17A 45 
 
 
  avgbs_10609    avgbs_10610    avgbs_67384    avgbs_6K_75415    avgbs_230803    avgbs_60212    avgbs_34403    avgbs_225727    avgbs_205798   
   7C-17A 46 
 
 
  avgbs_220834    avgbs_67561    avgbs_36723    avgbs_36724    avgbs_36725    avgbs_72635    avgbs_117946    avgbs_232547    avgbs_234084    avgbs_242006    avgbs_36580    avgbs_200683   
   7C-17A 47 
 
 
  avgbs_32962    avgbs_232600    avgbs_4330    avgbs_228816    avgbs_20299    avgbs_124480    avgbs_237619    avgbs_113293   
   7C-17A 48 
  GMI_ES15_c349_532    
 
  avgbs_66989    avgbs_80042    avgbs_80043    avgbs_215273    avgbs_222014    avgbs_87237    avgbs_87238    avgbs_108951    avgbs_82126    avgbs_110281    avgbs_98695    avgbs_98696    avgbs_229420    avgbs_39862    avgbs_86580    avgbs_114438    avgbs_3237    avgbs_117207    avgbs_243423    avgbs_85484    avgbs_85485    avgbs_90171    avgbs_90172    avgbs_105852    avgbs_17802    avgbs_66954    avgbs_124669    avgbs_233298    avgbs_202182    avgbs_230793    avgbs_19554    avgbs_19555    avgbs_123178    avgbs_14024    avgbs_14025    avgbs_115916    avgbs_2132    avgbs_2133    avgbs_22934    avgbs_69779    avgbs_204352    avgbs_76627    avgbs_44227    avgbs_62500    avgbs_62501    avgbs_73994    avgbs_74253    avgbs_74254    avgbs_233166    avgbs_238543    avgbs_204544    avgbs_237671    avgbs_241144    avgbs_68001    avgbs_100967    avgbs_100968    avgbs_72041    avgbs_4561    avgbs_57436    avgbs_57437    avgbs_223338    avgbs_16085    avgbs_16086    avgbs_17004    avgbs_205119    avgbs_101974    avgbs_103318    avgbs_103319    avgbs_101694    avgbs_200738   
   7C-17A 49 
 
 
  avgbs_85342    avgbs_85343   
   7C-17A 50 
 
 
  avgbs_83704    avgbs_83705    avgbs_83706    avgbs_13303    avgbs_229115    avgbs_18450    avgbs_18451    avgbs_18452   
   7C-17A 51 
 
 
  avgbs_59380    avgbs_59384    avgbs_98121    avgbs_66240    avgbs_94749    avgbs_230772    avgbs_13658    avgbs_81672    avgbs_87141   
   7C-17A 52 
 
 
  avgbs_97900    avgbs_231291    avgbs_59848    avgbs_59849    avgbs_41574    avgbs_76360    avgbs_76361    avgbs_63628    avgbs_94281    avgbs_94346    avgbs_22223    avgbs_215533   
   7C-17A 53 
 
 
  avgbs_215479    avgbs_12138    avgbs_24475    avgbs_24476    avgbs_54105    avgbs_111719    avgbs_215806    avgbs_10106    avgbs_10107    avgbs_15800    avgbs_15801    avgbs_12885    avgbs_81442    avgbs_19725    avgbs_202365    avgbs_213566    avgbs_213732   
   7C-17A 54 
 
 
  avgbs_5612    avgbs_12738    avgbs_48056    avgbs_104232    avgbs_63755    avgbs_63757    avgbs_214578    avgbs_15216    avgbs_15217    avgbs_20055    avgbs_203784    avgbs_206659    avgbs_6K_65960    avgbs_213522   
   7C-17A 55 
 
  GMI_ES02_c29199_390   
  avgbs_2896    avgbs_71775    avgbs_216340    avgbs_18608    avgbs_53884    avgbs_200125    avgbs_110641    avgbs_231870    avgbs_2191    avgbs_2192    avgbs_19580    avgbs_29696    avgbs_36190    avgbs_36191    avgbs_38655    avgbs_113646    avgbs_94513    avgbs_240765    avgbs_47034    avgbs_124292    avgbs_215149    avgbs_18500    avgbs_18501   
   7C-17A 56 
 
 
  avgbs_216617    avgbs_220230    avgbs_116981    avgbs_7965    avgbs_89761    avgbs_231590    avgbs_91625    avgbs_20025    avgbs_24494    avgbs_58855    avgbs_58856    avgbs_58857    avgbs_62338    avgbs_87009    avgbs_231298    avgbs_9520    avgbs_31916    avgbs_51495    avgbs_230282    avgbs_229868    avgbs_81368    avgbs_7271    avgbs_16381    avgbs_81709    avgbs_114432    avgbs_114435    avgbs_207241    avgbs_38607    avgbs_39836    avgbs_113199    avgbs_113200    avgbs_17180    avgbs_86605    avgbs_124511    avgbs_90977    avgbs_40321    avgbs_40322    avgbs_1853    avgbs_30268    avgbs_18850    avgbs_30791    avgbs_202884   
   7C-17A 57 
 
 
  avgbs_95752    avgbs_215408    avgbs_58919    avgbs_222169    avgbs_221    avgbs_124846    avgbs_221250    avgbs_29078    avgbs_218093    avgbs_12718    avgbs_123530    avgbs_208149    avgbs_6K_41996    avgbs_59087    avgbs_230539    avgbs_23482    avgbs_55084    avgbs_55085    avgbs_50889    avgbs_114133    avgbs_117264    avgbs_207169    avgbs_207525    avgbs_75829    avgbs_238952    avgbs_207726    avgbs_77066    avgbs_106891    avgbs_213519    avgbs_11909    avgbs_42307    avgbs_42308    avgbs_44000    avgbs_44001    avgbs_14434    avgbs_30083    avgbs_225465    avgbs_11250    avgbs_93762    avgbs_107588    avgbs_107590    avgbs_88581    avgbs_104785    avgbs_79351   
   7C-17A 58 
  GMI_DS_CC3048_64    
 
  avgbs_6748    avgbs_109935    avgbs_3711    avgbs_103787    avgbs_94327    avgbs_219724    avgbs_215370    avgbs_29148    avgbs_89348    avgbs_6K_89190    avgbs_118889    avgbs_221113    avgbs_2985    avgbs_12753    avgbs_94756    avgbs_120067    avgbs_216968    avgbs_205759    avgbs_120842    avgbs_220251    avgbs_11631    avgbs_40133    avgbs_86799    avgbs_98005    avgbs_218338    avgbs_17763    avgbs_47896    avgbs_27711    avgbs_52082    avgbs_58965    avgbs_58967    avgbs_202142    avgbs_26166    avgbs_216023    avgbs_115987    avgbs_25033    avgbs_4501    avgbs_9497    avgbs_120814    avgbs_201228    avgbs_86475    avgbs_123327    avgbs_205308    avgbs_107654    avgbs_215699    avgbs_103101    avgbs_74736    avgbs_74737    avgbs_231062    avgbs_69892    avgbs_230    avgbs_40510    avgbs_38639    avgbs_62008    avgbs_66421    avgbs_91519    avgbs_104026    avgbs_202085    avgbs_72222    avgbs_239045    avgbs_70776    avgbs_17821    avgbs_60599    avgbs_82989    avgbs_103479    avgbs_121130    avgbs_123353    avgbs_214457    avgbs_242845    avgbs_2675    avgbs_20621    avgbs_20783    avgbs_34912    avgbs_34913    avgbs_34914    avgbs_52847    avgbs_85519    avgbs_89347    avgbs_108856    avgbs_114776    avgbs_114812    avgbs_213087    avgbs_202566    avgbs_203708    avgbs_34559    avgbs_40398    avgbs_126023    avgbs_20179    avgbs_82761    avgbs_233976    avgbs_234301    avgbs_28956    avgbs_47883    avgbs_94606    avgbs_80611    avgbs_80612    avgbs_125395    avgbs_71287    avgbs_208297    avgbs_2560    avgbs_79253    avgbs_123326    avgbs_23346    avgbs_23347    avgbs_54306    avgbs_78396    avgbs_10856    avgbs_52079    avgbs_241675    avgbs_41526    avgbs_2178    avgbs_62607    avgbs_94755    avgbs_237998    avgbs_103150    avgbs_67762    avgbs_117185    avgbs_223684    avgbs_2685    avgbs_122443    avgbs_21671    avgbs_98818    avgbs_98819    avgbs_120815    avgbs_122570    avgbs_115620    avgbs_224910    avgbs_38566    avgbs_21921    avgbs_21922    avgbs_37777    avgbs_205123    avgbs_45087    avgbs_13127    avgbs_16715    avgbs_201667    avgbs_10797    avgbs_65724    avgbs_213286    avgbs_202073    avgbs_213825    avgbs_204613    avgbs_53051    avgbs_65213    avgbs_201706    avgbs_213359    avgbs_214626    avgbs_205512    avgbs_205571    avgbs_215424    avgbs_215540    avgbs_23254    avgbs_39684    avgbs_74807    avgbs_116684    avgbs_207496    avgbs_1543    avgbs_1544    avgbs_53973    avgbs_59740    avgbs_214434    avgbs_6K_85932    avgbs_82789   
   7C-17A 59 
  GMI_ES17_c14163_394     GMI_ES02_c35031_175     GMI_ES01_c9241_407     GMI_ES17_c4498_222     GMI_ES01_lrc28828_382    
 
  avgbs_4932    avgbs_112135    avgbs_36253    avgbs_11215    avgbs_63569    avgbs_63570    avgbs_74306    avgbs_79580    avgbs_83807    avgbs_108620    avgbs_213297    avgbs_217434    avgbs_2233    avgbs_40385    avgbs_118369    avgbs_2167    avgbs_2168    avgbs_2169    avgbs_2476    avgbs_13284    avgbs_20858    avgbs_34798    avgbs_34799    avgbs_115614    avgbs_214056    avgbs_15808    avgbs_79574    avgbs_92060    avgbs_100334    avgbs_17570    avgbs_27712    avgbs_59163    avgbs_106889    avgbs_109891    avgbs_109892    avgbs_223558    avgbs_23345    avgbs_49232    avgbs_57922    avgbs_58077    avgbs_224379    avgbs_35700    avgbs_82606    avgbs_93601    avgbs_101797    avgbs_105919    avgbs_223781    avgbs_224941    avgbs_212875    avgbs_52081    avgbs_65552    avgbs_122320    avgbs_230750    avgbs_37502    avgbs_47745    avgbs_47746    avgbs_105878    avgbs_97340    avgbs_211940    avgbs_7268    avgbs_47603    avgbs_228915    avgbs_103473    avgbs_236113    avgbs_97103    avgbs_97104    avgbs_97105    avgbs_55477    avgbs_55478    avgbs_238523    avgbs_89828    avgbs_124374    avgbs_124375    avgbs_233128    avgbs_170    avgbs_117278    avgbs_117279    avgbs_125798    avgbs_208405    avgbs_77201    avgbs_71379    avgbs_214153    avgbs_25393    avgbs_66999    avgbs_16122    avgbs_16123    avgbs_206491    avgbs_58634    avgbs_73055    avgbs_206540    avgbs_55025    avgbs_92749    avgbs_57177    avgbs_94757    avgbs_15797    avgbs_15798    avgbs_72016    avgbs_4900    avgbs_4901    avgbs_12328    avgbs_33625    avgbs_33626    avgbs_42187    avgbs_47697    avgbs_70970    avgbs_121017    avgbs_203304    avgbs_6075    avgbs_60521    avgbs_77216    avgbs_106890    avgbs_117049    avgbs_121349    avgbs_124963    avgbs_201758    avgbs_99951    avgbs_20710    avgbs_64483    avgbs_82106    avgbs_101973    avgbs_106151    avgbs_110325    avgbs_200395    avgbs_214598    avgbs_206737    avgbs_207470    avgbs_26404    avgbs_54272    avgbs_43689    avgbs_230653    avgbs_16815    avgbs_46626    avgbs_57983    avgbs_5724    avgbs_82622    avgbs_82623    avgbs_82624    avgbs_71378    avgbs_204418    avgbs_6K_4425    avgbs_45403    avgbs_223279    avgbs_230084    avgbs_206926    avgbs_69223    avgbs_69224    avgbs_36988    avgbs_108411    avgbs_108412    avgbs_206836    avgbs_62506    avgbs_202758    avgbs_15960    avgbs_81120    avgbs_236805    avgbs_47333    avgbs_6354    avgbs_43108    avgbs_20881    avgbs_20882    avgbs_12575    avgbs_106172    avgbs_91681    avgbs_47609    avgbs_91322    avgbs_59021    avgbs_91397    avgbs_78936    avgbs_94824    avgbs_103085    avgbs_98817    avgbs_98820    avgbs_25459    avgbs_200248    avgbs_87002    avgbs_107433    avgbs_200613    avgbs_56137    avgbs_64836    avgbs_85245    avgbs_85246    avgbs_213974    avgbs_214616    avgbs_4822    avgbs_87018    avgbs_215267    avgbs_90941   
   7C-17A 60 
  GMI_DS_CC10034_60    
 
  avgbs_19766    avgbs_79089    avgbs_79090    avgbs_58128    avgbs_104786    avgbs_214401    avgbs_112552    avgbs_204828    avgbs_207062    avgbs_36684    avgbs_219281    avgbs_58113    avgbs_13295    avgbs_89853    avgbs_205076    avgbs_42778    avgbs_51026    avgbs_19235    avgbs_65039    avgbs_22168    avgbs_22574    avgbs_38916    avgbs_45413    avgbs_45414    avgbs_66784    avgbs_68841    avgbs_73056    avgbs_110173    avgbs_6K_102741    avgbs_200985    avgbs_205926    avgbs_10922    avgbs_38441    avgbs_46687    avgbs_46845    avgbs_71969    avgbs_100198    avgbs_125805    avgbs_92741    avgbs_244649    avgbs_29759    avgbs_69882    avgbs_89232    avgbs_53457    avgbs_52919    avgbs_42780    avgbs_8083    avgbs_8084    avgbs_22331    avgbs_31976    avgbs_51345    avgbs_55980    avgbs_202642    avgbs_209581    avgbs_214242    avgbs_30790    avgbs_56204    avgbs_83477   
   7C-17A 61 
  GMI_ES17_c4663_648     GMI_ES17_c6857_627     BA_grs_c10685_353    
 
  avgbs_16145    avgbs_21673    avgbs_42532    avgbs_45881    avgbs_67028    avgbs_84559    avgbs_213604    avgbs_213689    avgbs_202804    avgbs_214034    avgbs_3012    avgbs_8190    avgbs_17152    avgbs_29889    avgbs_30845    avgbs_36448    avgbs_41807    avgbs_49158    avgbs_52867    avgbs_53517    avgbs_71993    avgbs_74386    avgbs_74387    avgbs_83696    avgbs_100179    avgbs_102612    avgbs_108019    avgbs_113356    avgbs_117248    avgbs_120007    avgbs_213215    avgbs_202641    avgbs_203291    avgbs_203360    avgbs_214148    avgbs_214298    avgbs_214307    avgbs_214925    avgbs_222087    avgbs_215828    avgbs_215854    avgbs_31027    avgbs_33274    avgbs_33275    avgbs_33628    avgbs_33722    avgbs_45615    avgbs_45948    avgbs_51085    avgbs_56402    avgbs_66847    avgbs_69693    avgbs_73168    avgbs_78755    avgbs_78756    avgbs_92532    avgbs_100899    avgbs_111294    avgbs_122718    avgbs_202776    avgbs_213836    avgbs_214316    avgbs_204280    avgbs_214700    avgbs_215422    avgbs_216079    avgbs_208062    avgbs_20334    avgbs_56593    avgbs_79987    avgbs_214364    avgbs_222673    avgbs_6270    avgbs_6271    avgbs_19090    avgbs_23089    avgbs_40969    avgbs_21152    avgbs_47148    avgbs_122611    avgbs_208045    avgbs_22234    avgbs_65763    avgbs_68453    avgbs_104388    avgbs_213491    avgbs_50173    avgbs_50174    avgbs_51126    avgbs_62485    avgbs_72753    avgbs_113053    avgbs_122594    avgbs_214544    avgbs_215847    avgbs_216098    avgbs_61455    avgbs_72634    avgbs_214776    avgbs_77982    avgbs_204788    avgbs_59216    avgbs_88010    avgbs_222471    avgbs_50528    avgbs_50886    avgbs_65943    avgbs_72077    avgbs_74514    avgbs_220104    avgbs_214838    avgbs_8699    avgbs_118985    avgbs_118986    avgbs_205311    avgbs_84592    avgbs_74380    avgbs_236274    avgbs_27076    avgbs_23864    avgbs_114964    avgbs_103893    avgbs_19236    avgbs_58816    avgbs_41561    avgbs_41562    avgbs_112504    avgbs_29448    avgbs_123334    avgbs_123335    avgbs_204851    avgbs_241453    avgbs_207864    avgbs_37775    avgbs_41577    avgbs_59117    avgbs_5613    avgbs_8577    avgbs_9068    avgbs_37706    avgbs_38686    avgbs_61454    avgbs_69749    avgbs_76269    avgbs_99781    avgbs_110172    avgbs_112880    avgbs_114046    avgbs_121859    avgbs_216521    avgbs_239208    avgbs_207399    avgbs_25332    avgbs_35638    avgbs_78315    avgbs_6796    avgbs_19929    avgbs_19930    avgbs_38142    avgbs_50226    avgbs_59217    avgbs_59452    avgbs_60545    avgbs_66524    avgbs_71716    avgbs_77237    avgbs_78195    avgbs_103651    avgbs_109774    avgbs_112505    avgbs_121042    avgbs_121821    avgbs_122797    avgbs_126256    avgbs_202031    avgbs_241407    avgbs_206187    avgbs_244692    avgbs_1928    avgbs_6564    avgbs_35771    avgbs_49134    avgbs_79352    avgbs_79353    avgbs_201349    avgbs_202545    avgbs_213944    avgbs_237061    avgbs_6634    avgbs_18231    avgbs_20356    avgbs_23479    avgbs_25125    avgbs_28948    avgbs_32964    avgbs_36603    avgbs_39446    avgbs_43238    avgbs_47906    avgbs_47907    avgbs_51003    avgbs_51710    avgbs_58545    avgbs_58546    avgbs_78920    avgbs_91202    avgbs_97309    avgbs_105969    avgbs_110345    avgbs_111995    avgbs_113679    avgbs_113680    avgbs_126235    avgbs_126236    avgbs_201356    avgbs_202171    avgbs_213816    avgbs_214405    avgbs_239292    avgbs_215460    avgbs_35203    avgbs_42220    avgbs_74519    avgbs_74628    avgbs_76746    avgbs_88374    avgbs_112704    avgbs_120549    avgbs_203479    avgbs_215205    avgbs_655    avgbs_9957    avgbs_12010    avgbs_15483    avgbs_16998    avgbs_16999    avgbs_17312    avgbs_17313    avgbs_18819    avgbs_23969    avgbs_26485    avgbs_26923    avgbs_34422    avgbs_36894    avgbs_37255    avgbs_39632    avgbs_39778    avgbs_45193    avgbs_48446    avgbs_53352    avgbs_53995    avgbs_61541    avgbs_62172    avgbs_69490    avgbs_69491    avgbs_69820    avgbs_71555    avgbs_90586    avgbs_109223    avgbs_112227    avgbs_115570    avgbs_122207    avgbs_125956    avgbs_201565    avgbs_234258    avgbs_202290    avgbs_213667    avgbs_203409    avgbs_203465    avgbs_203625    avgbs_204575    avgbs_205561    avgbs_7005    avgbs_21937    avgbs_36421    avgbs_63881    avgbs_88390    avgbs_93889    avgbs_111745    avgbs_126134    avgbs_202607    avgbs_203918    avgbs_238256    avgbs_99437    avgbs_14385    avgbs_14386    avgbs_14387    avgbs_26717    avgbs_27824    avgbs_33684    avgbs_39108    avgbs_39419    avgbs_39420    avgbs_46844    avgbs_66640    avgbs_68843    avgbs_69631    avgbs_77202    avgbs_78698    avgbs_81393    avgbs_110068    avgbs_113806    avgbs_238330    avgbs_205690    avgbs_206766    avgbs_2284    avgbs_6889    avgbs_29025    avgbs_29089    avgbs_39274    avgbs_39275    avgbs_65489    avgbs_103152    avgbs_103154    avgbs_38606    avgbs_41573    avgbs_46031    avgbs_63800    avgbs_77461    avgbs_80008    avgbs_80936    avgbs_93045    avgbs_101191    avgbs_120179    avgbs_120180    avgbs_123195    avgbs_123196    avgbs_234990    avgbs_236474    avgbs_214697    avgbs_239096    avgbs_241951    avgbs_13453    avgbs_25688    avgbs_35339    avgbs_101361    avgbs_125979    avgbs_203342    avgbs_6306    avgbs_24379    avgbs_45517    avgbs_80349    avgbs_218587    avgbs_206570    avgbs_35875    avgbs_206995    avgbs_8324    avgbs_8325    avgbs_65973    avgbs_70412    avgbs_40379    avgbs_47904    avgbs_47905    avgbs_97127    avgbs_103457    avgbs_47710    avgbs_87067    avgbs_41578    avgbs_49264    avgbs_103907    avgbs_214190    avgbs_31007    avgbs_66381    avgbs_58765    avgbs_101093    avgbs_237724    avgbs_22449    avgbs_39961    avgbs_69594    avgbs_214696    avgbs_204798    avgbs_40989    avgbs_81080    avgbs_118030    avgbs_239227    avgbs_240592    avgbs_240601    avgbs_60403    avgbs_25555    avgbs_60402    avgbs_64238    avgbs_71879    avgbs_71880    avgbs_75674    avgbs_94678    avgbs_3173    avgbs_76146    avgbs_106130    avgbs_237357    avgbs_96245    avgbs_232832    avgbs_32948    avgbs_119991    avgbs_234585    avgbs_85239    avgbs_85240    avgbs_103212    avgbs_237240    avgbs_57143    avgbs_64223    avgbs_64224    avgbs_120566    avgbs_243764    avgbs_35628    avgbs_64129    avgbs_236457    avgbs_237301    avgbs_204041    avgbs_35933    avgbs_203277    avgbs_113308    avgbs_4876    avgbs_5850    avgbs_25541    avgbs_68310    avgbs_49563    avgbs_37776    avgbs_223955    avgbs_225638    avgbs_6K_74175    avgbs_224885    avgbs_68300    avgbs_6910    avgbs_15094    avgbs_15095    avgbs_15096    avgbs_15097    avgbs_45667    avgbs_45668    avgbs_6287    avgbs_79629    avgbs_224872    avgbs_2461    avgbs_2858    avgbs_2859    avgbs_2908    avgbs_2909    avgbs_3063    avgbs_3958    avgbs_5603    avgbs_5604    avgbs_8191    avgbs_8262    avgbs_8660    avgbs_8811    avgbs_8812    avgbs_9407    avgbs_9609    avgbs_11151    avgbs_11449    avgbs_11714    avgbs_11715    avgbs_14837    avgbs_15489    avgbs_15653    avgbs_6K_14791    avgbs_21207    avgbs_6K_19541    avgbs_21235    avgbs_22530    avgbs_22531    avgbs_28367    avgbs_30111    avgbs_30234    avgbs_30235    avgbs_31951    avgbs_32008    avgbs_34292    avgbs_35598    avgbs_35767    avgbs_35768    avgbs_35769    avgbs_35930    avgbs_36457    avgbs_36458    avgbs_36459    avgbs_37249    avgbs_39174    avgbs_39175    avgbs_45005    avgbs_47099    avgbs_52861    avgbs_52862    avgbs_52872    avgbs_53444    avgbs_54421    avgbs_54940    avgbs_54941    avgbs_55996    avgbs_60991    avgbs_60992    avgbs_62191    avgbs_62192    avgbs_67587    avgbs_68471    avgbs_70314    avgbs_75033    avgbs_75798    avgbs_76789    avgbs_77935    avgbs_80957    avgbs_80958    avgbs_81572    avgbs_83466    avgbs_84508    avgbs_84509    avgbs_87143    avgbs_91011    avgbs_92513    avgbs_93210    avgbs_93698    avgbs_94754    avgbs_95751    avgbs_97642    avgbs_99511    avgbs_100406    avgbs_100447    avgbs_101252    avgbs_103934    avgbs_104150    avgbs_104751    avgbs_104792    avgbs_104793    avgbs_106524    avgbs_106911    avgbs_106914    avgbs_113355    avgbs_113766    avgbs_113828    avgbs_117057    avgbs_118867    avgbs_119147    avgbs_119671    avgbs_120609    avgbs_121874    avgbs_122269    avgbs_223188    avgbs_223258    avgbs_223378    avgbs_223390    avgbs_223395    avgbs_213348    avgbs_223542    avgbs_223600    avgbs_223602    avgbs_223631    avgbs_223672    avgbs_202711    avgbs_223712    avgbs_223719    avgbs_223743    avgbs_223828    avgbs_223985    avgbs_203404    avgbs_224126    avgbs_203464    avgbs_224177    avgbs_203567    avgbs_224192    avgbs_224303    avgbs_224650    avgbs_224779    avgbs_224792    avgbs_214947    avgbs_224870    avgbs_224951    avgbs_225011    avgbs_225049    avgbs_225052    avgbs_225090    avgbs_225132    avgbs_225178    avgbs_225216    avgbs_225333    avgbs_225444    avgbs_225581    avgbs_225759    avgbs_6292    avgbs_13033    avgbs_13034    avgbs_15892    avgbs_16797    avgbs_16798    avgbs_16799    avgbs_29019    avgbs_32181    avgbs_32182    avgbs_32963    avgbs_33963    avgbs_33964    avgbs_35927    avgbs_35937    avgbs_35938    avgbs_36257    avgbs_36258    avgbs_36420    avgbs_36632    avgbs_36658    avgbs_36732    avgbs_37799    avgbs_44671    avgbs_47865    avgbs_50883    avgbs_58308    avgbs_66126    avgbs_68016    avgbs_68787    avgbs_69753    avgbs_70533    avgbs_70534    avgbs_72071    avgbs_76457    avgbs_77434    avgbs_78144    avgbs_98936    avgbs_99291    avgbs_99292    avgbs_102214    avgbs_102215    avgbs_103357    avgbs_104655    avgbs_107589    avgbs_112532    avgbs_112533    avgbs_112534    avgbs_114489    avgbs_115081    avgbs_122833    avgbs_125911    avgbs_223430    avgbs_223688    avgbs_223936    avgbs_224080    avgbs_224618    avgbs_224701    avgbs_224817    avgbs_224848    avgbs_207123    avgbs_1452    avgbs_2326    avgbs_15832    avgbs_45921    avgbs_224106    avgbs_4090    avgbs_14608    avgbs_24018    avgbs_26103    avgbs_34421    avgbs_36193    avgbs_36657    avgbs_36881    avgbs_40864    avgbs_40865    avgbs_44640    avgbs_54436    avgbs_60203    avgbs_61404    avgbs_61405    avgbs_67941    avgbs_67942    avgbs_72445    avgbs_72752    avgbs_78777    avgbs_78904    avgbs_95602    avgbs_95604    avgbs_95609    avgbs_101321    avgbs_101322    avgbs_223685    avgbs_223813    avgbs_223930    avgbs_36401    avgbs_223934    avgbs_225237    avgbs_225704    avgbs_9920    avgbs_68844    avgbs_99465    avgbs_101025    avgbs_102614    avgbs_106519    avgbs_106520    avgbs_112948    avgbs_223403    avgbs_5279    avgbs_29077    avgbs_39933    avgbs_39970    avgbs_43489    avgbs_45576    avgbs_45979    avgbs_45980    avgbs_47992    avgbs_52759    avgbs_63121    avgbs_106020    avgbs_109457    avgbs_109458    avgbs_110127    avgbs_119029    avgbs_223268    avgbs_223870    avgbs_6600    avgbs_72712    avgbs_97512    avgbs_26807    avgbs_27449    avgbs_36587    avgbs_37468    avgbs_37469    avgbs_37470    avgbs_37910    avgbs_47459    avgbs_47460    avgbs_47461    avgbs_47462    avgbs_70793    avgbs_77404    avgbs_77405    avgbs_82952    avgbs_95405    avgbs_108430    avgbs_213673    avgbs_225121    avgbs_205772    avgbs_225495    avgbs_114243    avgbs_93555    avgbs_215347    avgbs_94647    avgbs_215207    avgbs_215952    avgbs_27487    avgbs_46686    avgbs_51722    avgbs_61111    avgbs_83570    avgbs_83571    avgbs_213687    avgbs_2836    avgbs_3593    avgbs_5784    avgbs_10064    avgbs_15490    avgbs_23651    avgbs_35770    avgbs_51475    avgbs_56897    avgbs_74917    avgbs_84097    avgbs_97024    avgbs_100914    avgbs_109689    avgbs_213161    avgbs_201646    avgbs_215147    avgbs_215308    avgbs_215448    avgbs_1567    avgbs_19520    avgbs_19521    avgbs_77236    avgbs_79093    avgbs_86561    avgbs_90619    avgbs_213165    avgbs_214956    avgbs_205562    avgbs_9907    avgbs_9908    avgbs_9909    avgbs_48884    avgbs_214178    avgbs_11937    avgbs_11938    avgbs_22165    avgbs_22166    avgbs_103351    avgbs_103352    avgbs_202611    avgbs_202783   
   7C-17A 62 
  GMI_ES17_c18155_185     GMI_ES17_c3807_825     GMI_ES15_c12378_444    
  GMI_ES15_c13434_347    GMI_ES01_c8527_320   
  avgbs_85294    avgbs_220871    avgbs_57466    avgbs_2094    avgbs_6297    avgbs_14934    avgbs_19234    avgbs_6K_43789    avgbs_59375    avgbs_59971    avgbs_59972    avgbs_60813    avgbs_65336    avgbs_65337    avgbs_66136    avgbs_66138    avgbs_75167    avgbs_77189    avgbs_80494    avgbs_80802    avgbs_117906    avgbs_120064    avgbs_218235    avgbs_214465    avgbs_219591    avgbs_219664    avgbs_219913    avgbs_204330    avgbs_219991    avgbs_220278    avgbs_221850    avgbs_221867    avgbs_222854    avgbs_7146    avgbs_9785    avgbs_9786    avgbs_13475    avgbs_14263    avgbs_38943    avgbs_44815    avgbs_47100    avgbs_50573    avgbs_65474    avgbs_65475    avgbs_92001    avgbs_95603    avgbs_105231    avgbs_105447    avgbs_105448    avgbs_105449    avgbs_105704    avgbs_201058    avgbs_217226    avgbs_214079    avgbs_218692    avgbs_218870    avgbs_219625    avgbs_204195    avgbs_125579    avgbs_53553    avgbs_80297    avgbs_60867    avgbs_28906    avgbs_33472    avgbs_40972    avgbs_45876    avgbs_46022    avgbs_80848    avgbs_88344    avgbs_88351    avgbs_200233    avgbs_200565    avgbs_221078    avgbs_3131    avgbs_3132    avgbs_44638    avgbs_45465    avgbs_50046    avgbs_55581    avgbs_55582    avgbs_73175    avgbs_96127    avgbs_109365    avgbs_16469    avgbs_57310    avgbs_84095    avgbs_124440    avgbs_216145    avgbs_5801    avgbs_5802    avgbs_5803    avgbs_7844    avgbs_18846    avgbs_53718    avgbs_82353    avgbs_125642    avgbs_202294    avgbs_220796    avgbs_18808    avgbs_53503    avgbs_72188    avgbs_109297    avgbs_109299    avgbs_206684    avgbs_24056    avgbs_32662    avgbs_33937    avgbs_33938    avgbs_56473    avgbs_74756    avgbs_124073    avgbs_124368    avgbs_218664    avgbs_44711    avgbs_237929    avgbs_8213    avgbs_96185    avgbs_96186    avgbs_27332    avgbs_223747    avgbs_11007    avgbs_11008    avgbs_22916    avgbs_89814    avgbs_58555    avgbs_223983    avgbs_107024    avgbs_225489    avgbs_25244    avgbs_66647    avgbs_93126    avgbs_2875    avgbs_4831    avgbs_6631    avgbs_6795    avgbs_7838    avgbs_7869    avgbs_9302    avgbs_9303    avgbs_9339    avgbs_10335    avgbs_10738    avgbs_10790    avgbs_10923    avgbs_11014    avgbs_11127    avgbs_11148    avgbs_11149    avgbs_11150    avgbs_12921    avgbs_13057    avgbs_13381    avgbs_13382    avgbs_13683    avgbs_14544    avgbs_14749    avgbs_14821    avgbs_15488    avgbs_19209    avgbs_19781    avgbs_19782    avgbs_21136    avgbs_23327    avgbs_23328    avgbs_23491    avgbs_29209    avgbs_31580    avgbs_36880    avgbs_36887    avgbs_39682    avgbs_40650    avgbs_40651    avgbs_41320    avgbs_41982    avgbs_41983    avgbs_42950    avgbs_42974    avgbs_43071    avgbs_43321    avgbs_43330    avgbs_48289    avgbs_48695    avgbs_51325    avgbs_51326    avgbs_51522    avgbs_51523    avgbs_52554    avgbs_59158    avgbs_59974    avgbs_61244    avgbs_62516    avgbs_63880    avgbs_64485    avgbs_64486    avgbs_64745    avgbs_65726    avgbs_65749    avgbs_65864    avgbs_66422    avgbs_67435    avgbs_68560    avgbs_68728    avgbs_69824    avgbs_69825    avgbs_70313    avgbs_70593    avgbs_70594    avgbs_73577    avgbs_73877    avgbs_73885    avgbs_76836    avgbs_77390    avgbs_79084    avgbs_79085    avgbs_79086    avgbs_79821    avgbs_80306    avgbs_80307    avgbs_83118    avgbs_83119    avgbs_83315    avgbs_84369    avgbs_86881    avgbs_91108    avgbs_93697    avgbs_97757    avgbs_98143    avgbs_98720    avgbs_98947    avgbs_98948    avgbs_99969    avgbs_99970    avgbs_100096    avgbs_100503    avgbs_101107    avgbs_101319    avgbs_101320    avgbs_101740    avgbs_103160    avgbs_103414    avgbs_104340    avgbs_106014    avgbs_106370    avgbs_106607    avgbs_109364    avgbs_110168    avgbs_110849    avgbs_110952    avgbs_113110    avgbs_115605    avgbs_117835    avgbs_118489    avgbs_118662    avgbs_119051    avgbs_119539    avgbs_119540    avgbs_119541    avgbs_119619    avgbs_119896    avgbs_122026    avgbs_125117    avgbs_125519    avgbs_223182    avgbs_223252    avgbs_223278    avgbs_223280    avgbs_223299    avgbs_223316    avgbs_223324    avgbs_223375    avgbs_223387    avgbs_223405    avgbs_223414    avgbs_223415    avgbs_223416    avgbs_223495    avgbs_223576    avgbs_223595    avgbs_223652    avgbs_223718    avgbs_223720    avgbs_223748    avgbs_213744    avgbs_223841    avgbs_223880    avgbs_224019    avgbs_224022    avgbs_224050    avgbs_224058    avgbs_224095    avgbs_224127    avgbs_224151    avgbs_224222    avgbs_224258    avgbs_224269    avgbs_224293    avgbs_224334    avgbs_224429    avgbs_224440    avgbs_224456    avgbs_224511    avgbs_224527    avgbs_224561    avgbs_224579    avgbs_224581    avgbs_224601    avgbs_224612    avgbs_224617    avgbs_224622    avgbs_224646    avgbs_224682    avgbs_224684    avgbs_224719    avgbs_224736    avgbs_224754    avgbs_224772    avgbs_224806    avgbs_224823    avgbs_224900    avgbs_224909    avgbs_224947    avgbs_224952    avgbs_224959    avgbs_224961    avgbs_224968    avgbs_224969    avgbs_224971    avgbs_224993    avgbs_225024    avgbs_205416    avgbs_225263    avgbs_225282    avgbs_225366    avgbs_225395    avgbs_225542    avgbs_225665    avgbs_225670    avgbs_225676    avgbs_225692    avgbs_225705    avgbs_225713    avgbs_225732    avgbs_225756    avgbs_225843    avgbs_225853    avgbs_225857    avgbs_6K_5298    avgbs_96275    avgbs_9415    avgbs_10036    avgbs_13471    avgbs_16325    avgbs_30494    avgbs_39935    avgbs_39936    avgbs_45105    avgbs_46985    avgbs_46986    avgbs_48702    avgbs_48703    avgbs_59181    avgbs_62100    avgbs_62101    avgbs_65268    avgbs_76338    avgbs_85105    avgbs_92530    avgbs_92531    avgbs_104351    avgbs_107198    avgbs_115219    avgbs_115220    avgbs_115221    avgbs_119584    avgbs_126266    avgbs_223236    avgbs_223404    avgbs_223472    avgbs_223823    avgbs_223846    avgbs_224124    avgbs_224125    avgbs_224793    avgbs_225021    avgbs_225176    avgbs_225418    avgbs_225856    avgbs_80797    avgbs_112134    avgbs_224901    avgbs_225560    avgbs_7471    avgbs_10090    avgbs_14031    avgbs_18099    avgbs_22571    avgbs_33562    avgbs_34697    avgbs_36163    avgbs_36164    avgbs_36861    avgbs_36862    avgbs_36863    avgbs_41464    avgbs_41465    avgbs_41486    avgbs_51643    avgbs_52013    avgbs_72669    avgbs_75932    avgbs_76213    avgbs_90267    avgbs_95497    avgbs_95498    avgbs_95529    avgbs_95530    avgbs_96984    avgbs_107924    avgbs_114134    avgbs_117798    avgbs_223689    avgbs_224191    avgbs_214250    avgbs_225591    avgbs_225674    avgbs_77235    avgbs_19899    avgbs_27924    avgbs_27925    avgbs_28059    avgbs_28060    avgbs_61826    avgbs_67976    avgbs_224065    avgbs_225679    avgbs_29409    avgbs_224455    avgbs_117217    avgbs_117218    avgbs_6903    avgbs_6K_4542    avgbs_71257   
   7C-17A 63 
 
 
  avgbs_79562    avgbs_221247    avgbs_72754    avgbs_14740    avgbs_50045    avgbs_1121    avgbs_73148    avgbs_47773    avgbs_125667    avgbs_32665    avgbs_32666    avgbs_48542    avgbs_48543    avgbs_68688    avgbs_39436    avgbs_237173    avgbs_215965    avgbs_53508    avgbs_79563   
   7C-17A 64 
  GMI_ES_CC7307_489     GMI_ES02_c18621_282     GMI_ES_CC7849_819    
 
  avgbs_40189    avgbs_80662    avgbs_80663    avgbs_220617    avgbs_43084    avgbs_90131    avgbs_90150    avgbs_92793    avgbs_214255    avgbs_221158    avgbs_56485    avgbs_7484    avgbs_14398    avgbs_17994    avgbs_18990    avgbs_19699    avgbs_67589    avgbs_73171    avgbs_74742    avgbs_79411    avgbs_115090    avgbs_124298    avgbs_216905    avgbs_204267    avgbs_19218    avgbs_29345    avgbs_51951    avgbs_71434    avgbs_78049    avgbs_112124    avgbs_112125    avgbs_119732    avgbs_204790    avgbs_91052    avgbs_9923    avgbs_21590    avgbs_26333    avgbs_28263    avgbs_61544    avgbs_83284    avgbs_119675    avgbs_122993    avgbs_71347    avgbs_84488    avgbs_5813    avgbs_5814    avgbs_61539    avgbs_86594    avgbs_216882    avgbs_61543    avgbs_221299    avgbs_2397    avgbs_22324    avgbs_54042    avgbs_230357    avgbs_97812    avgbs_231906    avgbs_96259    avgbs_204136    avgbs_60792    avgbs_89656    avgbs_89657    avgbs_224454    avgbs_73170    avgbs_73216    avgbs_73217    avgbs_46989    avgbs_47743    avgbs_122937    avgbs_19219    avgbs_73815    avgbs_116969    avgbs_116970    avgbs_116971    avgbs_119511    avgbs_35705    avgbs_36187    avgbs_64117    avgbs_107617    avgbs_107618    avgbs_108784    avgbs_112116    avgbs_124398    avgbs_202500    avgbs_65002    avgbs_204154    avgbs_27407    avgbs_33163    avgbs_33164    avgbs_36396    avgbs_51839    avgbs_55496    avgbs_73806    avgbs_74123    avgbs_74158    avgbs_109786    avgbs_115085    avgbs_124406    avgbs_236287    avgbs_205233    avgbs_47516    avgbs_47517    avgbs_102674    avgbs_106905    avgbs_207882    avgbs_15728    avgbs_22029    avgbs_27560    avgbs_29456    avgbs_36922    avgbs_52609    avgbs_72622    avgbs_90593    avgbs_101573    avgbs_105954    avgbs_124284    avgbs_209597    avgbs_224714    avgbs_216108    avgbs_235747    avgbs_214915    avgbs_40945    avgbs_92068    avgbs_111124    avgbs_111511    avgbs_215297    avgbs_244491    avgbs_79699    avgbs_211189    avgbs_243730    avgbs_1658    avgbs_3470    avgbs_18460    avgbs_18461    avgbs_20734    avgbs_20735    avgbs_29470    avgbs_236517    avgbs_244647    avgbs_54000    avgbs_233099    avgbs_22538    avgbs_75905    avgbs_108058    avgbs_124839    avgbs_220332    avgbs_5500    avgbs_20277    avgbs_20278    avgbs_24585    avgbs_238897    avgbs_42559    avgbs_73180    avgbs_224838    avgbs_79956    avgbs_96821    avgbs_103278    avgbs_10134    avgbs_70197    avgbs_238546    avgbs_36053    avgbs_36054    avgbs_49294    avgbs_87276    avgbs_235326    avgbs_206489    avgbs_79752    avgbs_239319    avgbs_103151    avgbs_206152    avgbs_47428    avgbs_34372    avgbs_34373    avgbs_34374    avgbs_34375    avgbs_4652    avgbs_9795    avgbs_29013    avgbs_48950    avgbs_48951    avgbs_59114    avgbs_84357    avgbs_223400    avgbs_223890    avgbs_224990    avgbs_4993    avgbs_7485    avgbs_12031    avgbs_6K_13559    avgbs_16236    avgbs_17114    avgbs_29344    avgbs_43621    avgbs_51752    avgbs_59145    avgbs_59146    avgbs_60172    avgbs_60220    avgbs_62525    avgbs_87118    avgbs_87335    avgbs_87336    avgbs_99496    avgbs_102673    avgbs_106350    avgbs_110570    avgbs_110571    avgbs_117374    avgbs_6K_114344    avgbs_125418    avgbs_125748    avgbs_223459    avgbs_223545    avgbs_223829    avgbs_224089    avgbs_224746    avgbs_225034    avgbs_225048    avgbs_225123    avgbs_225312    avgbs_215652    avgbs_225458    avgbs_225618    avgbs_225689    avgbs_225716    avgbs_225818    avgbs_60712    avgbs_21221    avgbs_85723    avgbs_103994    avgbs_105292    avgbs_6396    avgbs_20537    avgbs_20538    avgbs_72472    avgbs_26823    avgbs_97055    avgbs_106351    avgbs_38653    avgbs_43085    avgbs_225099    avgbs_65809    avgbs_214641    avgbs_47143   
   7C-17A 65 
  GMI_ES02_c4957_300     GMI_ES_CC6185_80    
 
  avgbs_205149    avgbs_74389    avgbs_216161    avgbs_115804    avgbs_67914    avgbs_38237    avgbs_202899    avgbs_65702    avgbs_237992    avgbs_61476    avgbs_235681    avgbs_5139    avgbs_91524    avgbs_46112    avgbs_98798    avgbs_98799    avgbs_1018    avgbs_18994    avgbs_213254    avgbs_18739    avgbs_18740    avgbs_74388    avgbs_201346    avgbs_19804    avgbs_124028    avgbs_228003    avgbs_57829    avgbs_74993    avgbs_74994    avgbs_119759    avgbs_4089    avgbs_201194    avgbs_71329    avgbs_5830    avgbs_223311    avgbs_59068    avgbs_59069    avgbs_225128    avgbs_225377    avgbs_65379   
   7C-17A 66 
 
 
  avgbs_69284    avgbs_69285    avgbs_206068    avgbs_105132    avgbs_222095    avgbs_32301    avgbs_217935    avgbs_104801    avgbs_16386    avgbs_16387    avgbs_100901    avgbs_34850    avgbs_3846    avgbs_52204    avgbs_52205    avgbs_235265    avgbs_123072    avgbs_224681    avgbs_98094    avgbs_225296   
   7C-17A 67 
  GMI_ES_CC2088_506    
 
  avgbs_217697    avgbs_65725    avgbs_221983    avgbs_111914    avgbs_21153    avgbs_36683    avgbs_19949    avgbs_213520    avgbs_29886    avgbs_76175    avgbs_218346    avgbs_220429    avgbs_71500    avgbs_86032    avgbs_115131    avgbs_5619    avgbs_91752    avgbs_124397    avgbs_231011    avgbs_69627    avgbs_233213    avgbs_3894    avgbs_114233    avgbs_47341    avgbs_47342    avgbs_230150    avgbs_32638    avgbs_114616    avgbs_230808    avgbs_96228    avgbs_37572    avgbs_10231    avgbs_240070    avgbs_242257    avgbs_201157    avgbs_81512    avgbs_220658    avgbs_17284    avgbs_75845    avgbs_96192    avgbs_29998    avgbs_12263    avgbs_77327    avgbs_223691    avgbs_122517   
   7C-17A 68 
  GMI_ES15_c17005_479    
 
  avgbs_7581    avgbs_18867    avgbs_200127    avgbs_69890    avgbs_105773    avgbs_125191    avgbs_200237    avgbs_218324    avgbs_9475    avgbs_34296    avgbs_50157    avgbs_81570    avgbs_99613    avgbs_99614    avgbs_112526    avgbs_216758    avgbs_12497    avgbs_14104    avgbs_25139    avgbs_55336    avgbs_75640    avgbs_98092    avgbs_217617    avgbs_221719    avgbs_27465    avgbs_27772    avgbs_41440    avgbs_57555    avgbs_113929    avgbs_209931    avgbs_205570    avgbs_222719    avgbs_2022    avgbs_113756    avgbs_218865    avgbs_21825    avgbs_44862    avgbs_50496    avgbs_93225    avgbs_108458    avgbs_217466    avgbs_221331    avgbs_53354    avgbs_95965    avgbs_221560    avgbs_120879    avgbs_222896    avgbs_45613    avgbs_230090    avgbs_25826    avgbs_66321    avgbs_72229    avgbs_92647    avgbs_20110    avgbs_223614    avgbs_76679    avgbs_68271    avgbs_68272    avgbs_97116    avgbs_124853    avgbs_8750    avgbs_222964    avgbs_16028    avgbs_112384    avgbs_112021    avgbs_6K_103591    avgbs_231697    avgbs_61226    avgbs_230602    avgbs_230746    avgbs_80232    avgbs_231113    avgbs_243004    avgbs_70903    avgbs_230931    avgbs_3268    avgbs_14018    avgbs_61537    avgbs_29477    avgbs_101898    avgbs_19231    avgbs_29912    avgbs_77014    avgbs_223591    avgbs_224359    avgbs_6230    avgbs_13228    avgbs_26366    avgbs_123040    avgbs_223318    avgbs_225795    avgbs_966    avgbs_1013    avgbs_223725    avgbs_223831    avgbs_98832    avgbs_98833    avgbs_109890   
   7C-17A 69 
 
 
  avgbs_216261    avgbs_216943    avgbs_24967    avgbs_4091    avgbs_89837    avgbs_217202    avgbs_214303    avgbs_39465    avgbs_229068    avgbs_12586    avgbs_12587    avgbs_91630    avgbs_97000    avgbs_228951    avgbs_231422    avgbs_21881    avgbs_92131    avgbs_97454    avgbs_116947    avgbs_229399    avgbs_213761    avgbs_44704    avgbs_122190    avgbs_25163    avgbs_202646    avgbs_47225    avgbs_9129    avgbs_86379    avgbs_31200    avgbs_87003    avgbs_223834    avgbs_224871    avgbs_10388    avgbs_39706    avgbs_55604    avgbs_65551    avgbs_6K_69480    avgbs_76550    avgbs_90751    avgbs_94354    avgbs_103179    avgbs_120777    avgbs_223253    avgbs_223527    avgbs_223551    avgbs_223989    avgbs_224948    avgbs_225051    avgbs_225433    avgbs_225738    avgbs_36677    avgbs_68026    avgbs_224606    avgbs_225277    avgbs_22561    avgbs_95996    avgbs_98704    avgbs_225246    avgbs_225720    avgbs_16312    avgbs_16313    avgbs_105768   
   7C-17A 70 
  GMI_DS_CC7846_153     BA_grs_c6565_175    
 
  avgbs_10570    avgbs_216823    avgbs_30284    avgbs_113854    avgbs_113855    avgbs_218243    avgbs_27410    avgbs_218191    avgbs_89492    avgbs_59215    avgbs_49128    avgbs_10560    avgbs_80175    avgbs_120530    avgbs_120531    avgbs_223644    avgbs_7650    avgbs_17617    avgbs_21144    avgbs_21145    avgbs_22887    avgbs_31018    avgbs_47785    avgbs_49852    avgbs_51220    avgbs_126263    avgbs_226002    avgbs_230164    avgbs_231731    avgbs_232102    avgbs_44039    avgbs_45872    avgbs_67309    avgbs_96541    avgbs_100123    avgbs_118128    avgbs_118129    avgbs_118130    avgbs_118131    avgbs_121799    avgbs_121800    avgbs_125002    avgbs_83136    avgbs_92132    avgbs_108963    avgbs_108964    avgbs_15836    avgbs_33357    avgbs_232752    avgbs_37301    avgbs_68073    avgbs_696    avgbs_707   
   7C-17A 71 
  GMI_ES15_c6979_338     GMI_ES01_c10107_481     GMI_ES01_lrc14087_138     GMI_ES15_c6193_143    
 
  avgbs_12069    avgbs_219317    avgbs_45367    avgbs_103941    avgbs_103985    avgbs_210633    avgbs_60505    avgbs_114307    avgbs_33061    avgbs_57820    avgbs_93929    avgbs_119474    avgbs_230968    avgbs_231980    avgbs_85012    avgbs_232632    avgbs_40353    avgbs_64534    avgbs_229724    avgbs_16931    avgbs_202066    avgbs_126002    avgbs_8471    avgbs_60506    avgbs_40292    avgbs_85206    avgbs_118649    avgbs_81100    avgbs_61033    avgbs_98125    avgbs_98126    avgbs_98127    avgbs_88911    avgbs_100178    avgbs_101213    avgbs_108424    avgbs_111234    avgbs_236368    avgbs_200794    avgbs_242602    avgbs_73514    avgbs_55244    avgbs_44090    avgbs_6K_34263    avgbs_38626    avgbs_42855    avgbs_112394    avgbs_223810    avgbs_224221    avgbs_224643    avgbs_225334    avgbs_7389    avgbs_10251    avgbs_11840    avgbs_11841    avgbs_21896    avgbs_22368    avgbs_32993    avgbs_34289    avgbs_41964    avgbs_46915    avgbs_51424    avgbs_61264    avgbs_6K_73700    avgbs_84096    avgbs_91642    avgbs_97792    avgbs_100862    avgbs_104377    avgbs_113829    avgbs_119714    avgbs_119715    avgbs_119716    avgbs_223186    avgbs_223540    avgbs_223915    avgbs_224132    avgbs_224796    avgbs_225253    avgbs_225320    avgbs_225583    avgbs_225813    avgbs_9180    avgbs_49032    avgbs_53174    avgbs_87124    avgbs_114176    avgbs_114177    avgbs_224270    avgbs_6501    avgbs_55569    avgbs_80147    avgbs_99293    avgbs_57272    avgbs_61772    avgbs_224369   
   7C-17A 72 
  GMI_DS_CC7281_186    
 
  avgbs_72998    avgbs_200491    avgbs_90241    avgbs_110727    avgbs_40352    avgbs_78909    avgbs_61456    avgbs_87220    avgbs_99394    avgbs_99417    avgbs_455    avgbs_13183    avgbs_19131    avgbs_75884    avgbs_80570    avgbs_109509    avgbs_109510    avgbs_109950    avgbs_121156    avgbs_200972    avgbs_229108    avgbs_43328    avgbs_43329    avgbs_19070    avgbs_46563    avgbs_53496    avgbs_77471    avgbs_93503    avgbs_113579    avgbs_23091    avgbs_80152    avgbs_81045    avgbs_204985    avgbs_28090    avgbs_88460    avgbs_40241    avgbs_46631    avgbs_7713    avgbs_7714    avgbs_86970    avgbs_67407    avgbs_233409    avgbs_239770    avgbs_80604    avgbs_51071    avgbs_9741    avgbs_6K_88838    avgbs_117700    avgbs_241688    avgbs_207555    avgbs_234669    avgbs_12332    avgbs_235749    avgbs_87567    avgbs_100650    avgbs_121634    avgbs_77589    avgbs_235137    avgbs_85132    avgbs_233265    avgbs_91364    avgbs_234885    avgbs_242568    avgbs_232806    avgbs_234729    avgbs_242990    avgbs_21657    avgbs_714    avgbs_100643   
   7C-17A 73 
  GMI_ES01_c13834_134     GMI_ES17_c806_849    
 
  avgbs_103599    avgbs_87816    avgbs_66500    avgbs_66501    avgbs_93689    avgbs_104151    avgbs_217840    avgbs_23036    avgbs_108969    avgbs_26772    avgbs_119793    avgbs_8231    avgbs_105143    avgbs_105144    avgbs_105898    avgbs_105899    avgbs_229482    avgbs_13546    avgbs_37230    avgbs_37231    avgbs_37232    avgbs_37964    avgbs_53635    avgbs_77472    avgbs_229870    avgbs_203158    avgbs_230338    avgbs_231286    avgbs_120397    avgbs_13923    avgbs_31495    avgbs_49590    avgbs_109350    avgbs_109351    avgbs_125243    avgbs_240362    avgbs_244576    avgbs_236140    avgbs_243421    avgbs_52350    avgbs_5398    avgbs_59408    avgbs_59409    avgbs_223768    avgbs_17001    avgbs_80538    avgbs_80539    avgbs_84441    avgbs_87573    avgbs_201368    avgbs_223494    avgbs_225729    avgbs_4690    avgbs_5325    avgbs_6518    avgbs_6730    avgbs_9121    avgbs_11168    avgbs_12754    avgbs_12755    avgbs_13252    avgbs_15078    avgbs_15079    avgbs_17002    avgbs_19877    avgbs_30242    avgbs_30398    avgbs_31524    avgbs_37308    avgbs_41061    avgbs_44615    avgbs_6K_46341    avgbs_52809    avgbs_63417    avgbs_63418    avgbs_63552    avgbs_65239    avgbs_72543    avgbs_72646    avgbs_72647    avgbs_72648    avgbs_74193    avgbs_74537    avgbs_74538    avgbs_74539    avgbs_75480    avgbs_75481    avgbs_75694    avgbs_76085    avgbs_80011    avgbs_84972    avgbs_86762    avgbs_86763    avgbs_87539    avgbs_87540    avgbs_93809    avgbs_96691    avgbs_98917    avgbs_101707    avgbs_104256    avgbs_104257    avgbs_105900    avgbs_106354    avgbs_106867    avgbs_109740    avgbs_110502    avgbs_110503    avgbs_113802    avgbs_117177    avgbs_117450    avgbs_120492    avgbs_125325    avgbs_125773    avgbs_223315    avgbs_223326    avgbs_223425    avgbs_223511    avgbs_223661    avgbs_223819    avgbs_224077    avgbs_224178    avgbs_224186    avgbs_224510    avgbs_224595    avgbs_224713    avgbs_224752    avgbs_224886    avgbs_224970    avgbs_225082    avgbs_225097    avgbs_225116    avgbs_225151    avgbs_205623    avgbs_225215    avgbs_225269    avgbs_225460    avgbs_225554    avgbs_207662    avgbs_225743    avgbs_225789    avgbs_225837    avgbs_225852    avgbs_6K_835    avgbs_40454    avgbs_3091    avgbs_7843    avgbs_11523    avgbs_23664    avgbs_34408    avgbs_52792    avgbs_52793    avgbs_64020    avgbs_64021    avgbs_75391    avgbs_75392    avgbs_83970    avgbs_83971    avgbs_100808    avgbs_100809    avgbs_113325    avgbs_115802    avgbs_115803    avgbs_119792    avgbs_223464    avgbs_223647    avgbs_223680    avgbs_223904    avgbs_86262    avgbs_86263    avgbs_4742    avgbs_33358    avgbs_34368    avgbs_37607    avgbs_49929    avgbs_90826    avgbs_100501    avgbs_100502    avgbs_102177    avgbs_120890    avgbs_224367    avgbs_225141    avgbs_225241    avgbs_225476    avgbs_114884    avgbs_53986    avgbs_111873    avgbs_35931    avgbs_119046    avgbs_223929   
   7C-17A 74 
  GMI_ES02_c884_341    
 
  avgbs_81314    avgbs_92839    avgbs_222148    avgbs_118286    avgbs_30827    avgbs_78213    avgbs_96809    avgbs_96810    avgbs_230715    avgbs_22668    avgbs_53524    avgbs_116183    avgbs_48039    avgbs_48040    avgbs_39534   
   7C-17A 75 
  GMI_ES_CC7894_200    
 
  avgbs_119107    avgbs_28450    avgbs_41697    avgbs_51335    avgbs_51336    avgbs_65265    avgbs_105254    avgbs_219988    avgbs_218703    avgbs_70222    avgbs_222902    avgbs_71229    avgbs_71230    avgbs_58387    avgbs_230516    avgbs_39536    avgbs_4295    avgbs_234534   
   7C-17A 76 
 
 
  avgbs_78155    avgbs_217286    avgbs_220469    avgbs_70346    avgbs_222440    avgbs_219569    avgbs_32109    avgbs_51114    avgbs_95550    avgbs_105133    avgbs_57131    avgbs_39059    avgbs_236441    avgbs_108484    avgbs_108485    avgbs_108482    avgbs_108483   
   7C-17A 77 
 
 
  avgbs_20056    avgbs_220083    avgbs_18102    avgbs_18103    avgbs_18104    avgbs_35923    avgbs_36579    avgbs_49659    avgbs_49660    avgbs_49661    avgbs_6834    avgbs_92878    avgbs_217223    avgbs_217329    avgbs_221336    avgbs_14307    avgbs_14308    avgbs_21162    avgbs_43891    avgbs_43892    avgbs_93801    avgbs_232370    avgbs_3686    avgbs_62811    avgbs_118256    avgbs_118257    avgbs_118258    avgbs_228784    avgbs_231377    avgbs_33823    avgbs_114365    avgbs_51669    avgbs_243548    avgbs_203555    avgbs_19409    avgbs_235047    avgbs_207161    avgbs_24684   
   7C-17A 78 
  GMI_DS_oPt-17385_80    
 
  avgbs_50219    avgbs_90211    avgbs_97457    avgbs_124955    avgbs_221161    avgbs_72536    avgbs_114631    avgbs_220136    avgbs_222573    avgbs_11365    avgbs_55611    avgbs_70961    avgbs_5621    avgbs_5622    avgbs_22364    avgbs_22365    avgbs_30546    avgbs_30548    avgbs_35593    avgbs_58424    avgbs_95160    avgbs_106325    avgbs_221496    avgbs_222155    avgbs_222183    avgbs_222729    avgbs_110941    avgbs_218653    avgbs_28082    avgbs_100143    avgbs_67858    avgbs_67859    avgbs_67860    avgbs_70993    avgbs_89526    avgbs_114549    avgbs_123619    avgbs_232534    avgbs_18671    avgbs_77154    avgbs_77155    avgbs_102158    avgbs_10382    avgbs_47928    avgbs_9109    avgbs_104124    avgbs_8096    avgbs_89844    avgbs_31828    avgbs_235955    avgbs_108962    avgbs_105918    avgbs_106701    avgbs_115994    avgbs_241848    avgbs_243292    avgbs_20850    avgbs_4158    avgbs_74420    avgbs_118001    avgbs_122252    avgbs_241700    avgbs_51979    avgbs_237259    avgbs_18273    avgbs_43051    avgbs_66560    avgbs_237940    avgbs_99135    avgbs_240838    avgbs_18677    avgbs_18678    avgbs_18679    avgbs_25291    avgbs_71769    avgbs_10651    avgbs_75169    avgbs_94696    avgbs_97857    avgbs_115854    avgbs_118416    avgbs_201365    avgbs_234106    avgbs_40078    avgbs_61015    avgbs_89353    avgbs_89354    avgbs_19739    avgbs_29104    avgbs_34669    avgbs_61886    avgbs_84802    avgbs_92277    avgbs_92278    avgbs_101030    avgbs_101031    avgbs_104125    avgbs_109471    avgbs_118617    avgbs_118618    avgbs_119682    avgbs_236123    avgbs_204316    avgbs_204693    avgbs_240903    avgbs_45793    avgbs_112437    avgbs_242749    avgbs_2518    avgbs_36058    avgbs_45858    avgbs_77841    avgbs_116152    avgbs_122730    avgbs_123241    avgbs_123527    avgbs_123528    avgbs_124661    avgbs_126003    avgbs_235391    avgbs_236267    avgbs_244638    avgbs_226096    avgbs_236363    avgbs_23940    avgbs_43550    avgbs_43551    avgbs_46564    avgbs_46715    avgbs_49445    avgbs_53552    avgbs_66906    avgbs_90030    avgbs_104155    avgbs_107318    avgbs_107319    avgbs_107320    avgbs_113517    avgbs_113518    avgbs_113519    avgbs_119322    avgbs_122663    avgbs_122664    avgbs_124682    avgbs_238145    avgbs_200887    avgbs_96842    avgbs_96843    avgbs_118127    avgbs_5620    avgbs_21424    avgbs_22010    avgbs_48775    avgbs_49603    avgbs_49604    avgbs_111846    avgbs_122946    avgbs_126260    avgbs_126261    avgbs_233436    avgbs_235329    avgbs_202731    avgbs_203776    avgbs_244396    avgbs_241928    avgbs_34447    avgbs_61014    avgbs_66482    avgbs_76065    avgbs_96539    avgbs_235770    avgbs_238980    avgbs_9322    avgbs_75974    avgbs_238971    avgbs_243872    avgbs_77494    avgbs_47502    avgbs_47503    avgbs_101511    avgbs_216230    avgbs_236365    avgbs_10307    avgbs_10308    avgbs_109003    avgbs_75341    avgbs_33317    avgbs_33318    avgbs_223317    avgbs_224103    avgbs_225590    avgbs_212816   
   7C-17A 79 
 
 
  avgbs_1842    avgbs_26340    avgbs_111421    avgbs_105996    avgbs_91838    avgbs_26375    avgbs_26376    avgbs_219632    avgbs_80421    avgbs_220609    avgbs_47327    avgbs_56546    avgbs_56547    avgbs_56548    avgbs_56549    avgbs_218831    avgbs_21420    avgbs_34896    avgbs_32288    avgbs_74973    avgbs_49858    avgbs_92362    avgbs_223642    avgbs_3928    avgbs_21818    avgbs_223654    avgbs_97139    avgbs_231864    avgbs_43721    avgbs_208140    avgbs_32287    avgbs_96959    avgbs_16487    avgbs_33250    avgbs_6331    avgbs_37352    avgbs_2771    avgbs_2772    avgbs_63211    avgbs_63212    avgbs_224751    avgbs_14156    avgbs_124862    avgbs_223373    avgbs_201829    avgbs_225832    avgbs_104359   
   7C-17A 80 
 
 
  avgbs_32184    avgbs_48813    avgbs_34486    avgbs_6K_81954    avgbs_115666    avgbs_115667    avgbs_115668    avgbs_220073    avgbs_6448    avgbs_4486    avgbs_21778    avgbs_218496    avgbs_80587    avgbs_3831    avgbs_11629    avgbs_107399    avgbs_201155    avgbs_223435    avgbs_223489    avgbs_224789    avgbs_52060    avgbs_52061    avgbs_79452    avgbs_18134    avgbs_93579    avgbs_76302    avgbs_76304    avgbs_34226    avgbs_55740    avgbs_203847    avgbs_124178    avgbs_59509    avgbs_64535    avgbs_64542    avgbs_73016    avgbs_73017    avgbs_223897    avgbs_223216    avgbs_225715    avgbs_6K_22793    avgbs_25484    avgbs_61787    avgbs_223550    avgbs_223697    avgbs_223815    avgbs_225399    avgbs_116102   
   7C-17A 81 
 
 
  avgbs_606    avgbs_6K_33046    avgbs_81313    avgbs_91019    avgbs_124481    avgbs_223092    avgbs_20941    avgbs_51388    avgbs_119591    avgbs_119592    avgbs_123715    avgbs_223058    avgbs_25164    avgbs_28490    avgbs_218276    avgbs_119934    avgbs_231959    avgbs_82636    avgbs_66917    avgbs_203129    avgbs_62810    avgbs_105778    avgbs_109755    avgbs_110863    avgbs_204399    avgbs_104152    avgbs_104153    avgbs_107351    avgbs_66192    avgbs_66193    avgbs_76235    avgbs_241758    avgbs_112764   
   7C-17A 82 
 
 
  avgbs_30875    avgbs_30876    avgbs_35455    avgbs_91262    avgbs_91263    avgbs_203899    avgbs_221211    avgbs_42343    avgbs_22209    avgbs_51729    avgbs_52527    avgbs_63899    avgbs_63900    avgbs_63901    avgbs_70347    avgbs_229114    avgbs_114366    avgbs_28125    avgbs_232736    avgbs_62912    avgbs_76750    avgbs_26610    avgbs_26611    avgbs_80422    avgbs_80423    avgbs_84976    avgbs_84977    avgbs_84978    avgbs_243043    avgbs_19437    avgbs_125929    avgbs_204044    avgbs_18573    avgbs_202265    avgbs_72599    avgbs_203639   
   7C-17A 83 
 
 
  avgbs_75039    avgbs_81    avgbs_122902    avgbs_18007    avgbs_85643   
   7C-17A 84 
  GMI_ES15_c8064_341    
 
  avgbs_11845    avgbs_108575    avgbs_6K_70801    avgbs_83553    avgbs_220548    avgbs_222756    avgbs_6K_8678    avgbs_30462    avgbs_118687    avgbs_220170    avgbs_52431    avgbs_8659    avgbs_219813    avgbs_84358    avgbs_43331    avgbs_43332    avgbs_123293    avgbs_223031    avgbs_52260    avgbs_71317    avgbs_71343    avgbs_71705    avgbs_79775    avgbs_217656    avgbs_220088    avgbs_109081    avgbs_14937    avgbs_25791    avgbs_77097    avgbs_217128    avgbs_34895    avgbs_39907    avgbs_39908    avgbs_120042    avgbs_35807    avgbs_80    avgbs_7978    avgbs_13993    avgbs_54130    avgbs_54131    avgbs_70338    avgbs_70339    avgbs_70340    avgbs_111422    avgbs_111444    avgbs_14297    avgbs_4841    avgbs_228834    avgbs_38434    avgbs_34894    avgbs_68191    avgbs_99770    avgbs_99771    avgbs_108418    avgbs_104959    avgbs_48812    avgbs_48814    avgbs_207908    avgbs_67289    avgbs_77249    avgbs_100169    avgbs_25185    avgbs_62497    avgbs_62498    avgbs_206629    avgbs_105880    avgbs_105881    avgbs_105882    avgbs_53164    avgbs_18093    avgbs_18094    avgbs_5256    avgbs_5257    avgbs_58788    avgbs_207260    avgbs_34949    avgbs_73982    avgbs_58078    avgbs_231035    avgbs_36156    avgbs_93009   
   7C-17A 85 
 
 
  avgbs_218479    avgbs_52690    avgbs_204636    avgbs_66628    avgbs_204220    avgbs_6176   
   7C-17A 86 
 
 
  avgbs_121838    avgbs_23421    avgbs_35315   
   7C-17A 87 
 
 
  avgbs_93510   
   7C-17A 88 
 
 
  avgbs_117661    avgbs_124679   
   7C-17A 89 
 
 
  avgbs_216658    avgbs_225509   
   7C-17A 91 
 
 
  avgbs_204932   
   7C-17A 92 
 
 
  avgbs_225812   
 
 
   Chromosome 17A-7C    Chr   Pos   Framework   Placed SNP  Placed GBS  
   17A-7C -17 
 
 
  avgbs_18591   
   17A-7C -16 
 
 
  avgbs_51373    avgbs_206055   
   17A-7C -11 
 
 
  avgbs_41751   
   17A-7C -4 
 
 
  avgbs_70430    avgbs_53102    avgbs_53105   
   17A-7C -3 
 
 
  avgbs_62423    avgbs_62424    avgbs_62425    avgbs_118069    avgbs_200874   
   17A-7C -1 
 
 
  avgbs_72759    avgbs_200489   
   17A-7C 0 
  GMI_ES17_c12316_291    
 
  avgbs_115256    avgbs_48075    avgbs_90900   
   17A-7C 1 
 
 
  avgbs_68625    avgbs_219875   
   17A-7C 2 
  GMI_ES01_c15628_381    
 
  avgbs_85861    avgbs_218354   
   17A-7C 3 
  GMI_ES02_c3577_672    
 
  avgbs_92030    avgbs_23238    avgbs_8098    avgbs_29192    avgbs_43443    avgbs_63099    avgbs_120452    avgbs_203402    avgbs_214891    avgbs_215156    avgbs_200894    avgbs_1259    avgbs_100361    avgbs_5195    avgbs_40580    avgbs_2095    avgbs_70334    avgbs_210188    avgbs_102695    avgbs_241849    avgbs_10852    avgbs_67099    avgbs_20494    avgbs_64961    avgbs_73887    avgbs_107204    avgbs_113337    avgbs_39442    avgbs_15013    avgbs_122203    avgbs_3180    avgbs_73597    avgbs_83765    avgbs_48366    avgbs_113178    avgbs_48367    avgbs_236924    avgbs_237511    avgbs_77074    avgbs_121262    avgbs_121263    avgbs_201326    avgbs_235305    avgbs_64933    avgbs_234045    avgbs_242395    avgbs_49346    avgbs_58550    avgbs_244625    avgbs_55107    avgbs_24608    avgbs_46446    avgbs_77089    avgbs_206874    avgbs_9125    avgbs_105025    avgbs_113619    avgbs_12524    avgbs_208975    avgbs_213729    avgbs_100763    avgbs_30627    avgbs_71486    avgbs_235533    avgbs_204427    avgbs_113491    avgbs_38856    avgbs_85912    avgbs_238513    avgbs_6K_30551    avgbs_243870    avgbs_46960    avgbs_16316    avgbs_235495    avgbs_202269    avgbs_89619    avgbs_72246    avgbs_72247    avgbs_8522    avgbs_233850    avgbs_232945   
   17A-7C 4 
 
 
  avgbs_40665    avgbs_44208    avgbs_214565   
   17A-7C 5 
 
 
  avgbs_46949    avgbs_213309    avgbs_200815    avgbs_33439    avgbs_101386    avgbs_213830    avgbs_53101    avgbs_53103    avgbs_53104   
   17A-7C 6 
 
 
  avgbs_27228    avgbs_95700    avgbs_20958    avgbs_117434    avgbs_117435   
   17A-7C 7 
 
 
  avgbs_105597    avgbs_16317    avgbs_46961    avgbs_49754   
   17A-7C 8 
 
 
  avgbs_78579   
   17A-7C 9 
  GMI_ES01_c1638_111     GMI_ES15_c6348_420    
 
  avgbs_71071    avgbs_78159    avgbs_220047    avgbs_1233    avgbs_1347    avgbs_3017    avgbs_3018    avgbs_3019    avgbs_9737    avgbs_11300    avgbs_16619    avgbs_33120    avgbs_40741    avgbs_48923    avgbs_59531    avgbs_69352    avgbs_69353    avgbs_83490    avgbs_93831    avgbs_102196    avgbs_102197    avgbs_102198    avgbs_106408    avgbs_111031    avgbs_216779    avgbs_217302    avgbs_203553    avgbs_203667    avgbs_219033    avgbs_219926    avgbs_204417    avgbs_220708    avgbs_205087    avgbs_49099    avgbs_111046    avgbs_111052    avgbs_9981    avgbs_10348    avgbs_21821    avgbs_27700    avgbs_27701    avgbs_44276    avgbs_49899    avgbs_63122    avgbs_63404    avgbs_78145    avgbs_80711    avgbs_82180    avgbs_82311    avgbs_83931    avgbs_97464    avgbs_220466    avgbs_205882    avgbs_43421    avgbs_52239    avgbs_73844    avgbs_79123    avgbs_84809    avgbs_219105    avgbs_220202    avgbs_204871    avgbs_80815    avgbs_220692    avgbs_1290    avgbs_18171    avgbs_26104    avgbs_26105    avgbs_60313   
   17A-7C 10 
 
 
  avgbs_63872    avgbs_200426   
   17A-7C 11 
 
 
  avgbs_107296    avgbs_120263    avgbs_11605    avgbs_126125   
   17A-7C 13 
 
 
  avgbs_44805   
   17A-7C 14 
 
 
  avgbs_30320   
   17A-7C 15 
 
 
  avgbs_5585    avgbs_84172   
   17A-7C 16 
  GMI_ES01_c8121_663    
 
  avgbs_91013    avgbs_49720    avgbs_52749    avgbs_74831    avgbs_106428    avgbs_119237    avgbs_203556    avgbs_222160    avgbs_14503    avgbs_34656    avgbs_201278    avgbs_221670    avgbs_60589    avgbs_61893    avgbs_115293    avgbs_222606    avgbs_75662    avgbs_86872   
   17A-7C 17 
 
 
  avgbs_75663   
   17A-7C 18 
 
 
  avgbs_81851    avgbs_81852    avgbs_88450    avgbs_88451    avgbs_205250    avgbs_221502    avgbs_205994   
   17A-7C 19 
 
 
  avgbs_66003    avgbs_122658   
   17A-7C 20 
 
 
  avgbs_34071    avgbs_215561    avgbs_60588    avgbs_49076    avgbs_74777   
   17A-7C 21 
 
 
  avgbs_105404    avgbs_105405    avgbs_40867    avgbs_120292    avgbs_120293    avgbs_203256    avgbs_113920    avgbs_203980    avgbs_222538    avgbs_43947   
   17A-7C 22 
 
 
  avgbs_113363    avgbs_124274    avgbs_12549    avgbs_19759    avgbs_19760    avgbs_6K_93277    avgbs_218252    avgbs_18341    avgbs_27854    avgbs_217754    avgbs_95366    avgbs_51960    avgbs_64525    avgbs_64526    avgbs_27982    avgbs_103563    avgbs_103564   
   17A-7C 23 
 
 
  avgbs_53044    avgbs_120294    avgbs_5925    avgbs_63288    avgbs_216545    avgbs_115680    avgbs_15364   
   17A-7C 24 
 
 
  avgbs_82512    avgbs_90769   
   17A-7C 25 
 
 
  avgbs_217532    avgbs_89984    avgbs_11554   
   17A-7C 26 
 
 
  avgbs_12205    avgbs_12218    avgbs_99974   
   17A-7C 27 
 
 
  avgbs_23329    avgbs_43367    avgbs_97361    avgbs_97362    avgbs_98347    avgbs_98348    avgbs_118156    avgbs_218374    avgbs_219516    avgbs_221628    avgbs_81764    avgbs_3822   
   17A-7C 28 
 
 
  avgbs_218503    avgbs_220531    avgbs_98850    avgbs_112898    avgbs_110824    avgbs_110825    avgbs_14228    avgbs_26054    avgbs_26055    avgbs_26056   
   17A-7C 29 
 
 
  avgbs_54957    avgbs_87191   
   17A-7C 31 
 
 
  avgbs_35470   
   17A-7C 32 
 
 
  avgbs_35471   
   17A-7C 33 
 
 
  avgbs_222267    avgbs_13354    avgbs_82449    avgbs_107797    avgbs_220555    avgbs_222231   
   17A-7C 34 
 
 
  avgbs_222530    avgbs_124115    avgbs_13918    avgbs_33154    avgbs_41480    avgbs_102805   
   17A-7C 35 
 
 
  avgbs_87464    avgbs_87465    avgbs_217053    avgbs_801    avgbs_10582    avgbs_14538    avgbs_17673    avgbs_51322    avgbs_87458    avgbs_103008    avgbs_220993    avgbs_46792    avgbs_115700    avgbs_216923    avgbs_203456    avgbs_221816    avgbs_222621    avgbs_23903    avgbs_90713    avgbs_15177   
   17A-7C 36 
 
 
  avgbs_87392    avgbs_220987    avgbs_19021    avgbs_52390    avgbs_91726    avgbs_91727    avgbs_124762    avgbs_124763    avgbs_125252    avgbs_125253    avgbs_222787    avgbs_222994    avgbs_90254    avgbs_90255    avgbs_90256    avgbs_90257   
   17A-7C 38 
  GMI_ES17_c2276_224    
 
  avgbs_73648    avgbs_73649    avgbs_73650    avgbs_52405    avgbs_63379    avgbs_5334    avgbs_216503   
   17A-7C 41 
 
 
  avgbs_13037   
   17A-7C 45 
  GMI_ES02_c17364_288    
 
  avgbs_28792    avgbs_217811    avgbs_28784    avgbs_221931    avgbs_3434    avgbs_3435    avgbs_12165    avgbs_43073    avgbs_43074    avgbs_66466    avgbs_213318    avgbs_12509    avgbs_17720    avgbs_19158    avgbs_19421    avgbs_20875    avgbs_21947    avgbs_28568    avgbs_51684    avgbs_58518    avgbs_68930    avgbs_68931    avgbs_76844    avgbs_82543    avgbs_87075    avgbs_94687    avgbs_95051    avgbs_95481    avgbs_95482    avgbs_114157    avgbs_114158    avgbs_114159    avgbs_114798    avgbs_228688    avgbs_229200    avgbs_213563    avgbs_230124    avgbs_230353    avgbs_215369    avgbs_225219    avgbs_225420    avgbs_3658    avgbs_17325    avgbs_17326    avgbs_17327    avgbs_31012    avgbs_31013    avgbs_55182    avgbs_6K_53244    avgbs_57618    avgbs_65924    avgbs_87344    avgbs_91669    avgbs_105211    avgbs_105212    avgbs_105213    avgbs_108306    avgbs_109934    avgbs_114799    avgbs_117629    avgbs_119323    avgbs_119935    avgbs_119936    avgbs_125877    avgbs_125880    avgbs_230249    avgbs_230562    avgbs_200616    avgbs_232277    avgbs_207551    avgbs_14769    avgbs_52712    avgbs_104383    avgbs_104384    avgbs_104385    avgbs_106583    avgbs_231782    avgbs_19420    avgbs_6K_22064    avgbs_118508    avgbs_101118    avgbs_101119    avgbs_1599    avgbs_7627    avgbs_15157    avgbs_37976    avgbs_22182    avgbs_28910    avgbs_34758    avgbs_37638    avgbs_85304    avgbs_209585   
   17A-7C 46 
  GMI_ES02_c18066_185    
 
  avgbs_117211    avgbs_24717    avgbs_92450    avgbs_85656    avgbs_90899    avgbs_104177    avgbs_104267    avgbs_104268    avgbs_104269    avgbs_106367    avgbs_31948   
   17A-7C 47 
 
 
  avgbs_90462   
   17A-7C 48 
 
 
  avgbs_216234    avgbs_222801    avgbs_87193    avgbs_108618    avgbs_108729    avgbs_16095    avgbs_27289    avgbs_93659    avgbs_109013    avgbs_109014    avgbs_217715    avgbs_221374    avgbs_206639   
   17A-7C 49 
 
 
  avgbs_3908    avgbs_3909    avgbs_74872    avgbs_42799    avgbs_42800    avgbs_222157    avgbs_82854   
   17A-7C 50 
 
 
  avgbs_97461    avgbs_97740    avgbs_116371   
   17A-7C 51 
 
 
  avgbs_99068    avgbs_9721    avgbs_30777    avgbs_64473    avgbs_64474    avgbs_72236    avgbs_222037   
   17A-7C 52 
 
 
  avgbs_99896    avgbs_99897    avgbs_216604    avgbs_221392    avgbs_216546    avgbs_44792    avgbs_84773    avgbs_93928    avgbs_220105   
   17A-7C 53 
 
 
  avgbs_219368    avgbs_221790   
   17A-7C 54 
 
 
  avgbs_75157   
   17A-7C 55 
 
 
  avgbs_102966   
   17A-7C 56 
 
 
  avgbs_41062    avgbs_41063    avgbs_19100    avgbs_76051   
   17A-7C 58 
 
 
  avgbs_44138    avgbs_44139    avgbs_86364    avgbs_28811   
   17A-7C 59 
 
 
  avgbs_105993    avgbs_76816    avgbs_76817    avgbs_104419    avgbs_216757   
   17A-7C 60 
 
 
  avgbs_75418    avgbs_102401    avgbs_122314    avgbs_219070    avgbs_220296    avgbs_221958   
   17A-7C 62 
 
 
  avgbs_220198    avgbs_73799   
 
 
   Chromosome 8A    Chr   Pos   Framework   Placed SNP  Placed GBS  
   8A -15 
 
 
  avgbs_92410   
   8A -7 
 
 
  avgbs_92370    avgbs_206323   
   8A -4 
 
 
  avgbs_16939    avgbs_16940    avgbs_16941   
   8A -3 
 
 
  avgbs_126281   
   8A -1 
 
 
  avgbs_121008    avgbs_121009    avgbs_121010   
   8A 0 
  GMI_ES15_lrc19149_99    
 
  avgbs_110838    avgbs_110839    avgbs_54122    avgbs_6617    avgbs_124488    avgbs_6885    avgbs_9919    avgbs_76101    avgbs_119234    avgbs_119657    avgbs_72269    avgbs_44304   
   8A 1 
 
 
  avgbs_67530    avgbs_61431    avgbs_227113    avgbs_6980    avgbs_6981   
   8A 2 
 
 
  avgbs_59180    avgbs_230544    avgbs_205679    avgbs_70665   
   8A 3 
 
 
  avgbs_77888    avgbs_77889    avgbs_5494    avgbs_5495    avgbs_6414    avgbs_6415    avgbs_6416    avgbs_12993    avgbs_15156    avgbs_42542    avgbs_42543    avgbs_93094    avgbs_30972   
   8A 4 
 
 
  avgbs_52859    avgbs_42020    avgbs_110835    avgbs_110836    avgbs_202613    avgbs_229680    avgbs_67344    avgbs_120509   
   8A 5 
 
 
  avgbs_90621    avgbs_90622    avgbs_43412    avgbs_46219    avgbs_230033    avgbs_204921   
   8A 6 
 
 
  avgbs_53486    avgbs_65131    avgbs_230739   
   8A 7 
 
 
  avgbs_225724   
   8A 10 
  GMI_ES17_c9652_803     GMI_DS_CC8640_82     GMI_ES_CC15237_134    
  GMI_ES15_c11428_323    GMI_ES01_c2320_569   
  avgbs_56776    avgbs_202178    avgbs_207281    avgbs_107304    avgbs_97912    avgbs_124121    avgbs_203333    avgbs_105330    avgbs_123270    avgbs_14729    avgbs_104229    avgbs_104230    avgbs_122579    avgbs_15630    avgbs_21312    avgbs_65263    avgbs_104228    avgbs_14265    avgbs_14266    avgbs_122580    avgbs_9793    avgbs_38127    avgbs_73330    avgbs_77227    avgbs_106635    avgbs_236387    avgbs_49167    avgbs_79690    avgbs_79691    avgbs_206008    avgbs_243189    avgbs_33903    avgbs_78994    avgbs_83757    avgbs_99598    avgbs_101634    avgbs_101635    avgbs_237154    avgbs_11028    avgbs_11029    avgbs_40774    avgbs_59653    avgbs_237795    avgbs_14471    avgbs_236801    avgbs_95835    avgbs_240839    avgbs_4396    avgbs_58655    avgbs_97959    avgbs_5823    avgbs_101849    avgbs_104079    avgbs_17041    avgbs_2628    avgbs_2629    avgbs_2630    avgbs_94170   
   8A 11 
 
 
  avgbs_80185   
   8A 12 
 
 
  avgbs_5915    avgbs_22981    avgbs_235933    avgbs_14258    avgbs_14259    avgbs_14260    avgbs_104905    avgbs_244678    avgbs_7246    avgbs_19900    avgbs_67461    avgbs_117342    avgbs_123585    avgbs_39118    avgbs_39119    avgbs_6K_7386    avgbs_94576    avgbs_113748    avgbs_120240    avgbs_124242    avgbs_124979    avgbs_214758    avgbs_240130    avgbs_7685    avgbs_8077    avgbs_60724    avgbs_78080    avgbs_122675    avgbs_125496    avgbs_201264    avgbs_204945    avgbs_53075    avgbs_99599    avgbs_125881    avgbs_239025    avgbs_40937   
   8A 15 
  GMI_DS_CC9972_92    
 
  avgbs_21395    avgbs_97668    avgbs_97669    avgbs_2689    avgbs_113747    avgbs_20598    avgbs_204309    avgbs_72026    avgbs_235133    avgbs_1477    avgbs_1478    avgbs_11026    avgbs_11027    avgbs_16004    avgbs_28776    avgbs_18207    avgbs_18208    avgbs_20842    avgbs_65827    avgbs_123403    avgbs_124330    avgbs_236988    avgbs_64397    avgbs_44549   
   8A 16 
  GMI_ES01_c24051_67    
 
 
   8A 17 
  GMI_DS_CC8923_203    
  GMI_ES01_c8015_409   
  avgbs_16627    avgbs_109724    avgbs_16353    avgbs_16354    avgbs_14488    avgbs_200419    avgbs_77159    avgbs_77160    avgbs_89776    avgbs_31356    avgbs_47314    avgbs_75114    avgbs_51045    avgbs_74487    avgbs_19079    avgbs_19080    avgbs_239146    avgbs_71308    avgbs_117754    avgbs_117755    avgbs_200871    avgbs_36750    avgbs_47336    avgbs_68000    avgbs_71143    avgbs_3452    avgbs_21678    avgbs_117799    avgbs_200872    avgbs_214    avgbs_109422    avgbs_40366    avgbs_43751    avgbs_114567    avgbs_243929    avgbs_106680    avgbs_125372    avgbs_9976    avgbs_12336    avgbs_34855    avgbs_40711    avgbs_40712    avgbs_42243    avgbs_78289    avgbs_78290    avgbs_203584    avgbs_79753    avgbs_234647    avgbs_1781    avgbs_9513    avgbs_6K_20626    avgbs_32304    avgbs_99877    avgbs_99878    avgbs_100915    avgbs_124250    avgbs_203511    avgbs_200339    avgbs_207081    avgbs_232975    avgbs_106507    avgbs_46569    avgbs_46570    avgbs_46571    avgbs_48750    avgbs_84958    avgbs_108598    avgbs_203520    avgbs_205147    avgbs_243912    avgbs_22670    avgbs_75389    avgbs_116525    avgbs_202634    avgbs_207477    avgbs_218990    avgbs_17303    avgbs_66687    avgbs_72396    avgbs_41172    avgbs_41173    avgbs_120078    avgbs_117011    avgbs_117012    avgbs_105785    avgbs_105786    avgbs_235291    avgbs_96340    avgbs_230219    avgbs_236249    avgbs_42515    avgbs_98659    avgbs_98660    avgbs_123638    avgbs_37851    avgbs_37852    avgbs_18644    avgbs_223325    avgbs_1344    avgbs_85176    avgbs_225003   
   8A 18 
  GMI_ES02_c16344_816    
 
  avgbs_113675    avgbs_43372    avgbs_111053    avgbs_33692    avgbs_33693    avgbs_36359    avgbs_3740    avgbs_36561    avgbs_36562    avgbs_76285    avgbs_200526    avgbs_225801    avgbs_97896    avgbs_97897    avgbs_225834    avgbs_12159    avgbs_125455    avgbs_125456    avgbs_77891    avgbs_74832   
   8A 19 
 
 
  avgbs_13895    avgbs_54461    avgbs_83052    avgbs_106815    avgbs_224304    avgbs_224513    avgbs_107859    avgbs_200510    avgbs_38354    avgbs_124723    avgbs_11576   
   8A 20 
  GMI_DS_CC10461_118     GMI_ES02_c11842_367     GMI_ES_CC9711_432    
 
  avgbs_48688    avgbs_79682    avgbs_111048    avgbs_201787    avgbs_230596    avgbs_231231    avgbs_1767    avgbs_19291    avgbs_29039    avgbs_47866    avgbs_73232    avgbs_75162    avgbs_75163    avgbs_82594    avgbs_120222    avgbs_120342    avgbs_223206    avgbs_229928    avgbs_200493    avgbs_205330    avgbs_232731    avgbs_1790    avgbs_1791    avgbs_71142    avgbs_109612    avgbs_119399    avgbs_210497    avgbs_231242    avgbs_101899    avgbs_241724    avgbs_209181    avgbs_120477    avgbs_57592    avgbs_244229    avgbs_104901    avgbs_5199    avgbs_66683    avgbs_78358    avgbs_46782    avgbs_119033    avgbs_119034    avgbs_124722    avgbs_98161    avgbs_98163    avgbs_98165    avgbs_86736    avgbs_44571   
   8A 21 
  GMI_ES01_c8817_242     GMI_ES17_c1115_127     GMI_ES01_c16239_143     GMI_ES17_c2941_220     GMI_ES01_c9236_115     GMI_ES02_c2898_314     GMI_ES01_lrc28711_407     GMI_ES01_c10033_104    
 
  avgbs_69420    avgbs_43353    avgbs_82791    avgbs_242803    avgbs_69305    avgbs_69306    avgbs_243947    avgbs_43902    avgbs_244136   
   8A 22 
 
 
  avgbs_5198   
   8A 23 
 
 
  avgbs_242246    avgbs_6818    avgbs_203568    avgbs_109746   
   8A 24 
 
 
  avgbs_9192    avgbs_9193    avgbs_236394   
   8A 25 
 
 
  avgbs_235435    avgbs_53930    avgbs_31255    avgbs_31256   
   8A 26 
 
 
  avgbs_1818    avgbs_8003   
   8A 27 
 
 
  avgbs_54822    avgbs_116951   
   8A 29 
 
 
  avgbs_111251    avgbs_44878    avgbs_44879   
   8A 31 
 
 
  avgbs_112823    avgbs_63342    avgbs_200418   
   8A 32 
 
 
  avgbs_7608    avgbs_88083   
   8A 33 
 
 
  avgbs_607    avgbs_200986    avgbs_1782    avgbs_1783    avgbs_33   
   8A 34 
 
 
  avgbs_16186    avgbs_119392    avgbs_124469    avgbs_124470    avgbs_20566   
   8A 35 
 
 
  avgbs_6683    avgbs_85434    avgbs_201362    avgbs_39562    avgbs_13352    avgbs_88898    avgbs_88899   
   8A 40 
  GMI_ES02_c38800_418    
 
  avgbs_82421    avgbs_215037    avgbs_2868    avgbs_49285    avgbs_49286    avgbs_99037    avgbs_76631    avgbs_12681    avgbs_64197    avgbs_103428    avgbs_46208    avgbs_46209    avgbs_46264    avgbs_78037    avgbs_89128    avgbs_200399    avgbs_209926    avgbs_17039    avgbs_89503    avgbs_99127    avgbs_52057   
   8A 42 
 
 
  avgbs_57500    avgbs_110447   
   8A 43 
 
 
  avgbs_12543    avgbs_216946   
   8A 46 
 
 
  avgbs_15857    avgbs_15858   
   8A 47 
  GMI_ES17_c5762_827    
 
  avgbs_45894    avgbs_78480    avgbs_220486    avgbs_51137    avgbs_76187    avgbs_76188    avgbs_70436    avgbs_63368    avgbs_63369    avgbs_243661    avgbs_71459    avgbs_207821    avgbs_67228    avgbs_67229    avgbs_67230    avgbs_203270    avgbs_82866    avgbs_47207    avgbs_117513    avgbs_19064    avgbs_12679    avgbs_79728    avgbs_79729    avgbs_202191    avgbs_49653    avgbs_24067    avgbs_239301    avgbs_233789    avgbs_116439    avgbs_240858    avgbs_18839    avgbs_95694    avgbs_95695    avgbs_233521    avgbs_108874    avgbs_14666    avgbs_19072    avgbs_79541    avgbs_201762    avgbs_235003    avgbs_200920    avgbs_207460    avgbs_4923    avgbs_55270    avgbs_93492    avgbs_93493    avgbs_93494    avgbs_100902    avgbs_100903    avgbs_123185    avgbs_14951    avgbs_88818    avgbs_123563    avgbs_61677    avgbs_91645    avgbs_205873    avgbs_102867    avgbs_236898    avgbs_206419    avgbs_242491    avgbs_207745    avgbs_234964    avgbs_213565    avgbs_49391    avgbs_32192    avgbs_84084    avgbs_16093    avgbs_16094    avgbs_57298    avgbs_57299    avgbs_239986    avgbs_71750    avgbs_71751    avgbs_244329    avgbs_9018    avgbs_30897    avgbs_37637    avgbs_233919    avgbs_60055    avgbs_60056    avgbs_234065    avgbs_60639    avgbs_60640    avgbs_239374    avgbs_207602    avgbs_68104    avgbs_68105    avgbs_25427    avgbs_33639   
   8A 50 
 
 
  avgbs_60894    avgbs_60895   
   8A 51 
 
 
  avgbs_90097    avgbs_122528    avgbs_219798   
   8A 52 
 
 
  avgbs_222602    avgbs_219697   
   8A 53 
  GMI_ES15_c285_271    
 
  avgbs_94851    avgbs_108201    avgbs_108202    avgbs_116595    avgbs_210523    avgbs_6K_55778    avgbs_214594    avgbs_114293    avgbs_55491    avgbs_37124    avgbs_213623    avgbs_204802    avgbs_4903    avgbs_4904    avgbs_53931    avgbs_73231    avgbs_26328   
   8A 54 
 
 
  avgbs_93163    avgbs_211720   
   8A 55 
 
 
  avgbs_57332   
   8A 57 
 
 
  avgbs_115138   
   8A 62 
  GMI_ES17_c15132_382     GMI_ES02_c15926_519     GMI_ES15_c5280_161     GMI_ES17_c11418_547    
 
  avgbs_51797    avgbs_49753    avgbs_68959    avgbs_85443    avgbs_87053    avgbs_114707    avgbs_118397    avgbs_200457    avgbs_215125    avgbs_215888    avgbs_1592    avgbs_6169    avgbs_10378    avgbs_10379    avgbs_25253    avgbs_39917    avgbs_46063    avgbs_46064    avgbs_51040    avgbs_53570    avgbs_75478    avgbs_114818    avgbs_115352    avgbs_124138    avgbs_124882    avgbs_124883    avgbs_124884    avgbs_216978    avgbs_217425    avgbs_220300    avgbs_222608    avgbs_222984    avgbs_108334    avgbs_17894    avgbs_54636    avgbs_57331    avgbs_9977    avgbs_9978    avgbs_9979    avgbs_203286    avgbs_17209    avgbs_50276    avgbs_52264    avgbs_96750    avgbs_63126    avgbs_90501    avgbs_123484    avgbs_214562    avgbs_231197    avgbs_232079    avgbs_216130    avgbs_6K_13607    avgbs_75475    avgbs_111531    avgbs_117862    avgbs_223507    avgbs_230211    avgbs_203668    avgbs_96751    avgbs_231847    avgbs_52246    avgbs_6864    avgbs_58037    avgbs_58038    avgbs_58039    avgbs_100040    avgbs_229869    avgbs_232629    avgbs_35397    avgbs_231886    avgbs_85949    avgbs_229217    avgbs_77488    avgbs_230476    avgbs_38640    avgbs_60072   
   8A 63 
  GMI_ES01_c10387_303    
 
  avgbs_3474    avgbs_31114    avgbs_66105    avgbs_222667   
   8A 65 
 
 
  avgbs_122733    avgbs_28056    avgbs_50275    avgbs_61499   
   8A 66 
 
 
  avgbs_69360    avgbs_69361    avgbs_28055    avgbs_97261    avgbs_229588    avgbs_124910   
   8A 67 
 
 
  avgbs_120347   
   8A 68 
  GMI_DS_A3_489_351
[truncated: 425,187 more chars]
